# The α-tertiary amine motif drives remarkable selectivity for Pd-catalyzed carbonylation of β-methylene C–H bonds†
†Electronic supplementary information (ESI) available: Experimental procedures, characterization data and kinetic details. CCDC 1570476. For ESI and crystallographic data in CIF or other electronic format see DOI: 10.1039/c7sc03876c


**DOI:** 10.1039/c7sc03876c

**Published:** 2017-10-09

**Authors:** Kirsten F. Hogg, Aaron Trowbridge, Andrea Alvarez-Pérez, Matthew J. Gaunt

**Affiliations:** a Department of Chemistry , University of Cambridge , Lensfield Road , Cambridge , UK . Email: mjg32@cam.ac.uk

## Abstract

The selective C–H carbonylation of methylene bonds in the presence of traditionally more reactive methyl C–H and C(sp^2^)–H bonds in α-tertiary amines is reported. The exceptional selectivity is driven by the bulky α-tertiary amine motif, which we hypothesise orientates the activating C–H bond proximal to Pd in order to avoid an unfavourable steric clash with a second α-tertiary amine on the Pd centre, promoting preferential cyclopalladation at the methylene position. The reaction tolerates a range of structurally interesting and synthetically versatile functional groups, delivering the corresponding β-lactam products in good to excellent yields.

## Abstract 2

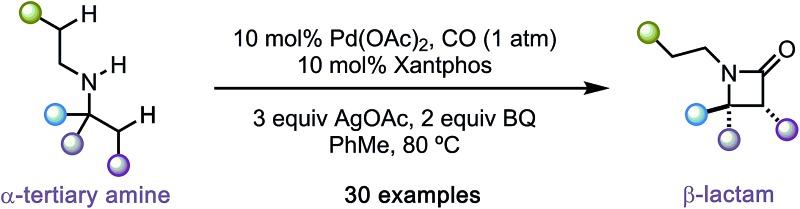
The selective C–H carbonylation of methylene bonds, in the presence of traditionally more reactive methyl C–H and C(sp^2^)–H bonds, in α-tertiary amines is reported.

## 

Methods that enable the catalytic functionalization of unreactive aliphatic C–H bonds have great potential in streamlining the synthesis of complex molecules such as natural products or medicinal agents.^1^ However, these molecules contain many types of C–H bond, each with a subtly different reactivity that is often influenced by an intricate interplay of factors including steric, inductive and conductive effects, and sometimes innate strain.^2^ As a result, catalytic processes that target certain C–H bonds are an important goal for chemical synthesis, and one that continues to inspire intense research effort.^3^

Arguably, the most common strategy employed for selective C–H activation involves the use of palladium(ii) catalysts, directed to a specific position by a resident polar functional group.^4^ Known as cyclopalladation, this activation mode most commonly targets the γ-C–H bond with respect to the directing group, to form a 5-membered ring intermediate from which further reaction takes place to install the new functionality. In most cases, the directing motifs needed to facilitate the C–H activation are bespoke auxiliaries or tailored protecting groups that need to be added to (and removed from) an intrinsic functionality of the parent molecule.^5^ While the use of auxiliaries has enabled many types of C–H activations, by contrast, the number of related transformations directed by functional groups that are native to aliphatic molecules (carboxylic acids, amines, hydroxyl groups) is more limited, despite the emergence of some important recent examples.^6^

Recently, we reported a new activation mode for C–H carbonylation of unprotected aliphatic secondary amines to form tertiary β-lactams.^7^ In contrast to other methods, the C–H activation step takes place at the β-C–H bond to the directing nitrogen functionality.^8^ This change in selectivity is brought about because the reaction follows a pathway that is distinct from classical cyclopalladation-mediated reactions. Rather than C–H activation preceding the CO insertion step, the new pathway uses an amine bound palladium(ii) carboxylate to first engage CO to form a carbamoyl–Pd(ii) complex. By virtue of CO already being inserted between the amine and the Pd(ii) centre, C–H activation *via* a 5-membered ring transition state now takes place at the β-C–H bond with respect to the resident amine motif. We have shown, firstly, that a wide range of aliphatic amines displaying α-branched methyl groups undergo β-C–H carbonylation to the corresponding β-lactams.^7^ Secondly, we found that in the absence of suitably disposed methyl groups, the C–H carbonylation was able to target the β-methylene C–H bond under slightly modified conditions to form *trans*-disubstituted β-lactams.^9^ The functional group tolerance exhibited by both of these C–H carbonylation processes is particularly notable and gives rise to a range of versatile and diverse β-lactam products.

During the course of our studies to further explore this carbonylation platform, we discovered a remarkable feature inherent to this C–H activation mode. α-Tertiary amines (ATAs) displaying both a β-methyl C–H bond and β-methylene C–H bond undergo exclusive carbonylation at the traditionally less reactive and more hindered methylene position.^10^ Central to the success of this selective C–H carbonylation is the presence of a fully substituted carbon atom on one side of the amine linkage, which steers the reaction to the C–H bond adjacent to this bulky structural feature (Scheme 1c). Herein, we report the development of a general C–H carbonylation exploiting this selectivity-inducing parameter. The ATA motif is widespread among natural products and pharmaceuticals displaying unique physiochemical properties (Scheme 2).^11^ However, due to the limited number of methods available in accessing these compounds, we believe that the direct functionalization of ATAs would provide convenient access to a range of molecular scaffolds that would be attractive to practitioners of synthetic and medicinal chemistry.

**Scheme 1:**
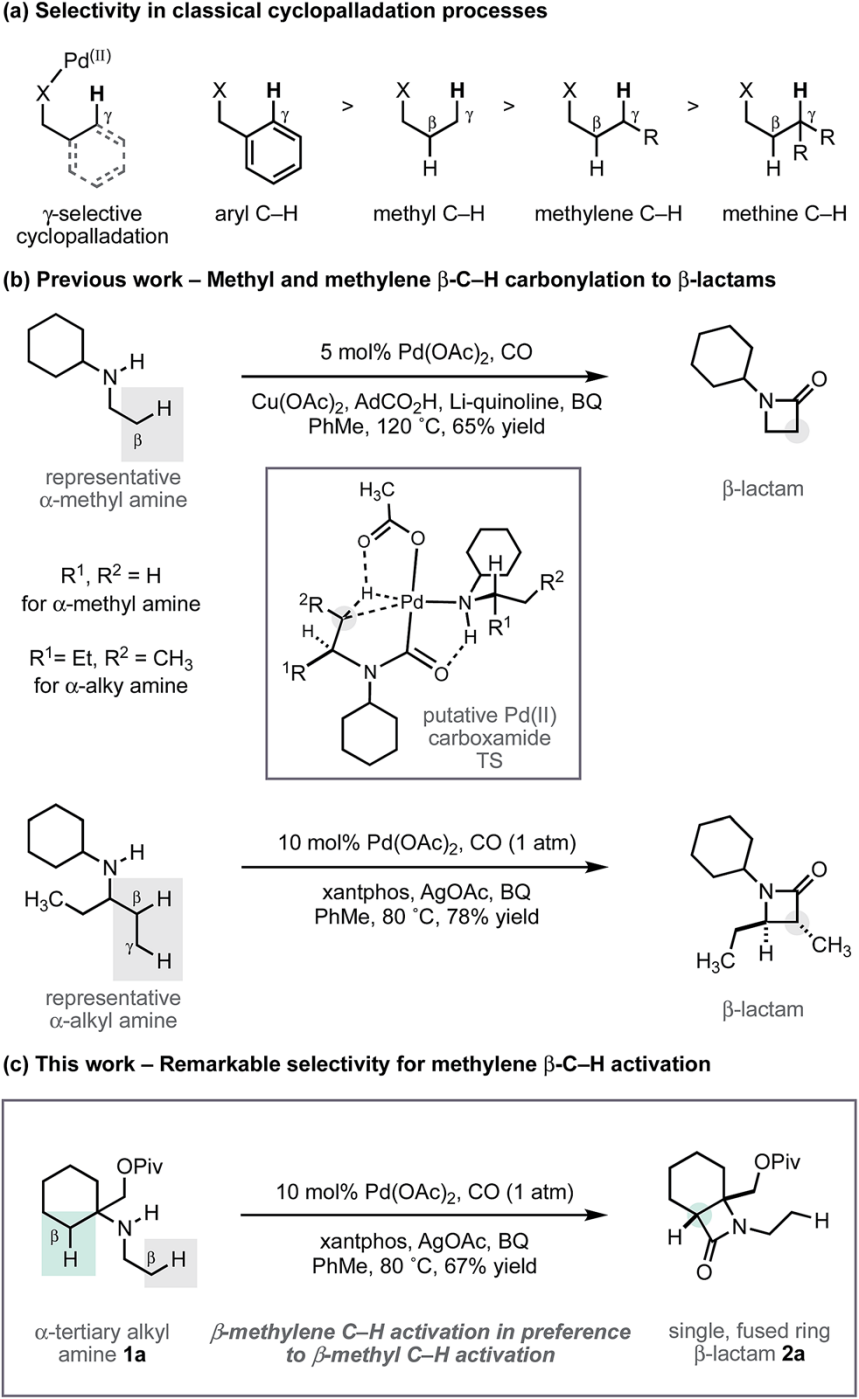
Overview of C–H carbonylation of aliphatic amines.

**Scheme 2:**
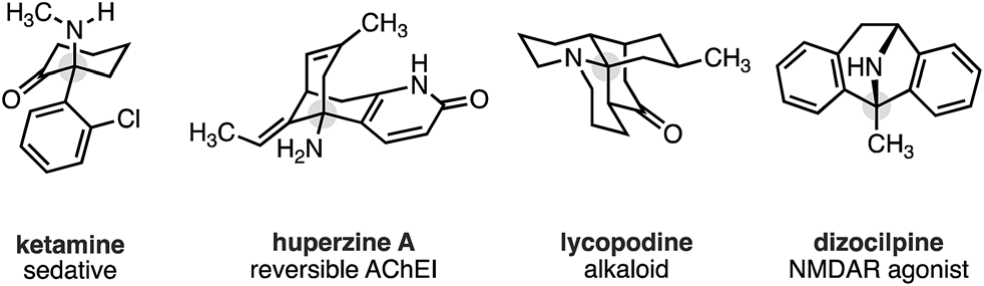
Pharmaceuticals and alkaloids containing the ATA motif.

Using the conditions developed for methylene C–H carbonylation,^9^ using xantphos as a ligand,^12^ we first assessed substrates displaying a variety of substituents in the α-position on the reacting side of the amine linkage; the secondary amines also contained a β-methyl C–H bond (in the form of an *N*-ethyl group) on the other side of the free (NH) motif (Table 1). Substrates containing protected α-hydroxymethylene substituents proved effective under the reaction conditions, delivering the fused bicyclic β-lactams (**2a** and **2b**) resulting from selective methylene C–H carbonylation in good yields.^13^ Moreover, an α-*n*-butyl chain was also sufficient to deliver the corresponding bicyclic β-lactam **2c** in 59% yield, remarkably without any activation of the exocyclic α-alkyl substituent, which contains a competitive methylene β-C–H bond. Exclusive methylene C–H activation also occurred on the corresponding acyclic substrate **1d**, further expanding the utility of the methodology. The corresponding *N*-isopropyl substrate **1e**, for which there is a 6 : 4 ratio of methyl to methylene C–H bonds, afforded a 1.5 : 1 mixture of β-lactams in favour of the methylene C–H activated product, exemplifying the remarkable selectivity inherent to this C–H activation process.

**Table 1:** *N*-Ethyl substituted ATA substrate scope for selective methylene C–H carbonylation

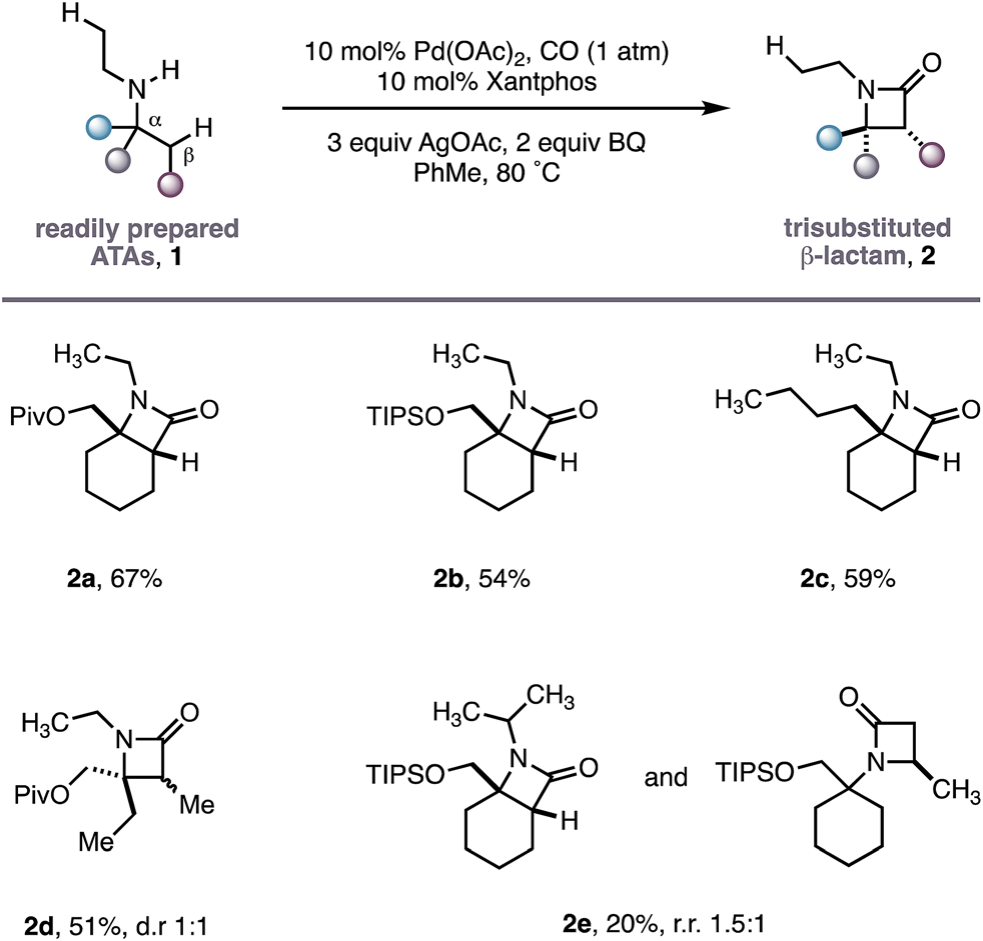

We hypothesize that the selectivity of this methylene C–H carbonylation process arises from the unique Pd(ii)–carboxamide intermediate (pathways A and B, Scheme 3). Based on our previous work,^7^ we propose that a key hydrogen-bond between the carboxamide carbonyl and ligated amine locks the relative conformation of these two substituents, in turn generating two potentially reactive carboxamide intermediates (int-**I** and int-**II**). We believe that the large α-tertiary amine substituent generates an unfavorable steric clash with the ligated amine (int-**II**), resulting in preferential activation of the highlighted methylene C–H bond (pathway A).^14^ While this model holds for the majority of the substrates, we believe that the large isopropyl amine substituent in amine **1e** may result in poorer steric differentiation between the two Pd–carboxamide transition states, leading to the formation of both methylene and methyl activated products (Table 1).

**Scheme 3:**
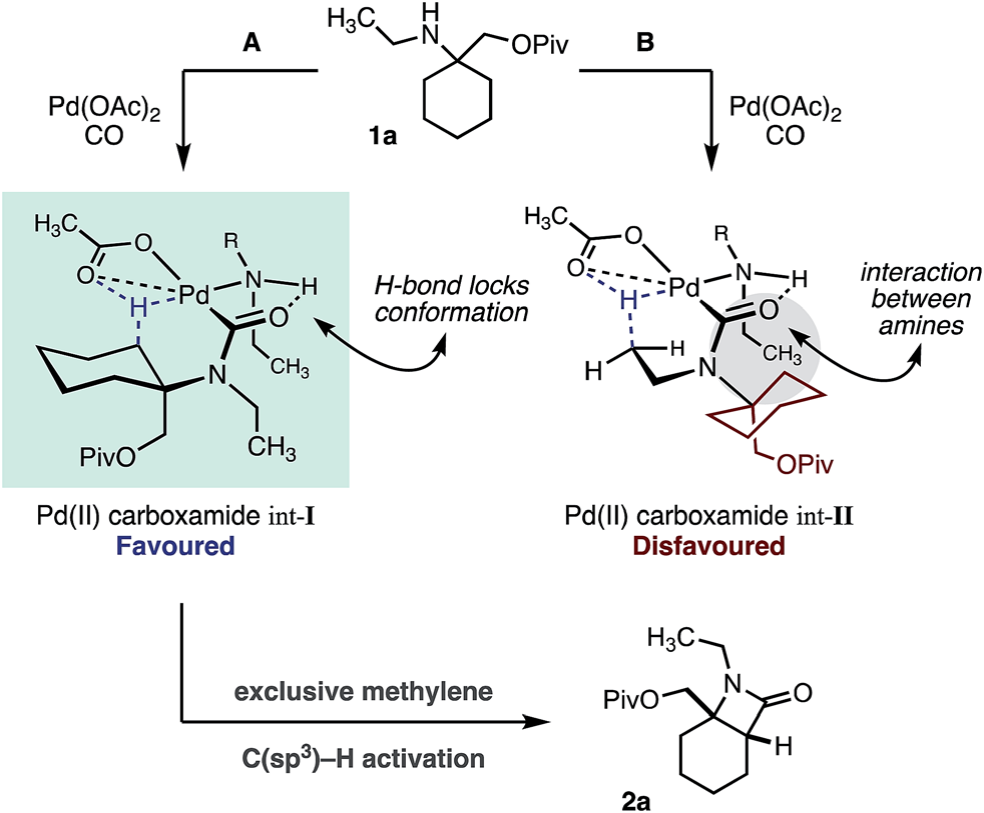
Mechanistic hypothesis for C–H activation of ATAs.

Having successfully demonstrated that a fully substituted centre in the α-position to the amine is sufficient to induce exclusive β-methylene C–H activation, we next explored how substituents on the non-reacting side of the amine affected the carbonylation process (Table 2). The competing classical 5-membered cyclopalladation was not observed in *n*-propyl-containing amine **2g** or *n*-heptyl amine **2f**, affording the corresponding bicyclic β-lactams in a 66% and 70% yield respectively. β-Amino ester **2h** and sulfone **2i** derivatives bearing acidic α-hydrogens, which have previously been shown to promote C–H activation,^9^ were tolerated in good yield and on gram scale.

**Table 2:** ATA directed methylene C–H carbonylation

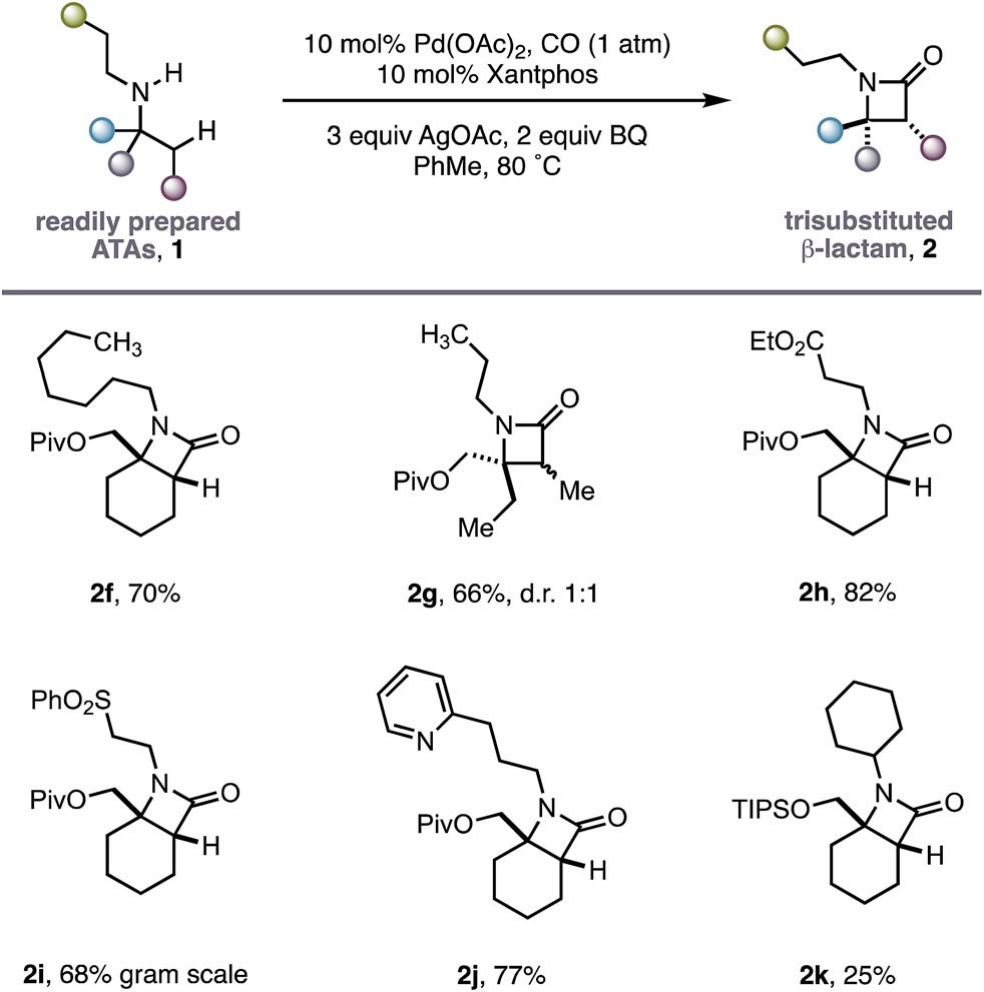

The use of Lewis basic heteroaromatics, such as pyridyl motifs, to direct C(sp^3^)–H activation is well established.5c,^15^ An amine displaying 2-pyridyl substituent **1j** was tolerated in good yield, with no competitive C–H activation on the propyl chain. Impressively, bis-cyclohexyl substrate **1k**, bearing two very similar sets of methylene C–H bonds, afforded a single β-lactam **2k** with activation occurring exclusively in the α-position to the quaternary carbon centre.

Having established the robustness of this methodology towards a range of functional groups, we turned our attention to substrates containing *N*-methyl amines (Table 3). Despite the ubiquity of *N*-Me amines in biologically active molecules and pharmaceutical agents, their deleterious reactivity with many electrophilic transition metal catalysts has rendered them challenging substrates for C–H activation.^16^ The facile oxidation of *N*-methylamines to the corresponding imine followed by nucleophilic capture has been exploited in numerous transformations.^17^ Due to the high pharmaceutical utility of *N*-methylamines,^18^ we sought to test the limits of our C–H activation methodology by investigating this important class of amine substrate. By virtue of our geometrically locked Pd–carboxamide intermediate, we reasoned that the *N*-methyl group would be placed in a remote position relative to the reactive palladium centre, thereby enabling a selective process.

**Table 3:** *N*-Methyl substituted ATAs as substrates for selective methylene C–H carbonylation

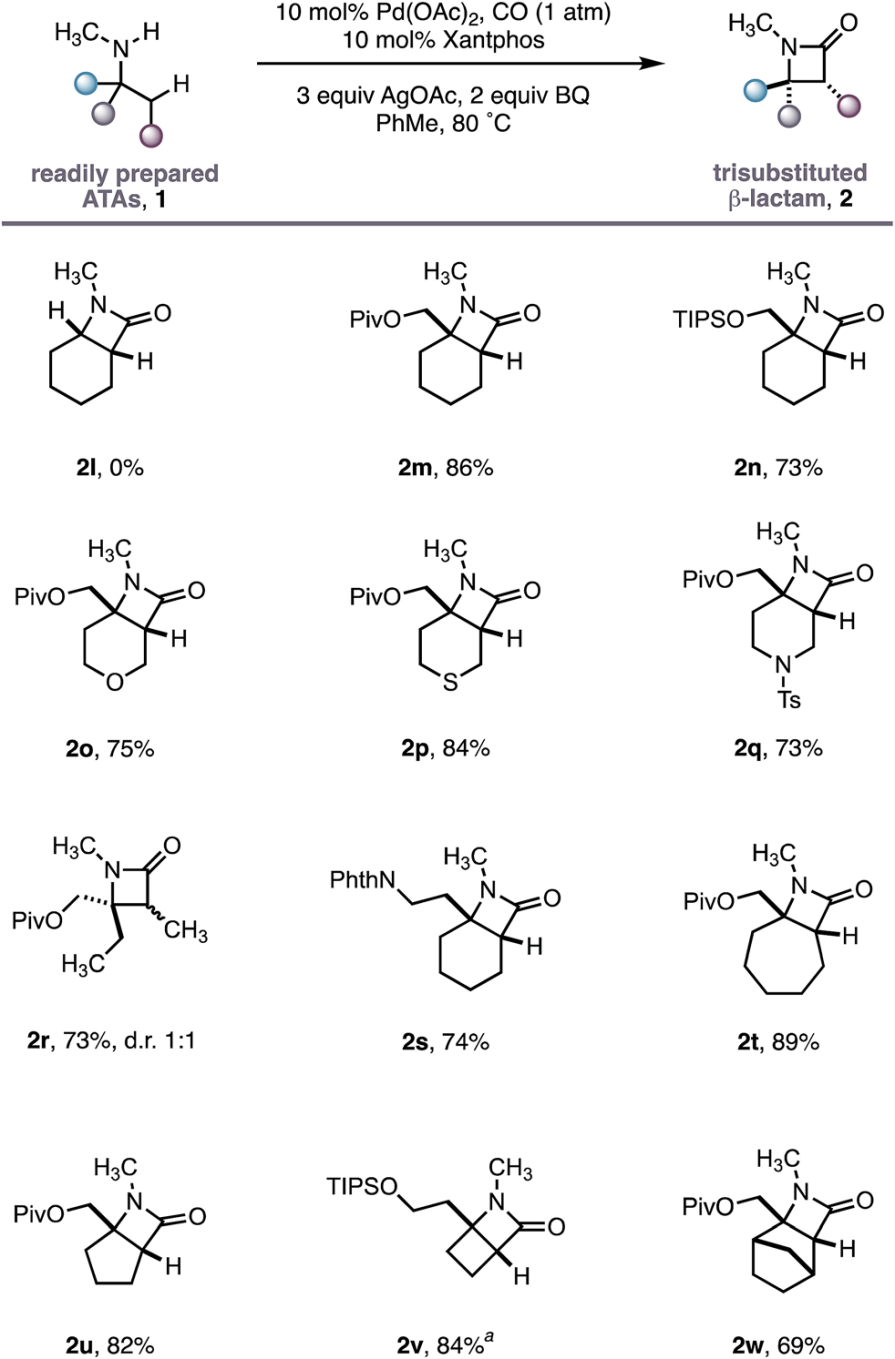

^*a*^Reaction with 10 mol% Pd(OPiv)_2_ and 3 equiv. AgOPiv.

As a control experiment, *N*-methylcyclohexylamine **1l**, lacking the important fully substituted α-tertiary centre, was subjected to our optimized conditions; none of the desired β-lactam product was observed and the starting amine decomposed. In line with our hypothesis, α-tertiary amino-alcohol derivatives **1m** and **1n** delivered the corresponding β-lactams (**2m–n**) in good yield without any demethylation. Piperidine and tetrahydropyran motifs are common among pharmaceutical agents but their functionalization at C3 and C4 positions can present a significant challenge;^19^ our methodology delivered the bicyclic β-lactam products **2o** and **2q** in good yields, allowing for further derivatization of the C3 position. The reaction also proved to be tolerant of a thioether moiety, known to deactivate transition metal catalysts, delivering the β lactam **2p** in a good 84% yield. Moreover, the reaction proved extremely versatile across a range of ring sizes (**2t** to **2w**) in good yield. Pleasingly, cyclobutylamine **1v** was readily transformed into highly strained 4,4-fused β-lactam **2v**, permitting access to functionalized hydrogenated variants of the ‘Dewar-pyridone’ scaffold.^20^

To test the limits of the positional selectivity of the ATA carbonylation among many potentially reactive C–H bonds, we prepared a range of functional amines that could lead to a number of different lactam products (Scheme 4). Indole rings are considered a “privileged” scaffold in medicinal chemistry,^21^ however, they often undergo facile C(sp^2^)–H activation.^22^ We were pleased to observe that tryptamine analogue **1x** bearing a cyclobutane ring was readily transformed into the 4,4-fused β-lactam **2x** in good yield without any competing C(sp^2^)–H activation. 3-Methylamino piperidine **1y**, containing two different ring C–H bond environments, afforded complete selectivity for the C4 position in useful yield (**2y**). Similarly, the 2-aminotetralin substrate **1z** proved to activate selectively at the benzylic position in good yield, revealing a class of tricyclic β-lactam scaffolds (**2z**).

**Scheme 4:**
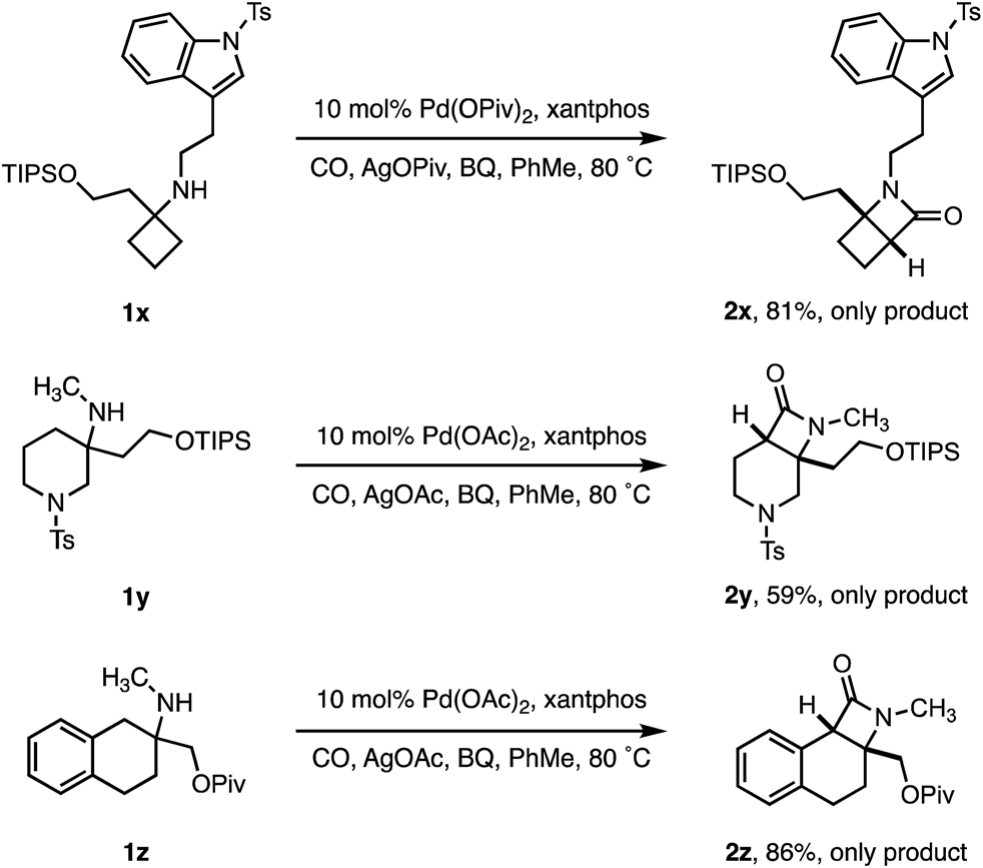
Selectivity of ATA methylene C–H carbonylation.

To challenge the capacity of the selective C–H carbonylation process, we next designed a substrate that would place a β-methylene C–H bond in competition with a C(sp^2^)–H bond on the ortho position of a benzylamine motif. The cyclopalladation of benzylamines is, arguably, one of the most facile and well understood C–H activation processes, with near exclusive C(sp^2^)–H activation control.^23^ Orito and coworkers have shown that alkyl-benzyl substituted secondary amines undergo selective C(sp^2^)–H carbonylation to benzolactams, with no trace of reaction at the C(sp^3^)–H bond (Scheme 5a).^24^ To benchmark the reactivity of our alkyl-benzyl amines, we applied Orito's conditions to *N*-benzyl amine derivative **1aa** and found that benzolactam **3aa** resulting from C(sp^2^)–H activation was produced as the sole product (Scheme 5a).

**Scheme 5:**
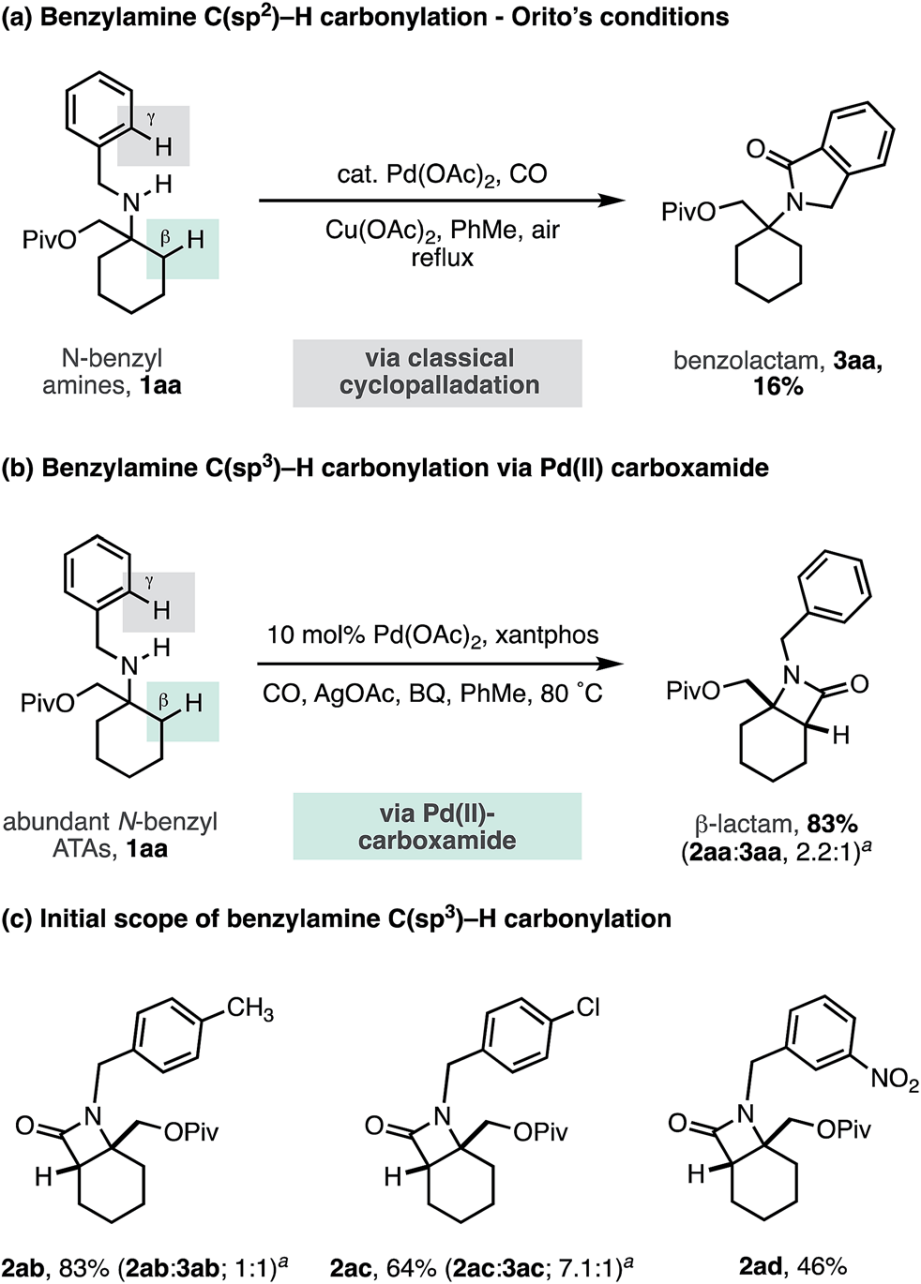
*N*-Benzyl ATA substrate scope for selective methylene C–H carbonylation. ^*a*^Ratio of β-lactam **2** to γ-benzolactam **3**.

Upon switching to our optimized C–H carbonylation conditions, we were delighted to see that a mixture of β-lactam **2aa** and benzolactam **3aa** was formed in a good 83% yield with a 2.2 : 1 ratio in favor of the C(sp^3^)–H activation product **2aa** (Scheme 5b). Encouragingly, we found that changing the electronic properties of the aromatic ring had a significant impact on the product distribution (Scheme 5c). Electron withdrawing substituents favored C(sp^3^)–H activation, with *m*-NO_2_Ph affording exclusively the β-lactam product **2ad**, with no C–H activation observed on the aromatic ring. These results suggest that classical C(sp^2^)–H activation to the benzolactam occurs *via* an electrophilic cyclopalladation pathway. To the best of our knowledge, this is the first example of a palladium catalyzed C–H activation that is selective for a β-methylene C–H bond in the presence of a γ-C(sp^2^)–H bond on an aromatic ring.

Finally, we transformed the β-lactam products into a range of useful chemical building blocks (Scheme 6). Alkylation to form β-lactams displaying vicinal fully substituted stereocenters proceeded in good yield (**4a**). Reduction of **2n** to the corresponding azetidinyl alcohol **4b**, a useful class of scaffold in the design of pharmaceutical agents, occurred in an excellent 90% yield. Importantly, the free (NH)lactam **4c** could be obtained in good yield under mild conditions from the corresponding sulfonyl β-lactam **2h**, offering a simple lactam deprotection protocol.

**Scheme 6:**
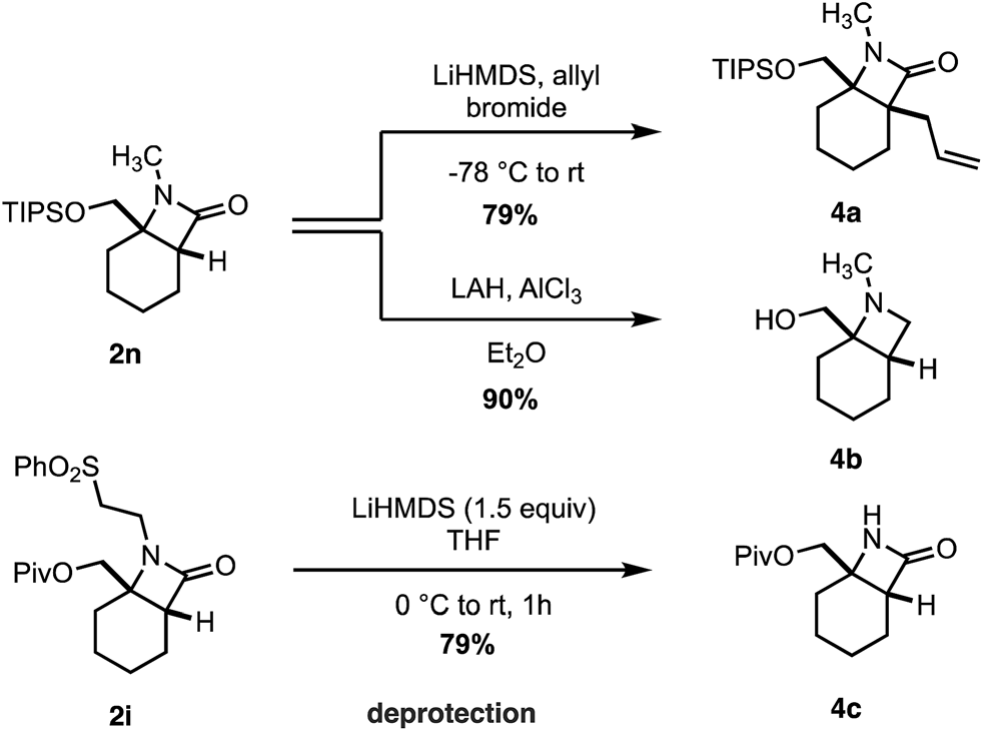
Transformation of β-lactam products.

In conclusion, we have developed a remarkable aliphatic amine C–H carbonylation reaction that is capable of selectively activating β-methylene C–H bonds in the presence of traditionally more reactive C(sp^3^) and C(sp^2^)–H bonds. The presence of a fully substituted carbon atom in the α-position to the amine appears to control this unprecedented selectivity. Using this methodology, a range of highly functionalized β-lactam building blocks have been synthesized in good yields, which can further be derivatised in order to access novel heterocyclic scaffolds that we believe we be useful to a range of synthetic and medicinal applications. Computational studies to explore the origin of this unique selectivity in further detail are currently ongoing within our group.

## Conflicts of interest

There are no conflicts to declare.

## Supplementary Material

Supplementary information

Crystal structure data
