# Supplementary material for: The α-tertiary amine motif drives remarkable selectivity for Pd-catalyzed carbonylation of β-methylene C–H bonds
Source: Chem Sci. 2017 Oct 9;8(12):8198–203. doi: 10.1039/c7sc03876c (PMC5855970; doi:10.1039/c7sc03876c)
Supplement: Supplementary file 1 [file SC-008-C7SC03876C-s001.pdf]

Electronic Supplementary Material (ESI) for Chemical Science.
This journal is © The Royal Society of Chemistry 2017

Supplementary Materials

Table of Contents

**General Experimental**	**S2**
**Synthesis of Starting Amines**	**S3**
**Synthesis of β–Lactam Products**	**S40**
**Functionalization of β–Lactam Products**	**S60**
**NMR Spectra of Starting Amines**	**S62**
**NMR Spectra of of β–Lactams**	**S93**
**NMR Spectra of of Functionalized Products**	**S127**

General Experimental

All reactions were run under an inert atmosphere (N_2 or Ar where specified) unless otherwise stated, with oven-dried glassware, using standard techniques. Anhydrous solvents were obtained from solvent stills (Et_2O was distilled from sodium triphenylmethane ketyl; THF from $LiAlH_4$; MeCN, CH_2Cl_2, hexane and toluene from CaH_2). All other commercial reagents were used as supplied unless otherwise stated. 1,4-benzoquinone was recrystallised from hexane prior to use and stored in the dark. Silver salts were obtained from Alfa Aesar and used as supplied. Palladium salts were obtained from Alfa Aesar, and used as supplied. Silver pivalate was prepared according to a literature procedure.[1]

Analytical thin-layer chromatography (TLC) was performed on Merck Kiesegel 60 F254 0.20 mm precoated, glass backed silica gel plates. Visualisation of the developed chromatogram was performed by UV absorbance (λ_{max} = 254 nm), and/or by aqueous $KMnO_4$. Flash column chromatography was performed using silica gel (Merck Geduran Si 60 [40-63 μm]) with the indicated solvent system. P.E. refers to 40-60 petroleum ether.

Nuclear magnetic resonance (NMR) spectra were recorded on a Bruker DPX 400 or DPX 500 spectrometer with cryoprobe. Chemical shifts (δ) for ^{1}H NMR spectra are recorded in ppm from Me_4Si with the solvent resonance as the internal standard ($CDCl_3$ = 7.26 ppm, D_2O = 4.79 ppm, $(CD_3)_2SO$ = 2.50 ppm, CD_3OD = 3.31 ppm). Data is reported as follows: chemical shift [integration, multiplicity (s = singlet, d = doublet, t = triplet, q = quartet, qt = quintet, sxt = sextet, spt = septet, m = multiplet, br = broad), coupling constant and molecular assignment]. ^{13}C NMR spectra are reported in ppm from Me_4Si with the solvent resonance as the internal standard ($CDCl_3$ = 77.16 ppm, $(CD_3)_2SO$ = 39.52, CD_3OD = 49.00).

Infrared spectra (FT-IR) were recorded using a Perkin-Elmer Paragon 1000 Fourier transform Spectrometer equipped with ATR and analysed as thin films, with absorption maxima (ν_{max}) being quoted in wavenumbers (cm^{-1}) and characteristic peaks being defined (s = strong, br = broad). High Resolution Mass spectrometry (HRMS) was carried out by the ESPRC Mass Spectrometry Service at the University of Swansea using an LTQ Orbitrap XL spectrometer with positive ion nano-electrospray. Melting points (m.p.) were recorded using a Gallenkamp melting point apparatus and are reported uncorrected.

[1]Endo, K.; Grubbs, R. H. *J. Am. Chem. Soc.* **2011**, *133*, 8525.

Procedures for the Synthesis of Starting Materials

(1-(ethylamino)cyclohexyl)methyl pivalate (1a)

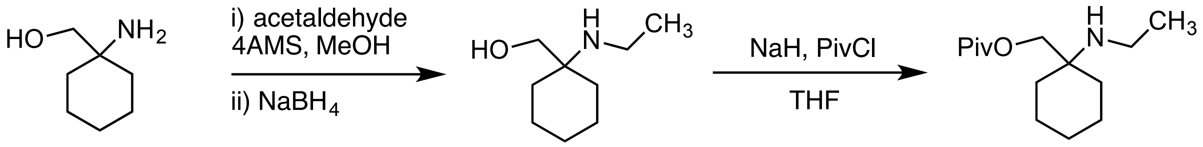

Step 1

To a solution of (1-aminocyclohexyl)methanol[2] (1.08 g, 8.4 mmol) in MeOH (10 mL) was added powdered 4Å molecular sieves (~1 g) and acetaldehyde (0.71 mL, 12.5 mmol) and the mixture stirred at ambient temperature for 16 h. The mixture was cooled to 0 °C, sodium borohydride (0.473 g, 12.5 mmol) was added portion-wise and mixture allowed to warm to room temperature and stirred for an additional 2 h. The reaction mixture was filtered and the MeOH removed *in vacuo*. 10% Aqueous NaOH solution was added and the mixture extracted with EtOAc (3 x 10 mL). The combined organic phases were dried ($MgSO_4$) and concentrated *in vacuo* to afford the crude product as a white solid (1.12 g, 85%) which was used in the next step without further purification. ^{1}H NMR (400 MHz, $CDCl_3$) δ: 3.29 (2 H, s), 2.50 (2 H, q, J = 7.1 Hz), 1.58 – 1.29 (10 H, m), 1.09 (3 H, t, J = 7.1 Hz).

Step 2

(1-(ethylamino)cyclohexyl)methanol (1.12 g, 7.12 mmol) was dissolved in THF (15 mL) and cooled to 0 °C. Sodium hydride (60% in mineral oil, 0.29 g, 7.12 mmol) was added portion-wise and the reaction mixture stirred for 30 minutes at 0 °C. Trimethylacetyl chloride (1.05 mL, 8.55 mmol) was added drop-wise and the reaction mixture allowed to stir for 2 h at room temperature. The solution was concentrated *in vacuo* and water (15 mL) was added. The aqueous layer was extracted with CH_2Cl_2 (2 x 20 mL), the combined organic layers washed with saturated aqueous $NaHCO_3$ (15 mL) then water (15 mL), dried over $MgSO_4$ and concentrated *in vacuo*. The residue was purified by flash column chromatography (alumina, Gradient elution: P.E. to 10% EtOAc in P.E.) to afford the title compound as a colourless oil (0.972 g, 57%). IR ν_{max}/cm^{-1} (film): 2929, 2857, 1728 (C=O), 1481, 1453, 1395, 1366, 1282, 115; ^{1}H NMR (500 MHz, $CDCl_3$) δ: 3.95 (2 H, s), 2.50 (2 H, q, J = 7.1 Hz), 1.64 – 1.54 (2 H, m), 1.54 – 1.44 (3 H, m), 1.43 – 1.32 (5 H, m), 1.21 (9 H, s), 1.06 (3 H, t, J = 7.1 Hz); ^{13}C NMR (126 MHz, $CDCl_3$) δ: 178.5, 67.5, 53.9, 39.1, 35.4, 33.0, 27.4, 26.2, 21.5, 16.3; m/z HRMS found $[M + H]^+$ 242.2108, $C_{14}H_{28}NO_2$ requires 242.2115.

[2] Meinzer, A.; Breckel, A.; Thaher, B. A.; Manicone, N.; Otto, H-H. *Helv. Chim. Acta*, **2004**, 90.

***N*-ethyl-1-(((triisopropylsilyl)oxy)methyl)cyclohexan-1-amine (1b)**

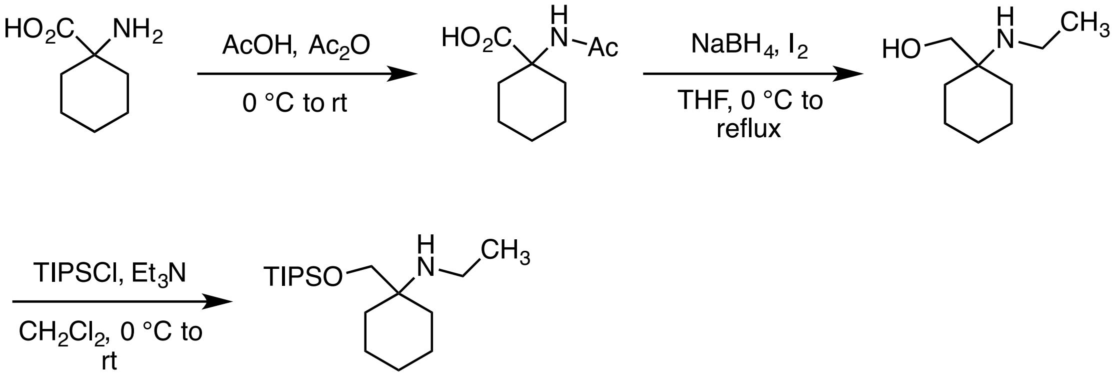

Step 1

Acetic anhydride (2.0 mL) was added dropwise to a solution of 1-aminocyclohexane-1-carboxylic acid (1.36 g, 9.5 mmol) in acetic acid (5 mL) at room temperature. After the addition was complete the reaction mixture was stirred for an additional 16 hours and quenched by the addition of ice water (10 mL). The solvent was removed *in vacuo* to give the crude *N*-acetyl amino acid which was used directly in the next step without further purification (1.55 g, 88%). ^{1}H NMR (400 MHz, $(CD_3)_2SO$) δ: 7.78 (1 H, s), 1.82 (3 H, s), 1.67 – 1.55 (4 H, m), 1.54 – 1.37 (6 H, m).

Step 2

To a vigorously stirred suspension of *N*-acetyl amino acid (1.55 g, 8.4 mmol) and sodium borohydride (0.89 g, 23.4 mmol) in anhydrous THF (25 mL) was added a solution of iodine (2.55 g, 10.0 mmol) in anhydrous THF (12 mL) dropwise at 0 °C. Once gas evolution had ceased the mixture was heated at reflux for 18 hours and subsequently cooled to room temperature. Methanol was added cautiously until the solution became clear and the solvent was subsequently removed *in vacuo* to afford a white paste. The paste was dissolved in the minimum amount of 20% aqueous KOH (ca. 7 mL) and stirred at room temperature for 1 hour. The mixture was diluted with 20% aqueous KOH (20 mL) and extracted with CH_2Cl_2 (3 x 30 mL). The organics were combined, dried over $MgSO_4$ and removed *in vacuo* to give the crude amino alcohol as a colorless oil (0.98 g, 75%) which was used in the next step without further purification ^{1}H NMR (400 MHz, $CDCl_3$) δ: 3.31 (2 H, s), 2.49 (2 H, q, J = 7.1 Hz), 1.57 – 1.35 (10 H, m), 1.11 (3 H, t, J = 7.1 Hz).

Step 3

Triisopropylsilyl chloride (0.74 mL, 3.5 mmol) was added dropwise to a solution of amino alcohol (0.50 g, 3.2 mmol) and triethylamine (0.88 mL, 6.3 mmol) in anhydrous CH_2Cl_2 (32 mL) at 0 ºC. The resulting solution was allowed to warm to room temperature over 16 hours and was quenched with

H_2O (30 mL). The organics were separated and washed with additional H_2O (2 x 30 mL) and brine (30 mL), dried over Na_2SO_4 and removed *in vacuo*. The crude oil was purified by flash column chromatography (EtOAc) to provide the desired amine as pale yellow oil (0.32g, 32%). IR ν_{max}/cm^{-1} (film): 2929, 2865, 1463, 1383, 1368, 1306, 1248, 1111, 1093, 1069, 1058. ^1H NMR (400 MHz, $CDCl_3$) δ: 3.51 (2 H, s), 2.48 (2 H, q, *J* = 7.1 Hz), 1.60 – 1.53 (2 H, m), 1.46 – 1.30 (9 H, m), 1.07 – 1.02 (24 H, m). ^{13}C NMR (101 MHz, $CDCl_3$) δ: 66.0, 55.2, 35.2, 32.6, 26.4, 22.0, 18.2, 16.2, 12.1. m/z HRMS found $[M + H]^+$ 314.2871, $C_{18}H_{40}ONSi$ requires 314.2874.

1-butyl-*N*-ethylcyclohexan-1-amine (1c)

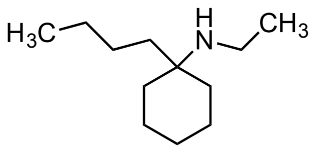

Cyclohexanone (2.07 mL, 20 mmol) and ethylamine (2M solution in THF, 10 mL, 20 mmol) were dissolved in THF (10 mL) over powdered molecular sieves (2 g) and the solution stirred for 18 h. The mixture was filtered and the solvent removed *in vacuo* to afford a light yellow liquid which was dissolved in hexane (4 mL) and added *via* syringe pump over 15 minutes to a stirred solution of *n*-butyl lithium (2.5 M solution in hexane, 16 mL, 40 mmol) in anhydrous hexane (10 mL) at 0 °C. The reaction mixture was allowed to warm to room temperature and stirred for 2 h after which the solution was poured slowly over crushed ice with swirling. The resulting aqueous solution was extracted with Et_2O (2 x 15 mL), dried over $MgSO_4$ and concentrated *in vacuo*. Purification by Kugelrohr distillation followed by flash column chromatography (gradient elution: 10% EtOAc in P.E. to 20% EtOAc in P.E.) afforded the desired compound as a light yellow oil (0.459 g, 2.5 mmol, 13%). IR ν_{max}/cm^{-1} (film): 2921, 2857, 1453, 1380, 1267, 1161, 1116, 1078; ^1H NMR (400 MHz, $CDCl_3$) δ: 2.46 (2 H, q, *J* = 7.1 Hz), 1.52 – 1.15 (16 H, m), 1.08 (3 H, t, *J* = 7.1 Hz), 0.90 (3 H, t, *J* = 7.2 Hz); ^{13}C NMR (101 MHz, $CDCl_3$) δ: 53.5, 36.4, 35.8, 34.9, 26.4, 25.0, 23.6, 22.1, 16.3, 14.4; m/z HRMS found $[M + H]^+$ 184.2055, $C_{12}H_{26}N$ requires 184.2060.

2-ethyl-2-(ethylamino)butyl pivalate (1d)

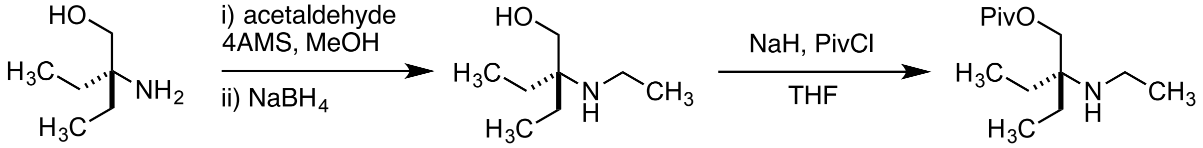

Step 1

To a solution of 2-amino-2-ethylbutan-1-ol[3] (0.586 g, 5.00 mmol) in MeOH (10 mL) was added powdered 4Å molecular sieves (~1 g) and acetaldehyde (0.42 mL, 7.5 mmol) and the mixture stirred at ambient temperature for 16 hours. The mixture was cooled to 0 °C, sodium borohydride (0.28 g, 7.5 mmol) was added portion-wise and mixture allowed to warm to ambient temperature and stirred for an additional 2 h. The reaction mixture was filtered and the MeOH removed *in vacuo*. 10% Aqueous NaOH solution was added and the mixture extracted with EtOAc (3 x 10 mL). The combined organic phases were dried ($MgSO_4$) and concentrated *in vacuo* to afford the crude product as a light yellow oil (0.796 g, 97%) which was used in the next step without further purification. ^1H NMR (400 MHz, $CDCl_3$) δ: 3.23 (2 H, s), 2.46 (2 H, q, J = 7.1 Hz), 1.35 (2 H, dq, J = 15.1, 7.6 Hz), 1.30 – 1.20 (2 H, m), 1.07 (3 H, t, J = 7.1 Hz), 0.79 (6 H, t, J = 7.6 Hz).

Step 2

2-ethyl-2-(ethylamino)butan-1-ol (0.706 g, 4.86 mmol) was dissolved in THF (10 mL) and cooled to 0 °C. Sodium hydride (0.194 g, 4.86 mmol) was added portion-wise and the reaction mixture stirred for 30 minutes at 0 °C. Trimethylacetyl chloride (0.719 mL, 5.83 mmol) was added drop-wise and the reaction mixture allowed to stir for 2 hours at room temperature. The solution was concentrated *in vacuo* and water (10 mL) was added. The aqueous layer was extracted with CH_2Cl_2 (2 x 10 mL), the combined organic layers washed with saturated aqueous $NaHCO_3$ (15 mL) then water (15 mL), dried over $MgSO_4$ and concentrated *in vacuo*. The residue was purified by flash column chromatography (alumina, gradient elution: P.E. to 10% EtOAc in P.E.) to afford the title compound as a colourless oil (0.528 g, 47%). IR ν_{max}/cm^{-1} (film): 2967, 2872, 1730, 1480, 1461, 1396, 1365, 1282, 1149, 1034; ^1H NMR (400 MHz, $CDCl_3$) δ: 3.90 (2 H, s), 2.49 (2 H, q, J = 7.1 Hz), 1.49 – 1.31 (4 H, m), 1.21 (9 H, s), 1.07 (3 H, t, J = 7.1 Hz), 0.83 (6 H, t, J = 7.5 Hz); ^{13}C NMR (101 MHz, $CDCl_3$) δ: 178.4, 65.8, 57.1, 39.1, 35.5, 27.4, 26.0, 16.2, 7.5; m/z HRMS found $[M + H]^+$ 230.2113, $C_{13}H_{28}NO_2$ requires 230.2115.

[3] Calleja, J.; Pla, D.; Gorman, T. W.; Domingo, V.; Haffemayer, B.; Gaunt, M. J. *Nat. Chem.*, **2015**, 1009.

***N*-cyclohexyl-1-(((triisopropylsilyl)oxy)methyl)cyclohexan-1-amine**

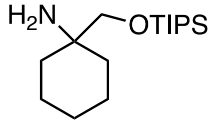

(1-aminocyclohexyl)methanol (6.46 g, 50.0 mmol) was dissolved in CH_2Cl_2 (100 mL). Triethylamine (13.95 mL, 60.0 mmol) was added followed by slow addition of triisopropylsilyl chloride (12.85 mL, 100.0 mmol). The reaction mixture was stirred at room temperature for 16 hours, quenched with water (100 mL) and extracted with CH_2Cl_2 (2 x 100 mL). The combined organic layers were washed with brine (100 mL), dried over $MgSO_4$ and concentrated *in vacuo*. The residue was purified by flash column chromatography (gradient elution: 100% CH_2Cl_2 to 5% MeOH in CH_2Cl_2) to afford the desired compound as a light yellow oil (5.48 g, 38%). IR ν_{max}/cm^{-1} (film): 2930, 2865, 1463, 1383, 1249, 1094, 1068, 1013; ^1H NMR (400 MHz, $CDCl_3$) δ: 3.46 (2 H, s), 1.59 – 1.24 (10 H, m), 1.14 – 0.99 (21 H, m); ^{13}C NMR (101 MHz, $CDCl_3$) δ: 72.4, 52.5, 35.3, 26.4, 22.1, 18.2, 12.1; m/z HRMS found $[M + H]^+$ 286.2554, $C_{16}H_{35}NOSi$ requires 286.2561.

***N*-isopropyl-1-(((triisopropylsilyl)oxy)methyl)cyclohexan-1-amine (1e)**

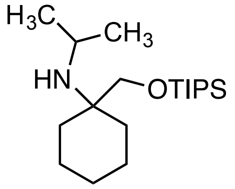

Acetone (1.65 mL, 22.5 mmol) was dissolved in titanium(IV) isopropoxide (6.66 mL, 22.5 mmol) followed by the addition of 1-(((triisopropylsilyl)oxy)methyl)cyclohexan-1-amine (4.28 g, 15.0 mmol) and stirred at ambient temperature overnight before cooling to 0 °C and adding MeOH (30 mL) to the reaction mixture. Sodium borohydride (1.02 g, 27.0 mmol) was added portion-wise and the reaction mixture stirred at 0 °C for 20 minutes then ambient temperature for 1 h. The reaction was quenched by the addition of 10% aqueous NaOH (30 mL) and then diluted with CH_2Cl_2 (30 mL). The mixture was filtered through Celite, eluting with CH_2Cl_2. The organic phase was separated, dried over $MgSO_4$ and concentrated *in vacuo*. The resulting crude product was purified by flash column chromatography (10% EtOAc in P.E) to afford the title compound as a light yellow oil (0.661 g, 14%). IR ν_{max}/cm^{-1} (film): 2928, 2865, 1462, 1378, 1361, 1248, 1171, 1111, 1090, 1062, 1012; ^1H NMR (400 MHz, $CDCl_3$) δ: 3.54 (2 H, s), 2.92 (1 H, hept, J = 6.2 Hz), 1.66 – 1.52 (2 H, m), 1.48 – 1.29 (8 H, m), 1.10 – 1.02 (27 H, m). ^{13}C NMR (101 MHz, $CDCl_3$) δ: 67.0, 56.3, 41.8, 33.3, 26.4, 26.4, 22.3, 18.3, 12.2; m/z HRMS found $[M + H]^+$ 328.3024, $C_{19}H_{42}NOSi$ requires 328.3030.

(1-(heptylamino)cyclohexyl)methyl pivalate (1f)

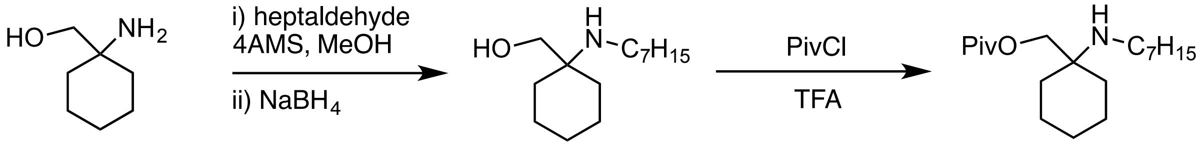

Step 1

To a solution of (1-aminocyclohexyl)methanol[2] (1.00 g, 7.7 mmol) in MeOH (10 mL) was added powdered 4Å molecular sieves (~1 g) and heptaldehyde (1.61 mL, 11.55 mmol) and the mixture stirred at ambient temperature for 16 h. The mixture was cooled to 0 °C, sodium borohydride (437 mg, 11.55 mmol) was added portion-wise and mixture allowed to warm to ambient temperature and stirred for an additional 2 h. The reaction mixture was filtered and the MeOH removed *in vacuo*. 10% Aqueous NaOH solution was added and the mixture extracted with EtOAc (3 x 10 mL). The combined organic phases were dried ($MgSO_4$) and concentrated *in vacuo* to afford the crude product as a white waxy solid (1.24 g, 71%) which was used in the next step without further purification. ^1H NMR (400 MHz, $CDCl_3$) δ: 3.31 (2 H, s), 2.46 (2 H, t, J = 7.0 Hz), 1.59 – 1.43 (8 H, m), 1.43 – 1.35 (4 H, m), 1.35 – 1.20 (8 H, m), 0.88 (3 H, t, J = 6.8 Hz).

Step 2

(1-(heptylamino)cyclohexyl)methanol (1.24 g, 5.45 mmol) was dissolved in trifluoroacetic acid (2.8 mL) and the solution cooled to 0 °C. Trimethylacetyl chloride (1 mL, 8.2 mmol) was added and the solution allowed to warm to room temperature overnight. The volatiles were removed *in vacuo* and the residue dissolved in CH_2Cl_2. Water (5 mL) and triethylamine (5 mL) were added until the solution was basic and the mixture stirred for 5 minutes. The resulting solution was extracted with CH_2Cl_2 (3 x 10 mL) and the combined organic layers washed with water, dried over $MgSO_4$ and concentrated *in vacuo*. Purification by flash column chromatography (15% EtOAc in P.E.) afforded the title compound as a light yellow oil (953 mg, 56%). IR ν_{max}/cm^{-1} (film): 2929, 2853, 1728, 1477, 1459, 1395, 1362, 1280, 1150, 1031; ^1H NMR (400 MHz, $CDCl_3$) δ: 3.95 (2 H, s), 2.45 (2 H, t, J = 7.0 Hz), 1.65 – 1.54 (2 H, m), 1.54 – 1.44 (3 H, m), 1.44 – 1.34 (7 H, m), 1.33 – 1.23 (8 H, m), 1.21 (9 H, s), 0.87 (3 H, t, J = 6.8 Hz); ^{13}C NMR (101 MHz, $CDCl_3$) δ: 178.5, 67.6, 53.8, 41.2, 39.1, 32.9, 32.0, 31.2, 29.4, 27.6, 27.4, 26.2, 22.8, 21.5, 14.2; m/z HRMS found $[M + H]^+$ 312.2893, $C_{19}H_{38}NO_2$ requires 312.2897.

2-ethyl-2-(propylamino)butyl pivalate (1g)

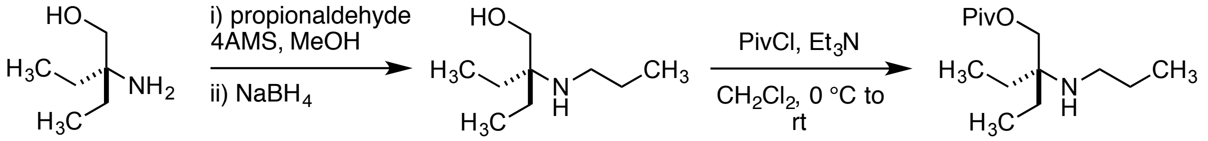

Step 1

To a solution of 2-amino-2-ethylbutan-1-ol (0.50 g, 4.27 mmol) in MeOH (10 mL) was added powdered 4Å molecular sieves (~1 g) and propionaldehyde (0.46 mL, 6.4 mmol) and the mixture stirred at ambient temperature for 16 hours. The mixture was cooled to 0 °C, sodium borohydride (0.24 g, 6.4 mmol) was added portion-wise and the mixture allowed to warm to ambient temperature and stirred for an additional 2 h. The reaction mixture was filtered and the MeOH removed *in vacuo*. 10% aqueous NaOH solution was added and the mixture extracted with EtOAc (3 x 10 mL). The combined organic phases were dried ($MgSO_4$) and concentrated *in vacuo* to afford the crude product as a colourless oil (0.694 g, quant.) which was used in the next step without further purification. ^{1}H NMR (400 MHz, $CDCl_3$) δ: 3.24 (2 H, s), 2.39 (2 H, t, J = 7.0 Hz), 1.51 – 1.40 (2 H, m), 1.40 – 1.30 (2 H, m), 1.29 – 1.22 (2 H, m), 0.93 (3 H, t, J = 7.3 Hz), 0.80 (6 H, t, J = 7.5 Hz).

Step 2

2-ethyl-2-(propylamino)butan-1-ol (0.694 g, 4.27 mmol) was dissolved in CH_2Cl_2 (10 mL) and cooled to 0 °C. Triethylamine (1.19 mL, 8.54 mmol) was added followed by drop-wise addition of trimethylacetyl chloride (0.63 mL, 5.12 mmol). The reaction mixture was allowed to warm slowly to ambient temperature and stirred for 16 hours after which it was quenched with water (10 mL) and extracted with CH_2Cl_2 (x2). The combined organic phases were washed with brine, dried ($MgSO_4$) and concentrated *in vacuo*. The resulting residue was purified by flash column chromatography (alumina, gradient elution, P.E. to 5% EtOAc in P.E.) to afford the tile compound as a colourless oil (0.718 g, 69%). IR ν_{max}/cm^{-1} (film): 2964, 2873, 1730, 1481, 1459, 1397, 1366, 1282, 1152, 1035; ^{1}H NMR (400 MHz, $CDCl_3$) δ: 3.89 (2 H, s), 2.40 (2 H, t, J = 7.1 Hz), 1.50 – 1.31 (6 H, m), 1.20 (9 H, s), 0.91 (3 H, t, J = 7.4 Hz), 0.82 (6 H, t, J = 7.5 Hz); ^{13}C NMR (101 MHz, $CDCl_3$) δ: 178.4, 65.8, 56.8, 43.2, 39.1, 27.4, 26.0, 24.0, 12.1, 7.4; m/z HRMS found $[M + H]^+$ 244.2269, $C_{14}H_{30}NO_2$ requires 244.2271.

(1-((3-ethoxy-3-oxopropyl)amino)cyclohexyl)methyl pivalate (1h)

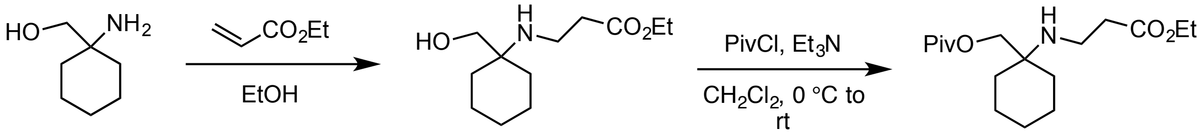

Step 1

(1-aminocyclohexyl)methanol[2] (0.646 g, 5.0 mmol) was dissolved in EtOH (5 mL) and the solution cooled to 0 °C. Ethyl acrylate (0.36 mL, 3.33 mmol) was added drop-wise and the mixture stirred at room temperature for 24 h after which it was concentrated *in vacuo*. Purification of the resulting residue by flash column chromatography (5% MeOH in CH_2Cl_2) afforded the desired compound as a light yellow oil (0.614 g, 80%). IR ν_{max}/cm^{-1} (film): 3429 (br), 2933, 2853, 1728, 1447, 1374, 1261, 1181, 1165, 1098, 1027; ^{1}H NMR (400 MHz, $CDCl_3$) δ: 4.16 (2 H, q, *J* = 7.1 Hz), 3.31 (2 H, s), 2.73 (2 H, t, *J* = 5.8 Hz), 2.48 (2 H, t, *J* = 5.7 Hz), 1.60 – 1.30 (10 H, m), 1.27 (3 H, t, *J* = 7.1 Hz); ^{13}C NMR (101 MHz, $CDCl_3$) δ: 173.3, 65.4, 60.8, 36.0, 35.4, 32.8, 26.2, 21.8, 14.4; m/z HRMS found $[M + H]^+$ 230.1750, $C_{12}H_{24}NO_3$ requires 230.1751.

Step 2

Ethyl 3-((1-(hydroxymethyl)cyclohexyl)amino)propanoate (0.60 g, 2.60 mmol) was dissolved in CH_2Cl_2 (5 mL) and cooled to 0 °C. Triethylamine (0.73 mL, 3.12 mmol) was added followed by drop-wise addition of trimethylacetyl chloride (0.38 mL, 5.20 mmol). The reaction mixture was allowed to warm slowly to ambient temperature and stirred for 16 h after which it was quenched with water (5 mL) and extracted with CH_2Cl_2 (2 x 10 mL). The combined organic phases were washed with brine, dried ($MgSO_4$) and concentrated *in vacuo*. The resulting residue was purified by flash column chromatography (20% EtOAc in P.E.) to afford the desired compound as a light yellow oil (0.673 g, 83%). IR ν_{max}/cm^{-1} (film): 2933, 2857, 1726, 1483, 1459, 1395, 1368, 1282, 1215, 1152, 1102, 1056, 1031; ^{1}H NMR (400 MHz, $CDCl_3$) δ: 4.13 (2 H, q, *J* = 7.1 Hz), 3.93 (2 H, s), 2.76 (2 H, t, *J* = 6.4 Hz), 2.44 (2 H, t, *J* = 6.3 Hz), 1.62 – 1.31 (10 H, m), 1.26 (3 H, t, *J* = 7.1 Hz), 1.22 (9 H, s); ^{13}C NMR (101 MHz, $CDCl_3$) δ: 178.5, 173.0, 68.3, 60.5, 54.0, 39.1, 37.0, 36.2, 32.7, 27.4, 26.2, 21.4, 14.4; m/z HRMS found $[M + H]^+$ 314.2332, $C_{17}H_{32}NO_4$ requires 314.2326.

(1-((2-(phenylsulfonyl)ethyl)amino)cyclohexyl)methyl pivalate (1i)

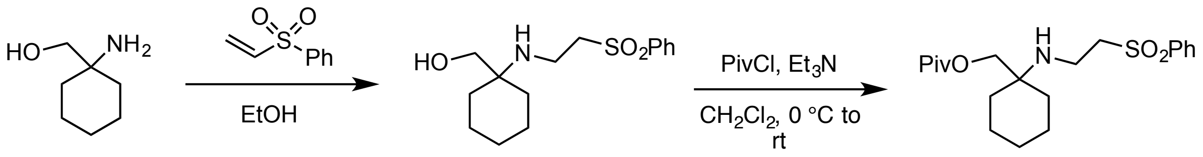

Step 1

(1-aminocyclohexyl)methanol[2] (1.00 g, 7.74 mmol) was dissolved in EtOH (5 mL) and the solution cooled to 0 °C. Phenyl vinyl sulfone (0.868 g, 5.16 mmol) in EtOH (10 mL) was added drop-wise and the mixture stirred at room temperature for 24 hours after which it was concentrated *in vacuo*. Purification of the resulting residue by flash column chromatography (5% MeOH in CH_2Cl_2) afforded the desired compound as a light yellow solid (1.38 g, 60%). m.p: 60–62 °C; IR ν_{max}/cm^{-1} (film): 3111 (br), 2917, 2845, 1479, 1447, 1415, 1312, 1290, 1259, 1221, 1179, 1152, 1088, 1070, 1052, 1036, 1015; ^1H NMR (400 MHz, $CDCl_3$) δ: 7.94 (2 H, m), 7.71 – 7.63 (1 H, m), 7.58 (2 H, td, *J* = 6.8, 1.5 Hz), 3.28 (2 H, t, *J* = 6.1 Hz), 3.27 (2 H, s), 2.97 – 2.84 (2 H, m), 1.56 – 1.38 (7 H, m), 1.37 – 1.21 (3 H, m); ^{13}C NMR (101 MHz, $CDCl_3$) δ: 139.6, 134.0, 129.6, 128.0, 65.3, 57.3, 55.4, 34.7, 32.6, 26.1, 21.7; m/z HRMS found $[M + H]^+$ 298.1469, $C_{15}H_{24}NO_3S$ requires 298.1471.

Step 2

(1-((2-(phenylsulfonyl)ethyl)amino)cyclohexyl)methanol (1.20 g, 4.0 mmol) was dissolved in CH_2Cl_2 (10 mL) and cooled to 0 °C. Triethylamine (1.11 mL, 8.0 mmol) was added followed by drop-wise addition of trimethylacetyl chloride (0.59 mL, 4.8 mmol). The reaction mixture was allowed to warm slowly to ambient temperature and stirred for 16 h after which it was quenched with water (10 mL) and extracted with CH_2Cl_2 (2 x 10 mL). The combined organic phases were washed with brine, dried ($MgSO_4$) and concentrated *in vacuo*. The resulting residue was purified by flash column chromatography (40% EtOAc in P.E.) to afford the title compound as a viscous colourless oil (1.52 g, quant.). IR ν_{max}/cm^{-1} (film): 2933, 2857, 1722, 1479, 1449, 1397, 1362, 1304, 1282, 1142, 1082, 1035; ^1H NMR (400 MHz, $CDCl_3$) δ: 7.94 – 7.90 (2 H, m), 7.69 – 7.63 (1 H, m), 7.60 – 7.54 (2 H, m), 3.89 (2 H, s), 3.27 (2 H, t, *J* = 6.3 Hz), 2.93 (2 H, t, *J* = 6.3 Hz), 1.62 – 1.25 (10 H, m), 1.20 (9 H, s); ^{13}C NMR (101 MHz, $CDCl_3$) δ: 178.5, 139.7, 133.9, 129.5, 128.1, 68.0, 57.6, 54.3, 39.1, 35.4, 32.5, 27.4, 26.0, 21.3; m/z HRMS found $[M + H]^+$ 382.2046, $C_{20}H_{32}NO_4S$ requires 382.2047.

(1-((3-(pyridin-2-yl)propyl)amino)cyclohexyl)methyl pivalate (1j)

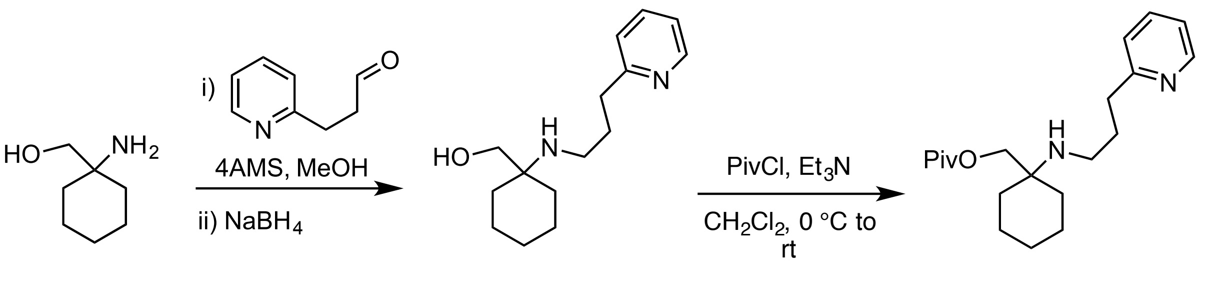

Step 1

To a solution of (1-aminocyclohexyl)methanol[2] (0.646 g, 5.00 mmol) in MeOH (10 mL) was added powdered 4Å molecular sieves (~1 g) and 3-(pyridin-2-yl)propanal[4] (0.811 g, 6.00 mmol) and the mixture stirred at ambient temperature for 16 hours. The mixture was cooled to 0 °C, sodium borohydride (0.227 g, 6.00 mmol) was added portion-wise and mixture allowed to warm to ambient temperature and stirred for an additional 3 h. The reaction mixture was filtered and the MeOH removed *in vacuo*. 10% aqueous NaOH solution was added and the mixture extracted with EtOAc (3 x 10 mL). The combined organic phases were dried ($MgSO_4$) and concentrated *in vacuo* to afford the crude product, which was purified by flash column chromatography (10% MeOH in CH_2Cl_2) to afford the desired compound as a light brown viscous oil (923 mg, 74%); ^1H NMR (400 MHz, $CDCl_3$) δ: 8.49 (1 H, d, *J* = 4.9 Hz), 7.60 (1 H, td, *J* = 7.7, 1.8 Hz), 7.16 (1 H, d, *J* = 7.8 Hz), 7.11 (1 H, dd, *J* = 7.4, 5.0 Hz), 3.28 (2 H, s), 2.88 (2 H, t, *J* = 7.3 Hz), 2.47 (2 H, t, *J* = 6.5 Hz), 1.89 (2 H, qt, *J* = 6.9 Hz), 1.52 – 1.27 (10 H, m); ^{13}C NMR (101 MHz, $CDCl_3$) δ: 161.8, 149.1, 136.8, 123.1, 121.3, 64.9, 55.0, 39.0, 35.5, 32.8, 30.8, 26.2, 21.8.

Step 2

(1-((3-(pyridin-2-yl)propyl)amino)cyclohexyl)methanol (0.90 g, 3.60 mmol) was dissolved in CH_2Cl_2 (7 mL) and cooled to 0 °C. Triethylamine (1.00 mL, 7.2 mmol) was added followed by drop-wise addition of trimethylacetyl chloride (0.532 mL, 4.32 mmol). The reaction mixture was allowed to warm slowly to ambient temperature and stirred for 16 h after which it was quenched with water (7 mL) and extracted with CH_2Cl_2 (2 x 10 mL). The combined organic phases were washed with brine (10 mL), dried ($MgSO_4$) and concentrated *in vacuo*. The resulting residue was purified by flash column chromatography (5% MeOH in CH_2Cl_2) to afford the title compound as a light yellow oil (0.802 g, 67%). IR ν_{max}/cm^{-1} (film): 2933, 2853, 1728, 1590, 1570, 1477, 1435, 1397, 1364, 1282, 1154, 1102, 1035; ^1H NMR (400 MHz, $CDCl_3$) δ: 8.50 (1 H, dd, *J* = 4.8, 0.8 Hz), 7.57 (1 H, td, *J* = 7.7, 1.8 Hz), 7.14 (1 H, d, *J* = 7.8 Hz), 7.08 (1 H, dd, *J* = 7.3, 5.6 Hz), 3.91 (2 H, s), 2.86 (2 H, t, *J* =

---

[4] Kitbunnadaj, R.; Zuiderveld, O.P.; Christophe, B.; Hulscher, S.; Menge, W. M. P.B.; Gelens, E.; Snip, E.; Bakker, R. A.; Celanire, S.; Gillard, M.; Talaga, P.; Timmerman, H.; Leurs; R. *J. Med Chem.*, **2004**, 2414.

7.8 Hz), 2.53 (2 H, t, J = 6.9 Hz), 1.86 (2 H, qt, J = 7.3 Hz), 1.63 – 1.27 (10 H, m), 1.18 (9 H, s); ^{13}C NMR (101 MHz, $CDCl_3$) δ: 178.5, 162.2, 149.3, 136.4, 122.8, 121.1, 68.0, 53.8, 40.7, 39.1, 36.4, 32.8, 31.3, 27.4, 26.2, 21.4; m/z HRMS found $[M + H]^+$ 333.2535, $C_{20}H_{33}N_2O_2$ requires 333.2527.

***N*-cyclohexyl-1-(((triisopropylsilyl)oxy)methyl)cyclohexan-1-amine (1k)**

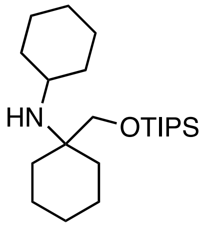

Cyclohexanone (0.40 mL, 3.85 mmol) was dissolved in titanium(IV) isopropoxide (1.55 mL, 5.25 mmol), 1-(((triisopropylsilyl)oxy)methyl)cyclohexan-1-amine (1.00 g, 3.50 mmol) was added and the reaction mixture stirred at room temperature overnight before cooling to 0 °C and adding methanol (15 mL). Sodium borohydride (0.24 g, 6.30 mmol) was added portion-wise and the reaction mixture stirred at 0 °C for 20 minutes then ambient temperature for 1 h. The reaction was quenched by the addition of 10% aqueous NaOH (15 mL) and then diluted with CH_2Cl_2 (15 mL). The mixture was filtered through Celite, eluting with CH_2Cl_2 (30 mL). The organic phase was separated, dried over $MgSO_4$ and concentrated *in vacuo*. The resulting crude product was purified by flash column chromatography (20% EtOAc in P.E) followed by SCX column, loaded in MeOH, washed with MeOH and eluted with ammonia in MeOH solution (2 M, 50 mL) to afford the title compound as a colourless oil (0.164 g, 13%). IR ν_{max}/cm^{-1} (film): 2930, 2864, 1462, 1383, 1247, 1093, 1068, 1012; ^{1}H NMR (400 MHz, $CDCl_3$) δ: 3.53 (2 H, s), 2.46 (1 H, tt, J = 10.2, 3.7 Hz), 1.77 (2 H, app d, J = 11.7 Hz), 1.68 (2 H, dt, J = 12.9, 2.9 Hz), 1.63 – 1.52 (3 H, m), 1.45 – 1.12 (13 H, m), 1.10 – 1.02 (21 H, m).^{13}C NMR (101 MHz, $CDCl_3$) δ: 67.2, 56.2, 50.1, 37.0, 33.4, 26.4, 26.0, 25.9, 22.3, 18.3, 12.2; m/z HRMS found $[M + H]^+$ 368.3336, $C_{22}H_{46}NOSi$ requires 368.3343.

(1-(methylamino)cyclohexyl)methanol

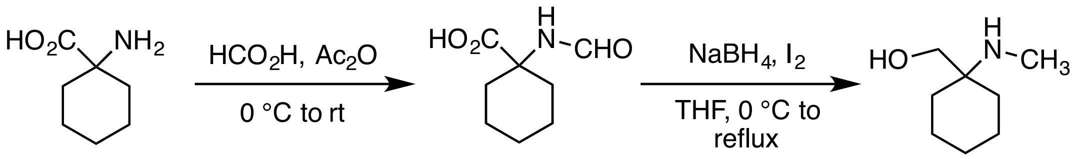

Step 1

To a dry 250 mL 3-neck round-bottomed flask equipped with pressure-equalizing addition funnel, thermometer and nitrogen inlet was added 1-aminocyclohexane-1-carboxylic acid (7.16 g, 50 mmol)

and formic acid (> 95%, 125 mL). Acetic anhydride (47.5 mL) was added dropwise over 30 minutes ensuring the temperature remained below 50 °C. The solution was stirred at room temperature for 90 minutes and was quenched with ice water (130 mL). The solvent was removed *in vacuo* to give the crude *N*-formyl amino acid which was used directly in the next step without further purification (quantitative yield). ^{1}H NMR (400 MHz, $(CD_3)_2SO$) δ: 12.02 (1 H, br s), 7.94 (1 H, s), 1.99 – 1.88 (2 H, s), 1.70 – 1.58 (2 H, m), 1.56 – 1.39 (6 H, m).

Step 2

To a dry 250 mL 3-neck round-bottomed flask equipped with pressure-equalizing addition funnel and condenser under nitrogen was added *N*-formyl amino acid (6.84 g, 40 mmol), sodium borohydride (4.26 g, 112.2 mmol) and anhydrous THF (100 mL). The mixture was cooled to 0 °C and a solution of iodine (11.85 g, 46.6 mmol) in anhydrous THF (40 mL) was added dropwise with vigorous stirring (*Caution – vigorous gas evolution can be delayed!*). Once gas evolution had ceased the mixture was heated to a vigorous reflux for 18 hours. The mixture was cooled to room temperature and then to 0 °C and quenched cautiously by the dropwise addition of methanol. Once the solution had become clear, the solvent was removed *in vacuo* and the residue stirred with a mixture of 20% aqueous KOH (50 mL) and CH_2Cl_2 (50 mL) for 36 hours. The mixture was diluted with brine (100 mL), CH_2Cl_2 (50 mL) and the organic layer separated. The aqueous was extracted with additional CH_2Cl_2 (3 x 50 mL), the organics were combined, dried over $MgSO_4$ and the solvent removed *in vacuo* to give the crude amino alcohol as a colorless oil (5.29 g, 92%) which was used in the next step without further purification. IR ν_{max}/cm^{-1} (film): 3304 (br), 2927, 2855, 1449, 1361, 1301, 1118, 1064, 1040. ^{1}H NMR (400 MHz, $CDCl_3$) δ: 3.29 (2 H, s), 2.23 (3 H, s), 1.51 – 1.32 (10 H, m). ^{13}C NMR (101 MHz, CDCl3) δ: 64.7, 54.9, 32.1, 27.2, 26.1, 21.8. m/z HRMS found $[M + H]^+$ 144.1381, $C_8H_{18}ON$ requires 144.1383

(1-(methylamino)cyclohexyl)methyl pivalate (1m)

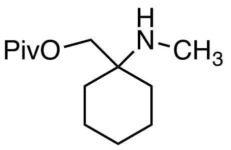

To a solution of (1-(methylamino)cyclohexyl)methanol (0.47 g, 3.3 mmol) in anhydrous CH_2Cl_2 (30 mL) was added triethylamine (0.92 mL, 6.6 mmol) and trimethylacetyl chloride (0.45 mL, 3.6 mmol) dropwise at 0 °C. The reaction mixture was warmed to room temperature over 16 hours and quenched by the addition of saturated aqueous $NaHCO_3$ (30 mL). The organic layer was separated and washed with additional saturated aqueous $NaHCO_3$ (2 x 30 mL), brine (30 mL) and dried over $MgSO_4$. The solvent was removed *in vacuo* and the crude oil purified by flash column chromatography (EtOAc) to

afford the product as a colourless oil (0.33 g, 44%). IR ν_{max}/cm^{-1} (film): 2931, 2855, 1728, 1480, 1463, 1397, 1364, 1282, 1150. ^{1}H NMR (400 MHz, $CDCl_3$) δ: 3.77 (2 H, s), 2.09 (3 H, s), 1.43 – 1.16 (10 H, m), 1.04 (9 H, s). ^{13}C NMR (101 MHz, $CDCl_3$) δ: 177.9, 66.8, 53.5, 38.7, 32.0, 27.6, 27.0, 25.8, 21.1. m/z HRMS found [M + H]$^+$ 228.1953, $C_{13}H_{26}O_2N$ requires 228.1958.

_N_-methyl-1-(((triisopropylsilyl)oxy)methyl)cyclohexan-1-amine (1n)

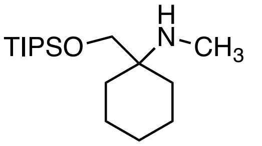

Triisopropylsilyl chloride (7.31 mL, 34.16 mmol) was added dropwise to a solution of (1-(methylamino)cyclohexyl)methanol (5.15 g, 36.0 mmol) and triethylamine (7.52 mL, 53.9 mmol) in anhydrous CH_2Cl_2 (100 mL) at 0 ºC. The resulting solution was allowed to warm to room temperature over 16 hours and was quenched with H_2O (100 mL). The organics were separated and washed with additional H_2O (2 x 100 mL) and brine (100 mL), dried over Na_2SO_4 and removed *in vacuo*. The crude oil was purified by flash column chromatography (gradient elution: 1% MeOH in CH_2Cl_2 to 5% MeOH in CH_2Cl_2) to provide the desired amine as pale yellow oil (5.56 g, 52%). IR ν_{max}/cm^{-1} (film): 2929, 2865, 1463, 1383, 1367, 1307, 1254, 1123, 1090, 1057. ^{1}H NMR (400 MHz, $CDCl_3$) δ: 3.51 (2 H, s), 2.25 (3 H, s), 1.85 (1 H, br s), 1.61 – 1.52 (2 H, m), 1.50 – 1.43 (3 H, m), 1.39 – 1.31 (5 H, m), 1.09 – 1.04 (21 H, m). ^{13}C NMR (101 MHz, $CDCl_3$) δ: 65.8, 55.3, 32.0, 27.9, 26.4, 21.8, 18.2, 12.1. m/z HRMS found [M + H]$^+$ 300.2715, $C_{17}H_{38}NOSi$ requires 300.2717

(4-(methylamino)tetrahydro-2H-pyran-4-yl)methyl pivalate (1o)

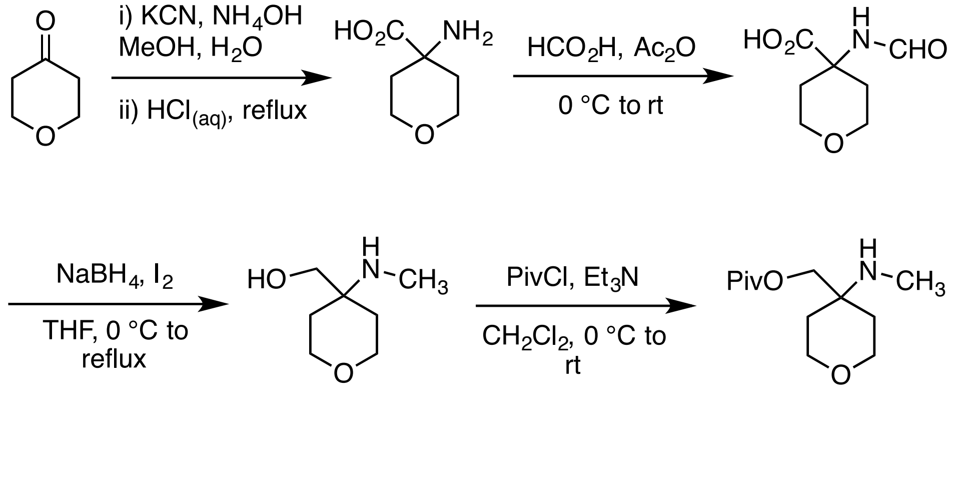

Step 1

A solution of tetrahydro-4H-pyran-4-one (2.5 mL, 27.1 mmol) in methanol (16 mL) was added to a vigorously stirred solution of potassium cyanide (1.76 g, 27.1 mmol), ammonium hydroxide solution (35%, 8.0 mL) and ammonium chloride (1.60 g, 29.8 mmol) in H_2O (5.5 mL) at room temperature. The reaction mixture was stirred overnight, diluted with CH_2Cl_2 (20 mL) and the organic layer separated. The aqueous phase was extracted with CH_2Cl_2 (2 x 20 mL), the organics were combined, dried over $MgSO_4$ and removed *in vacuo*. The resulting product was obtained as yellow oil (3.07 g, 90%) and used without further purification. ^{1}H NMR (400 MHz, $CDCl_3$) δ: 3.98 (2 H, dt, J = 12.4, 4.1 Hz), 3.66 (2 H, ddd, J = 12.4, 10.3, 2.5 Hz), 2.02 – 1.95 (2H, m), 1.75 (2 H, ddd, J = 13.9, 10.3, 4.1 Hz).

Step 2

The crude product was treated with conc. HCl (80 mL) and water (40 mL) and refluxed for 16 hours. The reaction mixture was subsequently cooled to room temperature and the solvent removed *in vacuo*. The resulting solid was dissolved in the minimum amount of water (80 ºC) and was treated with 10% aquous NaOH until pH = 6. The mixture was cooled to 0 ºC and the resulting precipitate removed by vacuum filtration to afford the product as a pale brown solid (2.40 g, 69%). (If no precipitate is formed the solvent is removed *in vacuo*, the residue taken up in ethanol and filtered, and the resulting filtrate removed *in vacuo* to afford the product). ^{1}H NMR (400 MHz, D_2O) δ: 2.89 – 2.76 (4 H m), 2.41 – 2.32 (2 H, m), 2.09 – 2.03 (2 H, m).

Step 3

Acetic anhydride (6 mL) was added dropwise to a solution of crude amino acid (1.05 g, 8.7 mmol) in formic acid (> 95%, 17 mL) at room temperature. After the addition was complete the reaction mixture was stirred for an additional hour and quenched by the addition of ice water (10 mL). The solvent was removed *in vacuo* to give the crude *N*-formyl amino acid which was used directly in the next step without further purification (quantitative yield). ^{1}H NMR (400 MHz, $(CD_3)_2SO$) δ: 7.96 (1 H, s), 2.81 – 2.70 (2 H, m), 2.48 – 2.43 (2 H, m), 2.29 – 2.21 (2 H, m), 1.97 – 1.88 (2 H, m).

Step 4

To a vigorously stirred suspension of *N*-formyl amino acid (1.25 g, 7.2 mmol) and sodium borohydride (0.77 g, 20.2 mmol) in anhydrous THF (20 mL) was added a solution of iodine (2.2 g, 8.7 mmol) in anhydrous THF (10 mL) dropwise at 0 °C. Once gas evolution had ceased the mixture was heated at reflux for 18 hours and subsequently cooled to room temperature. Methanol was added cautiously until the solution became clear and the solvent was subsequently removed *in vacuo* to afford a white paste. The paste was dissolved in the minimum amount of 20% aqueous KOH (ca. 5

mL) and stirred at room temperature for 1 hour. The mixture was diluted with 20% aqueous KOH (10 mL) and extracted with CH_2Cl_2 (3 x 30 mL). The organics were combined, dried over $MgSO_4$ and removed *in vacuo* to give the crude amino alcohol as a colorless oil (0.57 g, 54%) which was used in the next step without further purification ^{1}H NMR (400 MHz, $CDCl_3$) δ: 3.38 (2 H, s), 2.77 – 2.68 (2 H, m), 2.59 – 2.50 (2 H, m), 2.29 (3 H, s), 1.95 – 1.81 (2 H, m), 1.77 – 1.67 (2 H, m).

Step 5

To a solution of crude amino alcohol (0.57 g, 3.9 mmol) in anhydrous CH_2Cl_2 (30 mL) was added triethylamine (1.1 mL, 7.9 mmol) and trimethylacetyl chloride (0.53 mL, 4.3 mmol) dropwise at 0 °C. The reaction mixture was warmed to room temperature over 16 hours and quenched by the addition of saturated aqueous $NaHCO_3$ (20 mL). The organic layer was separated and washed with additional saturated aqueous $NaHCO_3$ (2 x 20 mL), brine (20 mL) and dried over $MgSO_4$. The solvent was removed *in vacuo* and the crude oil purified by flash column chromatography (EtOAc) to afford the product as a colourless oil (0.32 g, 36%). IR ν_{max}/cm^{-1} (film): 2959, 2928, 1726, 1480, 1460, 1428, 1387, 1365, 1281, 1152, 1102, 1078, 1036. ^{1}H NMR (400 MHz, $CDCl_3$) δ: 3.80 (2 H, s), 2.91 (2 H, ddd, J = 2.6, 11.5, 13.7 Hz), 2.24 (2 H, dt, J = 3.7, 13.6 Hz), 2.15 (3 H, s), 1.76 (2 H, dt, J = 3.2, 14.3 Hz), 1.57 (2 H, ddd, J = 3.4, 11.4, 13.4 Hz), 1.12 (9 H, s), 0.78 (1 H, br s). ^{13}C NMR (101 MHz, CDCl3) δ: 178.0, 67.4, 52.6, 38.9, 33.2, 27.3, 27.1, 22.8. m/z HRMS found $[M + H]^+$ 230.1749, $C_{12}H_{24}O_3N$ requires 230.1751.

(4-(methylamino)tetrahydro-2H-thiopyran-4-yl)methyl pivalate (1p)

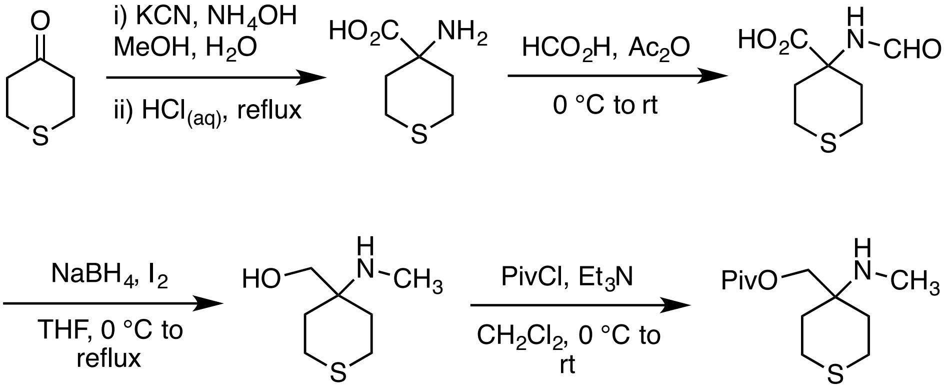

Step 1

A solution of tetrahydro-4H-thiopyran-4-one (2.0 g, 17.2 mmol) in methanol (10 mL) was added to a vigorously stirred solution of potassium cyanide (1.12 g, 17.2 mmol), ammonium hydroxide solution (35%, 5.0 mL) and ammonium chloride (1.01 g, 18.9 mmol) in H_2O (3.5 mL) at room temperature. The reaction mixture was stirred overnight, diluted with CH_2Cl_2 (15 mL) and the organic layer

separated. The aqueous was extracted with CH_2Cl_2 (2 x 15 mL), the organics were combined, dried over $MgSO_4$ and removed *in vacuo*. The resulting product was obtained as brown oil (2.18 g, 89%) and used without further purification. ^1H NMR (400 MHz, $CDCl_3$) δ: 2.91 – 2.82 (2 H, m), 2.79 – 2.69 (2 H, m), 2.32 – 2.22 (2 H, m), 1.91 – 1.82 (2 H, m).

Step 2

The crude product was treated with conc. HCl (50 mL) and water (25 mL) and refluxed for 16 hours. The reaction mixture was subsequently cooled to room temperature and the solvent removed *in vacuo*. The resulting solid was dissolved in the minimum amount of water (80 ºC) and was treated with 10% aquous NaOH until pH = 6. The mixture was cooled to 0 ºC and the resulting precipitate removed by vacuum filtration to afford the product as a pale brown solid (2.08 g, 84%). (If no precipitate is formed the solvent is removed *in vacuo*, the residue taken up in ethanol and filtered, and the resulting filtrate removed *in vacuo* to afford the product). ^1H NMR (400 MHz, D_2O) δ: 4.04 – 3.79 (4 H, m), 2.31 – 2.19 (2 H, m), 1.91 – 1.83 (2 H, m).

Step 3

Acetic anhydride (8 mL) was added dropwise to a solution of crude amino acid (1.50 g, 9.3 mmol) in formic acid (> 95%, 18 mL) at room temperature. After the addition was complete the reaction mixture was stirred for 1 hour and quenched by the addition of ice water (10 mL). The solvent was removed *in vacuo* to give the crude *N*-formyl amino acid which was used directly in the next step without further purification (quantitative yield). ^1H NMR (400 MHz, $(CD_3)_2SO$) δ: 7.98 (1 H, s), 3.74 – 3.60 (2 H, m), 3.58 – 3.50 (2 H, m), 1.91 – 1.83 (4 H, m).

Step 4

To a vigorously stirred suspension of *N*-formyl amino acid (1.70 g, 9.0 mmol) and sodium borohydride (0.95 g, 25.2 mmol) in anhydrous THF (25 mL) was added a solution of iodine (2.74 g, 10.8 mmol) in anhydrous THF (10 mL) dropwise at 0 °C. Once gas evolution had ceased the mixture was heated at reflux for 18 hours and subsequently cooled to room temperature. Methanol was added cautiously until the solution became clear and the solvent was subsequently removed *in vacuo* to afford a white paste. The paste was dissolved in the minimum amount of 20% aqueous KOH (ca. 7 mL) and stirred at room temperature for 1 hour. The mixture was diluted with 20% aqueous KOH (15 mL) and extracted with CH_2Cl_2 (3 x 40 mL). The organics were combined, dried over $MgSO_4$ and removed *in vacuo* to give the crude amino alcohol as a yellow oil (0.50 g, 39%) which was used in the next step without further purification ^1H NMR (400 MHz, $CDCl_3$) δ: 3.75 – 3.62 (4 H, m), 3.43 (2 H, s), 2.30 (3 H, s), 1.62 – 1.54 (4 H, m).

Step 5

To a solution of crude amino alcohol (0.49 g, 3.0 mmol) in anhydrous CH_2Cl_2 (30 mL) was added triethylamine (0.9 mL, 6.2 mmol) and trimethylacetyl chloride (0.42 mL, 3.4 mmol) dropwise at 0 °C. The reaction mixture was warmed to room temperature over 16 hours and quenched by the addition of saturated aqueous $NaHCO_3$ (20 mL). The organic layer was separated and washed with additional saturated aqueous $NaHCO_3$ (2 x 20 mL), brine (20 mL) and dried over $MgSO_4$. The solvent was removed *in vacuo* and the crude oil purified by flash column chromatography (EtOAc) to afford the product as a colourless oil (0.25 g, 33%). IR ν_{max}/cm^{-1} (film): 2956, 2867, 1727, 1480, 1463, 1397, 1364, 1282, 1238, 1151, 1110, 1091, 1033. ^1H NMR (400 MHz, $CDCl_3$) δ: 3.90 (2 H, s), 3.73 (2 H, app td, *J* = 2.8, 11.3 Hz), 3.58 (2 H, dt, *J* = 4.3, 11.5 Hz), 2.21 (3 H, s), 1.53 (2 H, ddd, *J* = 4.3, 10.0, 14.3 Hz), 145 – 1.41 (2 H, m), 1.15 (9 H, s), 1.02 (1 H, br s). ^{13}C NMR (101 MHz, $CDCl_3$) δ: 178.2, 66.9, 63.0, 51.9, 39.0, 32.5, 27.8, 27.2. m/z HRMS found $[M + H]^+$ 246.1521, $C_{12}H_{24}O_2NS$ requires 246.1522

(4-(methylamino)-1-tosylpiperidin-4-yl)methyl pivalate (1q)

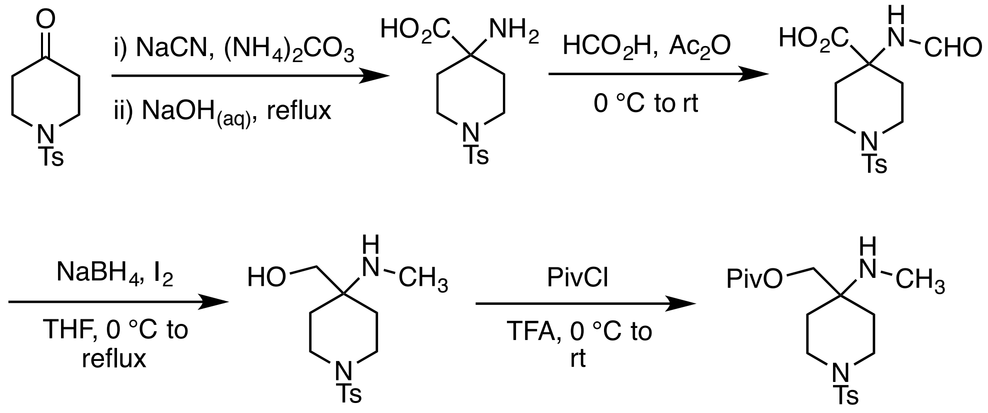

Step 1

1-tosyl-4-piperidone[5] (4.0 g, 15.8 mmol), sodium cyanide (0.85 g, 17.4 mmol), and ammonium carbonate (3.5 g, 75.8 mmol) were suspended in ethanol/water (1:1, 50 mL) and heated at 60 °C for 16 hours. The solution was cooled to room temperature and the resulting precipitate was collected by vacuum filtration, washed with water (50 mL) and dried under vacuum to afford the desired product as a white solid (5.0 g, 98%) which was used directly in the next step without further purification. ^1H NMR (400 MHz, $(CD_3)_2SO$) δ: 10.66 (1 H, br s), 8.42 (1 H, s), 7.63 (2 H, d, *J* = 8.0 Hz), 7.46 (2 H, d, *J* = 8.0 Hz), 3.50 – 3.42 (2 H, m), 2.79 – 2.70 (2 H, m), 2.42 (3 H, s), 1.92 – 1.75 (2 H, m), 1.69 – 1.56 (2 H, m).

---

[5] Pagenkopf, B. L.; Moustafa, M. *Org. Lett.*, **2010**, *12*, 3168

Step 2

Crude hydantoin (5.0 g, 15.5 mmol) was suspended in aqueous sodium hydroxide (5 M, 25 mL) and heated at 120 °C for 24 hours. The solution was cooled to room temperature and the solid removed by filtration. The mother liquor was acidified to pH = 6 and the resulting solid removed by vacuum filtration to afford the product as a white solid (3.81 g, 85%). ^{1}H NMR (400 MHz, CD_3OD) δ: 7.70 (2 H, d, *J* = 8.2 Hz), 7.45 (2 H, d, *J* = 8.2 Hz), 3.37 – 3.32 (2 H, m), 3.28 – 3.21 (2 H, m), 2.46 (3 H, s), 2.32 – 2.20 (2 H, m), 1.85 (2 H, m).

Step 3

Acetic anhydride (4 mL) was added dropwise to a solution of crude amino acid (1.40 g, 4.7 mmol) in formic acid (> 95%, 10 mL) at room temperature. After the addition was complete the reaction mixture was stirred for 1 hour and quenched by the addition of ice water (10 mL). The solvent was removed *in vacuo* to give the crude *N*-formyl amino acid which was used directly in the next step without further purification (1.43 g, 93%). ^{1}H NMR (400 MHz, $(CD_3)_2SO$) δ: 7.85 (1 H, s), 7.63 (2 H, d, *J* = 8.2 Hz), 7.45 (4 H, d, *J* = 8.2 Hz), (4 H, obscured), 2.41 (3 H, s), 2.05 – 1.97 (2 H, m), 1.94 – 1.86 (2 H, m).

Step 4

To a vigorously stirred suspension of *N*-formyl amino acid (1.35 g, 4.1 mmol) and sodium borohydride (0.44 g, 11.6 mmol) in anhydrous THF (11 mL) was added a solution of iodine (1.26 g, 4.8 mmol) in anhydrous THF (5 mL) dropwise at 0 °C. Once gas evolution had ceased the mixture was heated at reflux for 18 hours and subsequently cooled to room temperature. Methanol was added cautiously until the solution became clear and the solvent was subsequently removed *in vacuo* to afford a white paste. The paste was dissolved in the minimum amount of 20% aqueous KOH (ca. 5 mL) and stirred at room temperature for 1 hour. The mixture was diluted with 20% aqueous KOH (10 mL) and extracted with CH_2Cl_2 (3 x 20 mL). The organics were combined, dried over $MgSO_4$ and removed *in vacuo* to give the crude amino alcohol as a white solid (0.52 g, 42%) which was used in the next step without further purification ^{1}H NMR (400 MHz, $CDCl_3$) δ: 7.64 (2 H, d, *J* = 8.1 Hz), 7.32 (2 H, d, *J* = 8.1 Hz), 3.29 (2 H, d, *J* = 5.0 Hz), 3.25 – 3.16 (2 H, m), 2.94 – 2.81 (2 H, m), 2.45 (3 H, s), 2.18 (3 H, s), 1.63 – 1.54 (4 H, m).

Step 5

A 10 mL round-bottomed flask was charged with crude amino alcohol (0.50 g, 1.7 mmol) and cooled in an ice bath. Trifluoroacetic acid (1 mL) was added dropwise with vigorous stirring to afford a viscous solution. The reaction mixture was stirred at 0 °C for 5 minutes and trimethylacetyl chloride (0.30 mL, 2.5 mmol) was added dropwise. The flask was sealed with a glass-stopper and warmed to

room temperature over 16 hours. The solvent was removed *in vacuo,* the residue was dissolved in diethyl ether (10 mL) and treated with triethylamine until pH = 9. Water (10 mL) was added and the layers were separated and the aqueous was extracted with diethyl ether (3 x 10 mL). The organics were combined, dried over $MgSO_4$ and removed *in vacuo*. The crude product was purified by flash column chromatography (30% EtOAc in CH_2Cl_2) to afford the product as a white solid (0.26 g, 41%). IR ν_{max}/cm^{-1} (film): 2971, 2862, 1722, 1661, 1598, 1475, 1461, 1432, 1398, 1347, 1330, 1281, 1246, 1160, 1090. ^{1}H NMR (400 MHz, $CDCl_3$) δ: 7.59 (2 H, d, *J* = 8.1 Hz), 7.27 (2 H, d, *J* = 8.1 Hz), 3.82 (2 H, s), 3.43 – 3.33 (2 H, m), 2.79 – 2.70 (2 H, m), 2.38 (3 H, s), 2.09 (3 H, s), 1.58 – 1.51 (4 H, m), 1.14 (9 H, s), 0.96 (1 H, br s). ^{13}C NMR (101 MHz, $CDCl_3$) δ:177.9, 143.3, 133.6, 129.6, 127.5, 66.7, 51.8, 41.2, 38.9, 31.1, 27.5, 27.1, 21.4. m/z HRMS found $[M + H]^+$ 383.1997, $C_{19}H_{31}O_4N_2S$ requires 383.1999.

2-ethyl-2-(methylamino)butyl pivalate (1r)

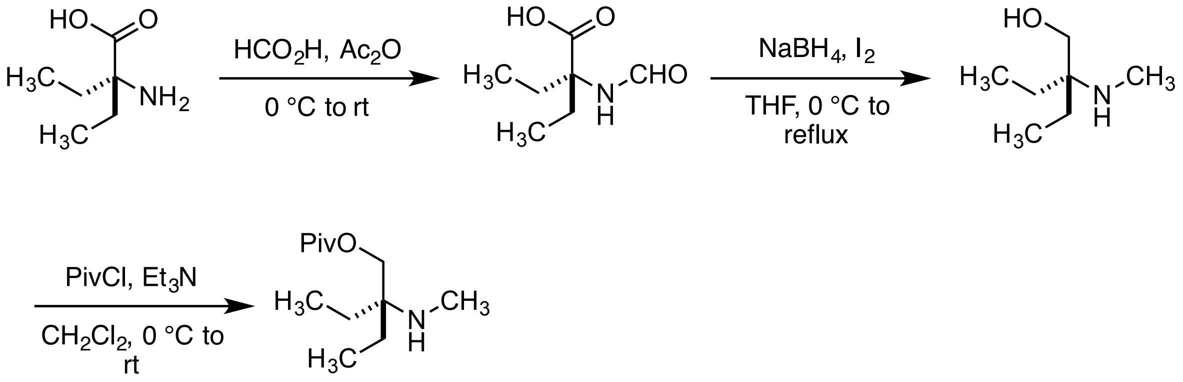

Step 1

Acetic anhydride (10 mL) was added drop-wise to a solution of 2-amino-2-ethylbutanoic acid (2.00 g, 15.2 mmol) in formic acid (> 95%, 32 mL) at 0 °C. After the addition was complete the reaction mixture allowed to warm to room temperature, stirred for 14 h and quenched by the addition of ice water (10 mL). The solvent was removed *in vacuo* to give the crude *N*-formyl amino acid as a white solid which was used directly in the next step without further purification (2.01 g, 87%). ^{1}H NMR (400 MHz, $(CD_3)_2SO$) δ: 8.24 (1 H, s), 1.77 – 1.57 (4 H, m), 0.85 (6 H, t, *J* = 7.5 Hz).

Step 2

In a 3-necked flask, to a vigorously stirred suspension of *N*-formyl amino acid (2.01 g, 13.2 mmol) and sodium borohydride (1.40 g, 37.0 mmol) in anhydrous THF (35 mL) was added a solution of iodine (4.01 g, 15.8 mmol) in anhydrous THF (15 mL) dropwise at 0 °C. Once gas evolution had ceased the mixture was heated at reflux for 18 hours and subsequently cooled to room temperature. MeOH was added cautiously until the solution became clear and the solvent was subsequently

removed *in vacuo* to afford a white paste. The paste was dissolved in 20% aqueous KOH (30 mL) and stirred at room temperature for 1.5 h. The mixture was extracted with CH_2Cl_2 (3 x 40 mL). The organics were combined, washed with brine (40 mL), dried over $MgSO_4$ and concentrated *in vacuo* to give the crude amino alcohol as a colourless oil (0.901 g, 52%) which was used in the next step without further purification. ^{1}H NMR (400 MHz, $CDCl_3$) δ: 3.29 (2 H, s), 2.22 (3 H, s), 1.58 – 1.51 (4 H, m), 0.93 (6 H, t, *J* = 7.3 Hz).

Step 3

2-ethyl-2-(methylamino)butan-1-ol (0.90 g, 6.86 mmol) was dissolved in CH_2Cl_2 (10 mL) and cooled to 0 °C. Triethylamine (1.91 mL, 13.72 mmol) was added followed by dropwise addition of trimethylacetyl chloride (1.01 mL, 8.23 mmol). The reaction mixture was allowed to warm slowly to ambient temperature and stirred for 16 hours after which it was quenched with water (10 mL) and extracted with CH_2Cl_2 (2 x 10 mL). The combined organic phases were washed with brine, dried ($MgSO_4$) and concentrated *in vacuo*. The resulting residue was purified by flash column chromatography (30% EtOAc in P.E.) to afford the title compound as a light yellow oil (0.119 g, 8%). IR ν_{max}/cm^{-1} (film): 2965, 2880, 1730, 1646, 1515, 1480, 1461, 1397, 1365, 1281, 1150, 1034; ^{1}H NMR (400 MHz, $CDCl_3$) δ: 3.94 (2 H, s), 2.30 (3 H, s), 1.45 (4 H, qq, *J* = 14.5, 7.4 Hz), 1.22 (9 H, s), 0.85 (6 H, t, *J* = 7.5 Hz); ^{13}C NMR (101 MHz, $CDCl_3$) δ: 178.4, 65.3, 57.7, 39.1, 28.0, 27.3, 25.3, 7.4; m/z HRMS found $[M + H]^+$ 216.1957, $C_{12}H_{27}NO_2$ requires 216.1958.

N-(2-(1-(methylamino)cyclohexyl)ethyl)phthalimide (1s)

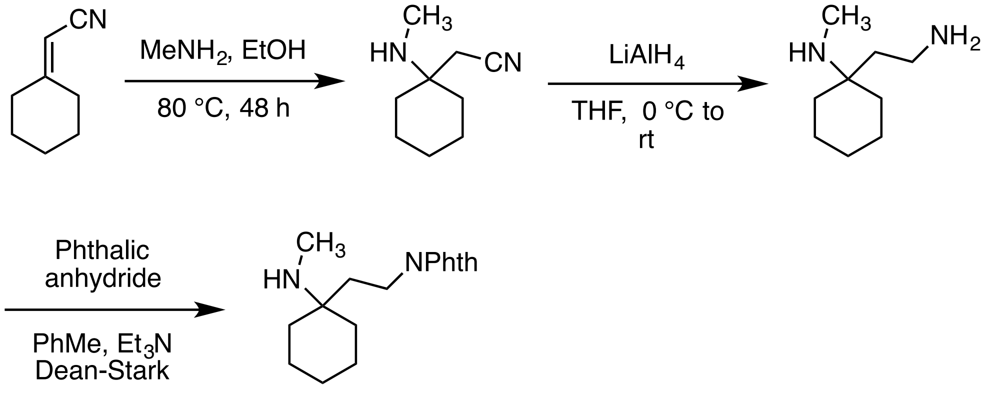

Step 1

A solution of 2-cyclohexylideneacetonitrile[6] (1.89 g, 15.7 mmol) in ethanol (2 mL) and methylamine (33% w/w in EtOH, 3.9 mL, 31.3 mmol) were stirred at 80 ºC in a sealed tube for 48 hours. After such time the solution was cooled to room temperature and the solvent removed *in vacuo*. The crude

[6] Karagiozov, S. K.; Abbott, F. S. *Synth. Commun.*, **2004**, *34*, 871

oil was purified by flash column chromatography (100% EtOAc) to provide the desired amine as a colorless oil (1.45 g, 61%). ^{1}H NMR (400 MHz, $CDCl_3$) δ: 2.43 (2 H, s), 2.29 (3 H, s), 1.66 – 1.23 (10 H, m), 1.18 (1 H, br s (N-H)).

Step 2

To a solution of ethyl 2-(1-(methylamino)cyclohexyl)acetonitrile (1.45 g, 9.5 mmol) in anhydrous Et_2O (50 mL) was added $LiAlH_4$ (0.90 g, 23.8 mmol) portionwise at 0 ºC and then heated at reflux for 16 hours. The resulting solution cooled to warm to room temperature and was quenched successively with H_2O (1 mL), 10% NaOH (1.3 mL) and H_2O (2.5 mL). The resulting slurry was stirred with $MgSO_4$ and filtered. The filter cake was washed with hot CH_2Cl_2 (100 mL). The filter cake was then removed and heated to 100 ºC in 10% NaOH (100 mL) for 2 hours. The mixture was cooled to room temperature, filtered and the solution extracted with CH_2Cl_2 (3 x 100 mL). The combined organics were dried over K_2CO_3 and removed *in vacuo* to afford the crude diamine as a colorless oil (1.16 g, 78%) which was used without further purification. ^{1}H NMR (400 MHz, $CDCl_3$) δ: 2.72 (2 H, t, J = 8.0 Hz), 2.26 (3 H, s), 1.54 – 1.41 (10 H, m), 1.37 – 1.29 (4 H, m).

Step 3

The crude diamine (1.16 g, 7.5 mmol), phthalic anhydride (1.10 g, 7.5 mmol) and triethylamine (1.04 mL, 7.5 mmol) were heated under Dean-Stark conditions in toluene (10 mL) for 6 hours. The solution was cooled to room temperature and the solvent removed *in vacuo* and the resulting oil purified by flash column chromatography (gradient elution: 1% MeOH in CH_2Cl_2 to 5% MeOH in CH_2Cl_2) to provide the desired amine as a pale red viscous oil (1.53 g, 72%). IR ν_{max}/cm^{-1} (film): 3351 (br), 2927, 2854, 2799, 1770, 1706, 1641, 1615, 1559, 1466, 1443, 11396, 1367, 1223, 1187, 1159, 1131. ^{1}H NMR (400 MHz, $CDCl_3$) δ: 7.79 (2 H, dd, J = 3.1, 5.4 Hz), 7.66 (2 H, dd, J = 3.1, 5.4 Hz), 3.71 – 3.66 (2 H, m), 2.32 (3 H, s), 1.70 – 1.66 (2 H, m), 1.52 – 1.32 (10 H, m). ^{13}C NMR (101 MHz, $CDCl_3$) δ 168.5, 133.9, 132.4, 123.1, 53.1, 35.1, 34.2, 33.2, 27.5, 26.1, 21.7. m/z HRMS found [M + H]$^+$ 287.1754, $C_{17}H_{23}O_2N_2$ requires 287.1754.

(1-(methylamino)cycloheptyl)methyl pivalate (1t)

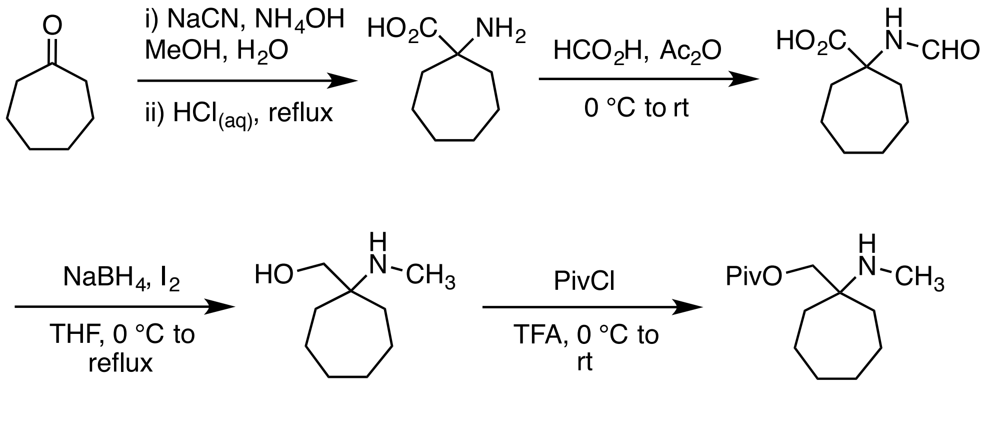

Step 1

A solution of cycloheptanone (2.38 mL, 21.2 mmol) in methanol (12.5 mL) was added to a vigorously stirred solution of sodium cyanide (1.07 g, 21.2 mmol), ammonium hydroxide solution (35%, 7.0 mL) and ammonium chloride (1.25 g, 23.3 mmol) in H_2O (4.2 mL) at room temperature. The reaction mixture was stirred for 16 h, diluted with CH_2Cl_2 (15 mL) and the organic layer separated. The aqueous was extracted with CH_2Cl_2 (2 x 15 mL), the organics were combined, dried over $MgSO_4$ and removed *in vacuo*. The resulting product was obtained as colourless oil (quantitative yield) and used without further purification. ^{1}H NMR (400 MHz, $CDCl_3$) δ: 2.10 – 2.00 (4 H, m), 1.77 – 1.51 (8 H, m).

Step 2

The crude product was treated with conc. HCl (70 mL) and water (35 mL) and refluxed for 16 hours. The reaction mixture was subsequently cooled to room temperature and the solvent removed *in vacuo*. The resulting solid was dissolved in the minimum amount of water (80 ºC) and was treated with 10% aquous NaOH until pH = 6. The mixture was cooled to 0 ºC and the resulting precipitate removed by vacuum filtration to afford the product as a white crystalline solid (1.75 g, 53%). (If no precipitate is formed the solvent is removed *in vacuo*, the residue taken up in ethanol and filtered, and the resulting filtrate removed *in vacuo* to afford the product). ^{1}H NMR (400 MHz, D_2O) 2.24 – 2.15 (2 H, m), 1.86 – 1.78 (2 H, m), 1.78 – 1.70 (2 H, m), 1.65 – 1.54 (6 H, m).

Step 3

Acetic anhydride (8 mL) was added dropwise to a solution of crude amino acid (1.70 g, 10.8 mmol) in formic acid (> 95%, 22 mL) at room temperature. After the addition was complete the reaction mixture was stirred for an additional hour and quenched by the addition of ice water (10 mL). The solvent was removed *in vacuo* to give the crude *N*-formyl amino acid which was used directly in the next step without further purification (1.66 g, 83%). ^{1}H NMR (400 MHz, $(CD_3)_2SO$) δ: 7.86 (1 H, s), 2.03 – 1.84 (4 H, m), 1.53 – 1.42 (8 H, m).

Step 4

To a vigorously stirred suspension of *N*-formyl amino acid (1.60 g, 8.6 mmol) and sodium borohydride (0.92 g, 24.2 mmol) in anhydrous THF (36 mL) was added a solution of iodine (2.36 g, 10.4 mmol) in anhydrous THF (10 mL) dropwise at 0 °C. Once gas evolution had ceased the mixture was heated at reflux for 18 hours and subsequently cooled to room temperature. Methanol was added cautiously until the solution became clear and the solvent was subsequently removed *in vacuo* to afford a white paste. The paste was dissolved in the minimum amount of 20% aqueous KOH (ca. 7 mL) and stirred at room temperature for 1 hour. The mixture was diluted with 20% aqueous KOH (15

mL) and extracted with CH_2Cl_2 (3 x 40 mL). The organics were combined, dried over $MgSO_4$ and removed *in vacuo* to give the crude amino alcohol as a colorless oil (1.29 g, 95%) which was used in the next step without further purification ^{1}H NMR (400 MHz, $CDCl_3$) δ: 3.28 (2 H, s), 2.28 (3 H, s), 1.61 – 1.41 (12 H, m).

Step 5

A 10 mL round-bottomed flask was charged with crude amino alcohol (0.36 g, 2.3 mmol) and cooled in an ice bath. Trifluoroacetic acid (1.2 mL) was added dropwise with vigorous stirring to afford a viscous solution. The reaction mixture was stirred at 0 °C for 5 minutes and trimethylacetyl chloride (0.43 mL, 3.5 mmol) was added dropwise. The flask was sealed with a glass-stopper and warmed to room temperature over 16 hours. The solvent was removed *in vacuo,* the residue was dissolved in diethyl ether (10 mL) and treated with triethylamine until pH = 9. Water (10 mL) was added and the layers were separated and the aqueous was extracted with diethyl ether (3 x 10 mL). The organics were combined, dried over $MgSO_4$ and removed *in vacuo*. The crude product was purified by SCX column, loaded in methanol, washed with methanol and eluted with ammonia in methanol solution (2 M) to afford the product as a colourless oil (0.24 g, 75%). IR ν_{max}/cm^{-1} (film): 2925, 2856, 2802, 1727, 1480, 1463, 1397, 1463, 1397, 1364, 1282, 1154. ^{1}H NMR (400 MHz, $CDCl_3$) δ: 3.86 (2 H, s), 2.22 (3 H, s), 1.54 – 1.37 (13 H, m), 1.17 (9 H, s). ^{13}C NMR (101 MHz, $CDCl_3$) δ: 178.4, 67.3, 57.7, 39.0, 35.3, 30.6, 28.5, 27.3, 22.5. m/z HRMS found $[M + H]^+$ 242.2112, $C_{14}H_{28}O_2N$ requires 242.2115.

(1-(methylamino)cyclopentyl)methyl pivalate (1u)

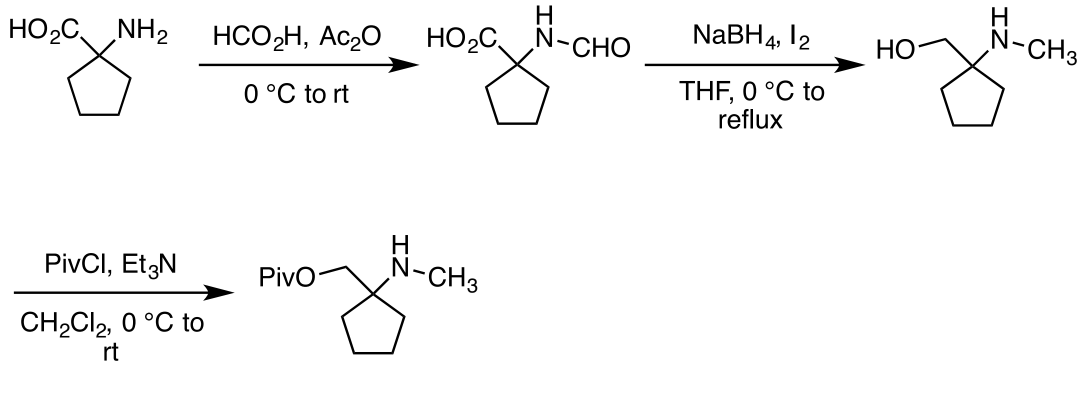

Step 1

Acetic anhydride (12 mL) was added dropwise to a solution of 1-aminocyclopentane-1-carboxylic acid (2.0 g, 15.5 mmol) in formic acid (> 95%, 30 mL) at room temperature. After the addition was complete the reaction mixture was stirred for an additional hour and quenched by the addition of ice water (10 mL). The solvent was removed *in vacuo* to give the crude *N*-formyl amino acid which was

used directly in the next step without further purification (quantitative yield). ^{1}H NMR (400 MHz, $(CD_3)_2SO$) δ: 7.89 (1 H, s), 2.11 – 1.92 (2 H, m), 1.92 – 1.83 (2 H, m), 1.73 – 1.59 (4 H, m).

Step 2

To a vigorously stirred suspension of *N*-formyl amino acid (2.4 g, 15.4 mmol) and sodium borohydride (1.87 g, 49.5 mmol) in anhydrous THF (40 mL) was added a solution of iodine (4.7 g, 18.6 mmol) in anhydrous THF (20 mL) dropwise at 0 °C. Once gas evolution had ceased the mixture was heated at reflux for 18 hours and subsequently cooled to room temperature. Methanol was added cautiously until the solution became clear and the solvent was subsequently removed *in vacuo* to afford a white paste. The paste was dissolved in the minimum amount of 20% aqueous KOH (ca. 10 mL) and stirred at room temperature for 1 hour. The mixture was diluted with 20% aqueous KOH (20 mL) and extracted with CH_2Cl_2 (3 x 50 mL). The organics were combined, dried over $MgSO_4$ and removed *in vacuo* to give the crude amino alcohol as a colorless oil (1.96 g, 97%) which was used in the next step without further purification ^{1}H NMR (400 MHz, $CDCl_3$) δ: 3.40 (2 H, s), 2.33 (3 H, s), 1.65 – 1.51 (8 H, m).

Step 3

To a solution of crude amino alcohol (1.94 g, 15.0 mmol) in anhydrous CH_2Cl_2 (150 mL) was added triethylamine (4.2 mL, 30.0 mmol) and trimethylacetyl chloride (2.0 mL, 16.5 mmol) dropwise at 0 °C. The reaction mixture was warmed to room temperature over 16 hours and quenched by the addition of saturated aqueous $NaHCO_3$ (100 mL). The organic layer was separated and washed with additional saturated aqueous $NaHCO_3$ (2 x 100 mL), brine (100 mL) and dried over $MgSO_4$. The solvent was removed *in vacuo* and the crude oil purified by flash column chromatography (EtOAc) to afford the product as a colourless oil (0.23 g, 7%). IR ν_{max}/cm^{-1} (film): 2958, 2871, 2798, 1728, 1480, 1397, 1365, 1331, 1283, 1151, 1096, 1034. ^{1}H NMR (400 MHz, $CDCl_3$) δ: 3.94 (2 H, s), 2.25 (3 H, s), 1.73 – 1.63 (2 H, m), 1.59 – 1.50 (4 H, m), 1.48 0- 1.41 (3 H, m), 1.16 (9 H, s). ^{13}C NMR (101 MHz, $CDCl_3$) δ: 178.4, 66.8, 64.4, 38.9, 34.8, 29.5, 27.3, 24.7. m/z HRMS found $[M + H]^+$ 214.1799, $C_{12}H_{24}O_2N$ requires 214.1802

***N*-methyl-1-(2-((triisopropylsilyl)oxy)ethyl)cyclobutan-1-amine (1v)**

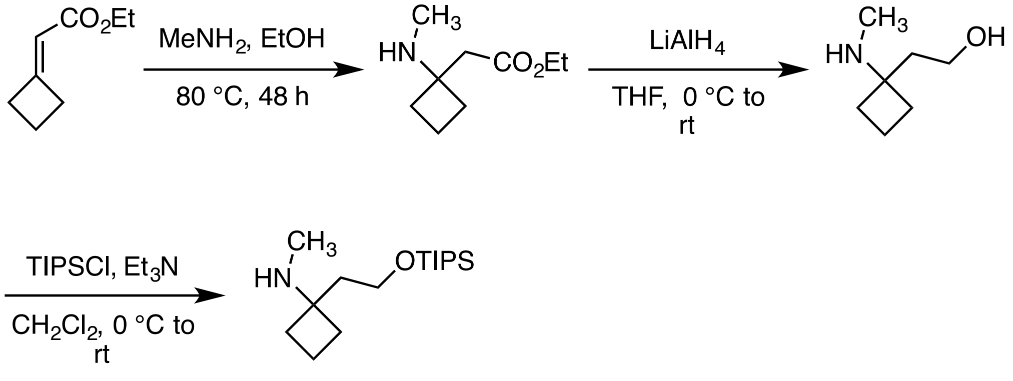

Step 1

A solution of ethyl 2-cyclobutylideneacetate[7] (2.80 g, 20 mmol) and methylamine (33% w/w in EtOH, 4.98 mL, 40 mmol) were stirred at 80 ºC in a sealed tube for 48 hours. After such time the solution was cooled to room temperature and the solvent removed *in vacuo* to afford the product as a colourless oil (3.13 g, 92%) which was used in the next step without further purification. ^{1}H NMR (400 MHz, $CDCl_3$) δ: 4.10 (2 H, q, J = 7.1 Hz), 2.59 (2 H, s), 2.27 (3 H, s), 2.04 – 1.97 (2 H, m), 1.93 – 1.85 (2 H, m), 1.87 – 1.65 (3 H, m, (N-H)), 1.23 (3 H, t, J = 7.1 Hz).

Step 2

To a solution of ethyl 2-(1-(methylamino)cyclobutyl)acetate (3.13 g, 18.3 mmol) in anhydrous THF (60 mL) was added $LiAlH_4$ (1.39 g, 36.6 mmol) portionwise at 0 ºC. The resulting solution was allowed to warm to room temperature over 16 hours and quenched successively with H_2O (1.5 mL), 10% NaOH (2 mL) and H_2O (4 mL). The resulting slurry was stirred with $MgSO_4$ and filtered. The filter cake was washed with Et_2O (100 mL) and the combined organics removed *in vacuo* to afford the crude amino alcohol in quantitative yield, which was used without further purification. ^{1}H NMR (400 MHz, $CDCl_3$) δ: 3.79 (2 H, t, J = 5.3 Hz), 2.29 (3 H, s), 1.97 – 1.70 (8 H, m).

Step 3

Triisopropylsilyl chloride (0.89 mL, 4.18 mmol) was added dropwise to a solution of amino alcohol (0.56 g, 4.4 mmol) and triethylamine (0.92 mL, 6.6 mmol) in anhydrous CH_2Cl_2 (20 mL) at 0 ºC. The resulting solution was allowed to warm to room temperature over 16 hours and was quenched with H_2O (20 mL). The organics were separated and washed with additional H_2O (2 x 20 mL) and brine (20 mL), dried over Na_2SO_4 and removed *in vacuo*. The crude oil was purified by flash column chromatography (gradient elution: 1% MeOH in CH_2Cl_2 to 5% MeOH in CH_2Cl_2) to provide the desired amine as colorless oil (0.55 g, 44%). IR ν_{max}/cm^{-1} (film): 2941, 2866, 1463, 1383, 1246, 1161, 1093, 1068, 1030. ^{1}H NMR (400 MHz, $CDCl_3$) δ: 3.79 (2 H, t, J = 6.9 Hz), 2.41 (1 H, br s), 2.29 (3 H, s), 2.03 – 1.96 (2 H, m), 1.91 – 1.85 (4 H, m), 1.80 – 1.68 (2 H, m), 1.12 – 1.04 (21 H, m). ^{13}C NMR (125 MHz, $CDCl_3$) δ 60.2, 59.4, 38.6, 32.1, 28.8, 18.2, 13.7, 12.1. m/z HRMS found $[M + H]^+$ 286.2559, $C_{16}H_{36}ONSi$ requires 286.2561

[7] Afzal, M.; Walton, John C. *J. Chem, Soc., Perkin Trans.* 2, **1999**, *5*, 937

***endo*-2-(methylamino)norbornane-2-methyl pivalate (1w)**

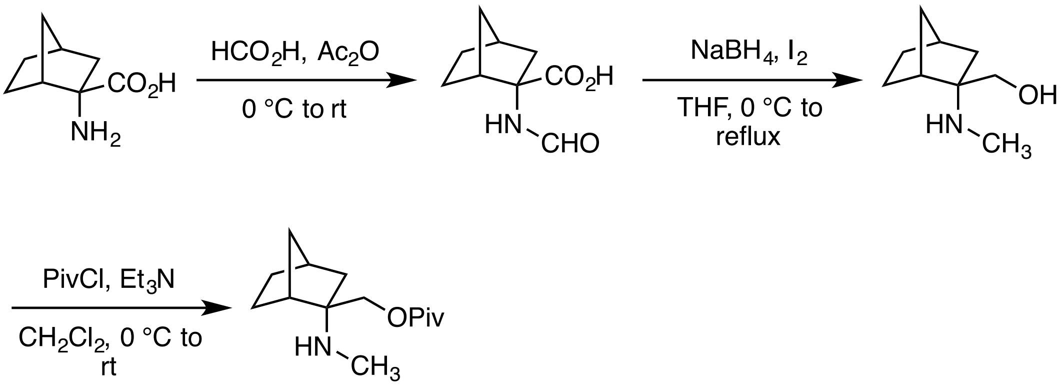

Step 1

Acetic anhydride (2.94 mL, 31.1 mmol) was added dropwise to a solution of *endo*-2-amino-2-norbornanecarboxylic acid (0.65 g, 4.19 mmol) in formic acid (>95%, 8.7 mL) at 0 °C. The resulting solution was allowed to stir at room temperature for 1 hour and quenched with ice water (6 mL). The solvent was removed *in vacuo* to give the crude *N*-formyl amino acid (0.72 g) which was used directly in the next step without further purification. ^{1}H NMR (400 MHz, $(CD_3)_2SO$) δ: 7.92 (1 H, s), 2.65 (1 H, d, J = 3.5 Hz), 2.17 – 2.10 (2 H, m), 2.07 (1 H, d, J = 10.0 Hz), 1.59 – 1.45 (2 H, m), 1.36 – 2.44 (3 H, m), 1.19 – 1.12 (1 H, m).

Step 2

To a vigorously stirred suspension of *N*-formyl amino acid (0.72 g, 3.9 mmol) and sodium borohydride (0.42 g, 11.1 mmol) in anhydrous THF (10 mL) was added a solution of iodine (1.17 g, 4.6 mmol) in anhydrous THF (5 mL) dropwise at 0 °C. Once gas evolution had ceased the mixture was heated at reflux for 18 hours and cooled to room temperature. Methanol was added cautiously until the solution became clear and the solvent was subsequently removed *in vacuo* to afford a white paste. The paste was dissolved in the minimum amount of 20% aqueous KOH (ca. 4 mL) and stirred at room temperature for 1 hour. The mixture was diluted with 20% aqueous KOH (10 mL) and extracted with CH_2Cl_2 (3 x 15 mL). The organics were subsequently dried (Na_2SO_4) and removed *in vacuo* to give the crude amino alcohol as a colorless oil (0.55 g, 91%) which was used in the next step without further purification ^{1}H NMR (400.1 MHz, $CDCl_3$) δ 3.50 (1 H, d, J = 10.2 Hz), 3.24 (1 H, d, J = 10.5 Hz), 2.22 (3 H, s), 2.22 – 2.20 (1 H, m), 2.09 (1 H, d, J = 4.5 Hz), 1.68 (1 H, ddd, J = 3.1, 4.4, 13.0 Hz), 1.64 – 1.40 (3 H, m), 1.31 – 1.12 (3 H, m), 0.76 (1 H, dd, J = 2.7, 13.2 Hz).

Step 3

To a solution of crude amino alcohol (0.55 g, 3.6 mmol) and 4-dimethylaminopyridine (22 mg, 0.18 mmol) in anhydrous CH_2Cl_2 (8 mL) was added trimethylacetyl chloride (0.52 mL, 4.3 mmol) dropwise. The reaction mixture was cooled to 0 °C and triethylamine (1.15 mL, 4.3 mmol) was added dropwise and the solution allowed to warm to room temperature over 16 h. The reaction was then diluted with CH_2Cl_2 (20 mL) and washed sequentially with water (2 x 30 mL), 0.1 M HCl (30 mL), sat. $NaHCO_3$ (30 mL) and brine (30 mL). The organic phase was dried over Na_2SO_4 and concentrated *in vacuo*. The crude oil was purified by flash column chromatography (gradient elution: 5% EtOAc in P.E. to 100% EtOAc) to provide the desired amine as colorless oil (0.19 g, 20%). IR ν_{max}/cm^{-1} (film) 2950, 2870, 2800, 1727, 1480, 1467, 1443, 1397, 1364, 1328, 1313, 1282, 1152, 1101, 1064, 1036; ^1H NMR (400 MHz, $CDCl_3$) δ: 4.12 (1 H, d, J = 11.4 Hz), 3.91 (1 H, d, J = 11.4 Hz), 2.25 (3 H, s), 2.20 (1 H, app. t, J = 3.9 Hz), 2.10 (1 H, app. d, J = 2.6 Hz), 1.92 – 1.85 (1 H, m), 1.58 – 1.50 (2 H, m), 1.45 (1 H, ddd, J = 2.9, 4.4, 12.4 Hz), 1.37 – 1.24 (3 H, m), 1.21 (9 H, s), 0.93 (1 H, dd, J = 2.9, 12.6 Hz). ^{13}C NMR (101 MHz, $CDCl_3$) δ: 178.7, 65.3, 62.7, 42.1, 41.9, 39.1, 38.2, 36.9, 30.4, 29.0, 27.4, 22.8. m/z HRMS found $[M + H]^+$ 240.1959, $C_{14}H_{26}NO_2$ requires 240.1958.

***N*-(2-(1-tosyl-1H-indol-3-yl)ethyl)-1-(2-((triisopropylsilyl)oxy)ethyl)cyclobutan-1-amine (1x)**

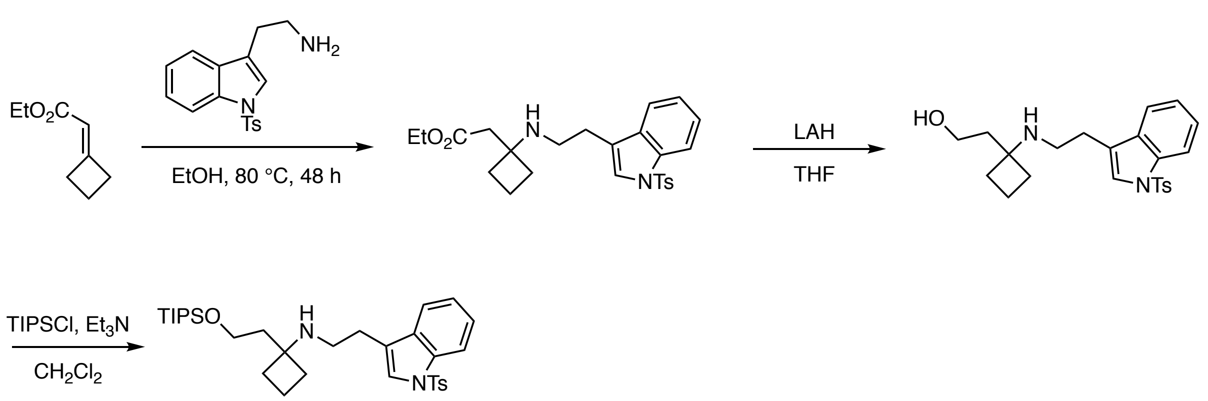

Step 1

2-(1-tosyl-1H-indol-3-yl)ethan-1-amine[8] (4.21 g, 13.40 mmol) and ethyl 2-cyclobutylideneacetate[4] (0.94 g, 6.70 mmol) were combined in EtOH (5 mL) and heated at 80 °C in a sealed tube for 2 days. The reaction mixture was concentrated *in vacuo* and the residue purified by flash column chromatography (50% EtOAc in P.E.) to afford the title compound as a viscous yellow oil (3.00 g, 98%). IR ν_{max}/cm^{-1} (film): 2979, 2936, 1726, 1446, 1367, 1274, 1241, 1170, 1118, 1095, 1019; ^1H NMR (400 MHz, $CDCl_3$) δ: 7.97 (1 H, d, J = 8.3 Hz), 7.75 (2 H, d, J = 8.3 Hz), 7.49 (1 H, d, J = 7.7 Hz), 7.40 (1 H, s), 7.29 (1 H, t, J = 7.7 Hz), 7.24 – 7.16 (3 H, m), 4.04 (2 H, q, J = 7.1 Hz), 2.83 (4 H, s), 2.61 (2 H, s), 2.31 (3 H, s), 1.98 – 1.69 (6 H, m), 1.17 (3 H, t, J = 7.1 Hz); ^{13}C NMR (101 MHz,

[8] Logers, M.; Overman, L. E.; Welmaker, G. S.; *J. Am. Chem. Soc.*, **1995**, 9139.

$CDCl_3$) δ: 171.8, 144.8, 135.4, 135.3, 131.1, 129.9, 126.9, 124.7, 123.2, 123.1, 121.1, 119.6, 113.8, 60.2, 58.3, 41.6, 41.4, 32.3, 26.4, 21.6, 14.3, 13.8; m/z HRMS found $[M + H]^+$ 455.1988, $C_{25}H_{31}N_2O_4S$ requires 455.1999.

Step 2

Ethyl 2-(1-((2-(1-tosyl-1H-indol-3-yl)ethyl)amino)cyclobutyl)acetate (2.90 g, 6.40 mmol) was dissolved in THF (30 mL) and the solution cooled to 0 °C. Lithium aluminium hydride (0.968 g, 25.00 mmol) was added portion-wise and the solution allowed to warm to room temperature and stirred for 30 minutes. Water (1 mL) was added to the reaction carefully at 0 °C followed by the sequential addition of 10% aqueous NaOH solution (1 mL) and water (3 mL). $MgSO_4$ was added to the reaction mixture and the solution filtered through celite, eluting with Et_2O, and concentrated *in vacuo* to afford the title compound as a white solid (2.04 g, 77%). ^1H NMR (400 MHz, $CDCl_3$) δ: 8.00 (1 H, d, *J* = 8.3 Hz), 7.76 (2 H, d, *J* = 8.4 Hz), 7.49 (1 H, d, *J* = 7.8 Hz), 7.37 (1 H, s), 7.40 – 7.29 (1 H, m), 7.25 – 7.17 (3 H, m), 3.86 – 3.77 (2 H, m), 2.90 – 2.84 (2 H, m), 2.83 – 2.77 (2 H, m), 2.33 (3 H, s), 1.97 – 1.57 (8 H, m); ^{13}C NMR (101 MHz, $CDCl_3$) δ: 145.0, 135.5, 135.4, 130.8, 130.0, 126.9, 125.0, 123.4, 123.3, 122.2, 119.5, 113.9, 60.7, 60.4, 41.0, 34.7, 33.0, 25.8, 21.7, 13.5.

Step 3

2-(1-((2-(1-tosyl-1H-indol-3-yl)ethyl)amino)cyclobutyl)ethan-1-ol (1.90 g, 4.60 mmol) was dissolved in CH_2Cl_2 (15 mL) and cooled to 0 °C. Triethylamine (0.96 mL, 6.90 mmol) was added followed by drop-wise addition of triisopropylsilyl chloride (0.94 mL, 4.37 mmol) and the solution was allowed to warm to room temperature overnight. The mixture was transferred to a separating funnel and diluted with CH_2Cl_2. The organic layer was washed sequentially with water (3 x 15 mL) then brine (15 mL), dried over $MgSO_4$ and concentrated *in vacuo*. The residue was purified by flash column chromatography (gradient elution: CH_2Cl_2 to 15% MeOH in CH_2Cl_2) to afford the title compound as a light yellow solid (1.18 g, 45%). m. p.: 68–70 °C; IR ν_{max}/cm^{-1} (film): 2928, 2863, 1461, 1444, 1363, 1307, 1275, 1171, 1128, 1116, 1095; ^1H NMR (400 MHz, $CDCl_3$) δ: 7.99 (1 H, d, *J* = 8.3 Hz), 7.74 (2 H, app d, *J* = 8.4 Hz), 7.49 (1 H, d, *J* = 7.8 Hz), 7.37 (1 H, s), 7.31 (1 H, td, *J* = 8.3, 1.1 Hz), 7.24 – 7.18 (3 H, m), 3.75 (2 H, t, *J* = 7.0 Hz), 2.81 (4 H, s), 2.33 (3 H, s), 1.88 (4 H, t, *J* = 7.0 Hz), 1.85 – 1.76 (2 H, m), 1.74 – 1.63 (2 H, m), 1.08 – 1.02 (21 H, m); ^{13}C NMR (101 MHz, $CDCl_3$) δ: 144.9, 135.5, 135.5, 131.1, 129.9, 126.9, 124.8, 123.3, 123.2, 121.2, 119.6, 113.9, 60.2, 58.9, 41.8, 33.0, 26.5, 21.7, 18.2, 17.9, 13.8, 12.1.; m/z HRMS found $[M + H]^+$ 569.3216, $C_{32}H_{49}N_2O_3SSi$ requires 569.3228.

***N*-methyl-1-tosyl-3-(2-((triisopropylsilyl)oxy)ethyl)piperidin-3-amine (1y)**

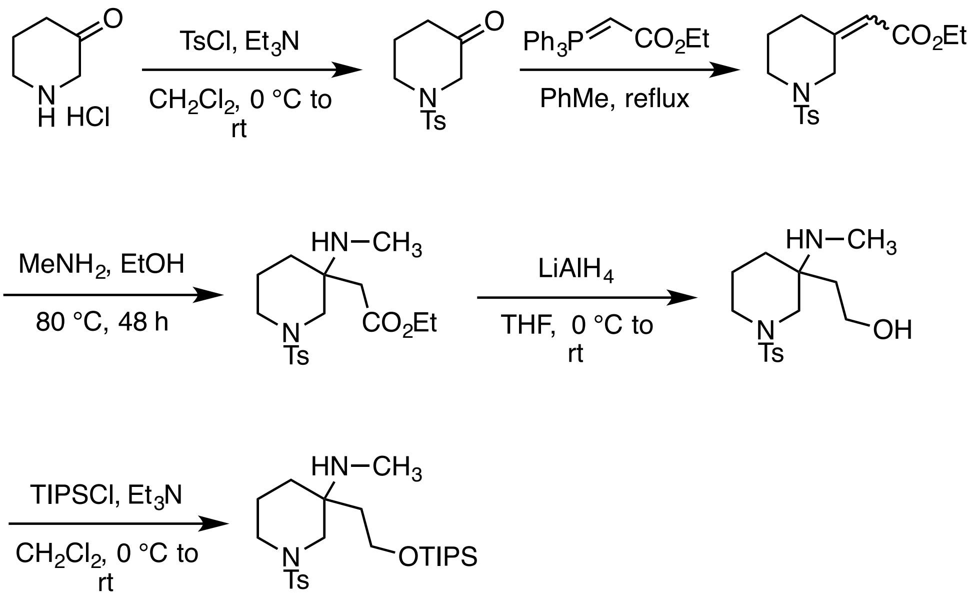

Step 1

Tosyl chloride (1.30 g, 6.8 mmol) was added portionwise to a solution of 3-piperidone hydrochloride hydrate (1.00 g, 6.5 mmol) and triethylamine (2.7 mL, 19.5 mmol) in dichloromethane (25 mL) at 0 ºC. The solution was allowed to warm to room temperature over 16 hours and was quenched with H_2O (20 mL). The organic layer was separated, and the aqueous extracted with additional dichloromethane (2 x 15 mL). The organics were combined and washed with 1M HCl (50 mL), brine (50 mL), dried over K_2CO_3 and removed *in vacuo* to afford the crude 1-tosyl-3-piperidone as a pale yellow solid which was used without further purification (1.37 g, 84%). ^{1}H NMR (400 MHz, $CDCl_3$) δ: 7.64 (2 H, d, *J* = 8.2 Hz), 7.33 (2 H, d, *J* = 8.2 Hz), 3.58 (2 H, s), 3.27 (2 H, t, *J* = 6.0 Hz), 2.42 (3 H, s), 2.34 (2 H, t, *J* = 6.9 Hz), 2.02 – 1.96 (2 H, m).

Step 2

A solution of 1-tosyl-3-piperidone (2.30 g, 9.1 mmol) and ethyl (triphenylphosphoranylidene)acetate (3.48 g, 10 mmol) was refluxed in toluene (20 mL) for 24 hours. The solution was subsequently cooled to room temperature and the solvent removed *in vacuo*. The resulting product was dissolved in 2:1 EtOAc/P.E. (50 mL), filtered over a bed of silica and celite, and washed with additional 2:1 EtOAc/P.E. (100 mL). The solvent was removed *in vacuo* and the resulting oil purified by flash column chromatography (gradient elution: 5% EtOAc in P.E. to 30% EtOAc in P.E.) to provide the desired product (1.97 g, 67%) as an inseparable mixture of diastereoisomers with *E*-form and *Z*-form (1:1). ^{1}H NMR (400 MHz, $CDCl_3$) δ: 7.69 – 7.63 (4 H, m), 7.33 – 7.29 (4 H, m), 5.77 (1 H, s), 5.68 (1 H, s), 4.28 (2 H, s), 4.21 – 4.11 (4 H, m), 3.54 (2 H, s), 3.20 (2 H, t, *J* = 5.0 Hz), 3.13 (2 H, t, *J* =

4.90), 2.79 (2 H, t, J = 5.6 Hz), 2.43 (3 H, s), 2.42 (3 H, s), 2.21 (2 H, t, J = 5.6 Hz), 1.78 – 1.70 (4 H, m), 1.31 – 1.25 (6 H, m).

Step 3

A solution of ethyl 2-(1-tosylpiperidin-3-ylidene)acetate (1.97 g, 6.1 mmol) in ethanol (2 mL) and methylamine (33% w/w in EtOH, 1.5 mL, 12.2 mmol) were stirred at 80 ºC in a sealed tube for 48 hours. After such time the solution was cooled to room temperature and the solvent removed *in vacuo*. The crude oil was purified by flash column chromatography (gradient elution: 1% MeOH in CH_2Cl_2 to 5% MeOH in CH_2Cl_2) to provide the desired amine as colorless oil (1.52 g, 70%). ^{1}H NMR (400 MHz, $CDCl_3$) δ: 7.60 (2 H, d, J = 8.4 Hz), 7.29 (2 H, d, J = 8.1 Hz), 4.11 (2 H, q, J = 7.1 Hz), 2.94 – 2.87 (4 H, m), 2.49 (2 H, s), 2.40 (3 H, s), 2.29 (3 H, s), 1.79 (1 H, br s, (N-H)), 1.72 – 1.62 (2 H, m), 1.52 – 1.41 (2 H, m), 1.24 (3 H, t, J = 7.1 Hz).

Step 4

To a solution of ethyl 2-(3-(methylamino)-1-tosylpiperidin-3-yl)acetate (1.52 g, 4.3 mmol) in anhydrous THF (30 mL) was added $LiAlH_4$ (0.65 g, 17.1 mmol) portionwise at 0 ºC. The resulting solution was allowed to warm to room temperature over 6 hours and quenched successively with H_2O (0.75 mL), 10% NaOH (1 mL) and H_2O (2 mL). The resulting slurry was stirred with $MgSO_4$ and filtered. The filter cake was washed with Et_2O (100 mL) and the combined organics removed *in vacuo* to afford the crude amino alcohol (1.23 g, 91%) as a viscous oil which solidified on standing and was used without further purification. ^{1}H NMR (400 MHz, $CDCl_3$) δ: 7.63 (2 H, d, J = 8.3 Hz), 7.33 (2 H, d, J = 7.8 Hz), 3.80 – 3.74 (2 H, m), 3.56 – 3.51 (2 H, m), 2.44 (3 H, s), 2.35 (3 H, s), 2.19 (1 H, d, J = 11.2 Hz), 1.86 – 1.48 (6 H, m), 1.22 – 1.13 (1 H, m).

Step 5

Triisopropylsilyl chloride (0.80 mL, 3.7 mmol) was added dropwise to a solution of amino alcohol (1.23 g, 3.9 mmol) and triethylamine (0.82 mL, 5.8 mmol) in anhydrous dichloromethane (15 mL) at 0 ºC. The resulting solution was allowed to warm to room temperature over 16 hours and was quenched with H_2O (20 mL). The organics were separated and washed with additional H_2O (2 x 20 mL) and brine (20 mL), dried over Na_2SO_4 and removed *in vacuo*. The crude oil was purified by flash column chromatography (gradient elution: 50% EtOAc in P.E. to 100% EtOAc) to provide the desired amine as a white crystalline solid (1.49 g, 82%). m.p. 76–77 ºC. IR ν_{max}/cm^{-1} (film): 3370, 2938, 2865, 2790, 1598, 1465, 1389, 1353, 1336, 1307, 1257, 1162, 1145, 1091, 1064. ^{1}H NMR (400 MHz, $CDCl_3$) δ: 7.61 (2 H, d, J = 8.6 Hz), 7.30 (2 H, d, J = 8.2 Hz), 3.81 (2 H, t, J = 6.7 Hz), 3.27 – 3.24 (1 H, m), 3.19 (1 H, d, J = 11.5 Hz), 2.58 – 2.53 (1 H, m), 2.50 (1 H, d, J = 11.5 Hz), 2.42 (3 H, s), 2.26 (3 H, s), 1.74 (1 H, br s), 1.71 – 1.55 (5 H, m), 1.32 – 1.25 (1 H, m), 1.06 – 1.02 (21 H, m). ^{13}C NMR

(101 MHz, $CDCl_3$) δ: 143.5, 133.3, 129.7, 127.8, 59.2, 53.8, 52.9, 46.8, 37.1, 32.2, 27.9, 21.6, 21.2, 18.2, 12.0. m/z HRMS found $[M + H]^+$ 469.2904, $C_{24}H_{45}O_3N_2SSi$ requires 469.2915.

(2-(methylamino)-1,2,3,4-tetrahydronaphthalen-2-yl)methyl pivalate (1z)

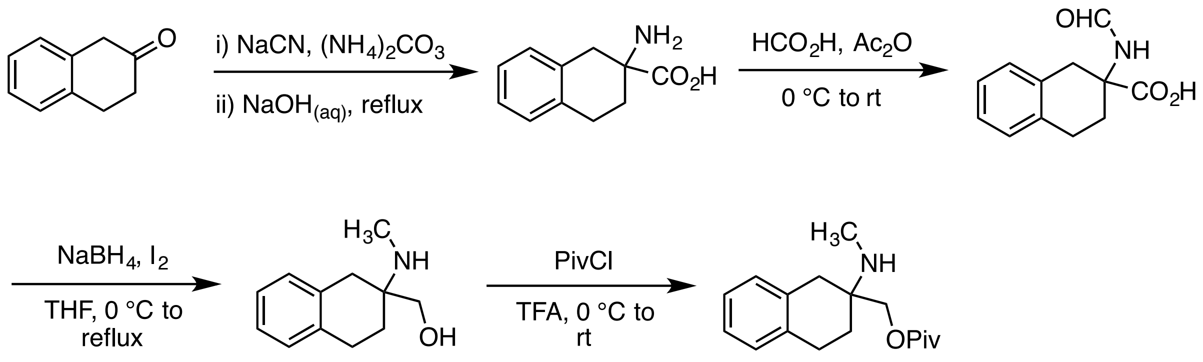

Step 1

β-tetralone (3.5 g, 23.9 mmol), sodium cyanide (2.13 g, 26.3 mmol), and ammonium carbonate (5.3 g, 114.7 mmol) were suspended in ethanol/water (1:1, 80 mL) and heated at 60 °C for 16 hours. The solution was cooled to room temperature and the resulting precipitate was collected by vacuum filtration, washed with water (50 mL) and dried under vacuum to afford the desired product as a white solid (4.5 g, 87%) which was used directly in the next step without further purification. ^{1}H NMR (400 MHz, $(CD_3)_2SO$) δ: 10.05 (1 H, br s), 8.24 (1 H, s), 7.32 – 6.74 (4 H, m), 3.11 (1 H, d, J = 17.0 Hz), 2.95 – 2.83 (2 H, m), 2.76 (1 H, d, J = 17.0 Hz), 1.97 – 1.89 (1 H, m), 1.87 – 1.76 (1 H, m).

Step 2

Crude hydantoin (4.5 g, 20.8 mmol) was suspended in aqueous sodium hydroxide (5 M, 25 mL) and heated at 120 °C for 24 hours. The solution was cooled to room temperature and the solid removed by filtration. The mother liquor was acidified to pH = 6 and the solvent removed *in vacuo.* The residue was suspended in ethanol (100 mL) and heated to reflux for 10 minutes. The solvent was rapidly filtered and the mother liquor cooled to room temperature and removed *in vacuo* to afford the crude amino acid as a pale pink solid (1.15 g, 25%). ^{1}H NMR (400 MHz, D_2O) δ: 7.30 – 7.18 (4 H, m), 3.23 (1 H, d, J = 16.7 Hz), 2.98 (1 H, d, J = 16.7 Hz), 2.89 – 2.76 (2 H, m), 2.42 – 2.31 (1 H, m), 2.05 – 1.99 (1 H, m).

Step 3

Acetic anhydride (4 mL) was added dropwise to a solution of crude amino acid (0.83 g, 5.2 mmol) in formic acid (> 95%, 10 mL) at room temperature. After the addition was complete the reaction mixture was stirred for an additional hour and quenched by the addition of ice water (10 mL). The solvent was removed *in vacuo* to give the crude *N*-formyl amino acid which was used directly in the

next step without further purification (0.76 g, 80%). ^{1}H NMR (400 MHz, $(CD_3)_2SO$) δ: 7.89 (1 H, s), 7.09 – 7.00 (4 H, m), 3.17 (1 H, d, *J* = 16.8 Hz), 3.04 (1 H, d, *J* = 16.8 Hz), 2.80 – 2.71 (2 H, m), 2.35 – 2.27 (1 H, m), 2.00 – 1.89 (1 H, m).

Step 4

To a vigorously stirred suspension of *N*-formyl amino acid (0.76 g, 3.5 mmol) and sodium borohydride (0.37 g, 9.7 mmol) in anhydrous THF (10 mL) was added a solution of iodine (1.06 g, 4.2 mmol) in anhydrous THF (5 mL) dropwise at 0 °C. Once gas evolution had ceased the mixture was heated at reflux for 18 hours and subsequently cooled to room temperature. Methanol was added cautiously until the solution became clear and the solvent was subsequently removed *in vacuo* to afford a white paste. The paste was dissolved in the minimum amount of 20% aqueous KOH (ca. 5 mL) and stirred at room temperature for 1 hour. The mixture was diluted with 20% aqueous KOH (10 mL) and extracted with CH_2Cl_2 (3 x 20 mL). The organics were combined, dried over $MgSO_4$ and removed *in vacuo* to give the crude amino alcohol as a brown oil (0.40 g, 59%) which was used in the next step without further purification. ^{1}H NMR (400 MHz, $CDCl_3$) δ: 7.14 – 7.16 (4 H, m), 3.52 – 3.33 (2 H, m), 2.86 – 2.67 (2 H, m), 2.33 (3 H, s), 1.91 – 1.82 (1 H, m), 1.79 – 1.69 (1 H, m), 1.62 – 1.52 (1 H, m), 1.44 – 1.33 (1 H, m).

Step 5

A 10 mL round-bottomed flask was charged with crude amino alcohol (0.40 g, 2.0 mmol) and cooled in an ice bath. Trifluoroacetic acid (1 mL) was added dropwise with vigorous stirring to afford a viscous solution. The reaction mixture was stirred at 0 °C for 5 minutes and trimethylacetyl chloride (0.37 mL, 3.0 mmol) was added dropwise. The flask was sealed with a glass-stopper and warmed to room temperature over 16 hours. The solvent was removed *in vacuo,* the residue was dissolved in diethyl ether (10 mL) and treated with triethylamine until pH = 9. Water (10 mL) was added and the layers were separated and the aqueous was extracted with diethyl ether (3 x 10 mL). The organics were combined, dried over $MgSO_4$ and removed *in vacuo*. The crude product was purified by flash column chromatography (30% EtOAc in P.E.) to afford the product as a white solid (0.26 g, 41%). m.p. 51–53 °C; IR ν_{max}/cm^{-1} (film): 3348, 2958, 2935, 2911, 1712, 1496, 1478, 1465, 1454, 1425, 1396, 1368, 1285, 1172, 1154, 1142, 1076, 1038. ^{1}H NMR (400 MHz, $CDCl_3$) δ: 7.11 – 7.07 (4 H, m), 4.05 (2 H, dd, *J* = 11.1, 18.1 Hz), 2.95 – 2.71 (4 H, m), 2.37 (3 H, s), 1.91 – 1.75 (2 H, m), 1.24 (9 H, s), 1.16 (1 H, s). ^{13}C NMR (101 MHz, $CDCl_3$) δ: 178.3, 135.6, 134.1, 129.6, 128.8 126.0, 126.0, 66.4, 53.8, 39.1, 37.3, 28.7, 28.3, 27.3, 25.6. m/z HRMS found $[M + H]^+$ 276.1957, $C_{17}H_{26}O_2N$ requires 276.1958

(1-(benzylamino)cyclohexyl)methyl pivalate (1aa)

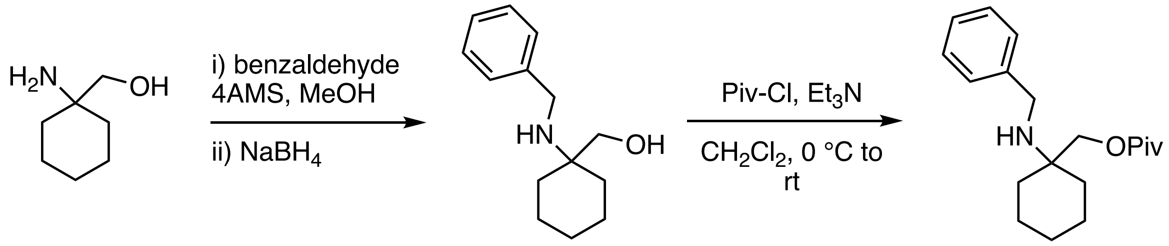

Step 1

To a solution of (1-aminocyclohexyl)methanol (1.00 g, 7.70 mmol) in MeOH (10 mL) was added powdered 4Å molecular sieves (~1 g) and benzaldehyde (1.17 mL, 11.6 mmol) and the mixture stirred at ambient temperature for 16 h. The mixture was cooled to 0 °C, sodium borohydride (0.43 g, 11.6 mmol) was added portion-wise and the mixture allowed to warm to ambient temperature and stirred for an additional 2 h. The reaction mixture was filtered and the MeOH removed *in vacuo*. 10% aqueous NaOH solution was added and the mixture was extracted with EtOAc (3 x 10 mL). The combined organic phases were dried ($MgSO_4$) and concentrated *in vacuo* to afford the crude product as a colourless oil (1.46 g, 86%) which was used in the following step without further purification. ^{1}H NMR (400 MHz, $CDCl_3$) δ: 7.39 – 7.29 (5 H, m), 3.64 (2 H, s), 3.39 (2 H, s), 1.69 – 1.59 (2 H, m), 1.58 – 1.34 (8 H, m).

Step 2

(1-(benzylamino)cyclohexyl)methanol (1.46 g, 6.66 mmol) was dissolved in CH_2Cl_2 (15 mL) and cooled to 0 °C. Triethylamine (1.86 mL, 13.3 mmol) was added followed by drop-wise addition of trimethylacetyl chloride (0.984 mL, 7.99 mmol). The reaction mixture was allowed to warm slowly to ambient temperature and stirred for 16 h after which it was quenched with water (7 mL) and extracted with CH_2Cl_2 (2 x 15 mL). The combined organic phases were washed with brine, dried ($MgSO_4$) and concentrated *in vacuo*. The resulting residue was purified by flash column chromatography (5% EtOAc in P.E.) to afford the title compound as a colourless oil (1.40 g, 70%). IR ν_{max}/cm^{-1} (film): 2925, 2845, 1717, 1481, 1465, 1453, 1393, 1362, 1286, 1165, 1086, 1066, 1035; ^{1}H NMR (400 MHz, $CDCl_3$) δ: 7.39 – 7.28 (4 H, m), 7.27 – 7.21 (1 H, m), 4.04 (2 H, s), 3.65 (2 H, s), 1.75 – 1.54 (5 H, m), 1.48 – 1.29 (5 H, m), 1.22 (9 H, s); ^{13}C NMR (101 MHz, $CDCl_3$) δ: 178.5, 141.5, 128.5, 128.4, 127.0, 68.1, 54.2, 45.9, 39.1, 32.9, 27.4, 26.3, 21.4; m/z HRMS found $[M + H]^+$ 304.2269, $C_{19}H_{30}NO_2$ requires 304.2271.

(1-((4-methylbenzyl)amino)cyclohexyl)methyl pivalate (1ab)

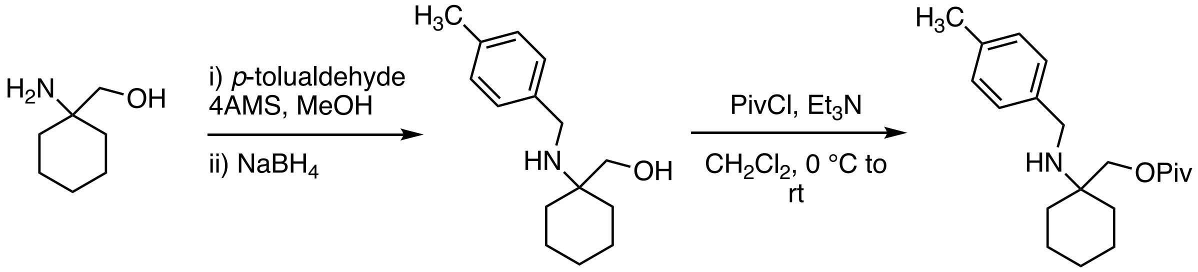

Step 1

To a solution of (1-aminocyclohexyl)methanol (1.0 g, 7.7 mmol) and powdered 4A molecular sieves (1 g) in anhydrous methanol (10 mL) was added *p*-tolualdehyde (1.36 mL, 11.6 mmol). The resulting mixture was stirred at room temperature for 16 hours and cooled to 0 ºC. Sodium borohydride (0.44 g, 11.6 mmol) was cautiously added portionwise and warmed to room temperature over 2 hours. The crude reaction mixture was filtered over celite, washed with methanol and the solvent removed *in vacuo*. The resulting white paste was dissolved in 10% aqueous NaOH (30 mL) and stirred at room temperature for 2 hours. The aqueous was extracted with CH_2Cl_2 (3 x 30 mL) and concentrated *in vacuo*. The resulting product was dissolved in 3M HCl (30 mL) and water (30 mL) and washed with diethyl ether (3 x 30 mL) and discarded. The resulting aqueous layer was cooled to 0 ºC and basified carefully to pH = 10 with sodium hydroxide pellets. The solution was extracted with dichloromethane (3 x 30 mL), dried over Na_2SO_4 and removed *in vacuo* to afford the crude amino alcohol as a white solid (1.43 g, 79%), which was used without further purification. ^{1}H NMR (400 MHz, $CDCl_3$) δ: 7.23 (2 H, d, J = 7.8 Hz), 7.14 (2 H, d, J = 7.8 Hz), 3.59 (2 H, s), 3.37 (2 H, s), 2.34 (3 H, s), 1.65 – 1.37 (10 H, m).

Step 2

To a solution of crude amino alcohol (0.90 g, 3.9 mmol) in anhydrous dichloromethane (20 mL) was added triethylamine (1.19 mL, 8.6 mmol) and trimethylacetyl chloride (0.52 mL, 4.3 mmol) dropwise at 0 °C. The reaction mixture was warmed to room temperature over 16 hours and quenched by the addition of saturated aqueous $NaHCO_3$ (20 mL). The organic layer was separated and washed with additional saturated aqueous $NaHCO_3$ (2 x 20 mL), brine (20 mL) and dried over $MgSO_4$. The solvent was removed *in vacuo* and the crude oil purified by flash column chromatography (gradient elution: 100% P.E. to 10% EtOAc in P.E.) to afford the product as a white solid (0.90 g, 73%). m.p. 42–44 ºC. IR ν_{max}/cm^{-1} (film): 3321, 2923, 2848, 1712, 1514, 1481, 1463, 1441, 1398, 1360, 1286, 1185, 1166, 1088. ^{1}H NMR (400 MHz, $CDCl_3$) δ: 7.24 (2 H, d, J = 7.7 Hz), 7.12 (2 H, d, J = 7.7 Hz), 4.03 (2 H, s), 3.60 (2 H, s), 2.33 (3 H, s), 1.73 – 1.56 (5 H, m), 1.47 – 1.30 (5 H, m), 1.22 (9 H, s). ^{13}C NMR (101 MHz, $CDCl_3$) δ: 178.6, 138.5, 136.5, 129.2, 128.4, 68.1, 54.2, 45.6, 39.1, 32.9, 27.4, 26.3, 21.4,

21.2. m/z HRMS found $[M + H]^+$ 318.2429, $C_{20}H_{32}NO_2$ requires 344.2428.

Ethyl 1-aminocyclohexanecarboxylate hydrochloride

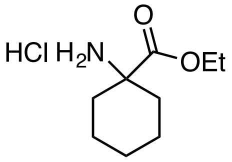

Thionyl chloride (30 mL) was added dropwise to a suspension of 1-aminocyclohexane-1-carboxylic acid hydrochloride (8.95 g, 50 mmol) in absolute ethanol (200 mL) at 0 ºC. The resulting mixture was heated to reflux for 16 hours and subsequently cooled to room temperature. The solvent was removed *in vacuo* and residue dried under hi-vac (>1 mbar, 75 ºC) to afford the product as a free-flowing white powder (10.2 g, 99%). m.p. 182 ºC (sharp). IR ν_{max}/cm^{-1} (film): 2938, 2897, 2853, 1743, 1597, 1569, 1519, 1471, 1452, 1391, 1367, 1293, 1244, 1154, 1107, 1042, 1023. ^1H NMR (400 MHz, D_2O) δ: 4.32 (2 H, q, J = 7.1 Hz), 2.17 – 2.10 (2 H, m), 1.85 – 1.73 (4 H, m), 1.60 – 1.50 (4 H, m), 1.32 (3 H, t, J = 7.1 Hz). ^{13}C NMR (101 MHz, D_2O) δ: 172.2, 63.6, 59.9, 31.1, 23.5, 20.2, 13.1. m/z HRMS found $[M + H]^+$ 172.1328, $C_9H_{18}NO_2$ requires 172.1332.

(1-((4-chlorobenzyl)amino)cyclohexyl)methyl pivalate (1ac)

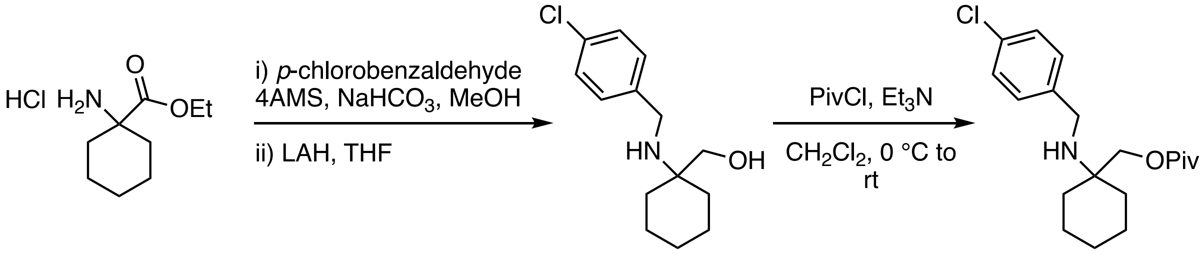

Step 1

To a solution of ethyl 1-aminocyclohexanecarboxylate hydrochloride (1.03 g, 5.0 mmol), sodium bicarbonate (0.42 g, 5.5 mmol) and powder 4A molecular sieves (1.0 g) in anhydrous methanol (10 mL) as added *p*-chlorobenzaldehyde (0.70 g, 5.0 mmol). The reaction was allowed to stir at room temperature for 16 hours and was subsequently filtered over celite and washed with methanol. The solvent was removed *in vacuo* and dried under vacuum. The resulting residue was dissolved in anhydrous THF (25 mL) and cooled to 0 °C before LAH (0.57 g, 15.0 mmol) was added portion-wise. The reaction mixture was warmed to room temperature over 2 hours, cooled to to 0 °C and quenched successively with H_2O (0.75 mL), 10% NaOH (1 mL) and H_2O (2 mL). The resulting slurry was stirred with $MgSO_4$ and filtered. The filter cake was washed with Et_2O (100 mL) and the combined organics removed *in vacuo* to afford the crude amino alcohol as a white solid (1.23 g, 97%), which

was used without further purification. ^{1}H NMR (400 MHz, $CDCl_3$) δ: 7.33 – 7.26 (5 H, m), 3.60 (2 H, s), 3.38 (2 H, s), 1.61 – 1.40 (10 H, m).

Step 2

To a solution of crude amino alcohol (1.01 g, 4.0 mmol) in anhydrous dichloromethane (20 mL) was added triethylamine (1.22 mL, 8.8 mmol) and trimethylacetyl chloride (0.53 mL, 4.4 mmol) dropwise at 0 °C. The reaction mixture was warmed to room temperature over 16 hours and quenched by the addition of saturated aqueous $NaHCO_3$ (20 mL). The organic layer was separated and washed with additional saturated aqueous $NaHCO_3$ (2 x 20 mL), brine (20 mL) and dried over $MgSO_4$. The solvent was removed *in vacuo* and the crude oil purified by flash column chromatography (gradient elution: 100% P.E. to 10% EtOAc in P.E.) to afford the product as a colourless oil (0.45 g, 33%). IR ν_{max}/cm^{-1} (film): 2933, 2855, 1727, 1490, 1479, 1461, 1397, 1364, 1282, 1150, 1091, 1034, 1015. ^{1}H NMR (400 MHz, $CDCl_3$) δ: 7.28 – 7.26 (5 H, m), 4.02 (2 H, s), 3.61 (2 H, s), 1.70 – 1.57 (5 H, m), 1.46 – 1.31 (5 H, m), 1.21 (9 H, s). ^{13}C NMR (101 MHz, $CDCl_3$) δ: 178.5, 140.1, 132.6, 129.7, 128.6, 68.1, 54.3, 45.2, 39.1, 32.8, 27.4, 26.2, 21.4. m/z HRMS found $[M + H]^+$ 338.1884, $C_{19}H_{29}ClNO_2$ requires 338.1881.

(1-((3-nitrobenzyl)amino)cyclohexyl)methyl pivalate (1ad)

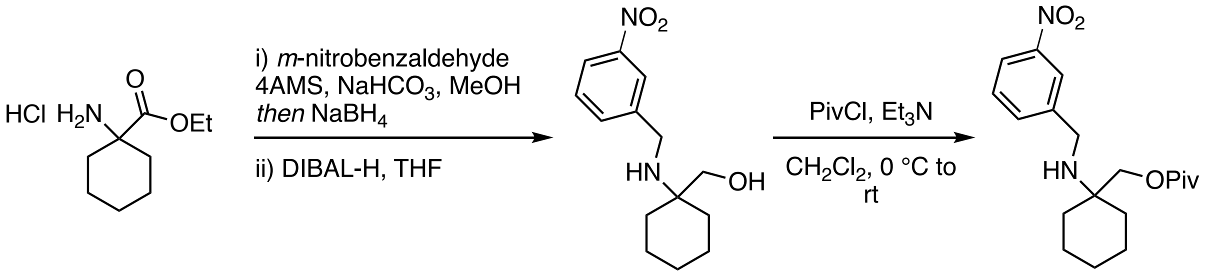

Step 1

To a solution of ethyl 1-aminocyclohexanecarboxylate hydrochloride (1.03 g, 5.0 mmol), sodium bicarbonate (0.42 g, 5.5 mmol) and powder 4A molecular sieves (1.0 g) in anhydrous methanol (10 mL) as added *m*-nitrobenzaldehyde (0.76 g, 5.0 mmol). The reaction was allowed to stir at room temperature for 16 hours and was subsequently cooled to 0 °C. Sodium borohydride (0.28 g, 7.5 mmol) was cautiously added portion-wise and the mixture was allowed to warm to room temperature over 2 hours. The reaction mixture was filtered over celite, washed with methanol and the solvent removed *in vacuo* to afford the amino ester as a yellow oil (1.45 g, 95%), which was used without further purification. ^{1}H NMR (400 MHz, $CDCl_3$) δ: 8.23 (1 H, app s), 8.09 (1 H, dd, J = 1.6, 8.2 Hz), 7.68 (1 H, d, J = 7.5 Hz), 7.47 (1 H, t, J = 8.0 Hz), 4.20 (2 H, q, J = 7.1 Hz), 3.70 (2 H, s), 1.95 – 1.89 (2 H, m), 1.75 – 1.37 (8 H, m), 1.30 (3 H, t, J = 7.1 Hz).

Step 2

To a solution of the crude amino ester (1.38 g, 4.5 mmol) in anhydrous THF (20 mL) at 0 °C was added DIBAL-H (1 M in THF, 13.5 mL, 13.5 mmol) dropwise. The resulting solution was allowed to warm to room temperature over 16 hours. The solution was cooled to 0 °C and quenched with Rochelle's salt (20 mL). The mixture was allowed to stir at room temperature for 3 hours and the organic layer was separated. The aqueous was extracted with ethyl acetate (3 x 20 mL) and the combined organic layers washed with brine, dried over Na_2SO_4 and concentrated *in vacuo*. The resulting solid was purified by flash column chromatography (gradient elution: 5% EtAOc in P.E. to 50% EtOAc in P.E.) to afford the amino alcohol as a pale yellow solid (0.58 g, 49%). ^{1}H NMR (400 MHz, $CDCl_3$) δ: 8.22 (1 H, app s), 8.11 (1 H, dd, *J* = 1.3, 8.2 Hz), 7.70 (1 H, d, *J* = 7.6 Hz), 7.50 (1 H, t, *J* = 7.9 Hz), 3.76 (2 H, s), 3.42 (2 H, s), 1.97 (1 H, br s), 1.63 – 1.41 (10 H, m).

Step 3

To a solution of crude amino alcohol (0.58 g, 2.2 mmol) in anhydrous dichloromethane (10 mL) was added triethylamine (0.67 mL, 4.8 mmol) and trimethylacetyl chloride (0.30 mL, 2.4 mmol) dropwise at 0 °C. The reaction mixture was warmed to room temperature over 16 hours and quenched by the addition of saturated aqueous $NaHCO_3$ (10 mL). The organic layer was separated and washed with additional saturated aqueous $NaHCO_3$ (2 x 10 mL), brine (10 mL) and dried over $MgSO_4$. The solvent was removed *in vacuo* and the crude oil purified by flash column chromatography (gradient elution: 100% P.E. to 25% EtOAc in P.E.) to afford the product as a colourless oil (0.47 g, 61%). IR ν_{max}/cm^{-1} (film): 2932, 2856, 1725, 1527, 1479, 1461, 1397, 1348, 1282, 1151, 1094, 1034. ^{1}H NMR (400 MHz, $CDCl_3$) δ: 8.25 (1 H, app s), 8.09 (1 H, dd, *J* = 1.0, 8.0 Hz), 7.70 (1 H, d, *J* = 7.8 Hz), 7.45 (1 H, t, *J* = 7.8 Hz), 4.04 (2 H, s), 3.77 (2 H, s), 1.67 – 1.56 (5 H, m), 1.50 – 1.38 (5 H, m), 1.22 (9 H, s). ^{13}C NMR (101 MHz, $CDCl_3$) δ: 178.5, 148.5, 143.5, 134.5, 129.3, 123.2, 122.1, 68.0, 54.5, 45.2, 39.2, 32.8, 27.4, 26.2, 21.4. m/z HRMS found $[M + H]^+$ 349.2122, $C_{19}H_{29}N_2O_4$ requires 349.2122

General procedure A for the synthesis of β–Lactams

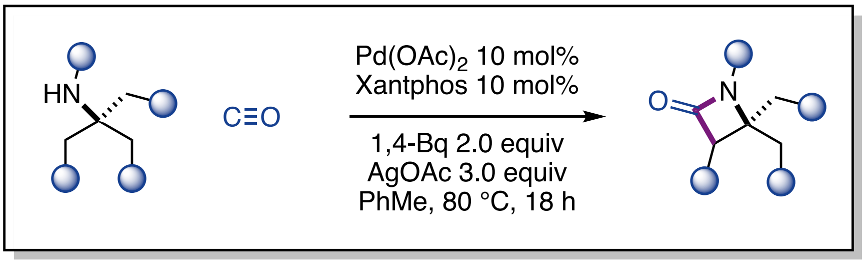

To a 10 mL oven-dried round-bottomed flask with large oval stirrer bar was added palladium (II) acetate (6.7 mg, 0.03 mmol, 0.1 equiv), silver(I) acetate (150 mg, 0.9 mmol, 3 equiv), Xantphos (17 mg, 0.03 mmol, 0.1 equiv) and 1,4-benzoquinone (66 mg, 0.6 mmol, 2 equiv). Anhydrous toluene (3 mL) and amine (0.3 mmol) were then added successively and the flask sealed with a new septum and Teflon tape. A balloon of carbon monoxide was placed on top and the flask purged (3 cycles). The flask was then placed into a preheated oil bath at 80 °C so as the solvent and oil level matched and left to stir at 500 rpm for 18 hours. After such time, the reaction mixture was allowed to cool to room temperature and the contents diluted with EtOAc (2 mL). The mixture was filtered over celite and washed with additional EtOAc (5 mL). The organics were removed under vacuum and the crude reaction mixture purified by flash column chromatography to afford the corresponding β-lactam.

cis-(7-ethyl-8-oxo-7-azabicyclo[4.2.0]octan-6-yl)methyl pivalate (2a)

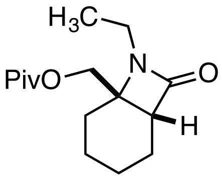

General procedure A was applied to (1-(ethylamino)cyclohexyl)methyl pivalate (72.4 mg, 0.3 mmol) for 18 hours. The crude product was purified by flash column chromatography (gradient elution: 5% EtOAc in P.E. to 30% EtOAc in P.E.) and filtered through a pad of activated charcoal (10% EtOAc in P.E.) to afford the product as a colourless oil (58.2 mg, 72%). IR (ν_{max}/cm^{-1}): 2937, 2872, 1727, 1481, 1459, 1399, 1376, 1282, 1228, 1148, 1035. ^{1}H NMR (400 MHz, $CDCl_3$) δ: 4.25 (1 H, d, J = 11.8 Hz), 4.01 (1 H, d. J = 12.0 Hz), 3.19 (1 H, sxt, J = 7.1 Hz), 3.10 (1 H, sxt, J = 7.1 Hz), 2.97 (1 H, dd, J = 3.1, 5.6 Hz), 1.90 – 1.85 (1 H, m), 1.81 – 1.77 (1 H, m), 1.66 – 1.49 (6 H, m), 1.20 – 1.17 (12 H, m). ^{13}C NMR (101 MHz, $CDCl_3$) δ: 178.2, 169.8, 66.6, 59.1, 49.7, 39.1, 34.5, 27.3, 25.3, 19.4, 18.7, 17.2, 14.3. m/z HRMS found $[M + H]^+$ 268.1908, $C_{15}H_{26}NO_3$ requires 268.1907.

***cis*-7-ethyl-6-(((triisopropylsilyl)oxy)methyl)-7-azabicyclo[4.2.0]octan-8-one (2b)**

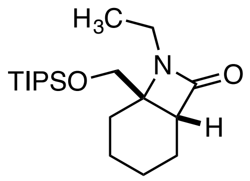

General procedure A was applied to *N*-ethyl-1-(((triisopropylsilyl)oxy)methyl)cyclohexan-1-amine (101.8 mg, 0.3 mmol) for 18 hours. The crude product was purified by flash column chromatography over silica (20% EtOAc in P.E.) and subsequently by flash column chromatography over alumina (gradient elution: 5% EtOAc in P.E. to 10% EtOAc in P.E.) to afford the product as a colourless oil (53.0 mg, 54%). IR (ν_{max}/cm^{-1}): 2941, 2866, 1743, 1462, 1401, 1375, 1199, 1116, 1066. ^{1}H NMR (400 MHz, $CDCl_3$) δ: 3.73 (2 H, dd, *J* = 10.4, 16.9 Hz), 3.23 – 3.11 (2 H, m), 2.90 (1 H, dd, *J* = 3.2, 6.1 Hz), 1.89 – 1.77 (2 H, m), 1.66 – 1.45 (6 H, m), 1.19 (3 H, t, *J* = 7.4 Hz), 1.08 – 1.04 (21 H, m). ^{13}C NMR (101 MHz, $CDCl_3$) δ: 170.5, 68.2, 61.0, 49.3, 34.6, 25.3, 19.8, 19.1, 18.1, 17.7, 14.5, 12.0. m/z HRMS found [M + H]$^{+}$ 340.2666, $C_{19}H_{38}O_2NSi$ requires 340.2666.

***cis*-6-butyl-7-ethyl-7-azabicyclo[4.2.0]octan-8-one (2c)**

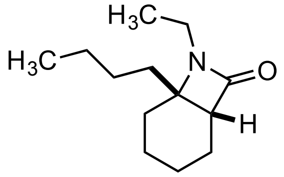

Prepared according to general procedure A using 1-butyl-*N*-ethylcyclohexan-1-amine (55.0 mg, 0.3 mmol), $Pd(OAc)_2$ (6.7 mg, 0.03 mmol), Xantphos (17 mg, 0.03 mmol), 1,4-benzoquinone (65 mg, 0.6 mmol) and silver(I) acetate (150 mg, 0.9 mmol) in toluene (3 mL) at 80 ºC for 18 hours. The crude product was purified by flash column chromatography (20% EtOAc in P.E.) and subsequently by flash column chromatography over alumina (20% EtOAc in P.E.) to afford the product as a colourless oil (37.0 mg, 59%). IR ν_{max}/cm^{-1} (film): 2929, 2861, 1734, 1455, 1399, 1376, 1334, 1302, 1191, 1142, 1086, 1056, 1035; ^{1}H NMR (400 MHz, $CDCl_3$) δ: 3.12 (2 H, d sept, *J* = 25.4, 7.2 Hz), 2.85 (1 H, dd, *J* = 2.9, 5.3 Hz), 1.94 – 1.80 (1 H, m), 1.75 – 1.63 (3 H, m), 1.63 – 1.45 (6 H, m), 1.39 – 1.23 (4 H, m), 1.19 (3 H, t, *J* = 7.3 Hz), 0.91 (3 H, t, *J* = 7.0 Hz); ^{13}C NMR (101 MHz, $CDCl_3$) δ: 170.5, 60.6, 51.5, 38.3, 34.1, 28.1, 26.7, 23.3, 19.8, 18.5, 17.7, 14.4, 14.2; m/z HRMS found [M + H]$^{+}$ 209.1786, $C_{13}H_{23}NO$ requires 209.1780.

(1,2-diethyl-3-methyl-4-oxoazetidin-2-yl)methyl pivalate (2d)

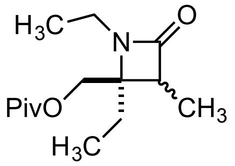

Prepared according to general procedure A using 2-ethyl-2-(ethylamino)butyl pivalate (64.0 mg, 0.3 mmol), $Pd(OAc)_2$ (6.7 mg, 0.03 mmol), Xantphos (17 mg, 0.03 mmol), 1,4-benzoquinone (65 mg, 0.6 mmol) and silver(I) acetate(150 mg, 0.9 mmol) in toluene (3 mL) at 80 ºC for 18 hours. The crude product was purified by flash column chromatography (gradient elution: 10% EtOAc in P.E. to 30% EtOAc in P.E.) to afford the product as a colourless oil (39.0 mg, 51%, inseparable 1:1 mixture of diastereomers). IR ν_{max}/cm^{-1} (film): 2972, 2935, 1728, 1480, 1461, 1399, 1368, 1280, 1148, 1092, 1035; ^1H NMR (400 MHz, $CDCl_3$) δ: 4.37 (1 H, d, *J* = 12.0 Hz), 4.35 (1 H, d, *J* = 12.0 Hz), 4.07 (2 H, d, *J* = 12.0 Hz), 3.21– 3.11 (2 H, m), 3.16 (2 H, quintet, *J* = 7.4 Hz), 3.00 (1 H, q, *J* = 7.7 Hz), 2.98 (1 H, q, *J* = 7.6 Hz), 1.93 (1 H, dq, *J*= 7.4, 14.8 Hz), 1.88 (1 H, dq, *J*= 7.6, 15.2 Hz), 1.70 (1 H, dq, *J*= 7.6, 15.1 Hz), 1.59 (1 H, dq, *J*= 7.6, 15.0 Hz), 1.25 – 1.15 (12 H, m), 1.22 (9 H, s), 1.21 (9 H, s), 0.94 (6 H, q, *J* = 7.5 Hz); ^{13}C NMR (101 MHz, $CDCl_3$) δ: 178.1, 178.1, 170.1, 170.1, 65.5, 65.2, 63.2, 63.0, 50.8, 49.6, 39.1, 39.0, 35.0, 34.9, 27.3, 27.3, 27.1, 23.8, 14.7 (2C), 9.2, 9.1, 9.0, 8.5; m/z HRMS found $[M + H]^+$ 256.1909, $C_{14}H_{26}NO_3$ requires 256.1907.

***cis*-7-isopropyl-6-(((triisopropylsilyl)oxy)methyl)-7-azabicyclo[4.2.0]octan-8-one (2e)**

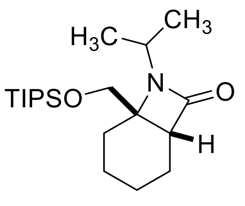

Prepared according to general procedure A using *N*-isopropyl-1-(((triisopropylsilyl)oxy)methyl)cyclohexan-1-amine (98.0 mg, 0.3 mmol), $Pd(OAc)_2$ (9.3 mg, 0.03 mmol), Xantphos (17 mg, 0.03 mmol), 1,4-benzoquinone (65 mg, 0.6 mmol) and silver(I) acetate (150 mg, 0.9 mmol) in toluene (3 mL) at 80 ºC for 18 hours. The crude product was purified by flash column chromatography (20% EtOAc in P.E.) to afford an inseparable mixture of *cis*-7-isopropyl-6-(((triisopropylsilyl)oxy)methyl)-7-azabicyclo[4.2.0]octan-8-one and 4-methyl-1-(1-(((triisopropylsilyl)oxy)methyl)cyclohexyl)azetidin-2-one as a colorless oil (21.0 mg, 20%, 1.5:1 ratio). The mixture was separated by preparative HPLC to obtain a clean sample of the title compound for analysis. IR ν_{max}/cm^{-1} (neat): 2943, 2867, 1743, 1464, 1382, 1119, 1067, 1014; ^1H NMR (400 MHz, $CDCl_3$) δ 3.74 (2H, d, *J* = 2.1 Hz), 3.55 (1H, spt, *J* = 6.8 Hz), 2.86 (1H, dd, *J* = 3.3, 6.3 Hz), 1.89 – 1.78 (2H, m), 1.72 – 1.63 (3H, m), 1.60 – 1.43 (3H, m), 1.33 (6H, dd, *J* = 2.7, 6.8 Hz),

1.17 – 0.98 (21H, m); ^{13}C NMR (101 MHz, $CDCl_3$) δ 169.9, 68.6, 61.6, 49.2, 44.7, 25.9, 21.8, 21.6, 19.9, 19.2, 18.2, 18.0, 12.1; m/z HRMS found $[M + H]^+$ 354.2827, $C_{20}H_{40}NO_2Si$ requires 354.2823.

4-methyl-1-(1-(((triisopropylsilyl)oxy)methyl)cyclohexyl)azetidin-2-one

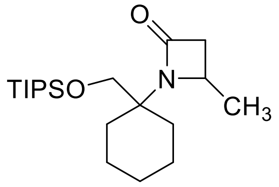

Prepared according to general procedure A using *N*-isopropyl-1-(((triisopropylsilyl)oxy)methyl)cyclohexan-1-amine (98.0 mg, 0.3 mmol), $Pd(OAc)_2$ (9.3 mg, 0.03 mmol), Xantphos (17 mg, 0.03 mmol), 1,4-benzoquinone (65 mg, 0.6 mmol) and silver(I) acetate (150 mg, 0.9 mmol) in toluene (3 mL) at 80 ºC for 18 hours. The crude product was purified by flash column chromatography (20% EtOAc in P.E.) to afford an inseparable mixture of 4-methyl-1-(1-(((triisopropylsilyl)oxy)methyl)cyclohexyl)azetidin-2-one and *cis*-7-isopropyl-6-(((triisopropylsilyl)oxy)methyl)-7-azabicyclo[4.2.0]octan-8-one as a colorless oil (21.0 mg, 20%, 1:1.5 ratio). The mixture was separated by preparative HPLC to obtain a clean sample of the title compound for analysis.

IR ν_{max}/cm^{-1} (neat): 2940, 2865, 1744, 1463, 1379, 1364, 1348, 1134, 1098, 1068; ^{1}H NMR (400 MHz, $CDCl_3$) δ 3.85 (1H, qt d, *J* = 2.1, 5.7 Hz), 3.70 (2H, dd, *J* = 9.6, 22.6 Hz), 2.97 (1H, dd, *J* = 5.3, 14.3 Hz), 2.33 (1 H, dd, *J* = 2.3, 14.3 Hz), 2.24 – 2.12 (1H, m), 1.94 (1H, app. d, *J* = 13.5 Hz), 1.68 – 1.43 (6H, m), 1.38 (3 H, d, *J* = 6.1 Hz), 1.35 – 1.24 (2H, m), 1.23 – 0.92 (21H, m); ^{13}C NMR (101 MHz, $CDCl_3$) δ 167.8, 68.4, 61.4, 47.2, 43.1, 30.7, 25.7, 22.6, 22.3, 22.2, 18.2, 12.1; m/z HRMS found $[M + H]^+$ 354.2828, $C_{20}H_{40}NO_2Si$ requires 354.2823.

***cis*-7-heptyl-8-oxo-7-azabicyclo[4.2.0]octan-6-yl)methyl pivalate (2f)**

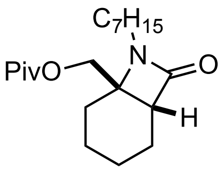

Prepared according to general procedure A using (1-(heptylamino)cyclohexyl)methyl pivalate (93.0 mg, 0.3 mmol), $Pd(OAc)_2$ (6.7 mg, 0.03 mmol), Xantphos (17 mg, 0.03 mmol), 1,4-benzoquinone (65 mg, 0.6 mmol) and silver(I) acetate (150 mg, 0.9 mmol) in toluene (3 mL) at 80 ºC for 18 hours. The

crude product was purified by flash column chromatography (gradient elution: 5% EtOAc in P.E. to 15% EtOAc in P.E.) to afford the product as a brown oil (71 mg, 70%). IR ν_{max}/cm^{-1} (film): 2933, 2861, 1730, 1481, 1461, 1393, 1368, 1276, 1148, 1037; ^{1}H NMR (400 MHz, $CDCl_3$) δ: 4.24 (1 H, d, *J* = 11.9 Hz), 4.02 (1 H, d, *J* = 11.9 Hz), 3.15 – 3.06 (1 H, m), 3.04 – 2.95 (2 H, m), 1.94 – 1.84 (1 H, m), 1.79 (1 H, dt, *J* = 3.6, 8.3 Hz), 1.72 – 1.43 (8 H, m), 1.33 – 1.24 (8 H, m), 1.21 (9 H, s), 0.87 (3 H, t, *J* = 6.9 Hz); ^{13}C NMR (101 MHz, $CDCl_3$) δ: 178.2, 170.1, 66.8, 59.0, 49.6, 40.2, 39.1, 31.9, 29.3, 29.1, 27.4, 27.3, 25.3, 22.7, 19.6, 18.8, 17.3, 14.2; m/z HRMS found $[M + H]^+$ 338.2691, $C_{20}H_{36}NO_3$ requires 338.2690.

(2-ethyl-3-methyl-4-oxo-1-propylazetidin-2-yl)methyl pivalate (2g)

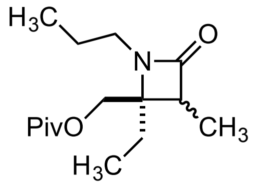

Prepared according to general procedure A using 2-ethyl-2-(propylamino)butyl pivalate (73.0 mg, 0.3 mmol), $Pd(OAc)_2$ (6.7 mg, 0.03 mmol), Xantphos (17 mg, 0.03 mmol), 1,4-benzoquinone (65 mg, 0.6 mmol) and silver(I) acetate(150 mg, 0.9 mmol) in toluene (3 mL) at 80 ºC for 18 hours. The crude product was purified by flash column chromatography (20% EtOAc in P.E.) and subsequently by flash column chromatography over alumina (20%% EtOAc in P.E.) to afford the product as a colourless oil (53.0 mg, 66%, inseparable 1:1 mixture of diastereomers). IR ν_{max}/cm^{-1} (film): 2972, 2873, 1730, 1483, 1459, 1399, 1365, 1278, 1148, 1094, 1033; ^{1}H NMR (400 MHz, $CDCl_3$) δ: 4.36 (1 H, d, *J* = 11.9 Hz), 4.34 (1 H, d, *J* = 12.0 Hz), 4.06 (2 H, d, *J* = 12.0 Hz), 3.10 – 2.90 (6 H, m), 1.99 – 1.82 (2 H, m), 1.75 – 1.63 (2 H, m), 1.64 – 1.50 (4 H, m), 1.26 – 1.16 (6 H, m), 1.21 (9 H, s), 1.20 (9 H, s), 0.96 – 0.90 (6 H, m), 0.89 (3 H, t, *J* = 7.5 Hz), 0.89 (3 H, t, *J* = 7.4 Hz); ^{13}C NMR (101 MHz, $CDCl_3$) δ: 178.1, 178.0, 170.4, 170.4, 65.5, 65.2, 63.1, 62.8, 50.7, 49.5, 42.2, 42.1, 39.1, 39.0, 27.3 (2C), 27.1, 23.7, 22.8, 22.7, 11.8, 11.8, 9.2, 9.1, 9.1, 8.5; m/z HRMS found $[M + H]^+$ 270.2065, $C_{15}H_{28}NO_3$ requires 270.2064.

(*cis* -7-(3-ethoxy-3-oxopropyl)-8-oxo-7-azabicyclo[4.2.0]octan-6-yl)methyl pivalate (2h)

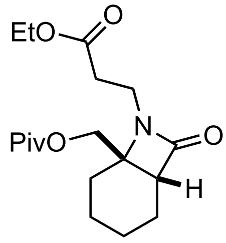

Prepared according to general procedure A using (1-((3-ethoxy-3-oxopropyl)amino)cyclohexyl)methyl pivalate (94.0 mg, 0.3 mmol), $Pd(OAc)_2$ (6.7 mg, 0.03 mmol), Xantphos (17 mg, 0.03 mmol), 1,4-benzoquinone (65 mg, 0.6 mmol) and silver(I) acetate (150 mg, 0.9 mmol) in toluene (3 mL) at 80 ºC for 18 hours. The crude product was purified by flash column chromatography (gradient elution: 20% EtOAc in P.E. to 30% EtOAc in P.E.) and subsequently by filtration through activated charcoal, eluting with EtOAc, to afford the product as a yellow oil (84 mg, 82%). IR ν_{max}/cm^{-1} (film): 2937, 2873, 1726, 1479, 1459, 1399, 1367, 1282, 1146, 1031; ^{1}H NMR (400 MHz, $CDCl_3$) δ: 4.23 (1 H, d, *J* = 11.9 Hz), 4.14 (2 H, q, *J* = 7.1 Hz), 4.04 (1 H, d, *J* = 11.9 Hz), 3.38 (2 H, dtd, *J* = 7.5, 14,4. 21.8 Hz), 2.97 (1 H, dd, *J* = 3.7, 5.9 Hz), 2.76 – 2.54 (2 H, m), 1.93 – 1.76 (2 H, m), 1.69 – 1.47 (6 H, m), 1.25 (3 H, t, *J* = 7.1 Hz), 1.21 (9 H, s); ^{13}C NMR (101 MHz, $CDCl_3$) δ: 178.1, 171.4, 170.4, 66.5, 61.0, 59.4, 49.7, 39.1, 35.7, 33.7, 27.3, 25.0, 19.5, 18.7, 17.3, 14.3; m/z HRMS found $[M + H]^+$ 340.2120, $C_{18}H_{30}NO_5$ requires 340.2118.

(*cis* -8-oxo-7-(2-(phenylsulfonyl)ethyl)-7-azabicyclo[4.2.0]octan-6-yl)methyl pivalate (2i)

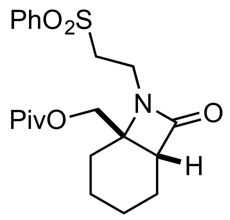

Prepared according to general procedure A using (1-((2-(phenylsulfonyl)ethyl)amino)cyclohexyl)methyl pivalate (1. 15 g, 3 mmol), $Pd(OAc)_2$ (67.0 mg, 0.3 mmol), Xantphos (170.0 mg, 0.3 mmol), 1,4-benzoquinone (655.0 mg, 6.0 mmol) and silver(I) acetate (1.50 g, 9.0 mmol) in toluene (30 mL) at 80 ºC for 18 hours. The crude product was purified by flash column chromatography (50% EtOAc in P.E.) to afford the product as a white amorphous solid (833 mg, 68%). m.p. 61–63 °C; IR ν_{max}/cm^{-1} (film): 2940, 2865, 1752, 1724, 1477, 1449, 1403, 1368, 1306, 1280, 1151, 1140, 1082, 1050, 1031; ^{1}H NMR (500 MHz, $CDCl_3$) δ: 7.92 (2 H, app d, *J* = 7.2 Hz), 7.69 (1 H, tt, *J* = 7.5, 1.1 Hz), 7.59 (2 H, app t, *J* = 7.7 Hz), 4.27 (1 H, d, *J* = 12.0 Hz), 3.97 (1 H, d, *J* = 12.0 Hz), 3.60 – 3.35 (4 H, m), 2.92 (1 H, dd, *J* = 6.5, 3.8 Hz), 1.88 – 1.77 (2 H, m), 1.68 – 1.39 (6

H, m), 1.20 (9 H, s); ^{13}C NMR (126 MHz, $CDCl_3$) δ: 178.0, 170.3, 138.9, 134.3, 129.7, 128.2, 66.7, 59.7, 54.1, 50.0, 39.1, 34.0, 27.3, 24.9, 19.4, 18.8, 17.3; m/z HRMS found $[M + H]^+$ 408.1836, $C_{21}H_{30}NO_5S$ requires 408.1839.

(*cis* -8-oxo-7-(3-(pyridin-2-yl)propyl)-7-azabicyclo[4.2.0]octan-6-yl)methyl pivalate (2j)

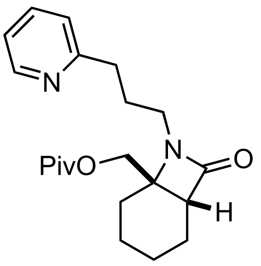

Prepared according to general procedure A using (1-((3-(pyridin-2-yl)propyl)amino)cyclohexyl)methyl pivalate (100.0 mg, 0.3 mmol), $Pd(OAc)_2$ (6.7 mg, 0.03 mmol), Xantphos (17 mg, 0.03 mmol), 1,4-benzoquinone (65 mg, 0.6 mmol) and silver(I) acetate (150 mg, 0.9 mmol) in toluene (3 mL) at 80 ºC for 18 hours. The crude product was purified by flash column chromatography (gradient elution: 60% EtOAc in P.E. to 70% EtOAc in P.E.) to afford the product as a dark brown oil (83 mg, 77%). IR ν_{max}/cm^{-1} (film): 2944, 2877, 1724, 1594, 1568, 1475, 1435, 1399, 1368, 1278, 1144, 1035; ^{1}H NMR (400 MHz, $CDCl_3$) δ: 8.51 (1 H, d, *J* = 4.1 Hz), 7.60 (1 H, td, *J* = 7.7, 1.8 Hz), 7.20 (1 H, d, *J* = 7.8 Hz), 7.12 (1 H, dd, *J* = 7.4, 5.0 Hz), 4.23 (1 H, d, *J* = 11.9 Hz), 4.04 (1 H, d, *J* = 11.9 Hz), 3.07 (2 H, d sept, *J* = 14.2, 7.6 Hz), 2.98 (1 H, dd, *J* = 5.9, 3.6 Hz), 2.83 (2H, t, *J* = 7.6 Hz), 2.04 (2 H, qt, *J* = 7.6 Hz), 1.95 – 1.84 (1 H, m), 1.84 – 1.74 (1 H, m), 1.74 – 1.44 (6 H, m), 1.18 (9 H, s); ^{13}C NMR (101 MHz, $CDCl_3$) δ: 178.2, 170.3, 160.8, 149.3, 136.8, 123.2, 121.4, 66.8, 59.1, 49.7, 39.7, 39.1, 35.8, 29.0, 27.3, 25.3, 19.6, 18.8, 17.4; m/z HRMS found $[M + H]^+$ 359.2329, $C_{21}H_{31}N_2O_3$ requires 359.2329.

***cis*-7-cyclohexyl-6-(((triisopropylsilyl)oxy)methyl)-7-azabicyclo[4.2.0]octan-8-one (2k)**

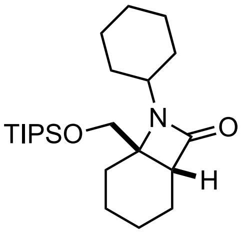

Prepared according to general procedure A using *N*-cyclohexyl-1-(((triisopropylsilyl)oxy)methyl)cyclohexan-1-amine (110 mg, 0.3 mmol), $Pd(OAc)_2$ (6.7 mg, 0.03

mmol), Xantphos (17 mg, 0.03 mmol), 1,4-benzoquinone (65 mg, 0.6 mmol) and silver(I) acetate (150 mg, 0.9 mmol) in toluene (3 mL) at 80 ºC for 18 hours. The crude mixture was purified by flash column chromatography (10% EtOAc in P.E.) and subsequently by flash column chromatography over alumina (10% EtOAc in P.E.) to afford the tile compound as a light yellow oil (29.0 mg, 25%). IR ν_{max}/cm^{-1} (neat): 2931, 2863, 1736, 1450, 1364, 1288, 1115, 1090, 1065; ^{1}H NMR (500 MHz, $CDCl_3$) δ 3.75 (2 H, s), 3.17 (1 H, tt, *J* = 3.9, 11.8 Hz), 2.86 (1 H, dd, *J* = 3.4, 6.3 Hz), 1.97 – 1.45 (16 H, m), 1.29 – 1.16 (2 H, m), 1.08 (21 H, m); ^{13}C NMR (126 MHz, $CDCl_3$) δ 169.7, 68.8, 61.4, 52.6, 49.0, 31.8, 31.6, 25.8, 25.8, 25.7, 25.3, 19.8, 19.1, 18.0, 17.8, 11.9; m/z HRMS found $[M + H]^+$ 394.3127, $C_{23}H_{44}NO_2Si$ requires 394.3136.

***cis*-7-methyl-8-oxo-7-azabicyclo[4.2.0]octan-6-yl)methyl pivalate (2m)**

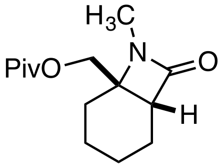

Prepared according to general procedure A using (1-(methylamino)cyclohexyl)methyl pivalate (68.2 mg, 0.3 mmol), $Pd(OAc)_2$ (6.7 mg, 0.03 mmol), Xantphos (17 mg, 0.03 mmol), 1,4-benzoquinone (65 mg, 0.6 mmol) and silver(I) acetate (150 mg, 0.9 mmol) in toluene (3 mL) at 80 ºC for 18 hours. Purification by flash column chromatography (30% EtOAc in P.E.) gave the desired product as a colorless oil (65.6 mg, 86%). IR ν_{max}/cm^{-1} (film): 2939, 2872, 1727, 1480, 1461, 1421, 1389, 1366, 1281, 1148, 1032. ^{1}H NMR (400 MHz, $CDCl_3$) δ: 4.30 (1 H, d, *J* = 11.9 Hz), 3.92 (1 H, d, *J* = 11.9 Hz), 2.99 – 2.97 (1 H, m), 2.65 (3 H, s), 1.88 – 1.72 (2 H, m), 1.66 – 1.45 (6 H, m), 1.17 (9H, s). ^{13}C NMR (101 MHz, $CDCl_3$) δ: 178.1, 170.0, 65.9, 58.6, 49.6, 39.0, 27.2, 24.8, 24.3, 19.4, 18.7, 17.1. m/z HRMS found $[M + H]^+$ 254.1751, $C_{14}H_{24}O_3N$ requires 254.1751

***cis*-7-methyl-6-(((triisopropylsilyl)oxy)methyl)-7-azabicyclo[4.2.0]octan-8-one (2n)**

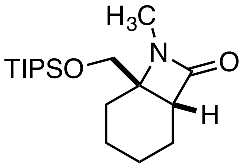

To a 250 mL round-bottomed flask equipped with large oval shaped stirrer bar was added palladium (II) acetate (168 mg, 0.75 mmol, 0.1 equiv), silver(I) acetate (3.75 g, 22.5 mmol, 3 equiv), Xantphos (425 mg, 0.75 mmol, 0.1 equiv) and 1,4-benzoquinone (1.65 g, 15 mmol, 2 equiv). *N*-methyl-1-

(((triisopropylsilyl)oxy)methyl)cyclohexan-1-amine (2.24 g, 7.5 mmol) was subsequently dissolved in toluene (30 mL) and added to the flask. The flask was sealed with a new septa and Teflon tape and purged with a balloon of CO (3 cycles). The flask was placed into a preheated oil bath at 80 °C and stirred vigorously for 18 hours. The flask was allowed to cool to room temperature, filtered over a plug of celite and washed with EtOAc. The solvent was removed *in vacuo* and the crude product was purified by flash column chromatography (5% EtOAc in P.E. to 30% EtOAc in P.E.) and subsequently by flash column chromatography over alumina (5% EtOAc in P.E. to 30% EtOAc in P.E.) to afford the product as a colourless oil (1.98 g, 81%). IR (ν_{max}/cm^{-1}): 2941, 2865, 1746, 1463, 1418, 1387, 1334, 1295, 1247, 1116, 1092, 1066. ^{1}H NMR (400 MHz, $CDCl_3$) δ: 3.74 (1 H, d, *J* = 10.1 Hz), 3.86 (1 H, d, *J* = 10.1 Hz), 2.92 – 2.90 (1 H, m), 2.71 (3 H, s), 1.88 – 1.77 (2 H, m), 1.62 – 1.45 (6 H, m), 1.09 – 1.02 (21 H, m). ^{13}C NMR (101 MHz, $CDCl_3$) δ: 170.6, 67.8, 60.2, 49.2, 25.1, 24.3, 19.7, 19.2, 18.1, 17.7, 12.0. m/z HRMS found $[M + H]^+$ 326.2503, $C_{18}H_{36}NO_2Si$ requires 326.2510

***cis*-(7-methyl-8-oxo-3-oxa-7-azabicyclo[4.2.0]octan-6-yl)methyl pivalate (2o)**

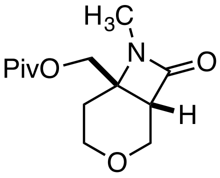

Prepared according to general procedure A using (4-(methylamino)tetrahydro-2*H*-pyran-4-yl)methyl pivalate (73.5 mg, 0.3 mmol), $Pd(OAc)_2$ (6.7 mg, 0.03 mmol), Xantphos (17 mg, 0.03 mmol), 1,4-benzoquinone (65 mg, 0.6 mmol) and silver(I) acetate (150 mg, 0.9 mmol) in toluene (3 mL) at 80 ºC for 18 hours. Purification by flash column chromatography (gradient elution: 50% EtOAc in P.E. to 80% EtOAc in P.E.) gave the desired product as a colorless oil (65.6 mg, 86%). IR ν_{max}/cm^{-1} (film): 2973, 2874, 1732, 1614, 1480, 1460, 1415, 1359, 1339, 1148, 1118. ^{1}H NMR (400 MHz, $CDCl_3$) δ: 5.59 (1 H, dd, *J* = 10.7, 17.3 Hz), 5.41 (1 H, d, *J* = 10.8 Hz), 5.22 (1 H, d, *J* = 17.4 Hz), 4.29 (1 H, d, *J* = 11.8 Hz), 4.11 (1 H, d, *J* = 11.8 Hz), 3.03 (1 H, td, *J* = 2.9, 12.3 Hz), 2.94 (3 H, s), 2.72 (1 H, ddd, *J* = 3.3, 5.9, 12.3 Hz), 2.32 (1 H, ddd, *J* = 3.2, 11.7, 14.4 Hz), 2.08 (1 H, ddd, *J* = 2.9, 4.9, 14.0 Hz), 1.19 (9 H, s). ^{13}C NMR (101 MHz, $CDCl_3$) δ: 177.8, 167.1, 135.3, 118.2, 66.1, 64.5, 39.0, 32.5, 31.2, 27.2, 22.8. m/z HRMS found $[M + H]^+$ 256.1540, $C_{13}H_{22}O_4N$ requires 256.1549

***cis*-(7-methyl-8-oxo-3-thia-7-azabicyclo[4.2.0]octan-6-yl)methyl pivalate (2p)**

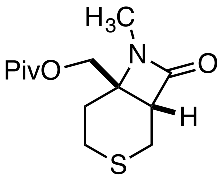

Prepared according to general procedure A using (4-(methylamino)tetrahydro-2*H*-thiopyran-4-yl)methyl pivalate (68.8 mg, 0.3 mmol), $Pd(OAc)_2$ (6.7 mg, 0.03 mmol), Xantphos (17 mg, 0.03 mmol), 1,4-benzoquinone (65 mg, 0.6 mmol) and silver(I) acetate (150 mg, 0.9 mmol) in toluene (3 mL) at 80 ºC for 18 hours. Purification by flash column chromatography (30% EtOAc in P.E.) gave the desired product as a colorless oil (65.5 mg, 84%). IR ν_{max}/cm^{-1} (film): 2979, 1732, 1683, 1480, 1462, 1396, 1280, 1149, 1070, 1037. ^1H NMR (400 MHz, $CDCl_3$) δ: 4.55 – 4.51 (1 H, m), 4.38 – 4.35 (1 H, m), 3.94 – 3.86 (3 H, m), 2.75 (3 H, s), 2.68 – 2.61 (1 H, m), 2.49 – 2.43 (1 H, m), 2.06 – 2.01 (1 H, m), 1.84 – 1.77 (1 H, m), 1.15 (9 H, s). ^{13}C NMR (101 MHz, $CDCl_3$) δ: 177.9, 173.3, 79.3, 73.5, 66.9, 64.0, 38.9, 38.2, 32.6, 27.2, 25.6. m/z HRMS found $[M + H]^+$ 272.1314, $C_{13}H_{22}O_3NS$ requires 272.1320.

***cis*-(7-methyl-8-oxo-3-tosyl-3,7-diazabicyclo[4.2.0]octan-6-yl)methyl pivalate (2q)**

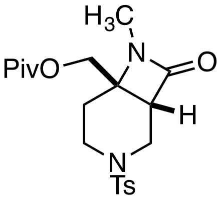

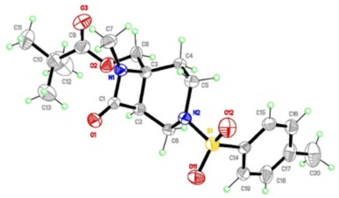

Prepared according to general procedure A using (4-(methylamino)-1-tosylpiperidin-4-yl)methyl pivalate (114.7 mg, 0.3 mmol), $Pd(OAc)_2$ (6.7 mg, 0.03 mmol), Xantphos (17 mg, 0.03 mmol), 1,4-benzoquinone (65 mg, 0.6 mmol) and silver(I) acetate (150 mg, 0.9 mmol) in toluene (3 mL) at 80 ºC for 18 hours. Purification by flash column chromatography (30% EtOAc in P.E.) gave the desired product as an off-white solid (89.0 mg, 73%). m.p. 140–142 °C; IR ν_{max}/cm^{-1} (film): 2959, 1756, 1737, 1716, 1599, 1461, 1399, 1378, 1358, 1340, 1308, 1286, 1166, 1090, 1040. ^1H NMR (400 MHz, $CDCl_3$) δ: 7.64 (2 H, d, J = 8.2 Hz), 7.29 (2 H, d, J = 8.2 Hz), 4.35 (1 H, d, J = 11.9 Hz), 3.92 (1 H, d, J = 11.9 Hz), 3.65 (1 H, dd, J = 3.3, 13.1 Hz), 3.47 (1 H, dt, J = 5.0, 12.0 Hz), 3.32 (1 H, dd, J = 5.9, 13.0 Hz), 3.14 (1 H, dd, J = 3.3, 5.6 Hz), 3.00 (1 H, td, J = 4.1, 11.7 Hz), 2.61 (3 H, s), 2.39 (3 H, s), 2.00 – 1.94 (1 H, m), 1.84 – 1.77 (1 H, m), 1.15 (9 H, s). ^{13}C NMR (101 MHz, $CDCl_3$) δ: 177.7, 166.7, 143.8, 134.3, 129.8, 127.5, 64.5, 56.8, 49.7, 40.0, 39.2, 39.0, 27.1, 25.2, 25.0, 21.6. m/z HRMS found $[M + H]^+$ 409.1793, $C_{20}H_{29}O_5N_2S$ requires 409.1792.

(2-ethyl-1,3-dimethyl-4-oxoazetidin-2-yl)methyl pivalate (2r)

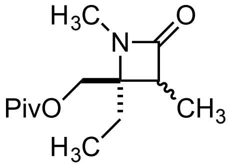

Prepared according to general procedure A using 2-ethyl-2-(methylamino)butyl pivalate (65.0 mg, 0.3 mmol), $Pd(OAc)_2$ (6.7 mg, 0.03 mmol), Xantphos (17 mg, 0.03 mmol), 1,4-benzoquinone (65 mg, 0.6 mmol) and silver(I) acetate(150 mg, 0.9 mmol) in toluene (3 mL) at 80 ºC for 18 hours. The crude product was purified by flash column chromatography (10% EtOAc in P.E.) and subsequently by flash column chromatography over alumina (10% EtOAc in P.E.) to afford the product as a yellow oil (53.0 mg, 73%, inseparable 1:1 mixture of diastereomers). IR ν_{max}/cm^{-1} (film): 2964, 2940, 1728, 1479, 1459, 1417, 1395, 1368, 1276, 1146, 1086, 1039; ^1H NMR (400 MHz, $CDCl_3$) δ: 4.41 (1 H, d, *J* = 12.0 Hz), 4.37 (1 H, d, *J* = 11.9 Hz), 4.08 (1 H, d, *J* = 11.9 Hz), 4.04 (1 H, d, *J* = 12.0 Hz), 3.01 (2 H, q, *J* = 7.6 Hz), 2.72 (3 H, s), 2.71 (3 H, s), 1.96 – 1.81 (2 H, m), 1.72 – 1.61 (1 H, m), 1.61 – 1.52 (1 H, m), 1.24-1.20 (6 H, m), 1.21 (9 H, s), 1.20 (9 H, s), 0.96 (3 H, t, *J* = 7.6 Hz), 0.93 (3 H, t, *J* = 7.5 Hz); ^{13}C NMR (101 MHz, $CDCl_3$) δ: 178.1, 178.1, 170.3, 170.2, 64.7, 64.7, 62.5, 62.3, 50.9, 49.7, 39.1, 39.0, 27.3, 27.3, 26.0, 25.4, 25.3, 22.9, 9.1, 9.1, 9.0, 8.3; m/z HRMS found $[M + H]^+$ 242.1751, $C_{13}H_{24}NO_3$ requires 242.1751.

***N*-(2-(*cis*-7-methyl-8-oxo-7-azabicyclo[4.2.0]octan-6-yl)ethyl)phthalimide (2s)**

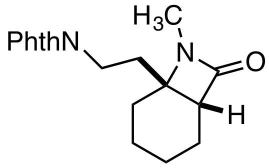

Prepared according to general procedure A using *N*-(2-(1-(methylamino)cyclohexyl)ethyl)phthalimide (57.3 mg, 0.3 mmol), $Pd(OAc)_2$ (9.3 mg, 0.03 mmol), Xantphos (17 mg, 0.03 mmol), 1,4-benzoquinone (65 mg, 0.6 mmol) and silver(I) pivalate (189 mg, 0.9 mmol) in toluene (3 mL) at 80 ºC for 18 hours. The crude product was purified by flash column chromatography (gradient elution: 30% EtOAc in P.E. to 60% EtOAc in P.E.) and subsequently by flash column chromatography over alumina (gradient elution: 20% EtOAc in P.E. to 50% EtOAc in P.E.) to afford the product as a white crystalline solid (74.5 mg, 80%) (Product contains 5 % impurity by ^1H NMR spectroscopy). m.p. 124–126 °C. IR ν_{max}/cm^{-1} (film): 2937, 2872, 1771, 1736, 1707, 1614, 1466, 1436, 1407, 1394, 1378, 1275, 1252, 1189, 1138, 1092. ^1H NMR (400 MHz, $CDCl_3$) δ: 7.85 (2 H, dd, *J* = 3.1, 5.5 Hz), 7.72 (2

H, dd, $J = 3.1, 5.5$ Hz), 3.81 – 3.66 (2 H, m), 3.08 – 3.06 (1 H, m), 2.74 (3 H, s), 2.09 – 2.01 (1 H, m), 1.97 – 1.89 (2 H, m), 1.76 – 1.45 (7 H, m). ^{13}C NMR (101 MHz, $CDCl_3$) δ: 170.0, 168.2, 134.3, 132.2, 123.5, 58.5, 51.4, 35.5, 33.5, 27.2, 24.5, 19.4, 18.2, 17.1. m/z HRMS found $[M + H]^+$ 313.1552, $C_{18}H_{21}N_2O_3$ requires 313.1552

***cis*-(8-methyl-9-oxo-8-azabicyclo[5.2.0]nonan-7-yl)methyl pivalate (2t)**

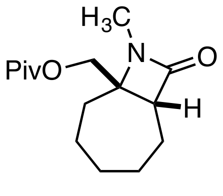

Prepared according to general procedure A using (1-(methylamino)cycloheptyl)methyl pivalate (72.4 mg, 0.3 mmol), $Pd(OAc)_2$ (6.7 mg, 0.03 mmol), Xantphos (17 mg, 0.03 mmol), 1,4-benzoquinone (65 mg, 0.6 mmol) and silver(I) acetate (150 mg, 0.9 mmol) in toluene (3 mL) at 80 ºC for 18 hours. Purification by flash column chromatography (20% EtOAc in P.E.) gave the desired product as a colorless oil (71.0 mg, 89%). IR ν_{max}/cm^{-1} (film): 2926, 2859, 1728, 1480, 1450, 1421, 1392, 1366, 1281, 1146, 1090, 1059, 1035. ^{1}H NMR (400 MHz, $CDCl_3$) δ: 4.35 – 4.31 (1 H, m), 3.97 (1 H, dt, $J = 1.6, 11.9$ Hz), 3.08 (1 H, d, 4.4 Hz), 2.66 (3 H, s), 1.89 – 1.80 (2 H, m), 1.64 – 1.33 (8 H, m), 1.17 (9 H, s). ^{13}C NMR (101 MHz, $CDCl_3$) δ: 178.0, 1692, 66.3, 63.8, 56.2, 39.0, 31.6, 30.0, 27.2, 27.1, 25.7, 24.8, 24.0. m/z HRMS found $[M + H]^+$ 268.1907, $C_{15}H_{26}O_3N$ requires 268.1907

***cis*-(6-methyl-7-oxo-6-azabicyclo[3.2.0]heptan-5-yl)methyl pivalate (2u)**

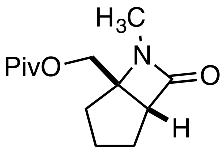

Prepared according to general procedure A using (1-(methylamino)cyclopentyl)methyl pivalate (64.0 mg, 0.3 mmol), $Pd(OAc)_2$ (6.7 mg, 0.03 mmol), Xantphos (17 mg, 0.03 mmol), 1,4-benzoquinone (65 mg, 0.6 mmol) and silver(I) acetate (150 mg, 0.9 mmol) in toluene (3 mL) at 80 ºC for 18 hours. Purification by flash column chromatography (40% EtOAc in P.E.) gave the desired product as a colorless oil (59.0 mg, 82%). IR ν_{max}/cm^{-1} (film): 2959, 2874, 1728, 1481, 1461, 1419, 1335, 1282, 1150, 1084. ^{1}H NMR (400 MHz, $CDCl_3$) δ: 4.50 (1 H, d, $J = 11.9$ Hz), 4.07 (1 H, d, $J = 11.9$ Hz), 3.22 (1 H, app d, $J = 7.6$ Hz), 2.65 (3 H, s), 2.00 – 1.95 (1 H, m), 1.92 – 1.84 (2 H, m), 1.68 – 1.55 (1

H, m), 1.51 – 1.42 (1 H, m), 1.31 – 1.22 (1 H, m), 1.17 (9 H, s). ^{13}C NMR (101 MHz, $CDCl_3$) δ: 178.0, 168.8, 68.6, 64.5, 57.2, 39.0, 27.8, 27.2, 25.1, 24.4, 23.7. m/z HRMS found $[M + H]^+$ 240.1593, $C_{13}H_{22}O_3N$ requires 240.1594.

***cis*-2-methyl-1-(2-((triisopropylsilyl)oxy)ethyl)-2-azabicyclo[2.2.0]hexan-3-one (2v)**

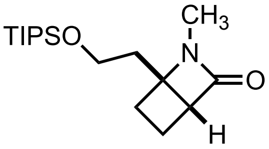

Prepared according to general procedure A using *N*-methyl-1-(2-((triisopropylsilyl)oxy)ethyl)cyclobutan-1-amine (85.7 mg, 0.3 mmol), $Pd(OPiv)_2$ (6.7 mg, 0.03 mmol), Xantphos (17 mg, 0.03 mmol), 1,4-benzoquinone (65 mg, 0.6 mmol) and silver(I) acetate (150 mg, 0.9 mmol) in toluene (3 mL) at 80 ºC for 18 hours. The crude product was purified by flash column chromatography (gradient elution: 5% EtOAc in P.E. to 30% EtOAc in P.E.) and subsequently by flash column chromatography over alumina (gradient elution: 5% EtOAc in P.E. to 30% EtOAc in P.E.) to afford the product as a colourless oil (78.9 mg, 84%). IR ν_{max}/cm^{-1} (film): 2942, 2866, 1744, 1463, 1413, 13784, 1292, 1246, 1225, 1187, 1097. ^{1}H NMR (400 MHz, $CDCl_3$) δ: 3.75 (2 H, dt, *J* = 1.0, 6.3 Hz), 3.40 (1 H, dd, *J* = 2.2, 8.9 Hz), 2.75 (3 H, s), 2.37 – 2.28 (1 H, m), 2.20 (1 H, dt, *J* = 5.5, 12.3 Hz), 2.06 – 1.92 (2 H, m), 1.90 (2 H, t, *J* = 6.3 Hz), 1.09 – 1.02 (21 H, m). ^{13}C NMR (101 MHz, $CDCl_3$) δ: 171.4, 62.5, 59.8, 52.8, 35.3, 27.1, 24.9, 18.1 (TIPS CH_3 and cyclo-C_4), 12.0. m/z HRMS found $[M + H]^+$ 312.2354, $C_{17}H_{34}NSi$ requires 312.2353

***cis*-3-methyl-4-oxo-3-azatricyclo[4.2.1.0^{2,5}]nonan-2-yl)methyl pivalate (2w)**

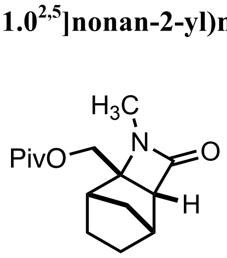

Prepared according to general procedure A using endo-2-(methylamino)norbornane-2-methyl pivalate (71.8 mg, 0.3 mmol), $Pd(OAc)_2$ (6.7 mg, 0.03 mmol), Xantphos (17 mg, 0.03 mmol), 1,4-benzoquinone (65 mg, 0.6 mmol) and silver(I) acetate (150 mg, 0.9 mmol) in toluene (3 mL) at 80 ºC for 18 hours. The crude product was purified by flash column chromatography (gradient elution: 5% EtOAc in P.E. to 20% EtOAc in P.E.) and subsequently by flash column chromatography over

alumina (gradient elution: 5% EtOAc in P.E. to 30% EtOAc in P.E.) to afford the product as a colourless oil (55.0 mg, 69%). IR ν_{max}/cm^{-1} (film): 2959, 2878, 1724, 1480, 1459, 1419, 1382, 1331, 1303, 1281, 1149. ^{1}H NMR (400 MHz, $CDCl_3$) δ: 4.49 (1 H, d, *J* = 12.3 Hz), 4.01 (1 H, d, *J* = 12.3 Hz), 3.19 (1 H, d, *J* = 5.7 Hz), 2.73 (3 H, s), 2.45 (1 H, br s), 2.24 (1 H, app. d, *J* = 3.3 Hz), 1.77 (1 H, d, *J* = 10.6 Hz), 1.67 – 1.53 (4 H, m), 1.44 – 1.39 (1 H, m), 1.19 (9 H, s). ^{13}C NMR (101 MHz, $CDCl_3$) δ: 178.2, 169.0, 69.1, 62.8, 61.3, 43.4, 39.3, 39.1, 36.5, 27.3, 27.1, 25.8, 24.3. m/z HRMS found $[M + H]^+$ 266.1752, $C_{15}H_{24}O_3N$ requires 266.1751

***cis*-2-(2-(1-tosyl-1H-indol-3-yl)ethyl)-1-(2-((triisopropylsilyl)oxy)ethyl)-2-azabicyclo[2.2.0]hexan-3-one (2x)**

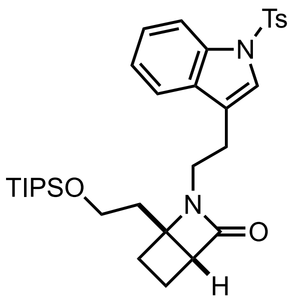

Prepared according to general procedure A using *N*-(2-(1-tosyl-1H-indol-3-yl)ethyl)-1-(2-((triisopropylsilyl)oxy)ethyl)cyclobutan-1-amine (171.0 mg, 0.3 mmol), $Pd(OAc)_2$ (6.7 mg, 0.03 mmol), Xantphos (17 mg, 0.03 mmol), 1,4-benzoquinone (65 mg, 0.6 mmol) and silver(I) acetate (150 mg, 0.9 mmol) in toluene (3 mL) at 80 ºC for 18 hours. The crude product was purified by flash column chromatography (20% EtOAc in P.E.) and subsequently by flash column chromatography over alumina (20%% EtOAc in P.E.) to afford the product as a viscous yellow oil (145 mg, 81%). IR ν_{max}/cm^{-1} (film): 2937, 2869, 1732, 1461, 1447, 1395, 1378, 1352, 1308, 1274, 1245, 1203, 1167, 1134, 1115, 1098, 1066, 1013; ^{1}H NMR (400 MHz, $CDCl_3$) δ: 7.99 (1 H, d, *J* = 8.3 Hz), 7.76 (2 H, d, *J* = 8.4 Hz), 7.49 (1 H, d, *J* = 7.8 Hz), 7.45 (1 H, s), 7.32 (1 H, app t, *J* = 7.7 Hz), 7.26 – 7.19 (3 H, m), 3.72 (2 H, td, *J* = 1.8, 6.1 Hz), 3.55 (1 H, ddd, *J* = 6.4, 9.1, 14.1 Hz), 3.45 – 3.35 (2 H, m), 3.11 – 2.95 (2 H, m), 2.33 (3 H, s), 2.30 – 2.18 (2 H, m), 1.90 (2 H, d, *J* = 7.2 Hz), 1.84 (2 H, t, *J* = 6.1 Hz), 1.08 – 0.96 (21 H, m); ^{13}C NMR (101 MHz, $CDCl_3$) δ: 171.6, 144.9, 135.5, 135.3, 130.7, 130.0, 127.0, 125.0, 123.4, 123.3, 119.7, 119.4, 113.9, 63.0, 59.9, 52.7, 40.1, 35.9, 28.2, 24.6, 21.7, 18.1, 17.9, 12.0; m/z HRMS found $[M + H]^+$ 595.3009, $C_{33}H_{47}N_2O_4SSi$ requires 595.3020.

***cis*-8-methyl-3-tosyl-1-(2-((triisopropylsilyl)oxy)ethyl)-3,8-diazabicyclo[4.2.0]octan-7-one (2y)**

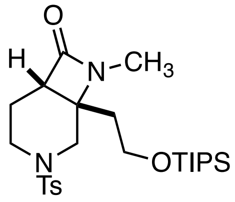

Prepared according to general procedure A using *N*-methyl-1-tosyl-3-(2-((triisopropylsilyl)oxy)ethyl)piperidin-3-amine (140.6 mg, 0.3 mmol), $Pd(OAc)_2$ (9.3 mg, 0.03 mmol), Xantphos (17 mg, 0.03 mmol), 1,4-benzoquinone (65 mg, 0.6 mmol) and silver(I) pivalate (189 mg, 0.9 mmol) in toluene (3 mL) at 80 ºC for 18 hours. The crude product was purified by flash column chromatography (gradient elution: 5% EtOAc in P.E. to 30% EtOAc in P.E.) and subsequently by flash column chromatography over alumina (gradient elution: 5% EtOAc in P.E. to 30% EtOAc in P.E.) to afford the product as a colourless oil (87.0 mg, 59%). IR ν_{max}/cm^{-1} (film): 2943, 2866, 1744, 1598, 1463, 1421, 1390, 1338, 1250, 1162, 1092. ^1H NMR (400 MHz, $CDCl_3$) δ: 7.65 (2 H, d, *J* = 8.3 Hz), 7.30 (2 H, *J* = 8.1 Hz), 3.81 – 3.76 (3 H, m), 3.49 (1 H, dt, *J* = 6.1, 11.6 Hz), 3.19 (1 H, t, *J* = 3.7 Hz), 3.13 (1 H, ddd, *J* = 2.3, 7.1, 10.8 Hz), 3.00 (1 H, d, *J* = 13.6 Hz), 2.67 (3 H, s), 2.41 (3 H, s), 2.06 (1 H, dqt, 3.1, 14.9 Hz), 1.92 – 1.69 (3 H, m), 1.03 – 0.99 (21 H, m). ^{13}C NMR (101 MHz, $CDCl_3$) δ: 168.2, 143.6, 134.9, 129.9, 127.3, 59.4, 59.3, 50.6, 44.6, 40.9, 36.8, 24.4, 21.6, 19.5, 18.1, 11.9. m/z HRMS found $[M + NH_4]^+$ 512.2964, $C_{25}H_{46}O_4N_3SSi$ requires 512.2973.

***cis*-(2-methyl-1-oxo-1,3,4,8b-tetrahydronaphtho[2,1-*b*]azet-2a(2*H*)-yl)methyl pivalate (2z)**

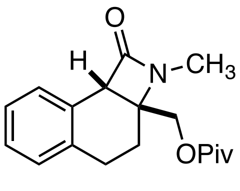

Prepared according to general procedure A using (2-(methylamino)-1,2,3,4-tetrahydronaphthalen-2-yl)methyl pivalate (82.6 mg, 0.3 mmol), $Pd(OAc)_2$ (6.7 mg, 0.03 mmol), Xantphos (17 mg, 0.03 mmol), 1,4-benzoquinone (65 mg, 0.6 mmol) and silver(I) acetate (150 mg, 0.9 mmol) in toluene (3 mL) at 80 ºC for 18 hours. Purification by flash column chromatography (30% EtOAc in P.E.) and subsequently by flash column chromatography over alumina (30% EtOAc in P.E.) gave the desired product as a colorless oil (77.0 mg, 86%). IR ν_{max}/cm^{-1} (film): 2971, 1746, 1729, 1480, 1458, 1419, 1387, 1366, 1281, 1253, 1226, 1208, 1143, 1052, 1036. ^1H NMR (400 MHz, $CDCl_3$) δ: 7.22 – 7.14 (4 H, m), 4.59 (1 H, d, *J* = 12.1 Hz), 4.08 – 4.05 (2 H, m), 2.78 (3 H, s), 2.75 – 2.61 (2 H, m), 2.19 (1 H,

dt, J = 3.5, 14.1 Hz), 1.48 (1 H, td, J = 4.3, 13.5 Hz), 1.25 (9 H, s). ^{13}C NMR (101 MHz, $CDCl_3$) δ: 178.0, 166.0, 137.5, 130.2, 129.5, 28.3, 127.5, 127.2, 65.4, 61.3, 56.2, 39.1, 27.3, 25.4, 25.2, 24.2. m/z HRMS found $[M + H]^+$ 302.1750, $C_{18}H_{24}O_3N$ requires 302.1751

(*cis* -7-benzyl-8-oxo-7-azabicyclo[4.2.0]octan-6-yl)methyl pivalate (2aa)

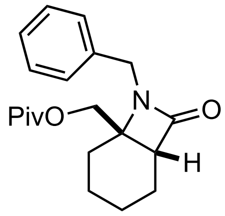

Prepared according to general procedure A using (1-(benzylamino)cyclohexyl)methyl pivalate (91.0 mg, 0.3 mmol), $Pd(OAc)_2$ (6.7 mg, 0.03 mmol), Xantphos (17 mg, 0.03 mmol), 1,4-benzoquinone (65 mg, 0.6 mmol) and silver(I) acetate (150 mg, 0.9 mmol) in toluene (3 mL) at 80 ºC for 18 hours. The crude mixture was purified by flash column chromatography (gradient elution, 10% EtOAc in P.E. to 30% EtOAc in P.E.) and subsequently by flash column chromatography over alumina (gradient elution, 10% EtOAc in P.E. to 30% EtOAc in P.E.) to afford (*cis* -7-benzyl-8-oxo-7-azabicyclo[4.2.0]octan-6-yl)methyl pivalate (56 mg, 57%). m.p. 74–76 °C; IR ν_{max}/cm^{-1} (film): 2933, 2877, 1719, 1498, 1479, 1467, 1451, 1435, 1403, 1358, 1336, 1284, 1199, 1163, 1054, 1033; ^{1}H NMR (400 MHz, $CDCl_3$) δ: 7.34 – 7.24 (5 H, m), 4.29 (2 H, q, J = 15.2 Hz), 4.10 (1 H, d, J = 12.0 Hz), 3.92 (1 H, d, J = 11.9 Hz) 3.06 (1 H, dd, J = 3.8, 6.4 Hz), 1.91 (1 H, ddd, J = 2.6, 5.3, 8.6 Hz), 1.71 – 1.54 (3 H, m), 1.51 – 1.34 (3 H, m), 1.32 – 1.20 (1 H, m), 1.16 (9 H, s); ^{13}C NMR (101 MHz, $CDCl_3$) δ: 178.1, 170.0, 136.8, 128.8, 128.6, 127.8, 66.2, 59.8, 50.1, 43.9, 39.1, 27.3, 25.1, 19.7, 18.7, 17.1; m/z HRMS found $[M + H]^+$ 330.2064, $C_{20}H_{28}NO_3$ requires 330.2064.

(1-(1-oxoisoindolin-2-yl)cyclohexyl)methyl pivalate (3aa)

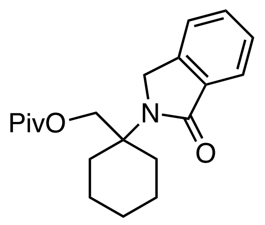

Prepared according to general procedure A using (1-(benzylamino)cyclohexyl)methyl pivalate (91.0 mg, 0.3 mmol), $Pd(OAc)_2$ (6.7 mg, 0.03 mmol), Xantphos (17 mg, 0.03 mmol), 1,4-benzoquinone (65 mg, 0.6 mmol) and silver(I) acetate (150 mg, 0.9 mmol) in toluene (3 mL) at 80 ºC for 18 hours. The crude mixture was purified by flash column chromatography (gradient elution, 10% EtOAc in P.E. to 30% EtOAc in P.E.) and subsequently by flash column chromatography over alumina (gradient elution, 10% EtOAc in P.E. to 30% EtOAc in P.E.) to afford (1-(1-oxoisoindolin-2-yl)cyclohexyl)methyl pivalate (23 mg, 23%) as a white solid. m.p. 86–88 °C; IR ν_{max}/cm^{-1} (film): 2936, 2863, 1723, 1671, 1479, 1469, 1443, 1388, 1366, 1327, 1301, 1281, 1138, 1071, 1034; ^1H NMR (400 MHz, $CDCl_3$) δ: 7.79 (1 H, d, *J* = 7.5 Hz), 7.52 (1 H, td, *J* = 1.1, 7.4 Hz), 7.46 – 7.40 (2 H, m), 4.57 (2 H, s), 4.39 (2 H, s), 2.60 – 2.52 (2 H, m), 1.82 – 1.73 (2 H, m), 1.72 – 1.51 (6 H, m), 1.10 (9 H, s); ^{13}C NMR (101 MHz, $CDCl_3$) δ: 178.3, 169.7, 141.0, 134.1, 131.3, 128.0, 123.5, 122.3, 67.7, 59.5, 49.2, 39.0, 31.9, 27.3, 25.8, 22.3; m/z HRMS found $[M + H]^+$ 330.2063, $C_{20}H_{28}NO_3$ requires 330.2064.

***cis*-7-(4-methylbenzyl)-8-oxo-7-azabicyclo[4.2.0]octan-6-yl)methyl pivalate (2ab)**

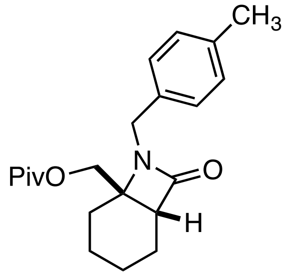

Prepared according to general procedure A using (1-((4-methylbenzyl)amino)cyclohexyl)methyl pivalate (95.2 mg, 0.3 mmol), $Pd(OAc)_2$ (9.3 mg, 0.03 mmol), Xantphos (17 mg, 0.03 mmol), 1,4-benzoquinone (65 mg, 0.6 mmol) and silver(I) acetate (150 mg, 0.9 mmol) in toluene (3 mL) at 80 ºC for 18 hours. The crude product was purified by flash column chromatography (gradient elution: 100% P.E. to 25% EtOAc in P.E.) and subsequently by flash column chromatography over alumina

(gradient elution: 100 % P.E. to 20% EtOAc in P.E.) to afford the product as a colorless oil (32.7 mg, 32%). IR ν_{max}/cm^{-1} (film): 2936, 2871, 1728, 1516, 1480, 1458, 1397, 1365, 1346, 1281, 1145, 1035. ^{1}H NMR (400 MHz, $CDCl_3$) δ: 7.20 (2 H, d, *J* = 8.0 Hz), 7.10 (2 H, d, *J* = 8.0 Hz), 4.29 (1 H, d, *J* = 15.1 Hz), 4.20 (1 H, d, *J* = 15.1 Hz), 4.08 (1 H, d, *J* = 11.7 Hz), 3.92 (1 H, d, *J* = 11.71 Hz), 3.06 – 3.04 (1 H, m), 2.31 (3 H, s), 1.93 – 1.88 (1 H, m), 1.67 – 1.56 (3 H, m), 1.49 – 1.38 (3 H, m), 1.29 – 1.23 (1 H, m), 1.17 (9 H, s). ^{13}C NMR (101 MHz, $CDCl_3$) δ: 178.1, 170.0, 137.5, 133.7, 129.5, 128.5, 66.1, 59.8, 50.0, 43.6, 39.0, 27.3, 25.1, 21.2, 19.6, 18.7, 17.1. m/z HRMS found $[M + H]^+$ 344.2222, $C_{21}H_{30}NO_3$ requires 344.2220

(1-(6-methyl-1-oxoisoindolin-2-yl)cyclohexyl)methyl pivalate (3ab)

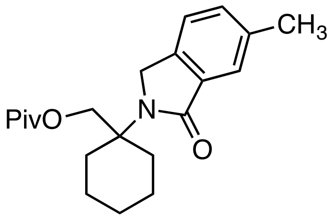

Prepared according to general procedure A using (1-((4-methylbenzyl)amino)cyclohexyl)methyl pivalate (95.2 mg, 0.3 mmol), $Pd(OAc)_2$ (9.3 mg, 0.03 mmol), Xantphos (17 mg, 0.03 mmol), 1,4-benzoquinone (65 mg, 0.6 mmol) and silver(I) acetate (150 mg, 0.9 mmol) in toluene (3 mL) at 80 ºC for 18 hours. The crude product was purified by flash column chromatography (gradient elution: 100% P.E. to 25% EtOAc in P.E.) and subsequently by flash column chromatography over alumina (gradient elution: 100 % P.E. to 20% EtOAc in P.E.) to afford the product as a colorless oil (51.7 mg, 51%). IR ν_{max}/cm^{-1} (film): 2933, 2866, 1727, 1680, 1628, 1497, 1480, 1449, 1385, 1321, 1281, 1229, 1194, 1149, 1035. ^{1}H NMR (400 MHz, $CDCl_3$) δ: 7.58 (1 H, s), 7.34 – 7.26 (2 H, m), 4.52 (2 H, s), 4.38 (2 H, s), 2.55 – 2.52 (2 H, m), 2.43 (3 H, s), 1.78 – 1.73 (2 H, m), 1.68 – 1.42 (6 H, m), 1.10 (9 H, s). ^{13}C NMR (101 MHz, $CDCl_3$) δ: 178.3, 169.8, 138.3, 138.0, 134.2, 132.4, 123.7, 122.0, 67.7, 59.4, 49.0, 39.0, 31.9, 27.3, 25.8, 22.3, 21.5. m/z HRMS found $[M + H]^+$ 344.2221, $C_{21}H_{30}NO_3$ requires 344.2220

***cis*-7-(4-chlorobenzyl)-8-oxo-7-azabicyclo[4.2.0]octan-6-yl)methyl pivalate (2ac)**

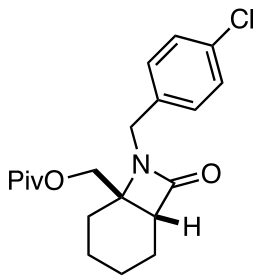

Prepared according to general procedure A using (1-((4-chlorobenzyl)amino)cyclohexyl)methyl pivalate (101.2 mg, 0.3 mmol), $Pd(OAc)_2$ (9.3 mg, 0.03 mmol), Xantphos (17 mg, 0.03 mmol), 1,4-benzoquinone (65 mg, 0.6 mmol) and silver(I) acetate (150 mg, 0.9 mmol) in toluene (3 mL) at 80 ºC for 18 hours. The crude product was purified by flash column chromatography (gradient elution: 100% P.E. to 25% EtOAc in P.E.) and subsequently by flash column chromatography over alumina (gradient elution: 100 % P.E. to 20% EtOAc in P.E.) to afford the product as a colorless oil (56.3 mg, 52%). IR ν_{max}/cm^{-1} (film): 2936, 2871, 1730, 1492, 1480, 1458, 1396, 1365, 1281,1146, 1092, 1035. ^1H NMR (400 MHz, $CDCl_3$) δ: 7.30 – 7.25 (4 H, m), 4.29 (1 H, d, J = 15.3 Hz), 4.18 (1 H, d, J = 15.3 Hz), 4.15 (1 H, d, J = 11.8 Hz), 3.93 (1 H, d, J = 11.8 Hz), 3.07 (1 H, dd, J = 3.5, 6.3 Hz), 1.93 – 1.86 (1 H, m), 1.69 – 1.55 (3 H, m), 1.51 – 1.39 (3 H, m), 1.26 – 1.19 (1 H, m), 1.16 (9 H, s). ^{13}C NMR (101 MHz, $CDCl_3$) δ: 178.1, 170.1, 135.5, 133.7, 129.8, 129.0, 66.0, 59.9, 50.0, 43.2, 39.0, 27.2, 25.1, 19.6, 18.7, 17.1. m/z HRMS found $[M + H]^+$ 364.1677, $C_{20}H_{27}ClNO_3$ requires 364.1674

(1-(6-chloro-1-oxoisoindolin-2-yl)cyclohexyl)methyl pivalate (3ac)

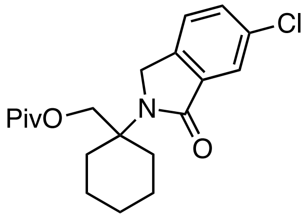

Prepared according to general procedure A using (1-((4-chlorobenzyl)amino)cyclohexyl)methyl pivalate (101.2 mg, 0.3 mmol), $Pd(OAc)_2$ (9.3 mg, 0.03 mmol), Xantphos (17 mg, 0.03 mmol), 1,4-benzoquinone (65 mg, 0.6 mmol) and silver(I) acetate (150 mg, 0.9 mmol) in toluene (3 mL) at 80 ºC for 18 hours. The crude product was purified by flash column chromatography (gradient elution: 100% P.E. to 25% EtOAc in P.E.) and subsequently by flash column chromatography over alumina (gradient elution: 100 % P.E. to 20% EtOAc in P.E.) to afford the product as a colorless needles (13.0 mg, 12%). m.p. 112 ºC (sharp). IR ν_{max}/cm^{-1} (film): 2934, 2867, 1729, 1687, 1449, 1386, 1318, 1282,

1263, 1205, 1152, 1036. ^{1}H NMR (400 MHz, $CDCl_3$) δ: 7.75 (1 H, d, *J* = 1.8 Hz), 7.49 (1 H, dd, *J* = 1.8, 8.0 Hz), 7.35 (1 H, d, *J* = 8.0 Hz), 4.54 (2 H, s), 4.38 (2 H, s), 2.51 (2 H, m), 1.77 (2 H, ddd, *J* = 3.0, 9.5, 12.6 Hz), 1.66 – 1.60 (3 H, m), 1.55 – 1.42 (3 H, m), 1.10 (9 H, s). ^{13}C NMR (101 MHz, $CDCl_3$) δ:178.2, 168.3, 139.1, 135.8, 134.4, 131.6, 123.7, 123.7, 67.6. 59.8, 48.8, 39.0, 31.9, 27.3, 25.7, 22.3. m/z HRMS found $[M + H]^+$ 364.1674, $C_{20}H_{27}ClNO_3$ requires 364.1677.

cis-7-(3-nitrobenzyl)-8-oxo-7-azabicyclo[4.2.0]octan-6-yl)methyl pivalate (2ad)

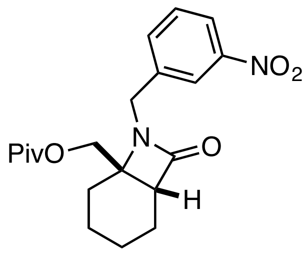

Prepared according to general procedure A using (1-((3-nitrobenzyl)amino)cyclohexyl)methyl pivalate (104.5 mg, 0.3 mmol), $Pd(OAc)_2$ (9.3 mg, 0.03 mmol), Xantphos (17 mg, 0.03 mmol), 1,4-benzoquinone (65 mg, 0.6 mmol) and silver(I) acetate (150 mg, 0.9 mmol) in toluene (3 mL) at 80 ºC for 18 hours. The crude product was purified by flash column chromatography (gradient elution: 100% P.E. to 35% EtOAc in P.E.) to afford the product as a pale yellow oil (52.1 mg, 46%). IR ν_{max}/cm^{-1} (film): 2937, 2871, 1728, 1529, 1480, 1461, 1397, 1348, 1281, 1145, 1097, 1035. ^{1}H NMR (400 MHz, $CDCl_3$) δ: 8.18 – 8.13 (2 H, m), 7.73 (1 H, d, *J* = 7.8 Hz), 7.52 (1 H, t, *J* = 7.3 Hz), 4.40 (1 H, d, *J* = 15.5 Hz), 4.34 (1 H, d, *J* = 15.1 Hz), 4.21 (1 H, d, *J* = 11.9 Hz), 3.96 (1 H, d, *J* = 11.9 Hz), 3.10 (1 H, dd, *J* = 3.7, 6.7 Hz), 1.95 – 1.88 (1 H, m), 1.72 – 1.43 (6 H, m), 1.30 – 1.22 (1 H, m), 1.14 (9 H, s). ^{13}C NMR (101 MHz, $CDCl_3$) δ: 178.0, 170.4, 148.5, 139.2, 134.5, 130.0 123.0, 122.9, 66.1, 60.2, 50.1, 43.2, 39.0, 27.2, 25.2, 19.6, 18.7, 17.2. m/z HRMS found $[M + H]^+$ 375.1916, $C_{20}H_{27}N_2O_5$ requires 375.1914

Functionalization of β-lactam products

***cis*-1-allyl-6-(((triisopropylsilyl)oxy)methyl)-7-methyl-7-azabicyclo[4.2.0]octan-8-one (4a)**

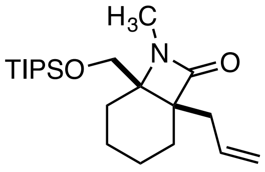

To a pre-cooled solution of *cis*-7-methyl-6-(((triisopropylsilyl)oxy)methyl)-7-azabicyclo[4.2.0]octan-8-one (97.6 mg, 0.3 mmol) in anhydrous THF (3 mL) was added a solution of freshly prepared LiHMDS (0.61 M, 0.75 mmol, 1.23 mL) dropwise at –78 °C (dry ice/acetone bath) under N_2. The solution was stirred at –78 °C for 2 hours and allyl bromide (104 μL, 1.2 mmol) was added in one portion. The solution was warmed to room temperature over 16 hours and was quenched with saturated aqueous NH_4Cl (3 mL). The organic layer was separated and the aqueous was extracted with diethyl ether (3 x 10 mL). The organics were combined, washed with brine, dried (Na_2SO_4) and the solvent removed *in vacuo*. The crude oil was purified by flash column chromatography (gradient elution: 5% EtOAc in P.E. to 25% EtOAc in P.E.) to afford the product as a colourless oil (86.8 mg, 79%). IR ν_{max}/cm^{-1} (film): 2942, 2866, 1744, 1640, 1463, 1417, 1383, 1100, 1060. ^{1}H NMR (400 MHz, $CDCl_3$) δ: 5.91 – 5.83 (1 H, m), 5.06 – 5.02 (2 H, m), 3.95 (1 H, d, *J* = 9.9 Hz), 3.83 (1 H, d, *J* = 9.9 Hz), 2.78 (3 H, s), 2.49 (1 H, app. dd, *J* = 6.6, 14.5 Hz), 2.32 (1 H, app. dd, *J* = 7.8, 14.5 Hz), 2.03 (1 H, app. dd, 5.9, 12.4 Hz), 1.70 (1 H, dt, *J* = 3.4, 12.7 Hz), 1.65 – 1.39 (6 H, m), 1.09 – 1.05 (21 H, m). ^{13}C NMR (101 MHz, $CDCl_3$) δ: 172.6, 134.5, 117.7, 67.3, 64.3, 57.7, 35.5, 26.0, 25.6, 23.4, 18.1, 17.8, 16.3, 12.0. m/z HRMS found $[M + H]^+$ 366.2824, $C_{21}H_{40}NO_2Si$ requires 366.2823.

***cis*-7-methyl-7-azabicyclo[4.2.0]octan-6-yl)methanol (4b)**

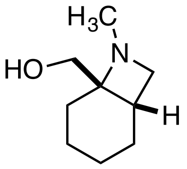

To a solution of $AlCl_3$ (120 mg, 0.9 mmol) in anhydrous Et_2O (2 mL) was added $LiAlH_4$ (34 mg, 0.9 mmol) carefully at 0 °C under N_2. The reaction mixture was stirred at 0 °C for 10 min and subsequently refluxed for 30 min. (±)-*cis*-7-methyl-6-(((triisopropylsilyl)oxy)methyl)-7-azabicyclo[4.2.0]octan-8-one (97.6 mg, 0.3 mmol) in anhydrous Et_2O (2 mL) was added dropwise and refluxed for 4 h. The reaction was cooled to room temperature and quenched with 10% NaOH (1 mL).

The aqueous was extracted with Et_2O (3 x 50 mL), dried over Na_2SO_4 and the solvent removed *in vacuo* (*Volatile!*) to obtain the title compound as a colorless oil (41.9 mg, 90%). IR ν_{max}/cm^{-1} (film): 3366 (br), 2926, 2855, 1481, 1450, 1374, 1339, 1274, 1186, 1158, 1120, 1087, 1063. ^1H NMR (400 MHz, $CDCl_3$) δ: 3.33 (1 H, d, *J* = 11.3 Hz), 3.21 (1 H, dd *J* = 5.8, 7.3 Hz), 3.09 (1 H, d, *J* = 11.3 Hz), 2.79 (1 H, dd *J* = 5.8, 9.7 Hz), 2.74 – 2.69 (1 H, m), 2.11 (3 H, s), 1.89 (1 H, td, *J* = 4.2, 13.6 Hz), 1.70 – 1.65 (1 H, m), 1.59 – 1.54 (1 H, m), 1.51 – 1.47 (1 H, m), 1.43 – 1.32 (2 H, m), 1.25 – 1.23 (1 H, m), 1.04 – 1.96 (1 H, m). ^{13}C NMR (101 MHz, $CDCl_3$) δ: 67.8, 64.1, 54.2, 48.3, 35.1, 29.9, 23.8, 23.7, 22.2, 21.4. m/z HRMS found $[M + H]^+$ 156.1382, $C_9H_{18}NO$ requires 156.1383

(*cis* -8-oxo-7-azabicyclo[4.2.0]octan-6-yl)methyl pivalate (4c)

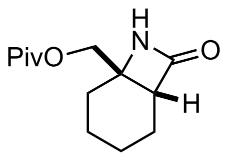

(*cis* -8-oxo-7-(2-(phenylsulfonyl)ethyl)-7-azabicyclo[4.2.0]octan-6-yl)methyl pivalate (123 mg, 0.3 mmol) was dissolved in THF (4.5 mL) and the solution cooled to -78 °C. Freshly prepared lithium *bis*(trimethylsilyl)amide (0.61 M in THF, 737 µL, 0.45 mmol) was added drop-wise and the reaction stirred at -78 °C for 30 minutes, then allowed to warm to room temperature over 30 minutes. The reaction mixture was quenched with water (5 mL) and extracted with EtOAc (3 x 10 mL). The combined organic layers were washed with brine (10 mL), dried over $MgSO_4$ and concentrated *in vacuo*. Purification of the residue by flash column chromatography (35% EtOAc in P.E.) afforded the title compound as a white solid (57 mg, 79%). m.p: 96–98 °C; IR ν_{max}/cm^{-1} (film): 3202 (br), 2937, 2865, 1740, 1719, 1479, 1451, 1395, 1312, 1286, 1157, 1082, 1039, 1017; ^1H NMR (400 MHz, $CDCl_3$) δ: 5.79 (1 H, br s, N-H), 4.32 (1 H, d, *J* = 11.6 Hz), 3.95 (1 H, d, *J* = 11.6 Hz), 3.09 – 2.97 (1 H, m), 1.96 – 1.85 (1 H, m), 1.80 – 1.63 (5 H, m), 1.60 – 1.44 (2 H, m), 1.21 (9 H, s); ^{13}C NMR (101 MHz, $CDCl_3$) δ: 178.5, 170.6, 67.9, 55.5, 50.1, 39.1, 27.8, 27.3, 20.0, 18.9, 17.5; m/z HRMS found $[M + H]^+$ 240.1595, $C_{13}H_{22}NO_3$ requires 240.1594.

NMR of Staring Amines

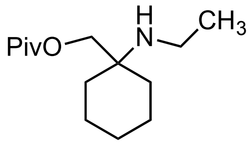
PivO
H
N
CH3

1a

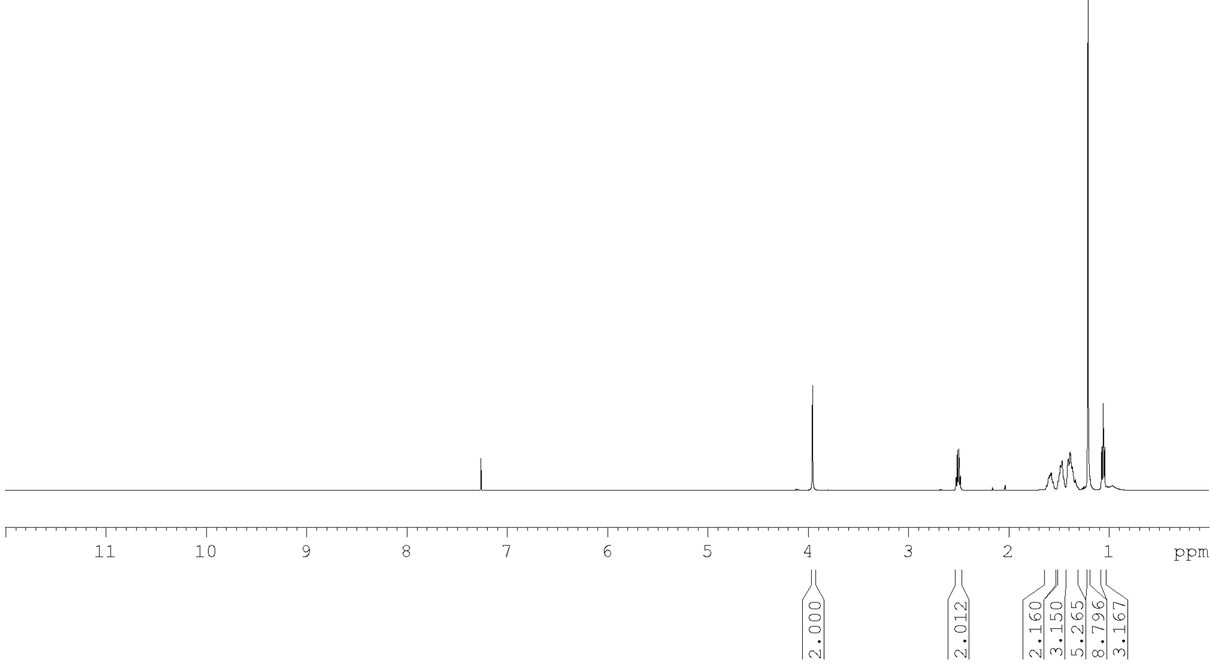
7.260
3.952
2.522
2.508
2.494
2.480
1.631
1.591
1.574
1.542
1.491
1.465
1.441
1.428
1.403
1.323
1.206
1.069
11 10 9 8 7 6 5 4 3 2 1 ppm
2.000
2.012
2.160
3.150
5.265
8.796
3.167

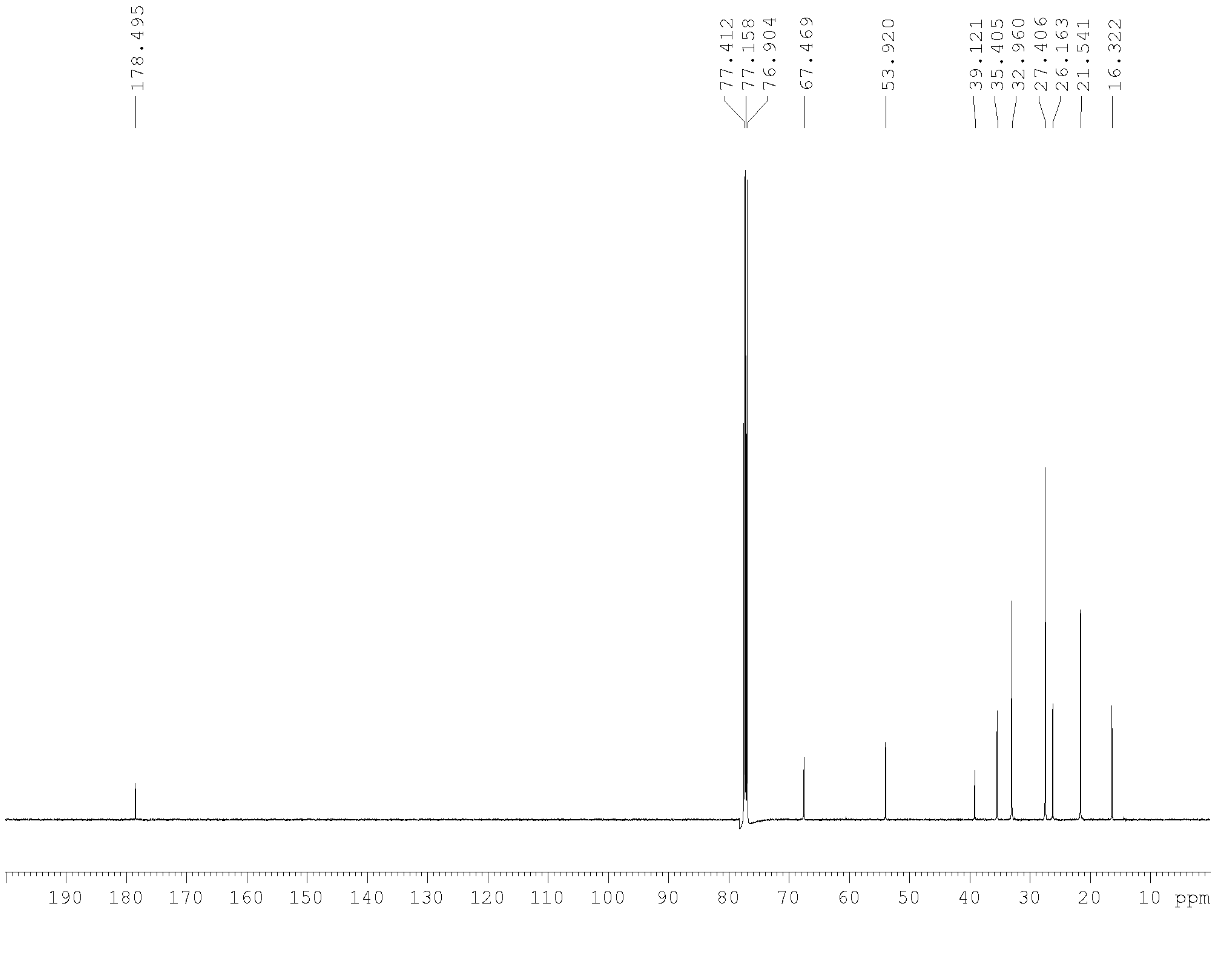
178.495
77.412
77.158
76.904
67.469
53.920
39.121
35.405
32.960
27.406
26.163
21.541
16.322
190 180 170 160 150 140 130 120 110 100 90 80 70 60 50 40 30 20 10 ppm

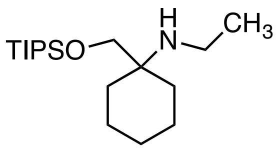
TIPSO
H
N
CH3

1b

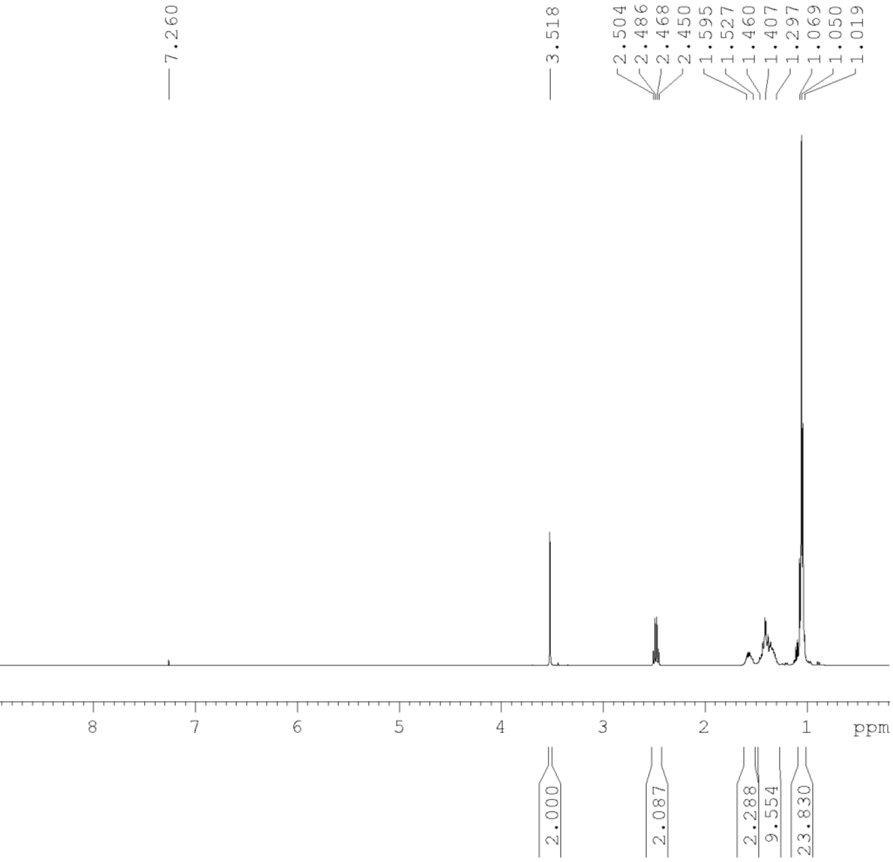
7.260
3.518
2.504
2.486
2.468
2.450
1.595
1.527
1.460
1.407
1.297
1.069
1.050
1.019
11 10 9 8 7 6 5 4 3 2 1 ppm
2.000
2.087
2.288
9.554
23.830

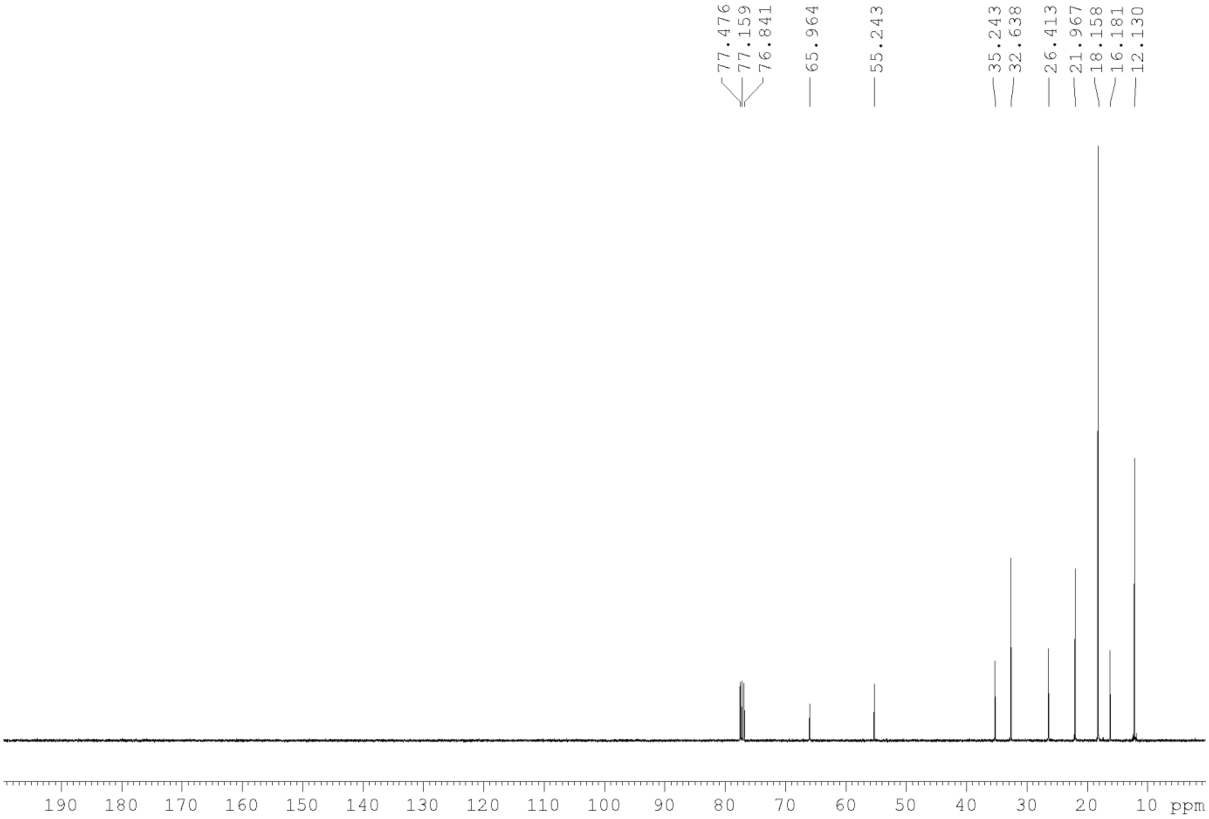
77.476
77.159
76.841
65.964
55.243
35.243
32.638
26.413
21.967
18.158
16.181
12.130
190 180 170 160 150 140 130 120 110 100 90 80 70 60 50 40 30 20 10 ppm

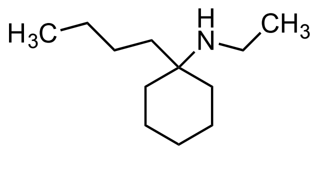
H3C
H
N
CH3

1c

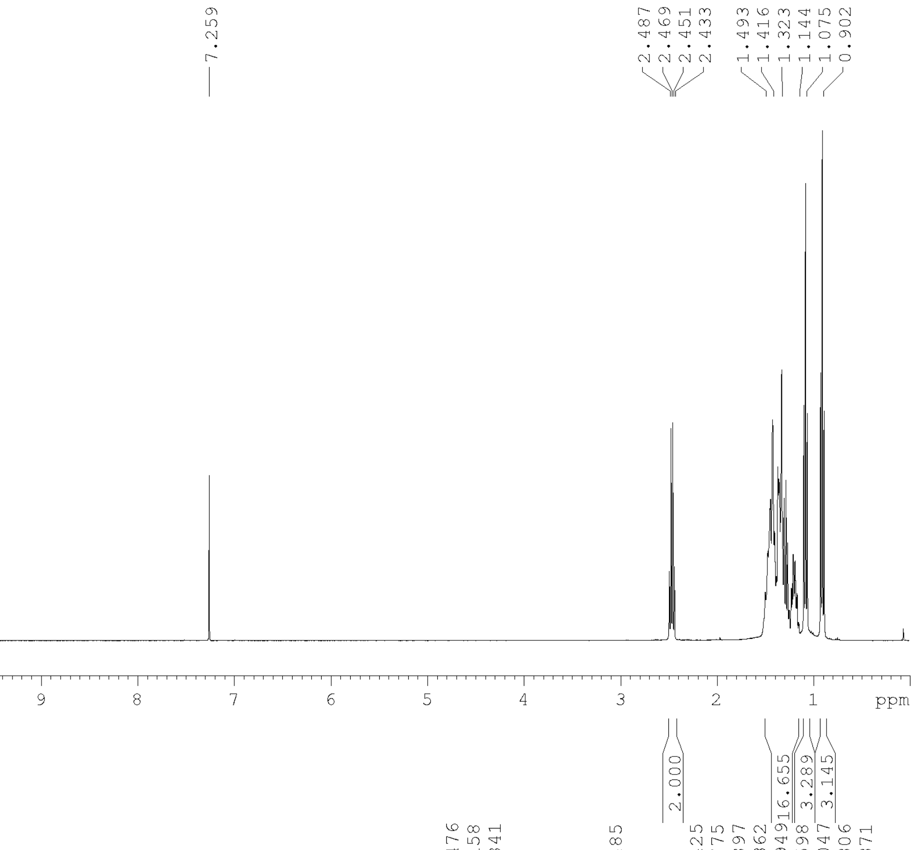
7.259
2.487
2.469
2.451
2.433
1.493
1.416
1.323
1.144
1.075
0.902
11 10 9 8 7 6 5 4 3 2 1 ppm
2.000
16.655
3.289
3.145

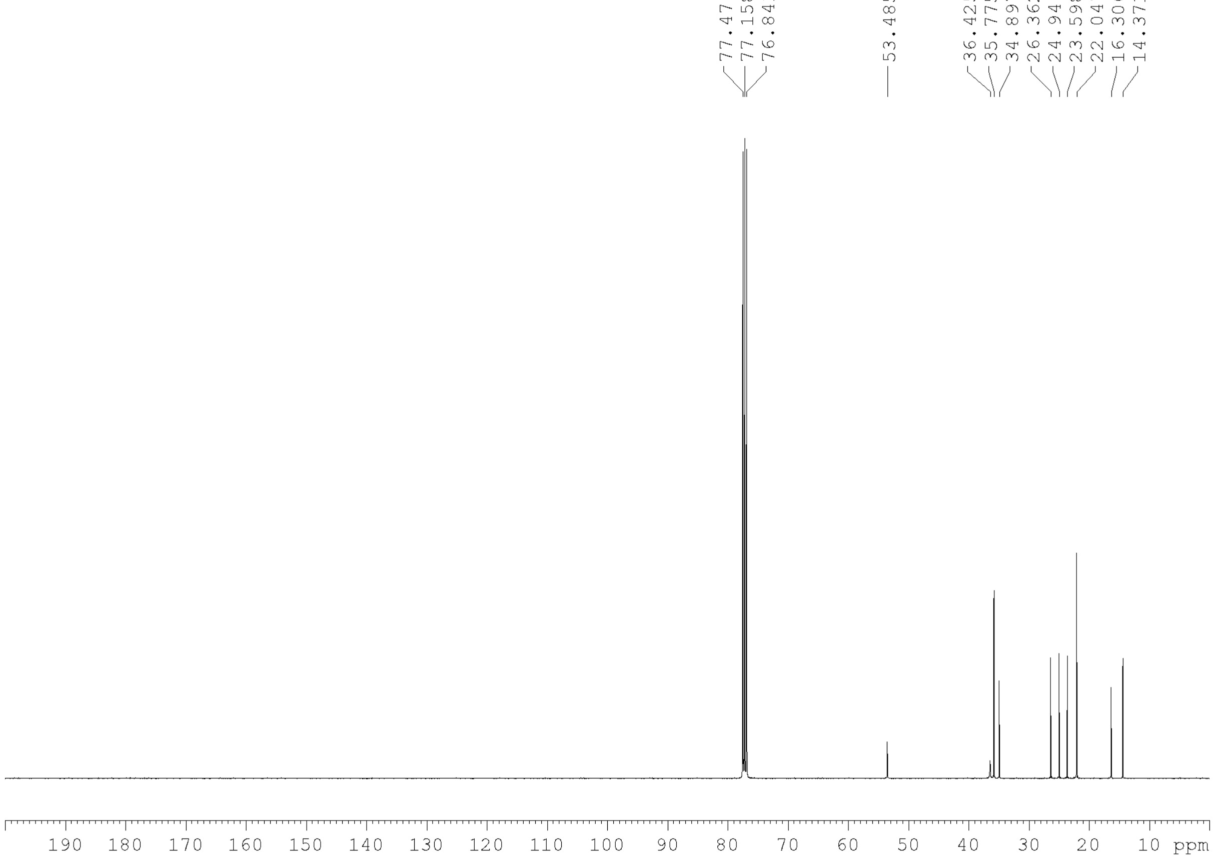
77.476
77.158
76.841
53.485
36.425
35.775
34.897
26.362
24.949
23.598
22.047
16.306
14.371
190 180 170 160 150 140 130 120 110 100 90 80 70 60 50 40 30 20 10 ppm

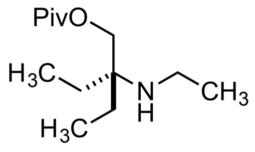
PivO
H_3C
N
H
CH_3
H_3C

1d

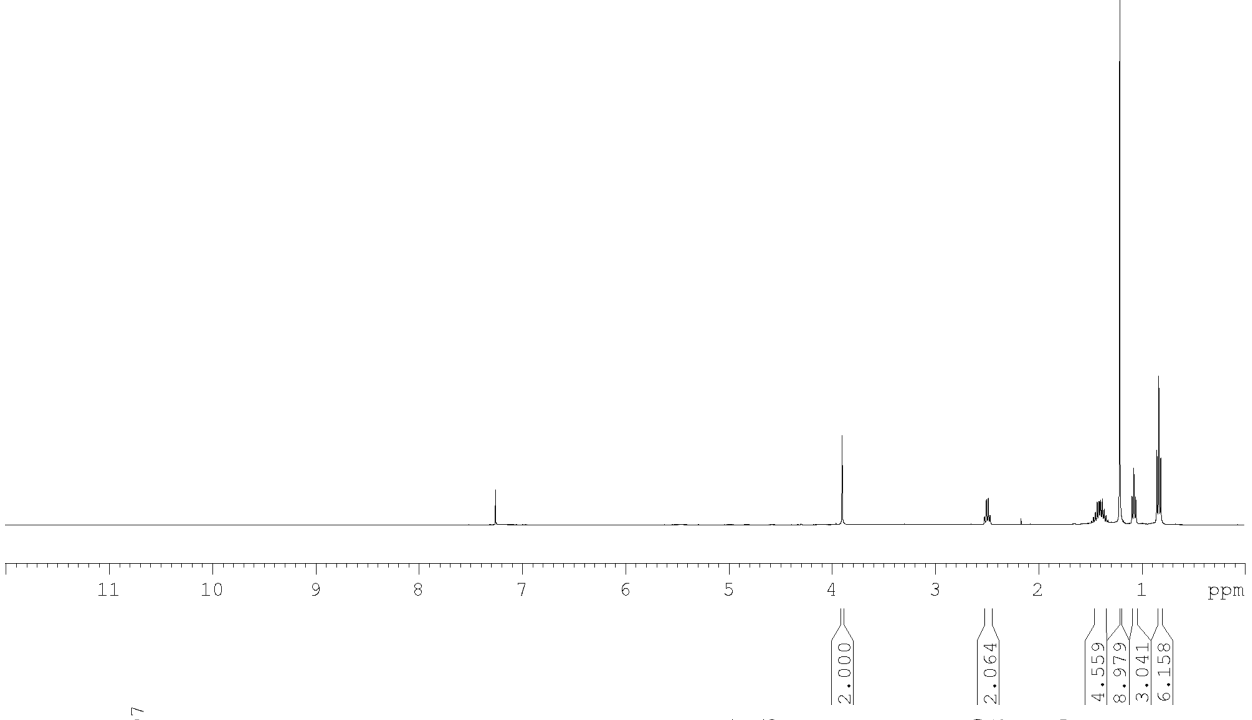
7.259
3.903
2.517
2.498
2.480
2.463
1.480
1.425
1.406
1.393
1.375
1.321
1.206
1.087
1.069
1.051
0.846
11 10 9 8 7 6 5 4 3 2 1 ppm
2.000
2.064
4.559
8.979
3.041
6.158

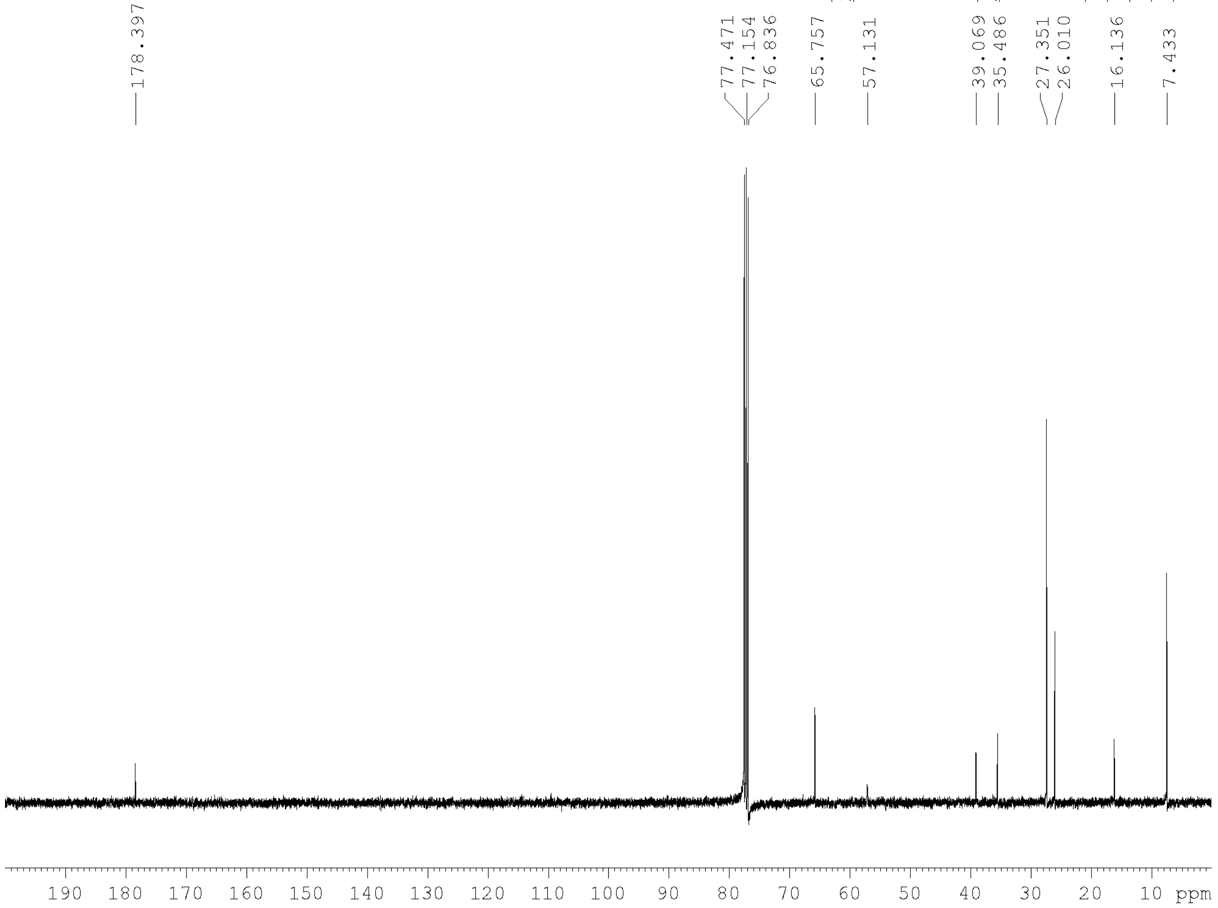
178.397
77.471
77.154
76.836
65.757
57.131
39.069
35.486
27.351
26.010
16.136
7.433
190 180 170 160 150 140 130 120 110 100 90 80 70 60 50 40 30 20 10 ppm

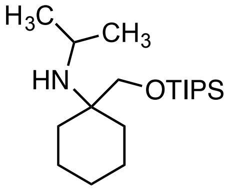
H3C
CH3
HN
OTIPS

1e

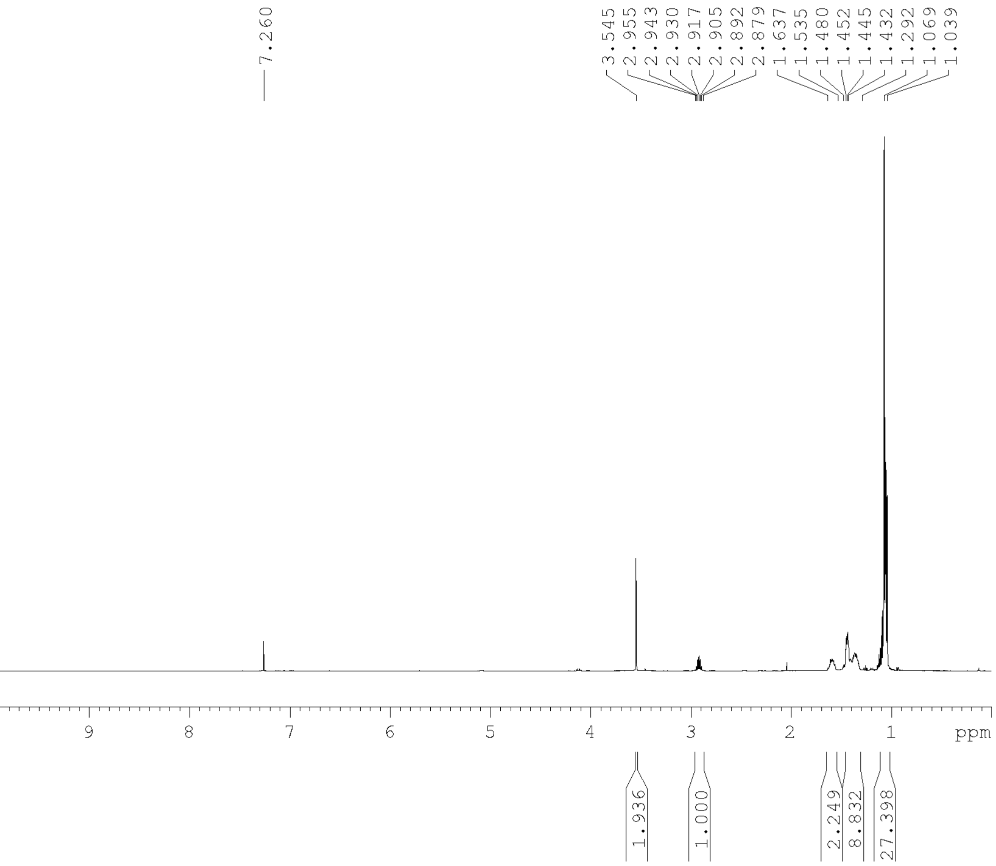
7.260
3.545
2.955
2.943
2.930
2.917
2.905
2.892
2.879
1.637
1.535
1.480
1.452
1.445
1.432
1.292
1.069
1.039
11 10 9 8 7 6 5 4 3 2 1 ppm
1.936
1.000
2.249
8.832
27.398

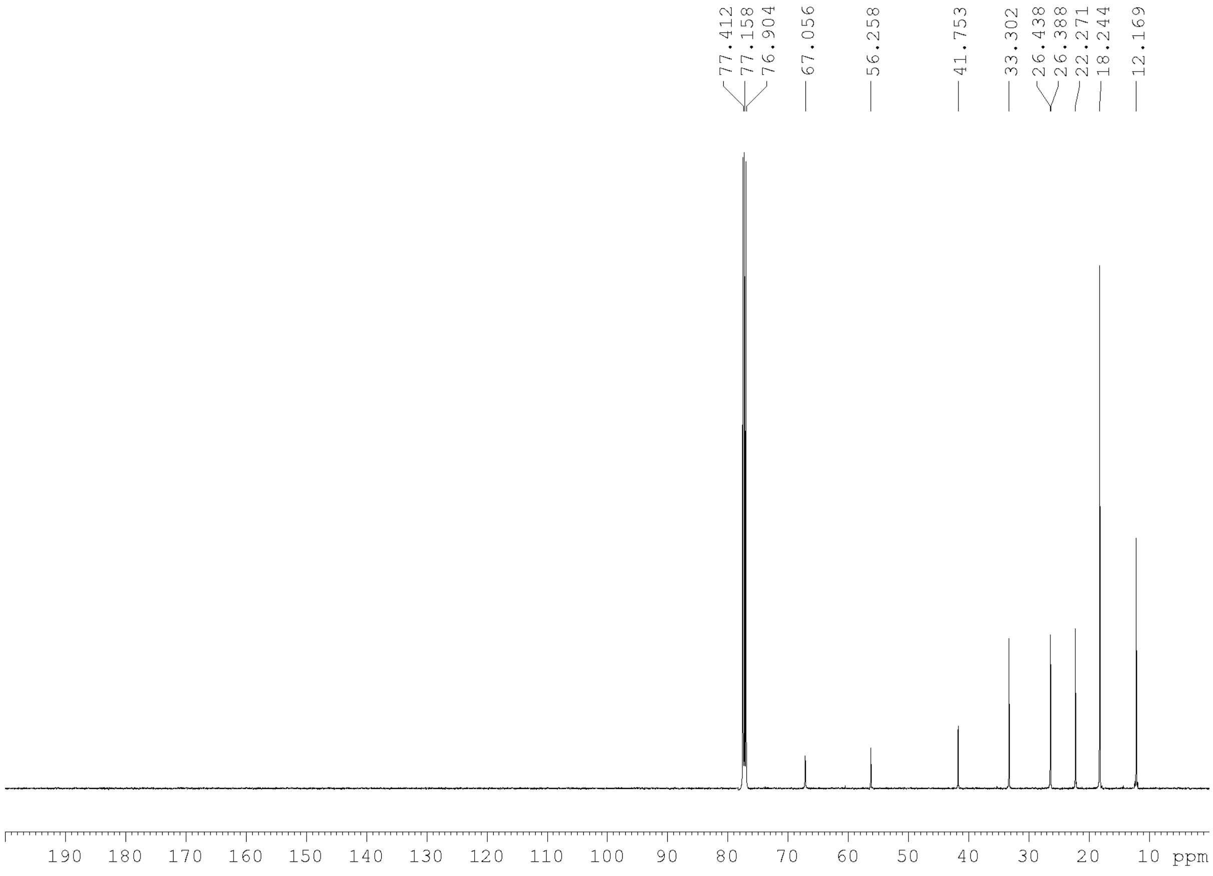
77.412
77.158
76.904
67.056
56.258
41.753
33.302
26.438
26.388
22.271
18.244
12.169
190 180 170 160 150 140 130 120 110 100 90 80 70 60 50 40 30 20 10 ppm

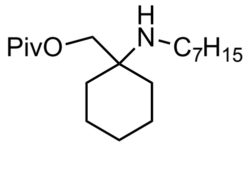
PivO
N
H
C_7H_{15}

1f

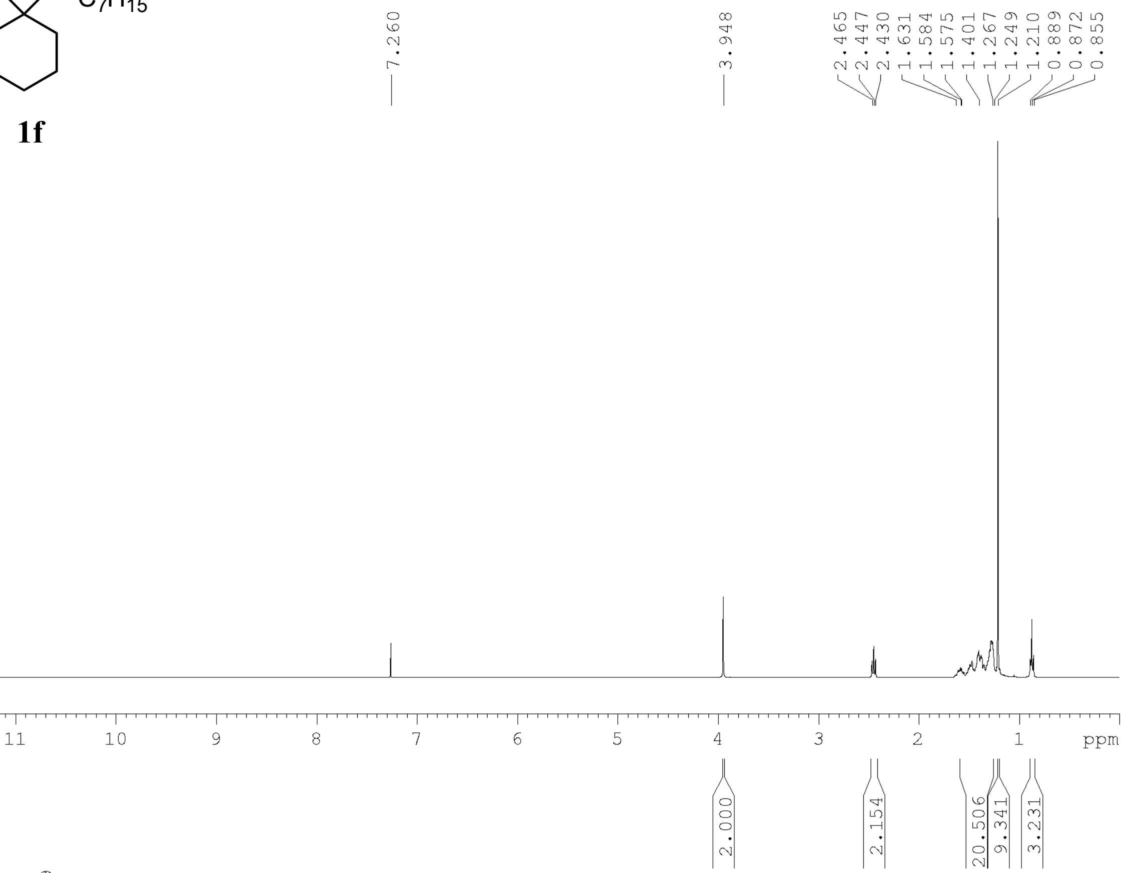
7.260
3.948
2.465
2.447
2.430
1.631
1.584
1.575
1.401
1.267
1.249
1.210
0.889
0.872
0.855
11 10 9 8 7 6 5 4 3 2 1 ppm
2.000
2.154
20.506
9.341
3.231

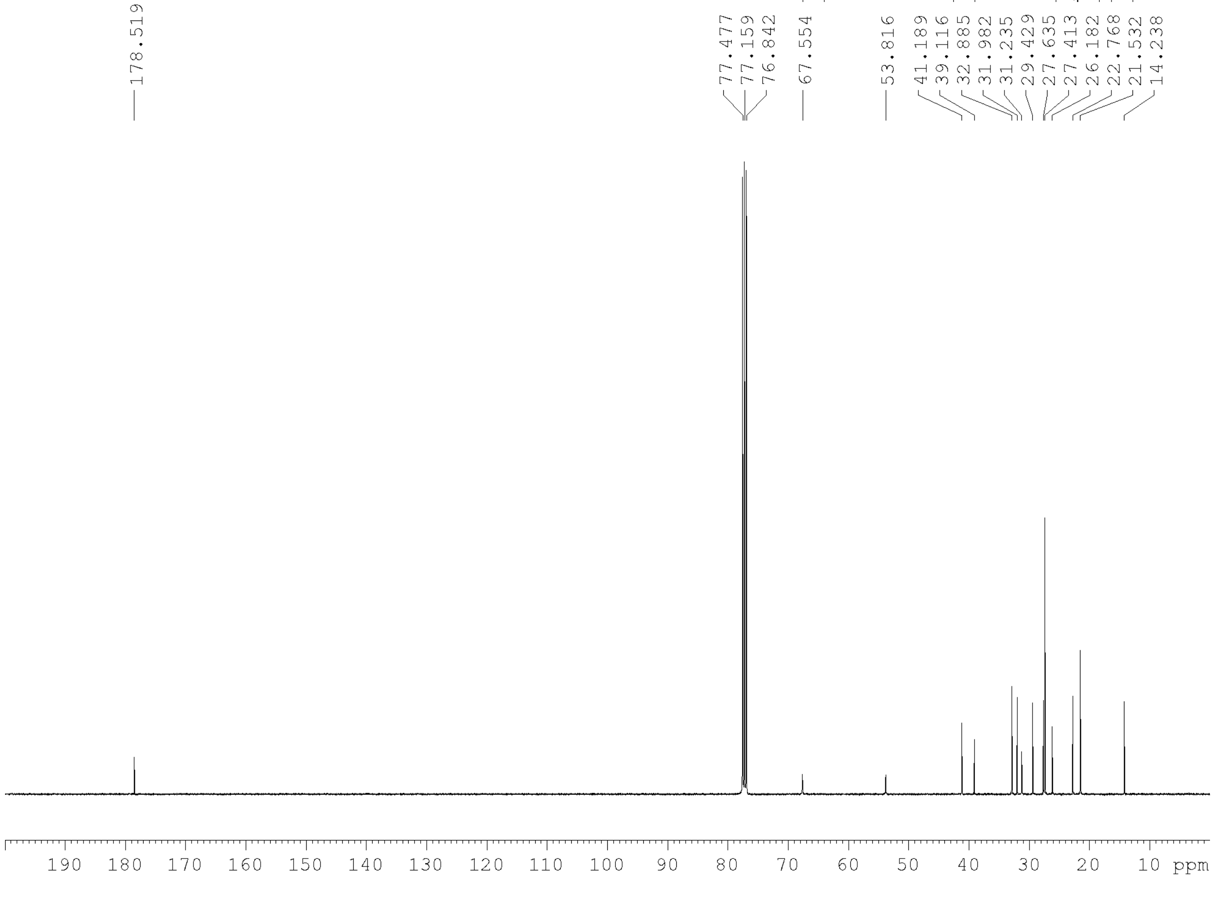
178.519
77.477
77.159
76.842
67.554
53.816
41.189
39.116
32.885
31.982
31.235
29.429
27.635
27.413
26.182
22.768
21.532
14.238
190 180 170 160 150 140 130 120 110 100 90 80 70 60 50 40 30 20 10 ppm

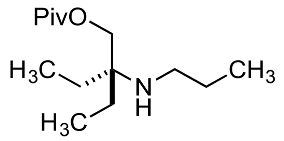
PivO
H3C
N
H
CH3
H3C

1g

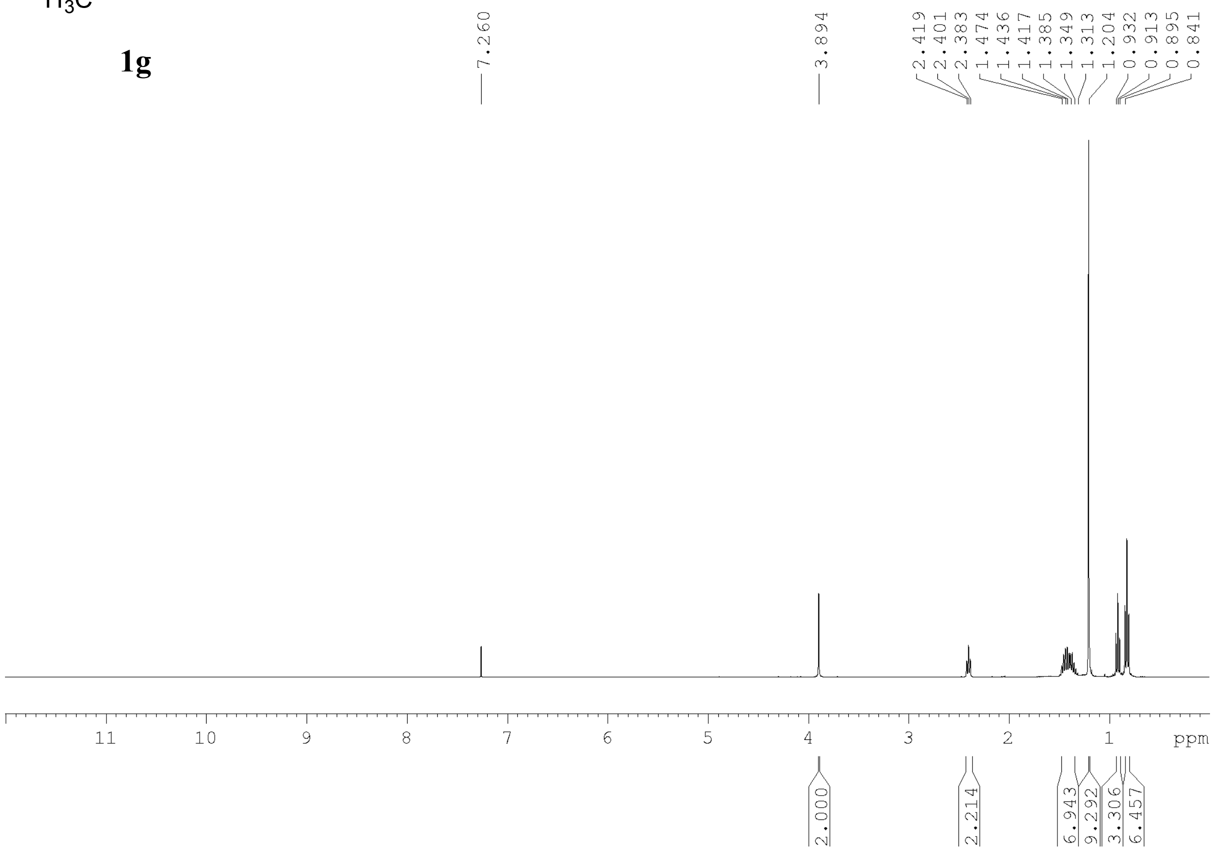
7.260
3.894
2.419
2.401
2.383
1.474
1.436
1.417
1.385
1.349
1.313
1.204
0.932
0.913
0.895
0.841
11 10 9 8 7 6 5 4 3 2 1 ppm
2.000
2.214
6.943
9.292
3.306
6.457

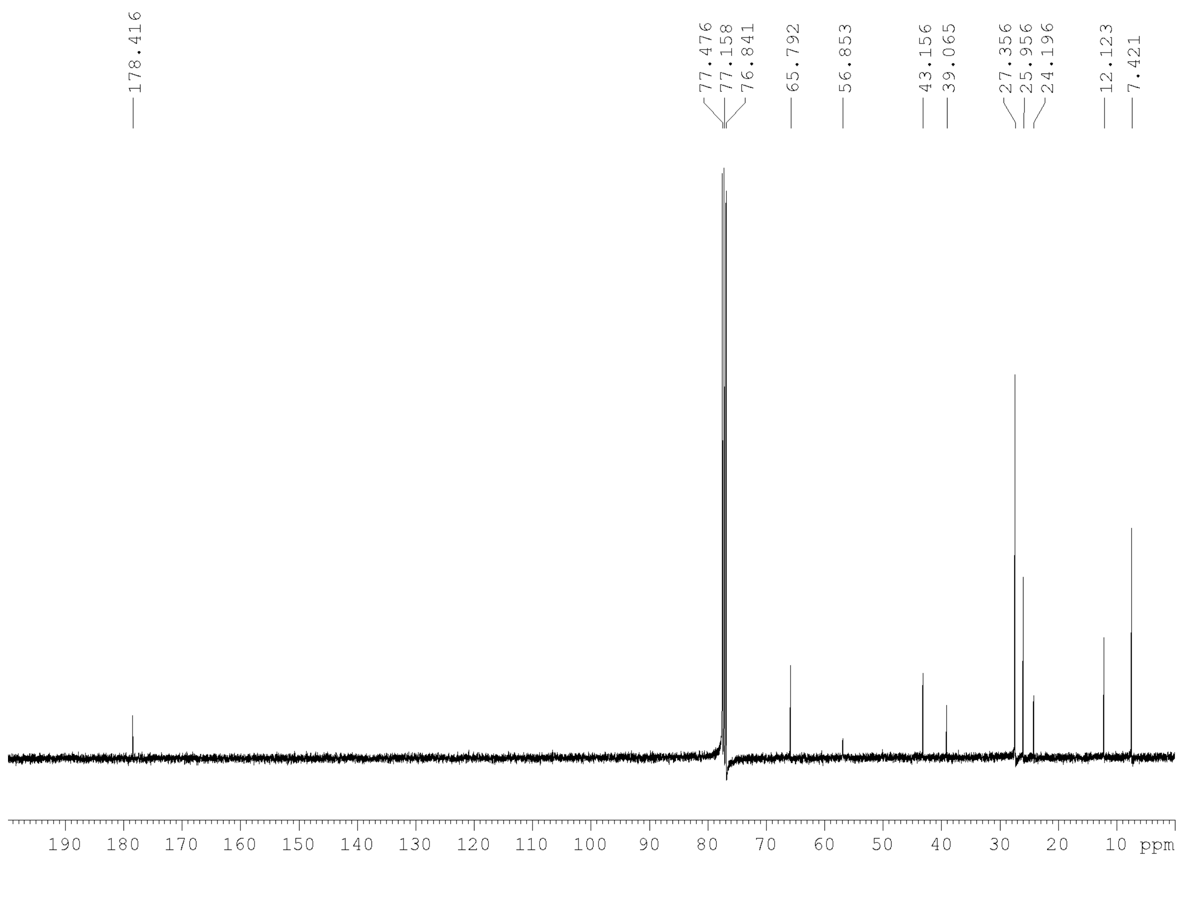
178.416
77.476
77.158
76.841
65.792
56.853
43.156
39.065
27.356
25.956
24.196
12.123
7.421
190 180 170 160 150 140 130 120 110 100 90 80 70 60 50 40 30 20 10 ppm

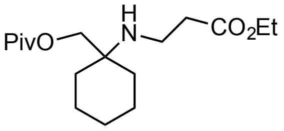
PivO
H
N
CO2Et

1h

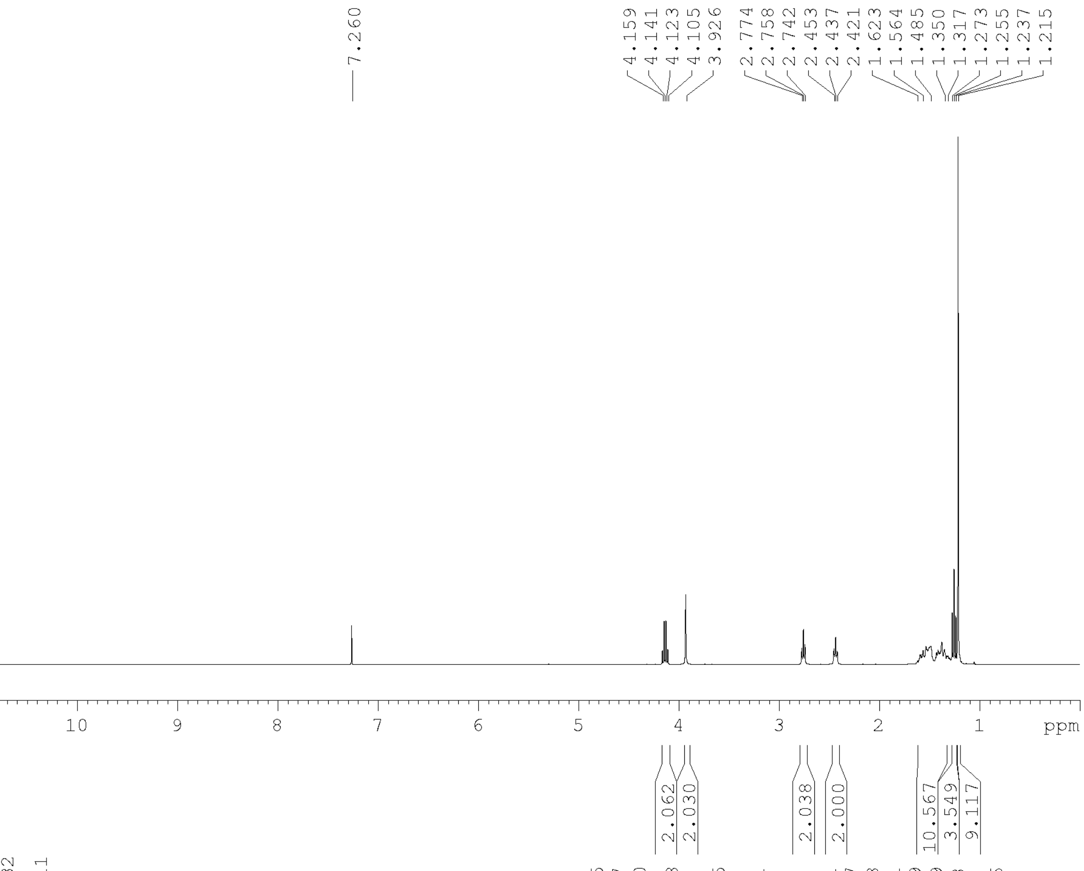
7.260
4.159
4.141
4.123
4.105
3.926
2.774
2.758
2.742
2.453
2.437
2.421
1.623
1.564
1.485
1.350
1.317
1.273
1.255
1.237
1.215
11 10 9 8 7 6 5 4 3 2 1 ppm
2.062
2.030
2.038
2.000
10.567
3.549
9.117

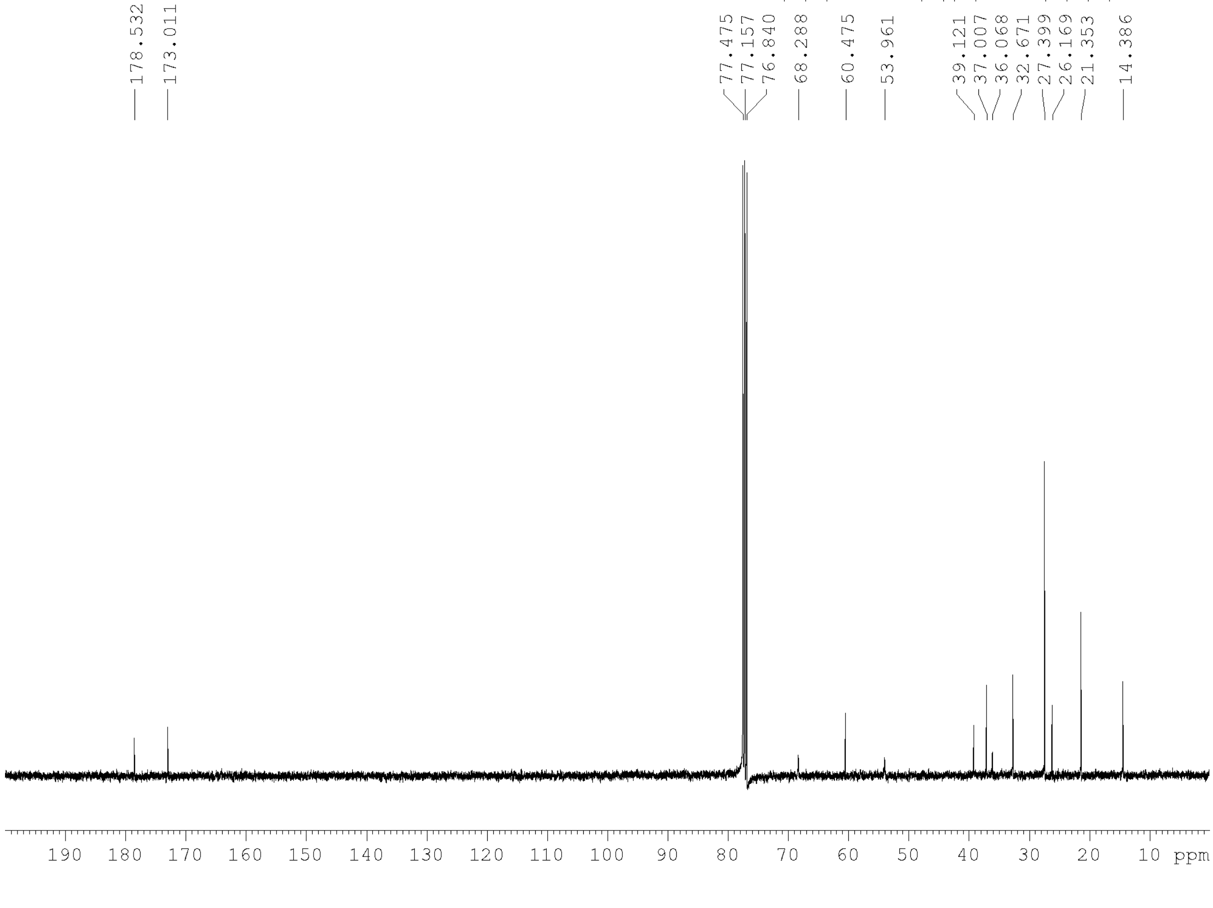
178.532
173.011
77.475
77.157
76.840
68.288
60.475
53.961
39.121
37.007
36.068
32.671
27.399
26.169
21.353
14.386
190 180 170 160 150 140 130 120 110 100 90 80 70 60 50 40 30 20 10 ppm

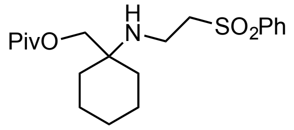

PivO
H
N
SO2Ph

1i

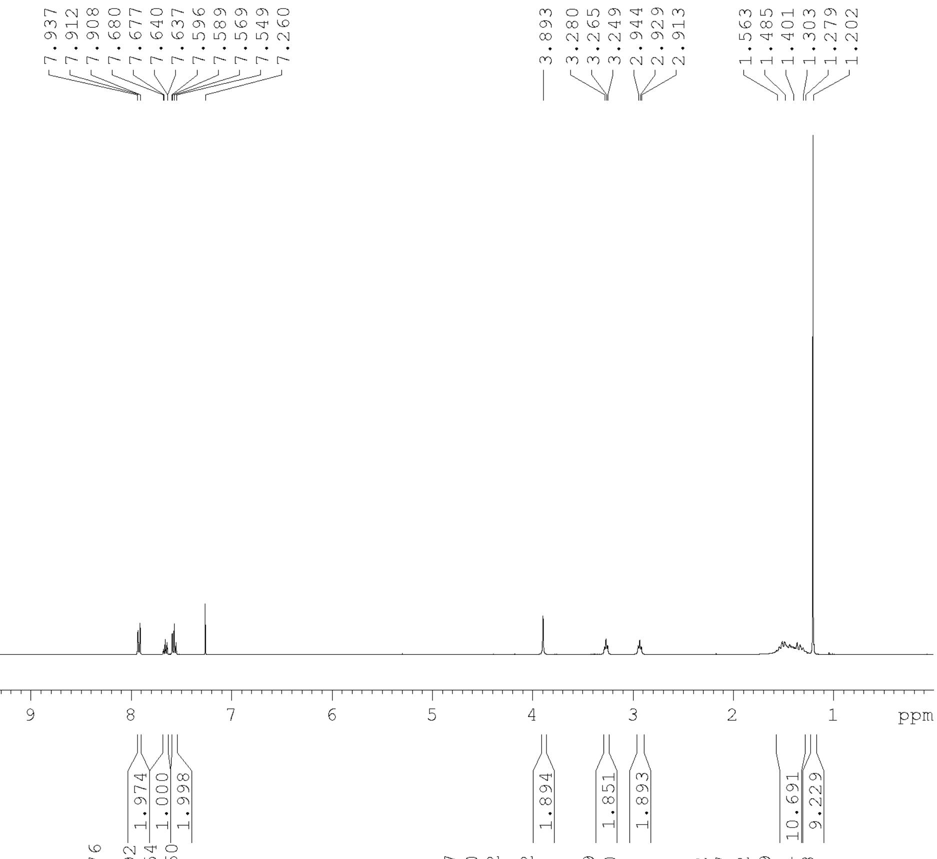

7.937
7.912
7.908
7.680
7.677
7.640
7.637
7.596
7.589
7.569
7.549
7.260
3.893
3.280
3.265
3.249
2.944
2.929
2.913
1.563
1.485
1.401
1.303
1.279
1.202
11
10
9
8
7
6
5
4
3
2
1
ppm
1.974
1.000
1.998
1.894
1.851
1.893
10.691
9.229

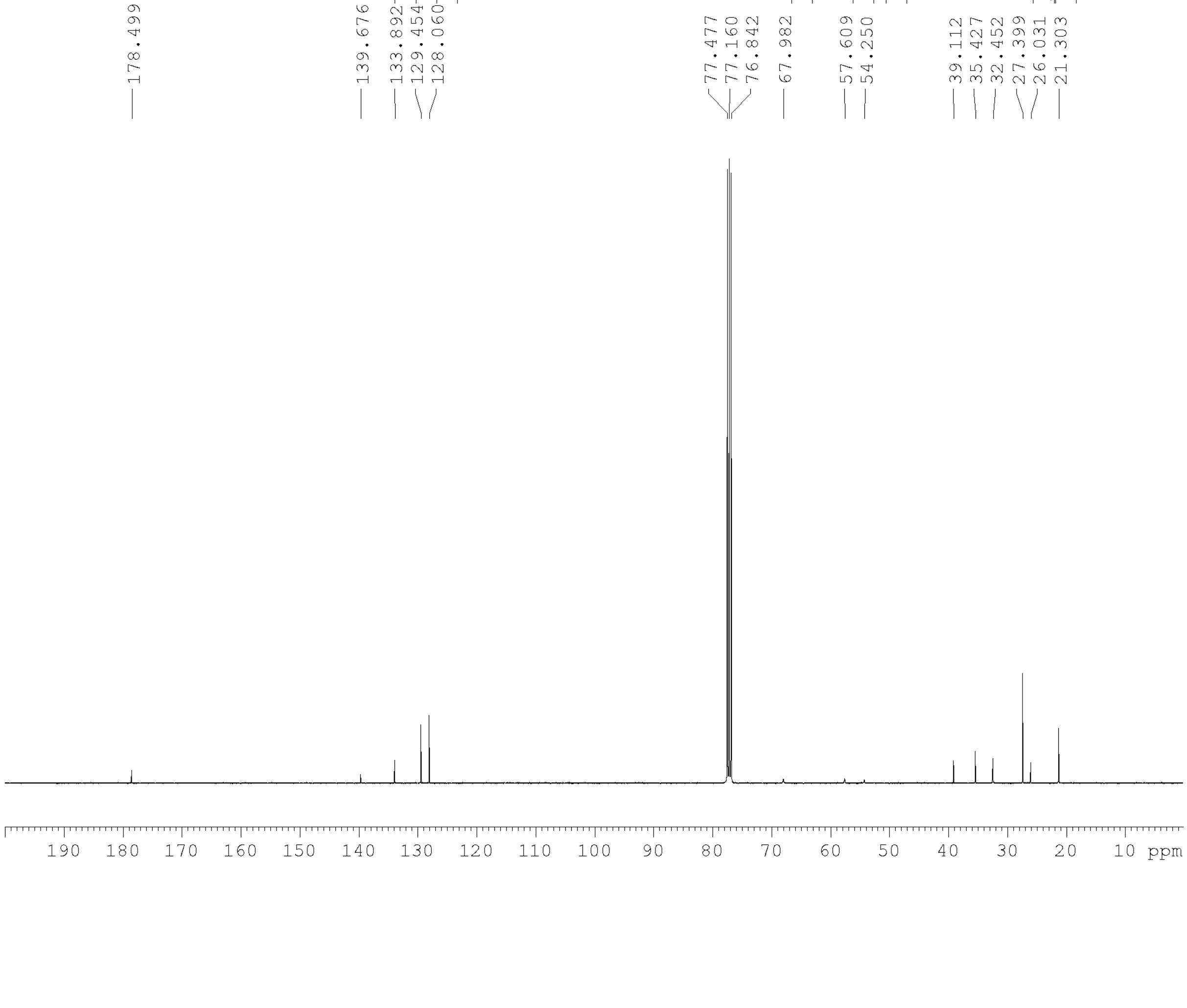

178.499
139.676
133.892
129.454
128.060
77.477
77.160
76.842
67.982
57.609
54.250
39.112
35.427
32.452
27.399
26.031
21.303
190
180
170
160
150
140
130
120
110
100
90
80
70
60
50
40
30
20
10 ppm

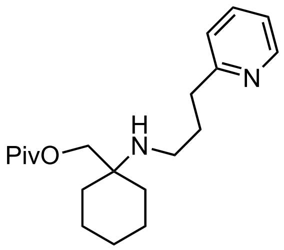
N
H
N
PivO

1j

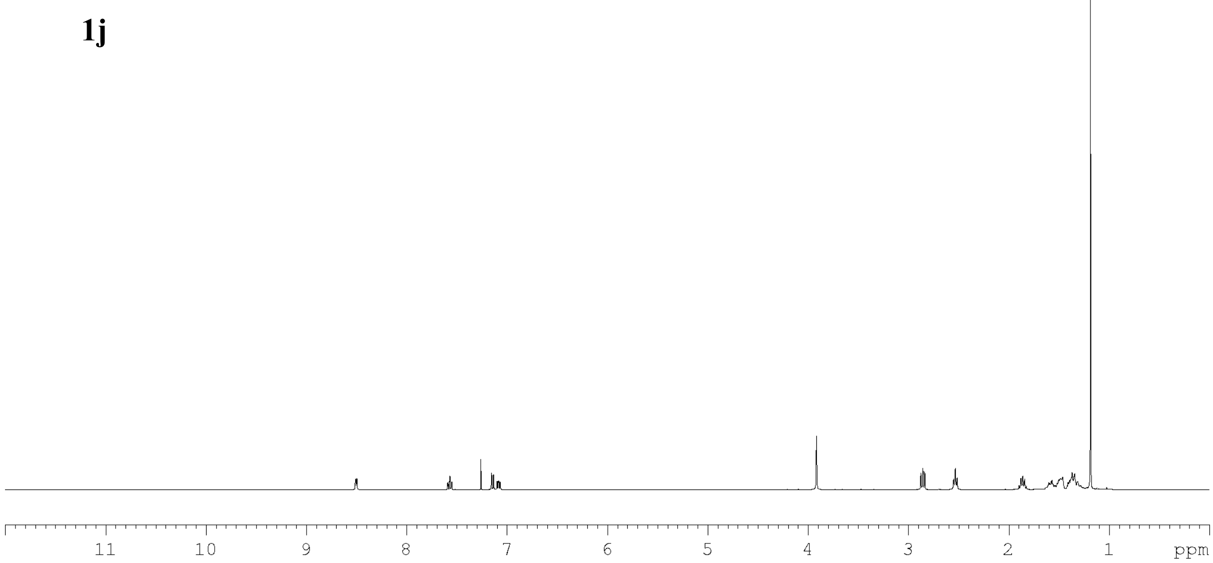
8.510
8.508
8.498
8.496
7.592
7.587
7.572
7.568
7.553
7.549
7.260
7.153
7.134
7.099
7.085
7.080
7.068
3.915
2.876
2.857
2.837
2.549
2.531
2.514
1.896
1.879
1.859
1.841
1.823
1.625
1.569
1.462
1.368
1.342
1.280
1.185
11
10
9
8
7
6
5
4
3
2
1
ppm
1.017
1.000
1.039
1.011
2.045
2.094
2.016
2.069
10.194
9.339

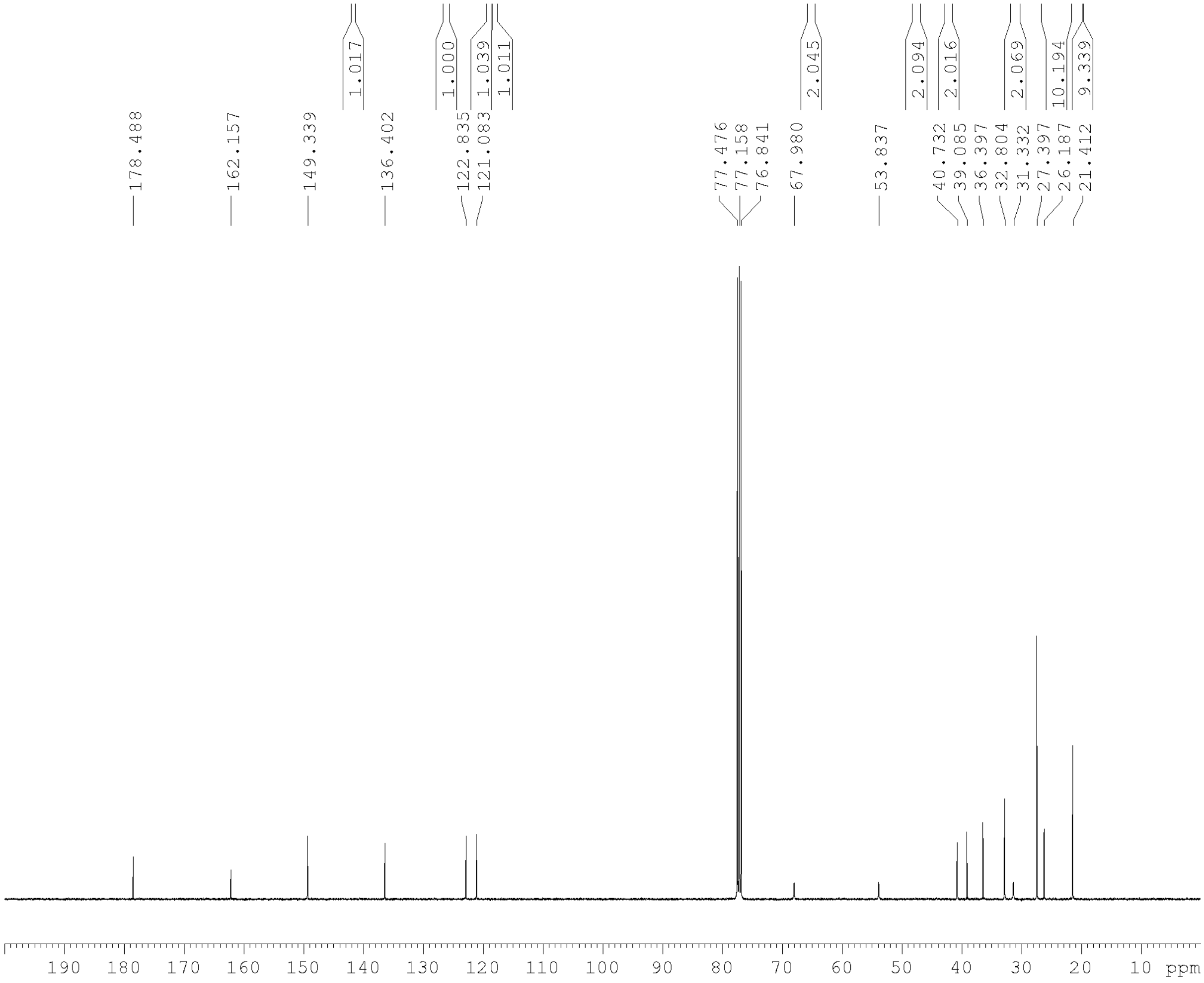
178.488
162.157
149.339
136.402
122.835
121.083
77.476
77.158
76.841
67.980
53.837
40.732
39.085
36.397
32.804
31.332
27.397
26.187
21.412
190
180
170
160
150
140
130
120
110
100
90
80
70
60
50
40
30
20
10
ppm

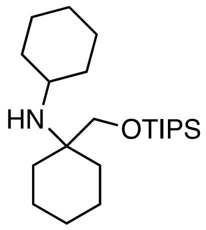
HN
OTIPS

1k

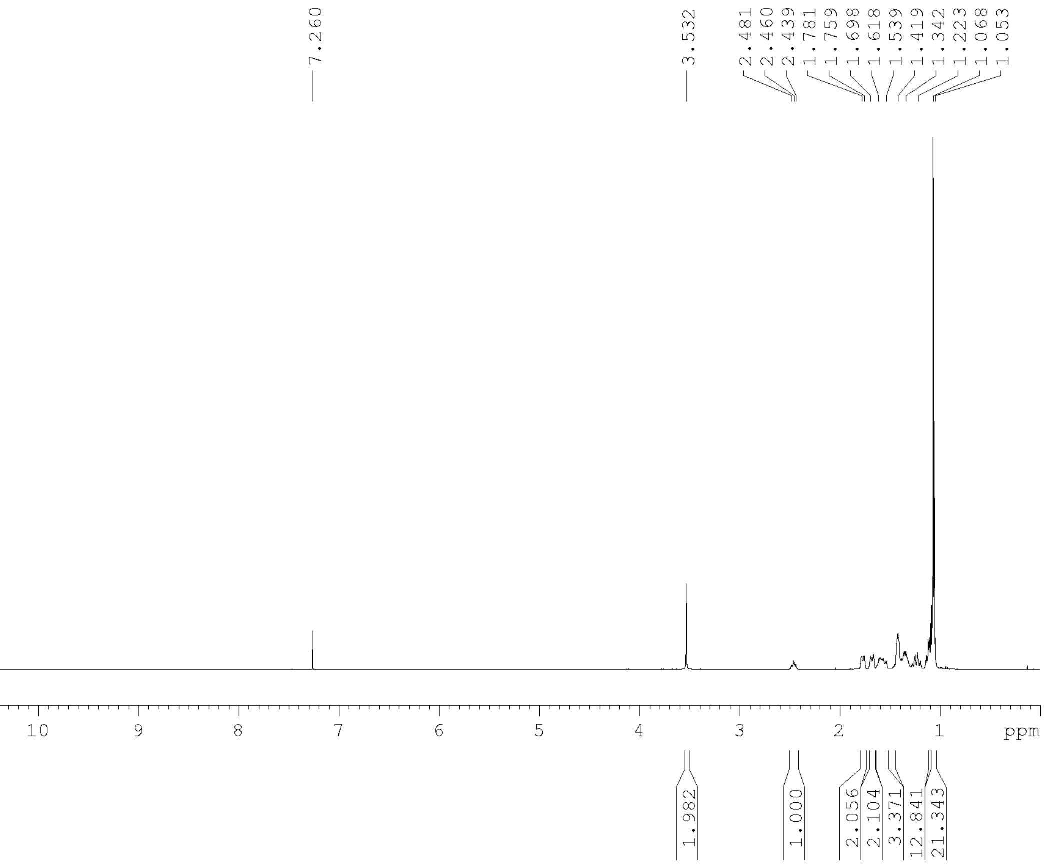
7.260
3.532
2.481
2.460
2.439
1.781
1.759
1.698
1.618
1.539
1.419
1.342
1.223
1.068
1.053
11 10 9 8 7 6 5 4 3 2 1 ppm
1.982
1.000
2.056
2.104
3.371
12.841
21.343

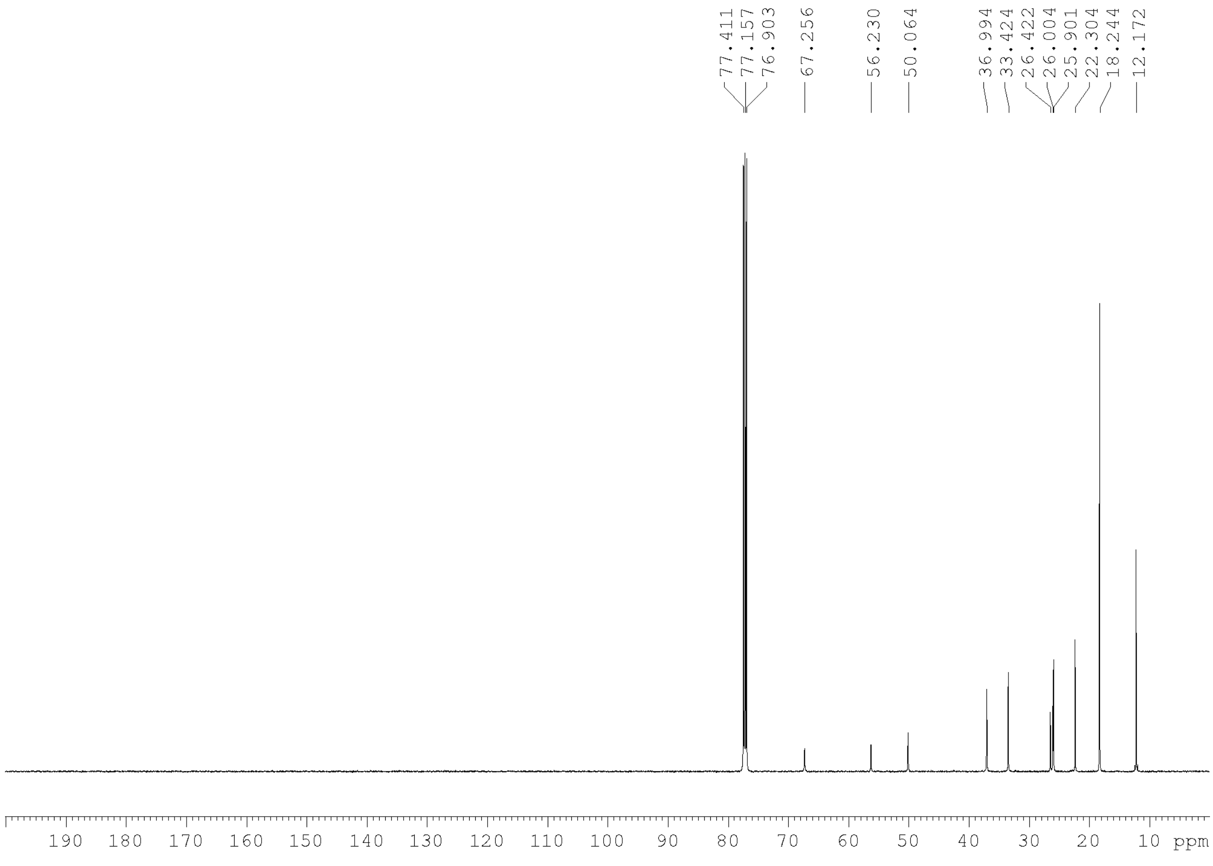
77.411
77.157
76.903
67.256
56.230
50.064
36.994
33.424
26.422
26.004
25.901
22.304
18.244
12.172
190 180 170 160 150 140 130 120 110 100 90 80 70 60 50 40 30 20 10 ppm

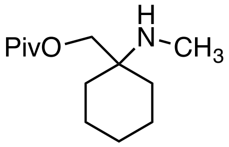
PivO
H
N
CH3

1m

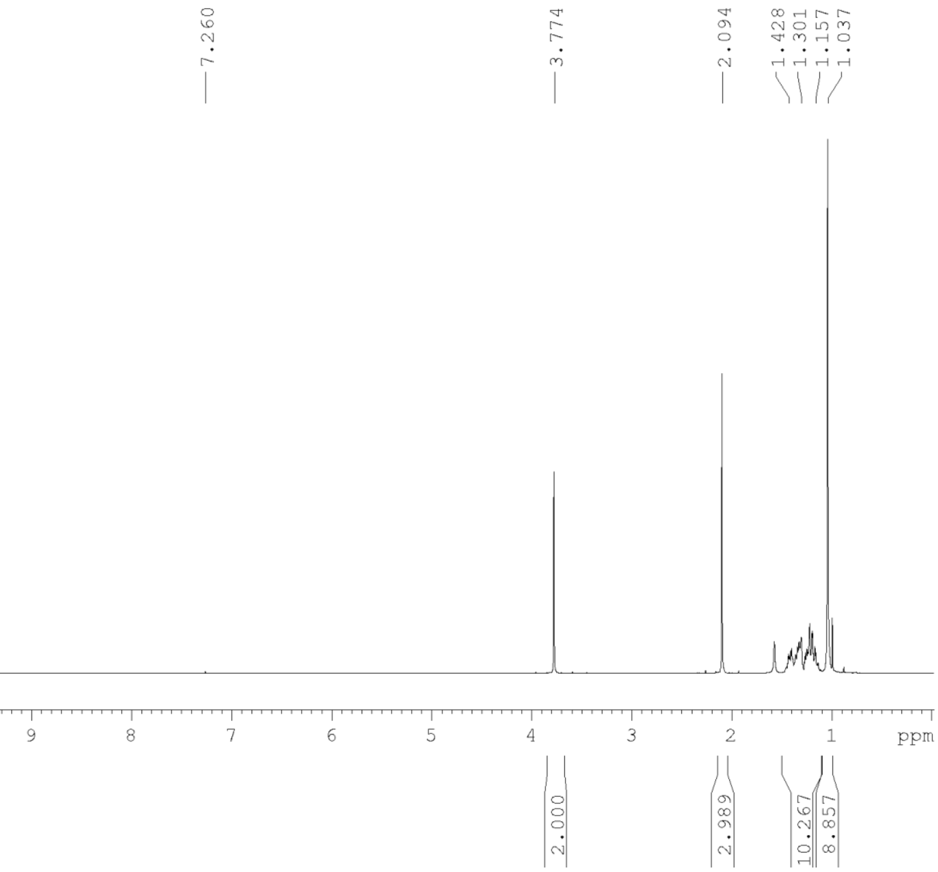
7.260
3.774
2.094
1.428
1.301
1.157
1.037
2.000
2.989
10.267
8.857
11 10 9 8 7 6 5 4 3 2 1 ppm

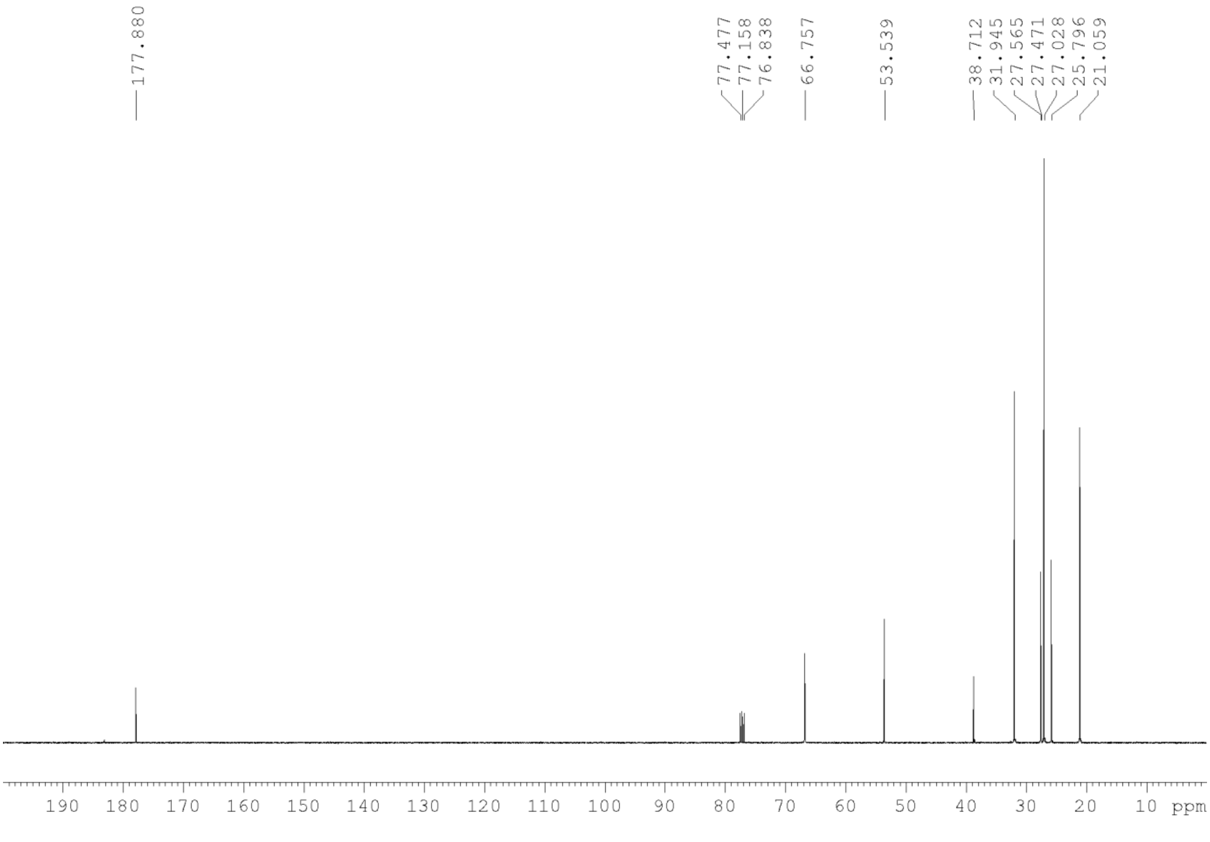
177.880
77.477
77.158
76.838
66.757
53.539
38.712
31.945
27.565
27.471
27.028
25.796
21.059
190 180 170 160 150 140 130 120 110 100 90 80 70 60 50 40 30 20 10 ppm

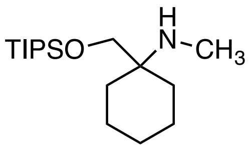

TIPSO
H
N
CH3

1n

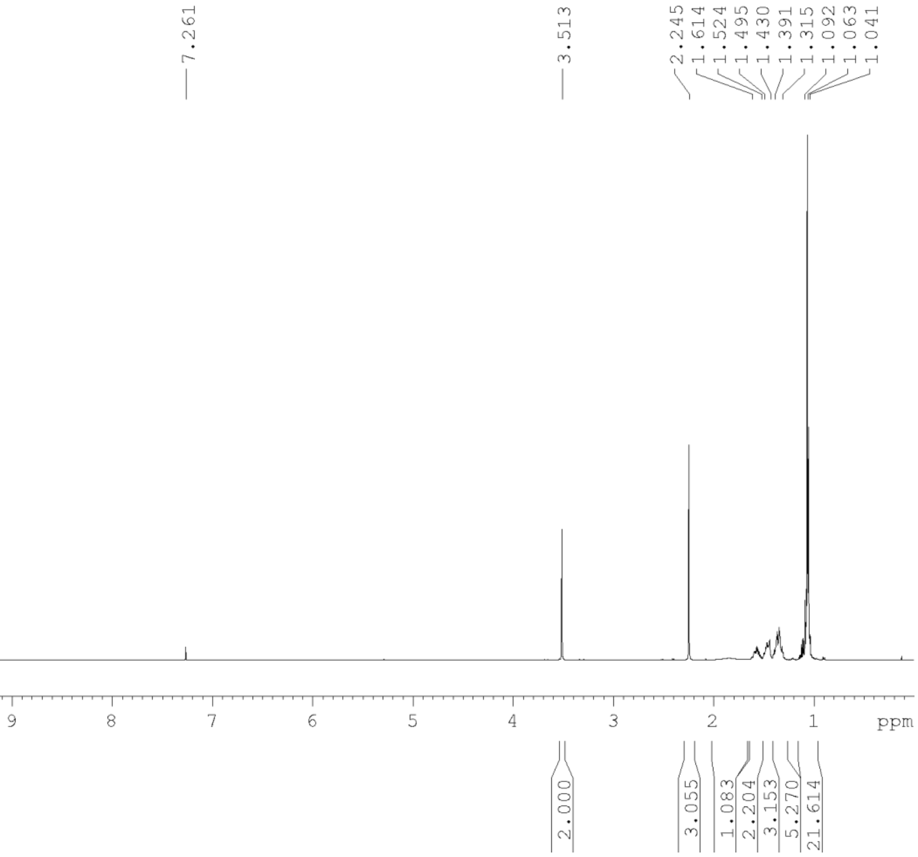

7.261
3.513
2.245
1.614
1.524
1.495
1.430
1.391
1.315
1.092
1.063
1.041
11
10
9
8
7
6
5
4
3
2
1
ppm
2.000
3.055
1.083
2.204
3.153
5.270
21.614

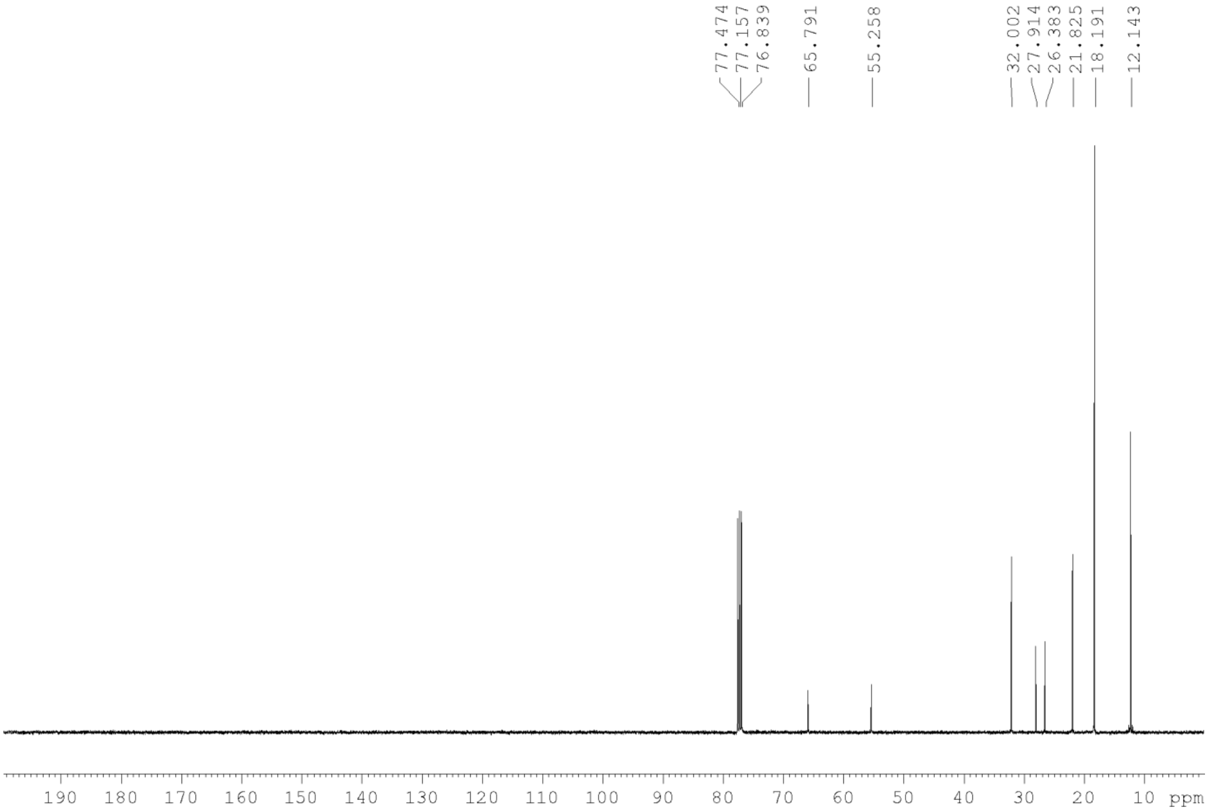

77.474
77.157
76.839
65.791
55.258
32.002
27.914
26.383
21.825
18.191
12.143
190
180
170
160
150
140
130
120
110
100
90
80
70
60
50
40
30
20
10
ppm

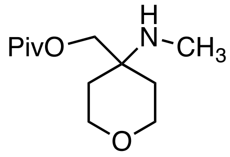
PivO
H
N
CH3
O

1o

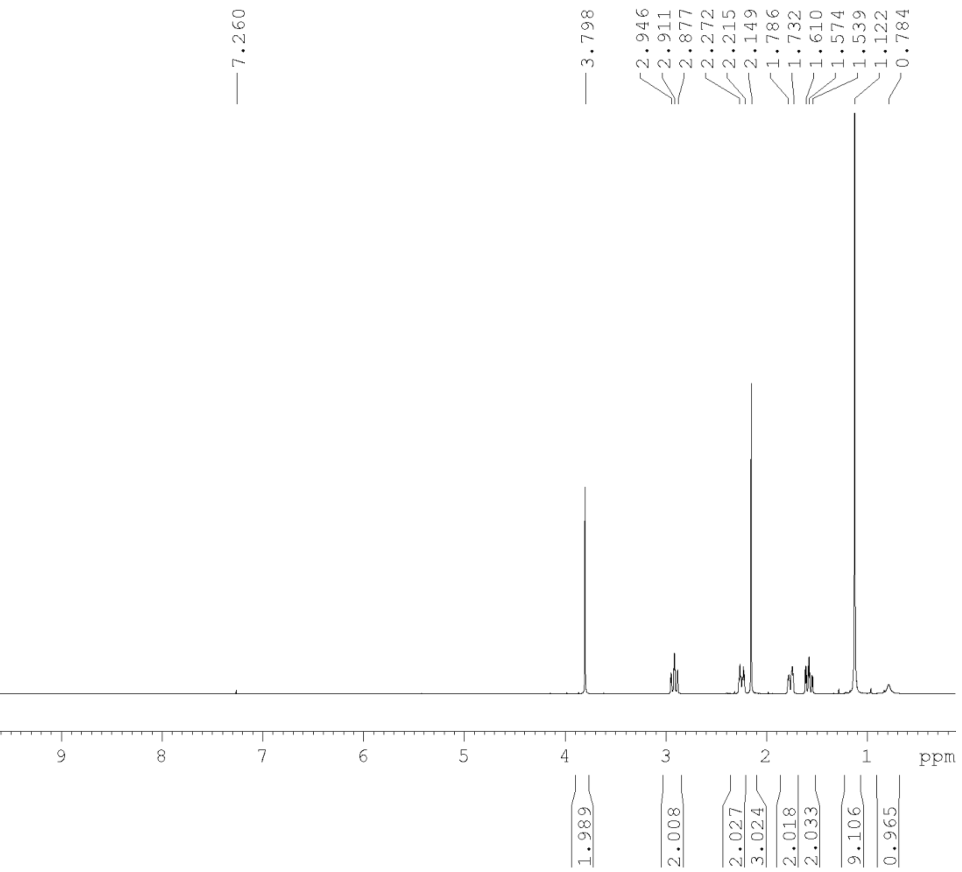
7.260
3.798
2.946
2.911
2.877
2.272
2.215
2.149
1.786
1.732
1.610
1.574
1.539
1.122
0.784
11 10 9 8 7 6 5 4 3 2 1 ppm
1.989
2.008
2.027
3.024
2.018
2.033
9.106
0.965

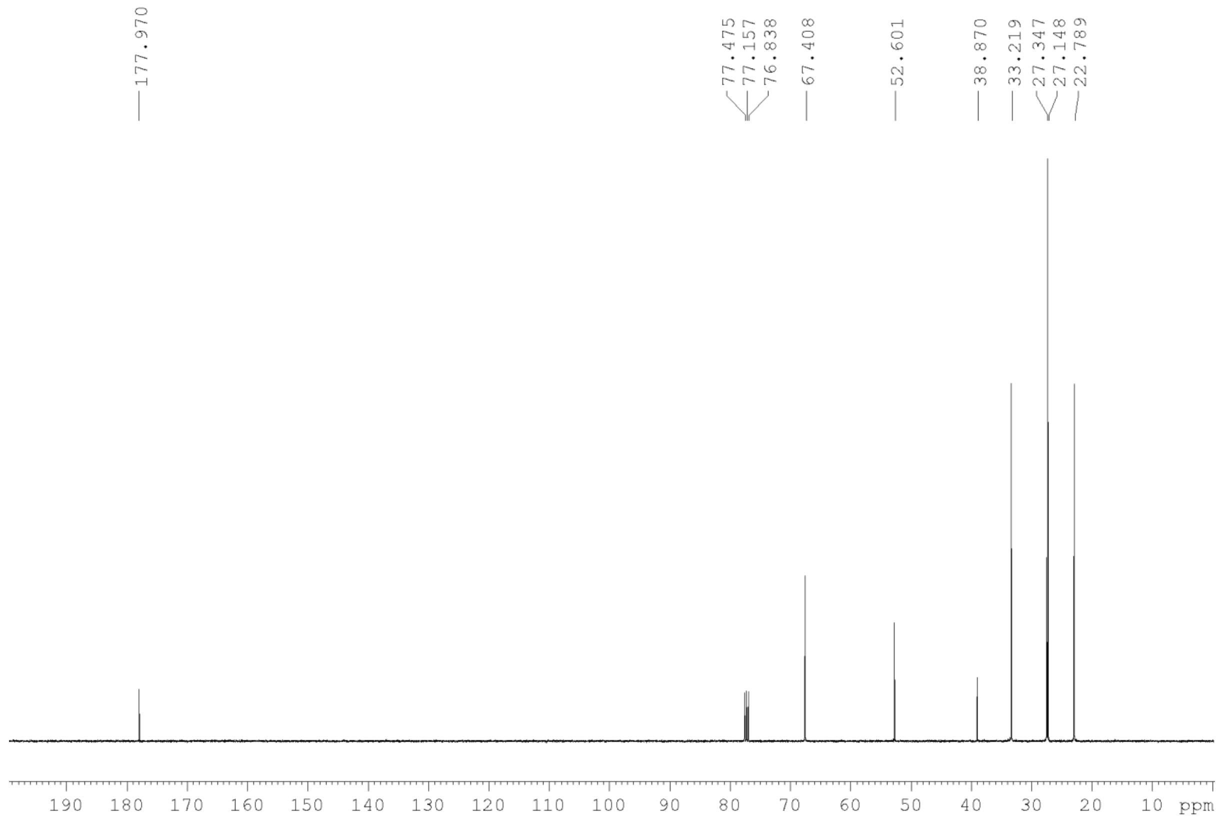
177.970
77.475
77.157
76.838
67.408
52.601
38.870
33.219
27.347
27.148
22.789
190 180 170 160 150 140 130 120 110 100 90 80 70 60 50 40 30 20 10 ppm

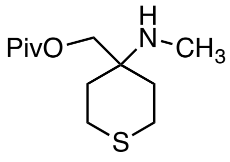

PivO
N
H
CH3
S

1p

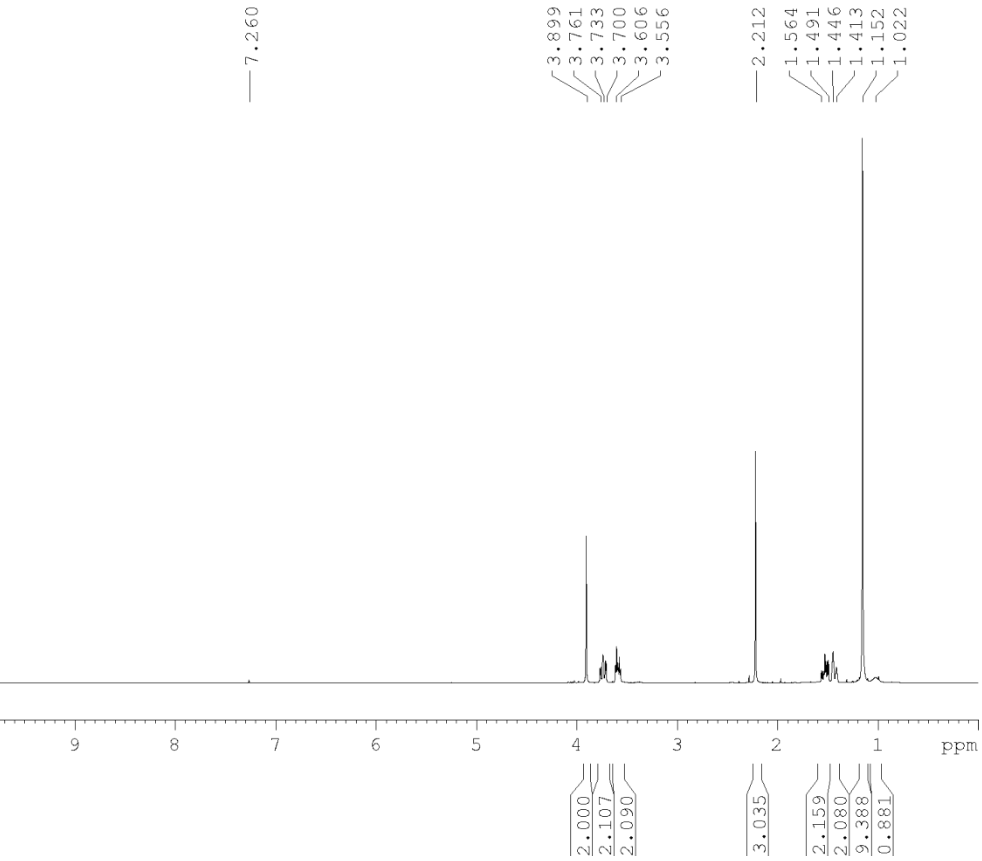

7.260
3.899
3.761
3.733
3.700
3.606
3.556
2.212
1.564
1.491
1.446
1.413
1.152
1.022
11 10 9 8 7 6 5 4 3 2 1 ppm
2.000
2.107
2.090
3.035
2.159
2.080
9.388
0.881

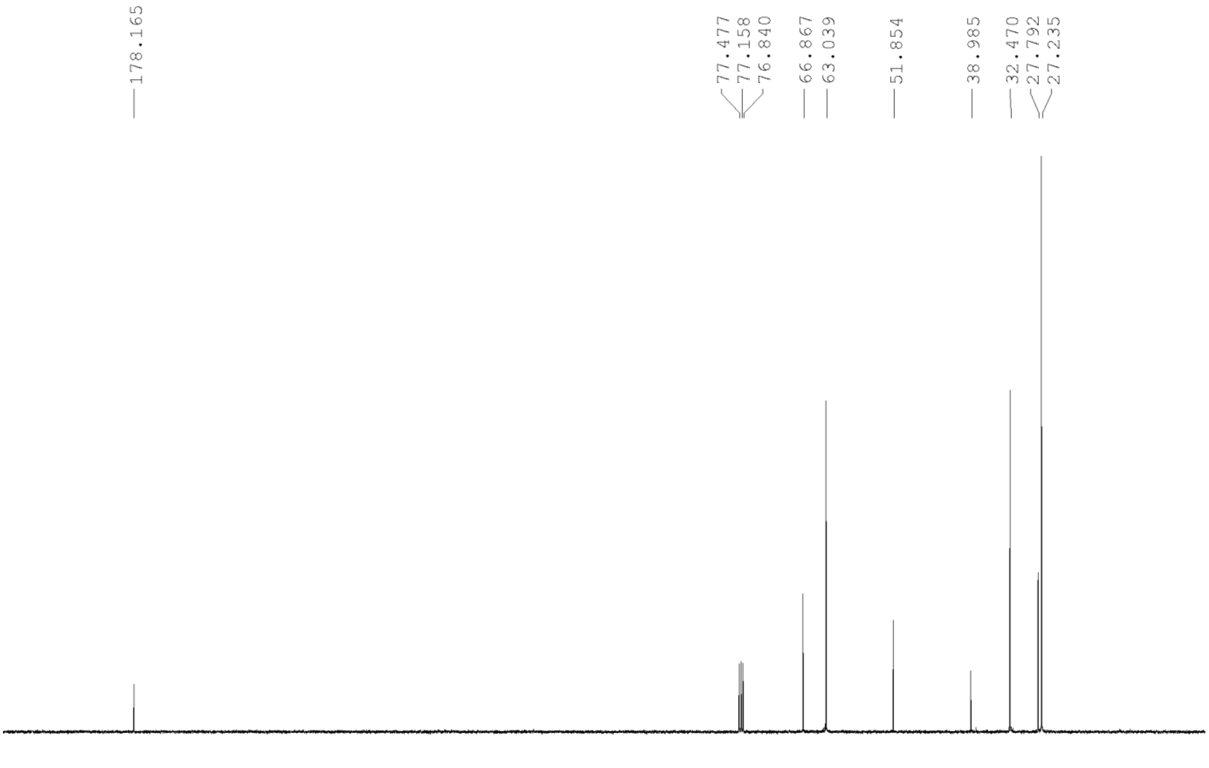

178.165
77.477
77.158
76.840
66.867
63.039
51.854
38.985
32.470
27.792
27.235
190 180 170 160 150 140 130 120 110 100 90 80 70 60 50 40 30 20 10 ppm

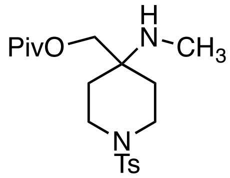
PivO
N
CH3
N
Ts

1q

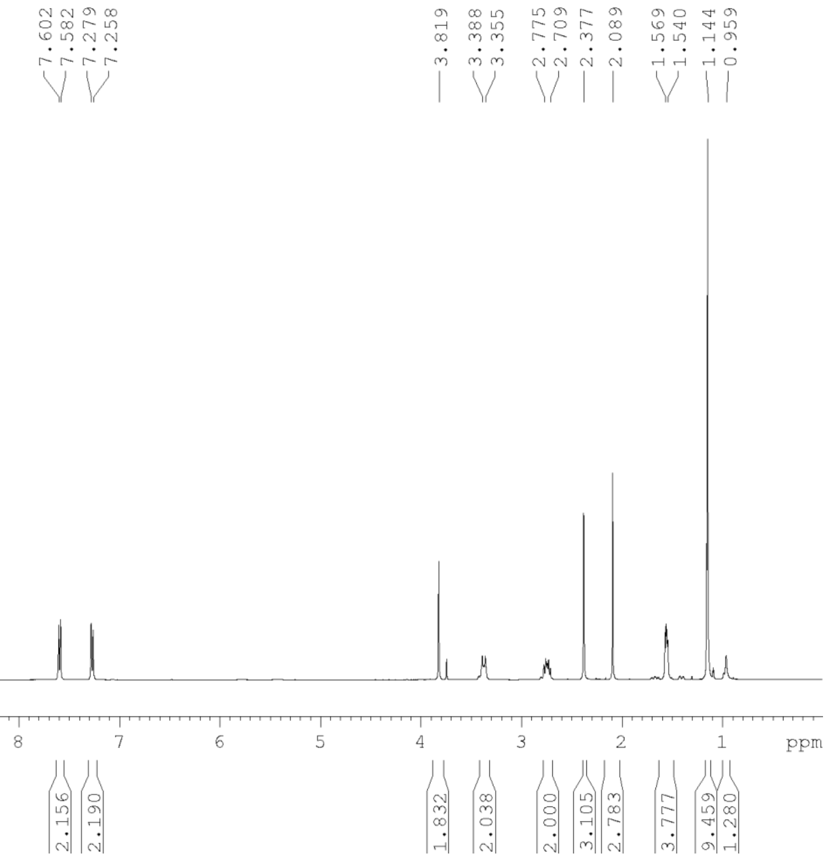
7.602
7.582
7.279
7.258
3.819
3.388
3.355
2.775
2.709
2.377
2.089
1.569
1.540
1.144
0.959
11
10
9
8
7
6
5
4
3
2
1
ppm
2.156
2.190
1.832
2.038
2.000
3.105
2.783
3.777
9.459
1.280

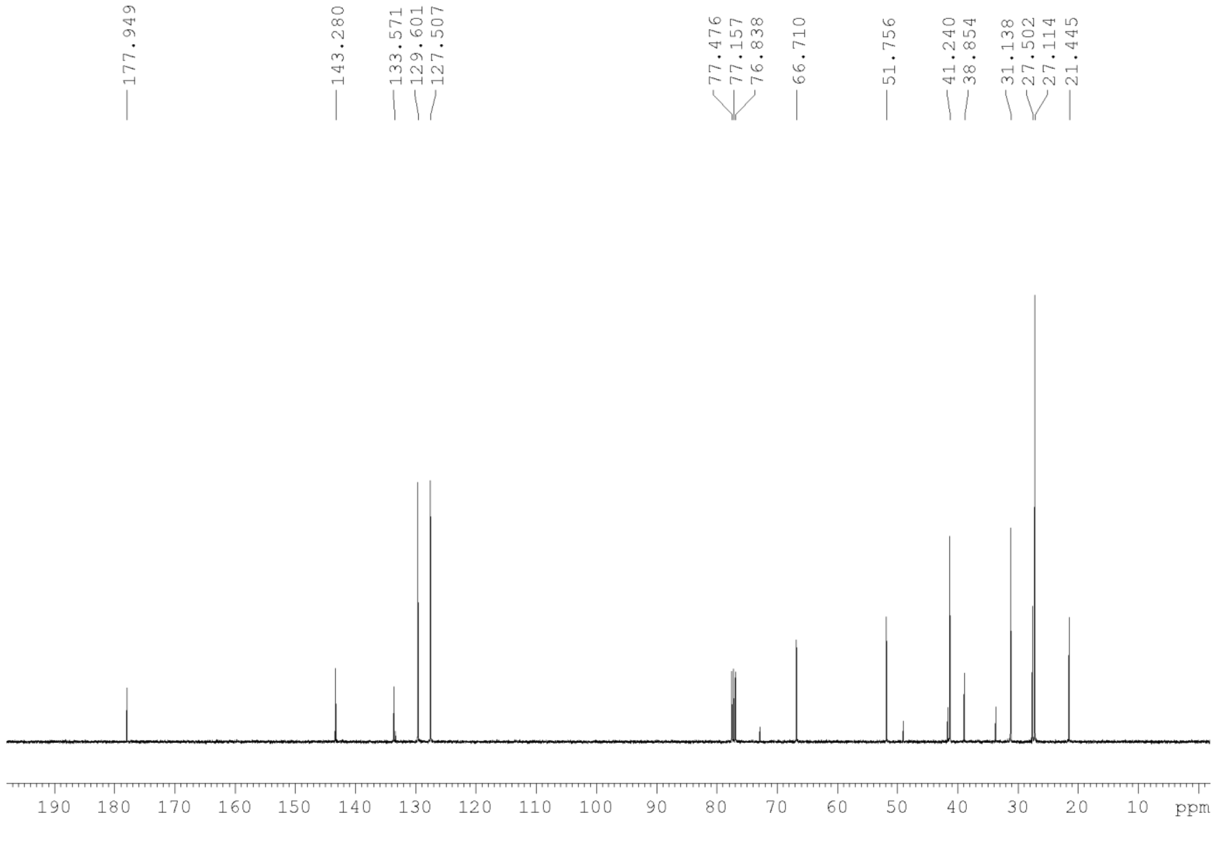
177.949
143.280
133.571
129.601
127.507
77.476
77.157
76.838
66.710
51.756
41.240
38.854
31.138
27.502
27.114
21.445
190
180
170
160
150
140
130
120
110
100
90
80
70
60
50
40
30
20
10
ppm

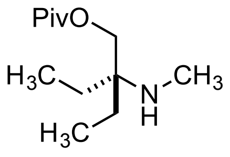
PivO
H_3C
N
CH_3
H
H_3C

1r

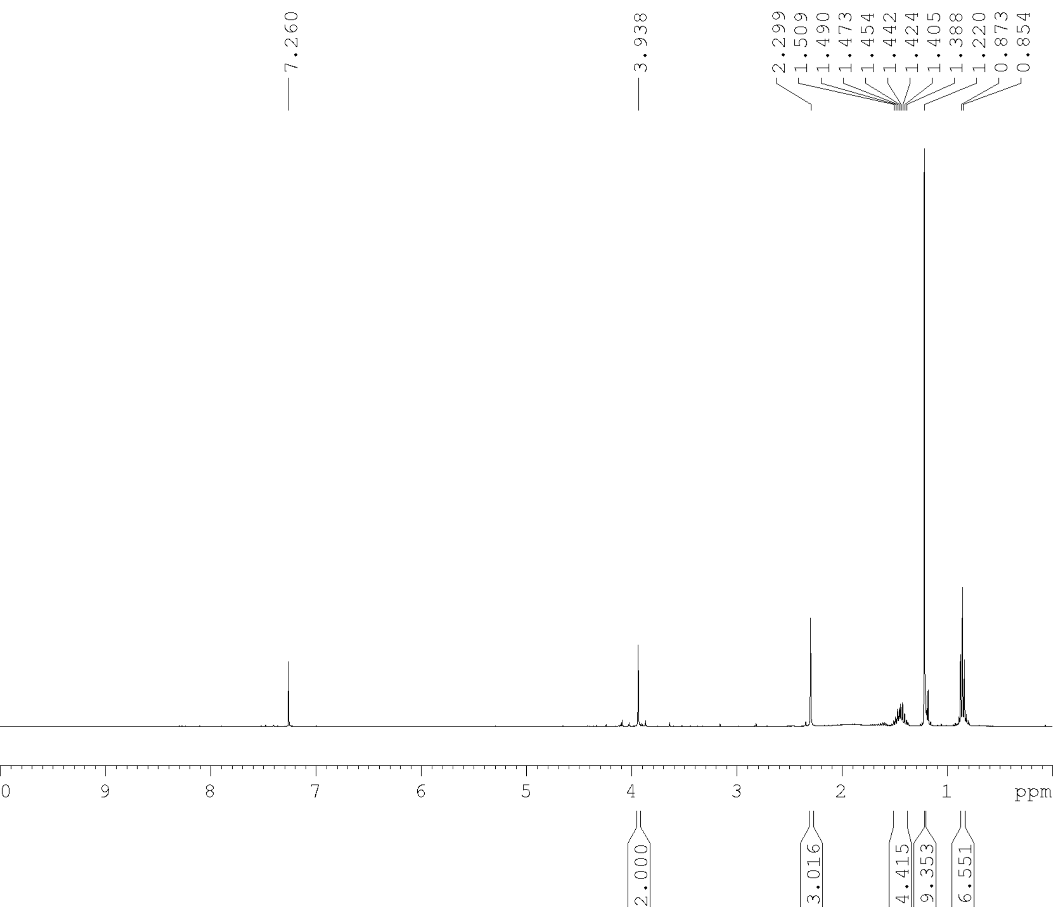
7.260
3.938
2.299
1.509
1.490
1.473
1.454
1.442
1.424
1.405
1.388
1.220
0.873
0.854
11 10 9 8 7 6 5 4 3 2 1 ppm
2.000
3.016
4.415
9.353
6.551

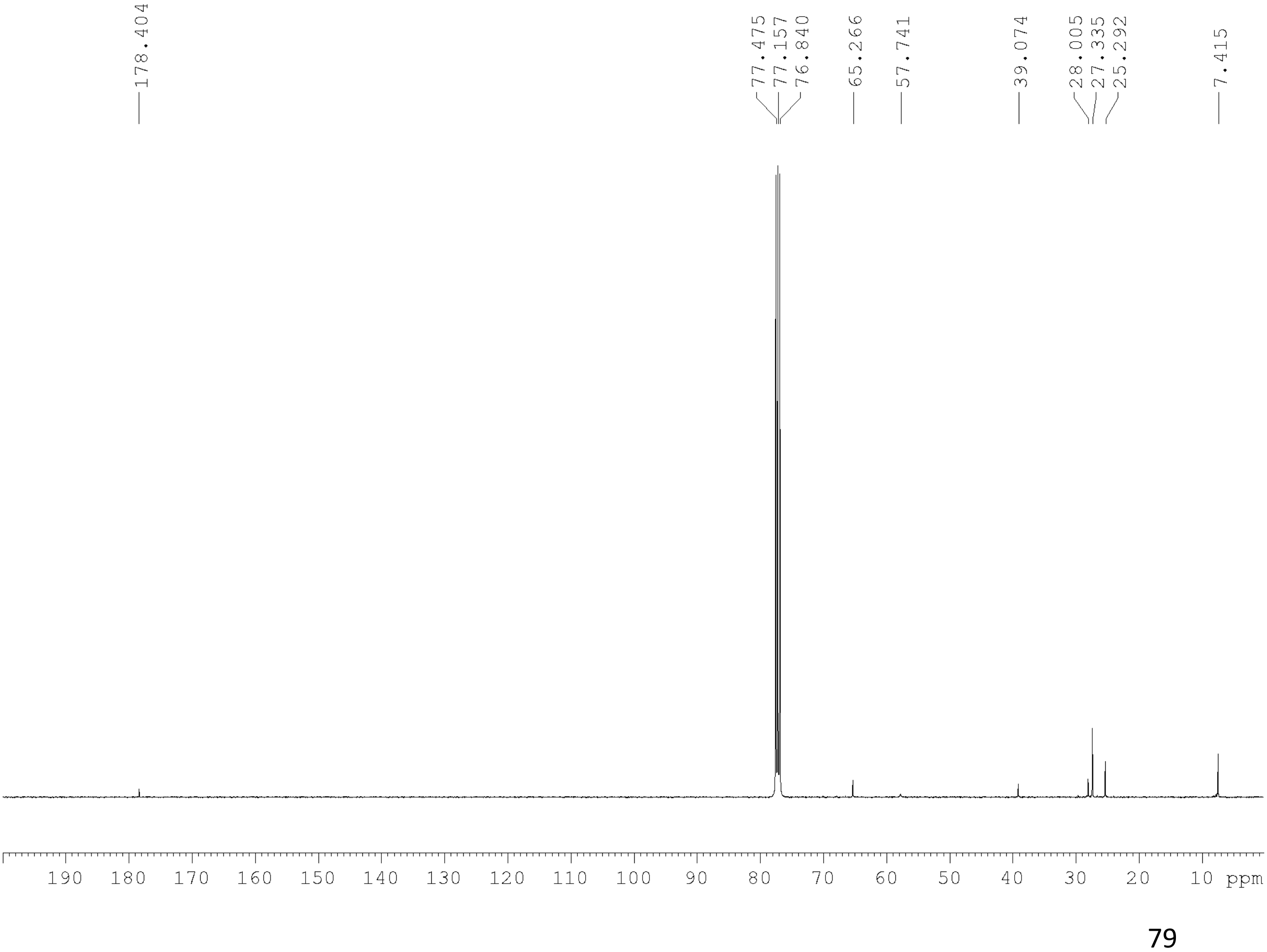
178.404
77.475
77.157
76.840
65.266
57.741
39.074
28.005
27.335
25.292
7.415
190 180 170 160 150 140 130 120 110 100 90 80 70 60 50 40 30 20 10 ppm

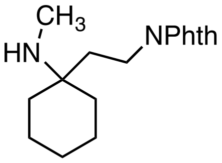
CH3
HN
NPhth

1s

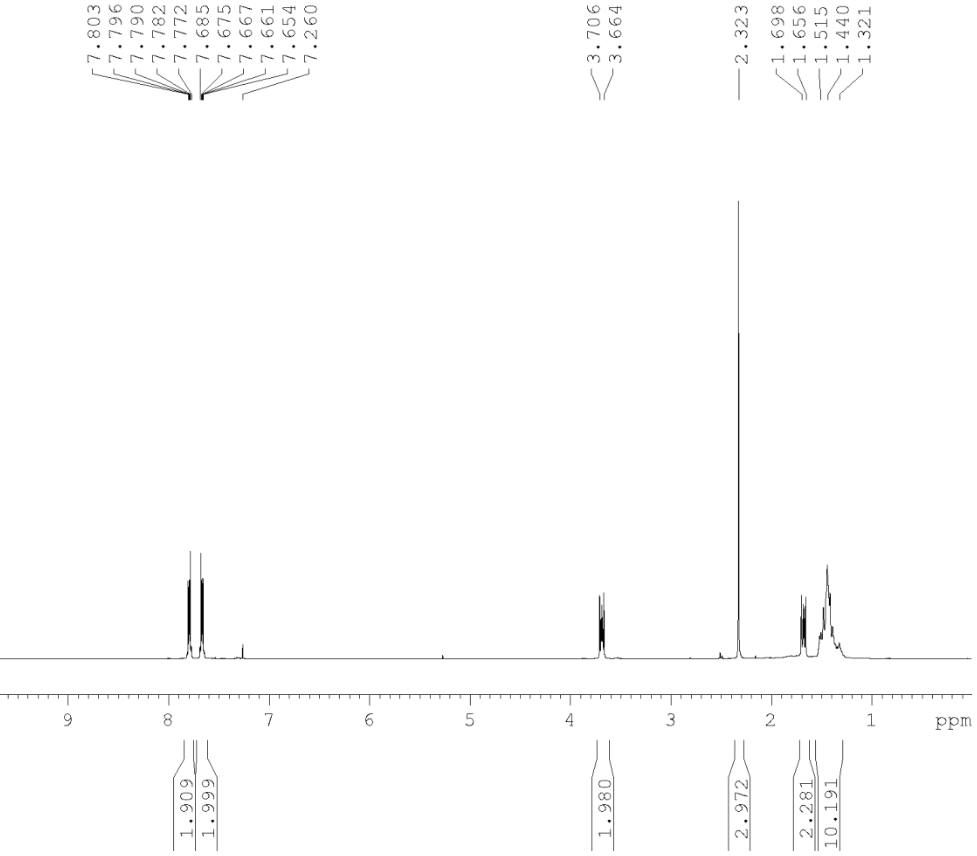
7.803
7.796
7.790
7.782
7.772
7.685
7.675
7.667
7.661
7.654
7.260
3.706
3.664
2.323
1.698
1.656
1.515
1.440
1.321
ppm
1.909
1.999
1.980
2.972
2.281
10.191

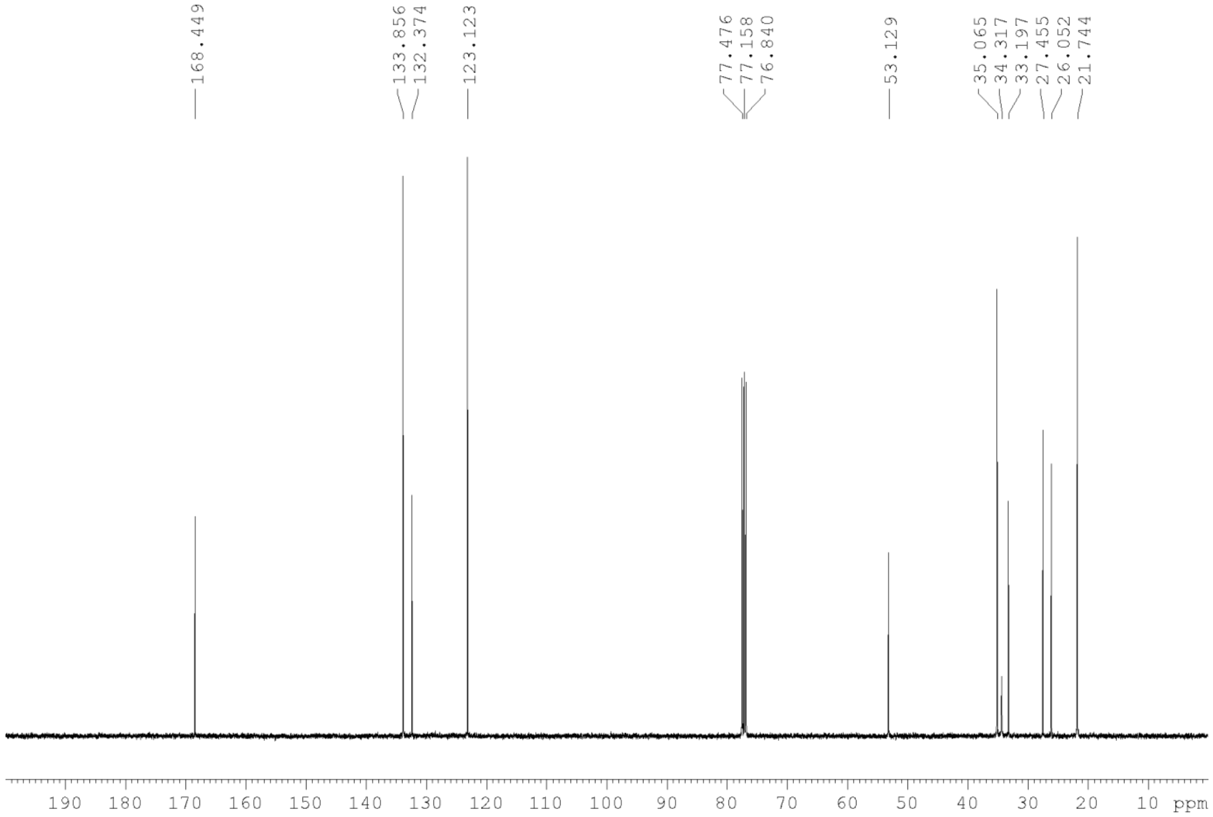
168.449
133.856
132.374
123.123
77.476
77.158
76.840
53.129
35.065
34.317
33.197
27.455
26.052
21.744
ppm

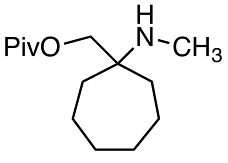
PivO
H
N
CH3

1t

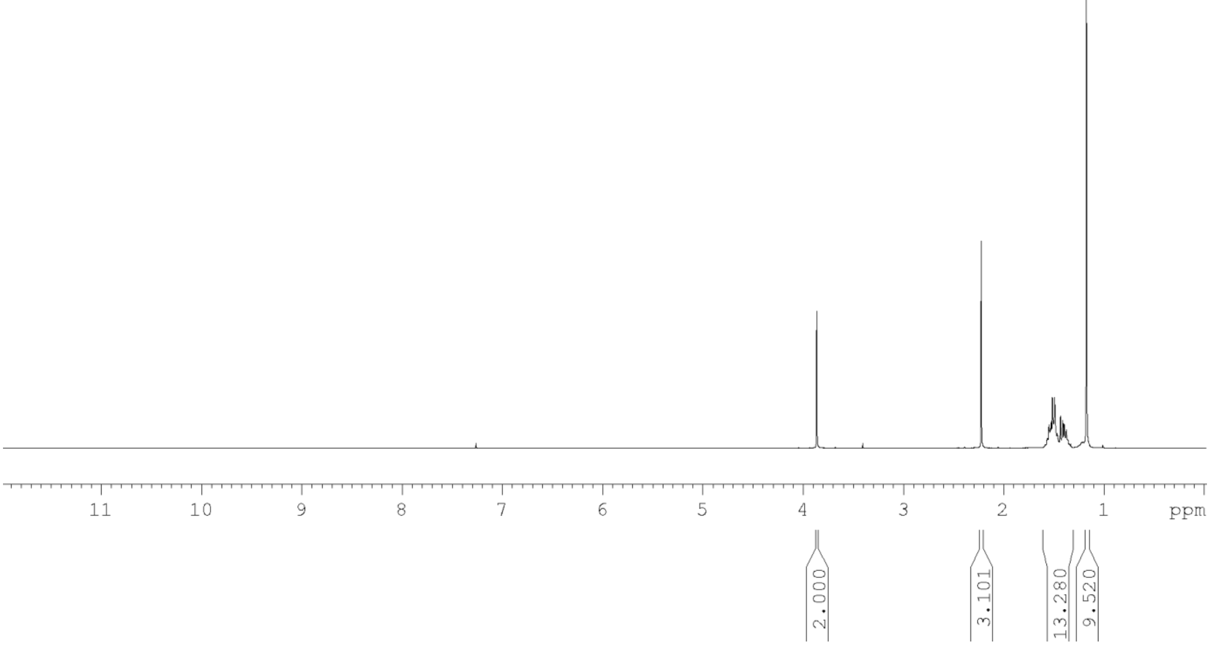
7.259
3.861
2.220
1.545
1.465
1.369
1.170
11
10
9
8
7
6
5
4
3
2
1
ppm
2.000
3.101
13.280
9.520

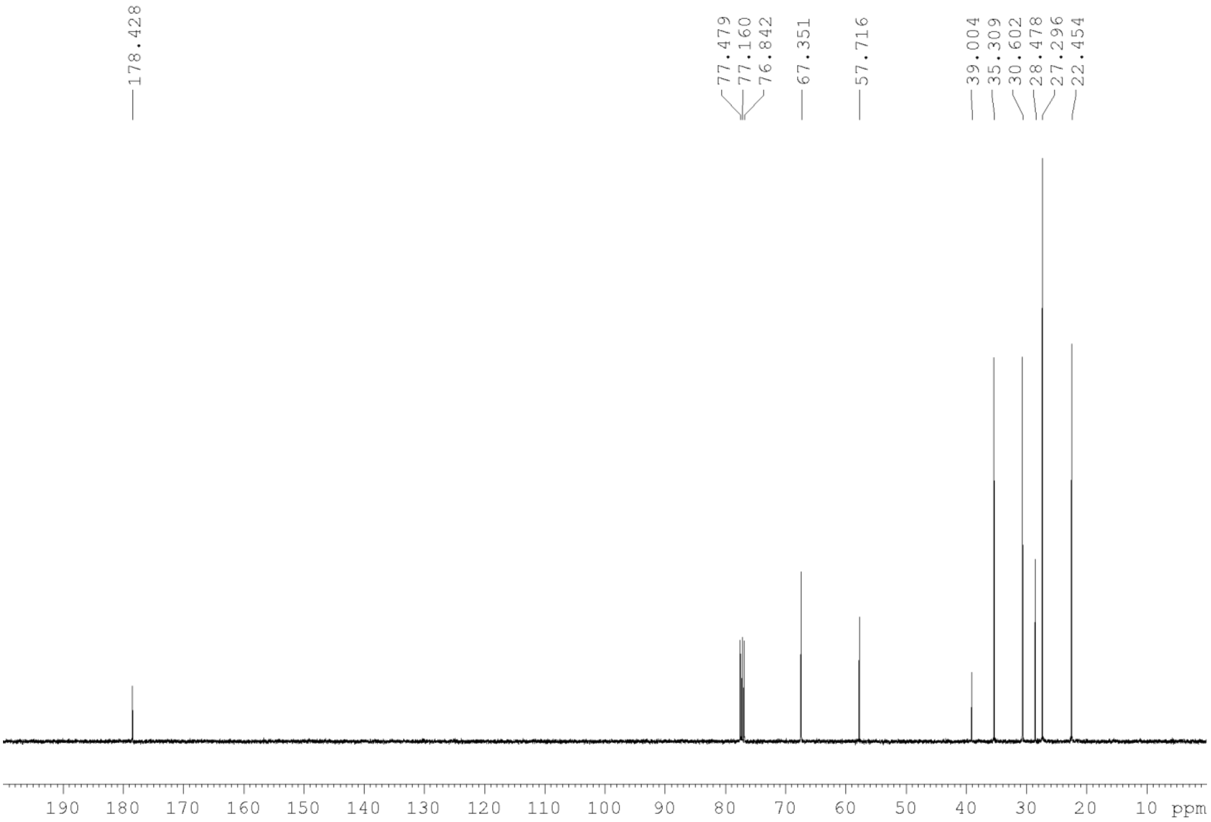
178.428
77.479
77.160
76.842
67.351
57.716
39.004
35.309
30.602
28.478
27.296
22.454
190
180
170
160
150
140
130
120
110
100
90
80
70
60
50
40
30
20
10
ppm

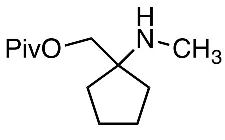
PivO
H
N
CH3

1u

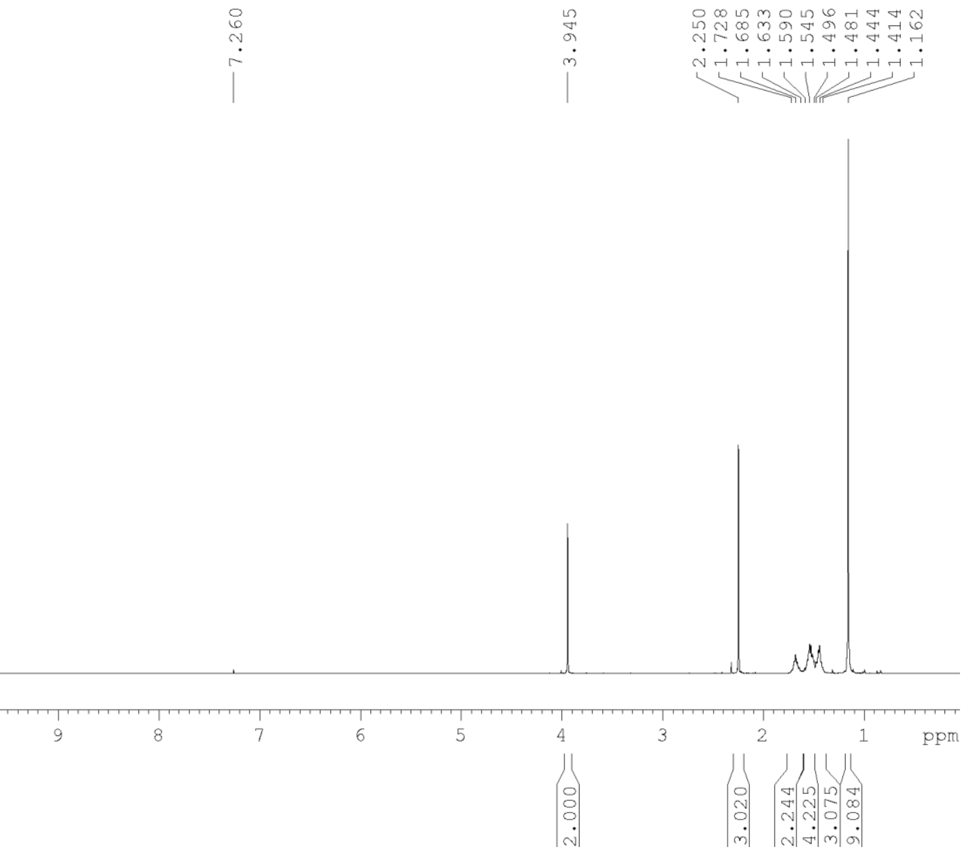
7.260
3.945
2.250
1.728
1.685
1.633
1.590
1.545
1.496
1.481
1.444
1.414
1.162
11 10 9 8 7 6 5 4 3 2 1 ppm
2.000
3.020
2.244
4.225
3.075
9.084

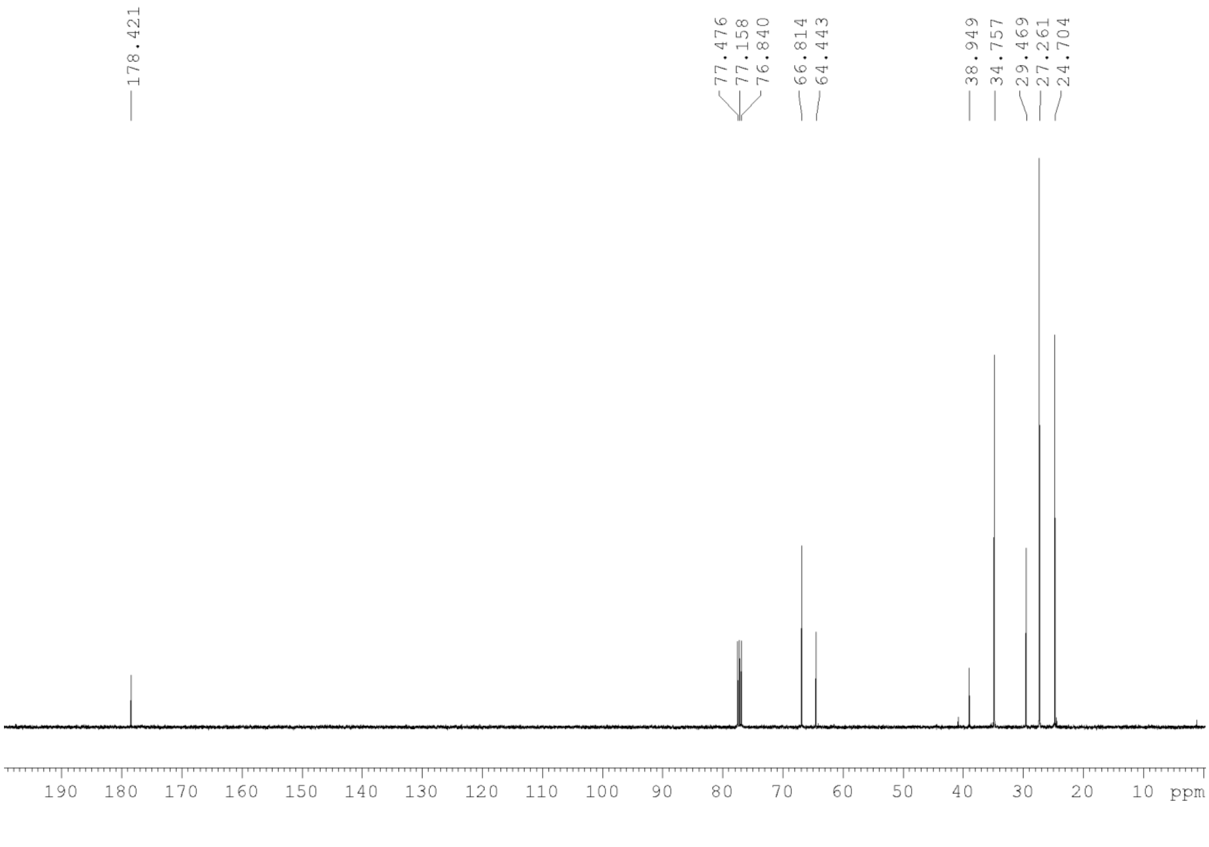
178.421
77.476
77.158
76.840
66.814
64.443
38.949
34.757
29.469
27.261
24.704
190 180 170 160 150 140 130 120 110 100 90 80 70 60 50 40 30 20 10 ppm

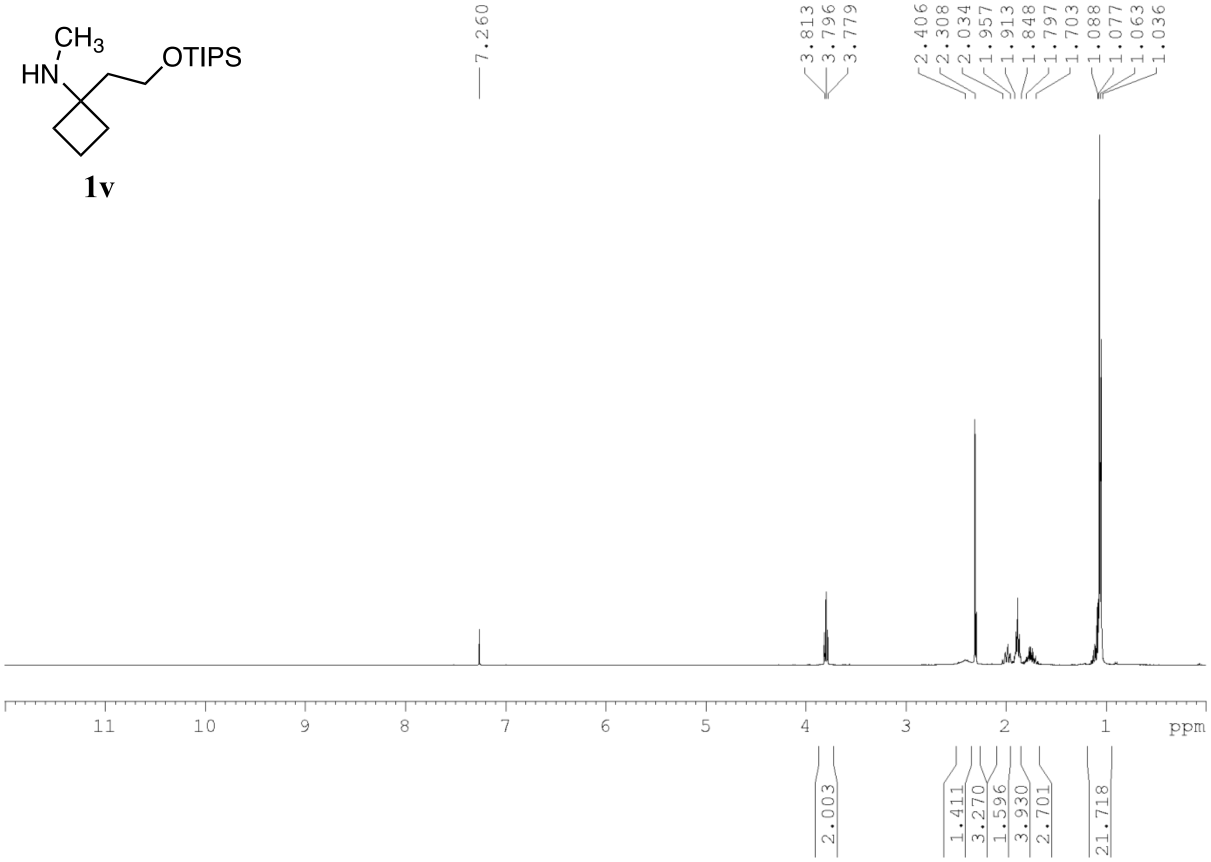
CH3
HN
OTIPS
1v
7.260
3.813
3.796
3.779
2.406
2.308
2.034
1.957
1.913
1.848
1.797
1.703
1.088
1.077
1.063
1.036
11 10 9 8 7 6 5 4 3 2 1 ppm
2.003
1.411
3.270
1.596
3.930
2.701
21.718

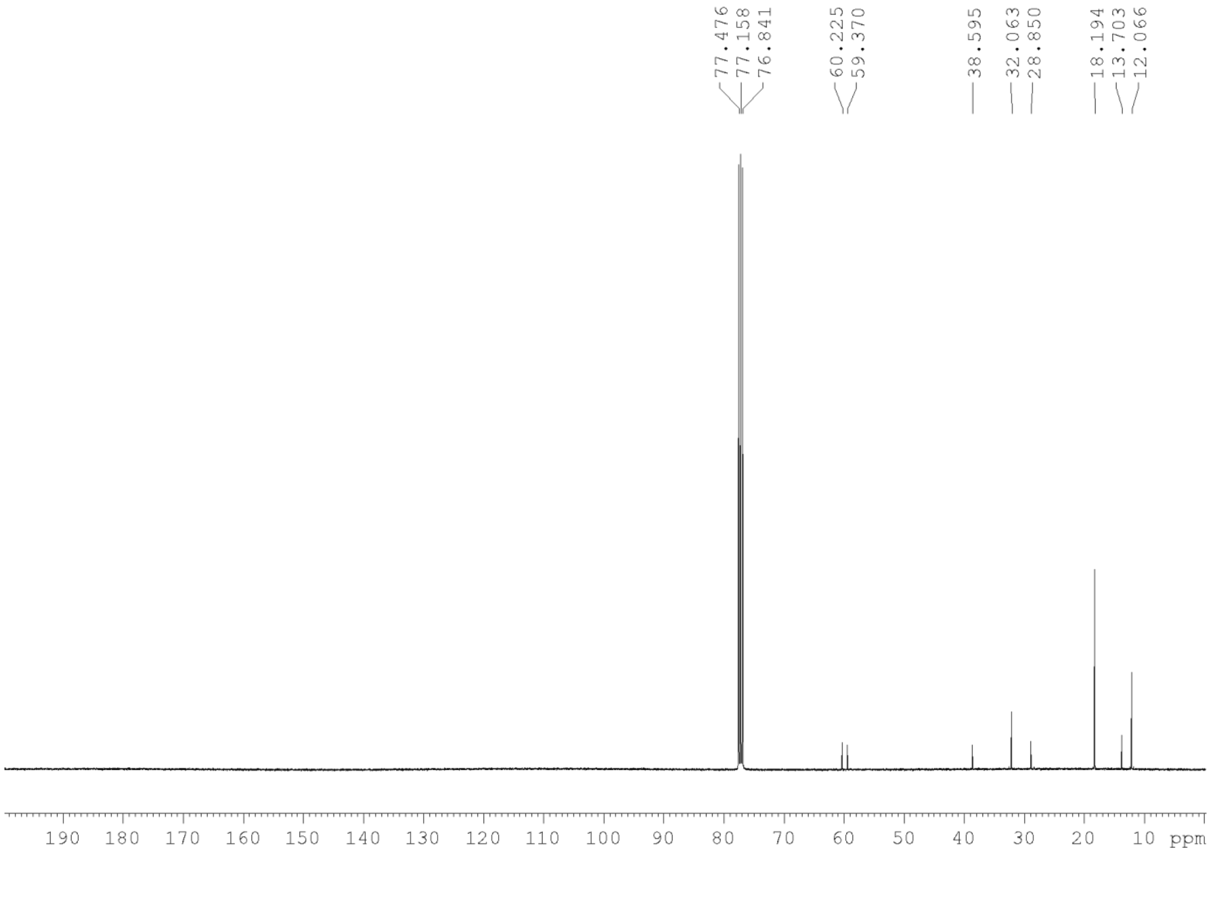
77.476
77.158
76.841
60.225
59.370
38.595
32.063
28.850
18.194
13.703
12.066
190 180 170 160 150 140 130 120 110 100 90 80 70 60 50 40 30 20 10 ppm

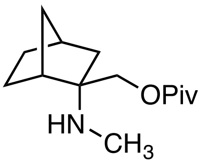

OPiv
HN
CH3

1w

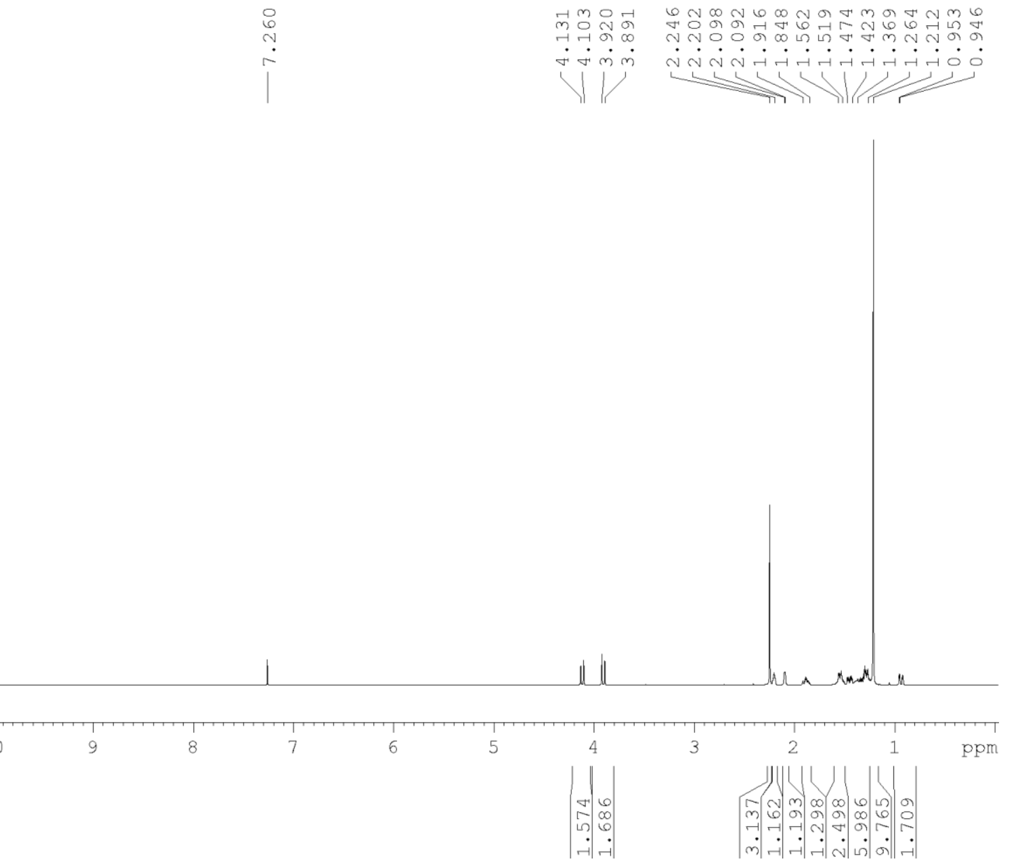

7.260
4.131
4.103
3.920
3.891
2.246
2.202
2.098
2.092
1.916
1.848
1.562
1.519
1.474
1.423
1.369
1.264
1.212
0.953
0.946
11 10 9 8 7 6 5 4 3 2 1 ppm
1.574
1.686
3.137
1.162
1.193
1.298
2.498
5.986
9.765
1.709

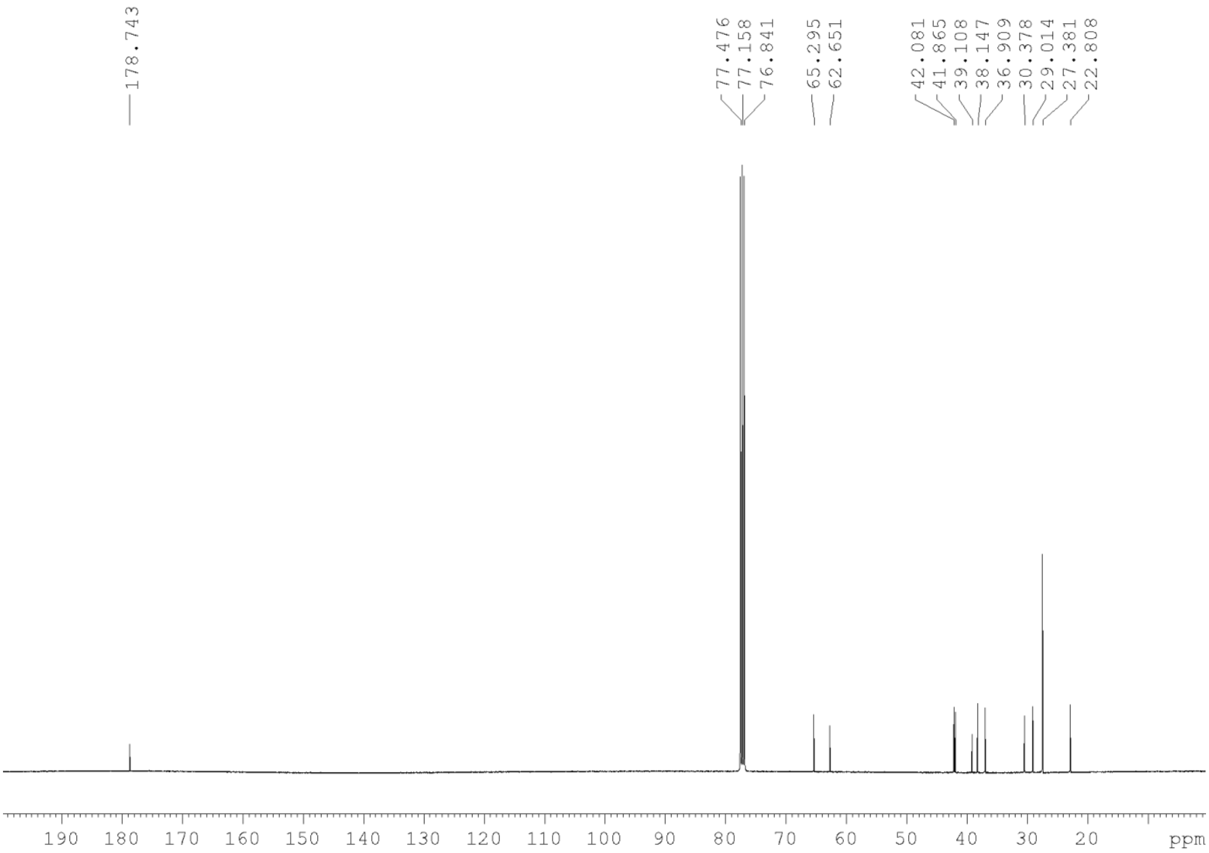

178.743
77.476
77.158
76.841
65.295
62.651
42.081
41.865
39.108
38.147
36.909
30.378
29.014
27.381
22.808
190 180 170 160 150 140 130 120 110 100 90 80 70 60 50 40 30 20 ppm

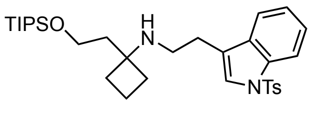
TIPSO
H
N
NTs

1x

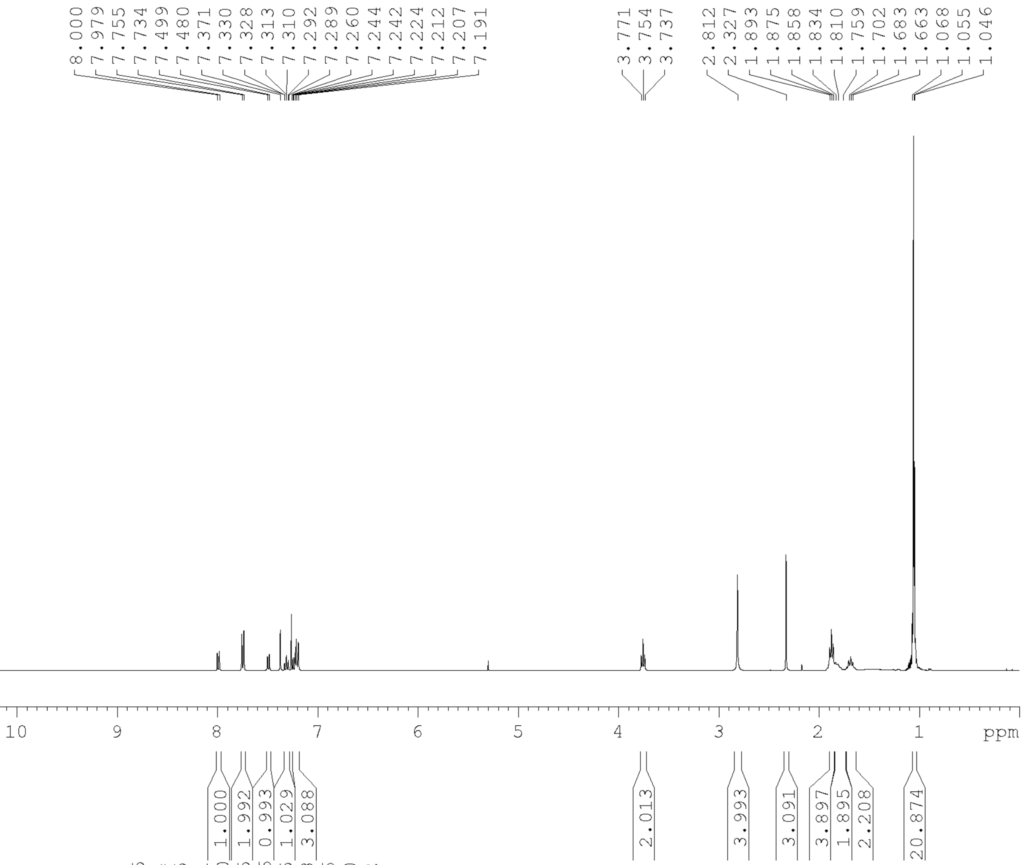
8.000
7.979
7.755
7.734
7.499
7.480
7.371
7.330
7.328
7.313
7.310
7.292
7.289
7.260
7.244
7.242
7.224
7.212
7.207
7.191
3.771
3.754
3.737
2.812
2.327
1.893
1.875
1.858
1.834
1.810
1.759
1.702
1.683
1.663
1.068
1.055
1.046
11
10
9
8
7
6
5
4
3
2
1
ppm
1.000
1.992
0.993
1.029
3.088
2.013
3.993
3.091
3.897
1.895
2.208
20.874

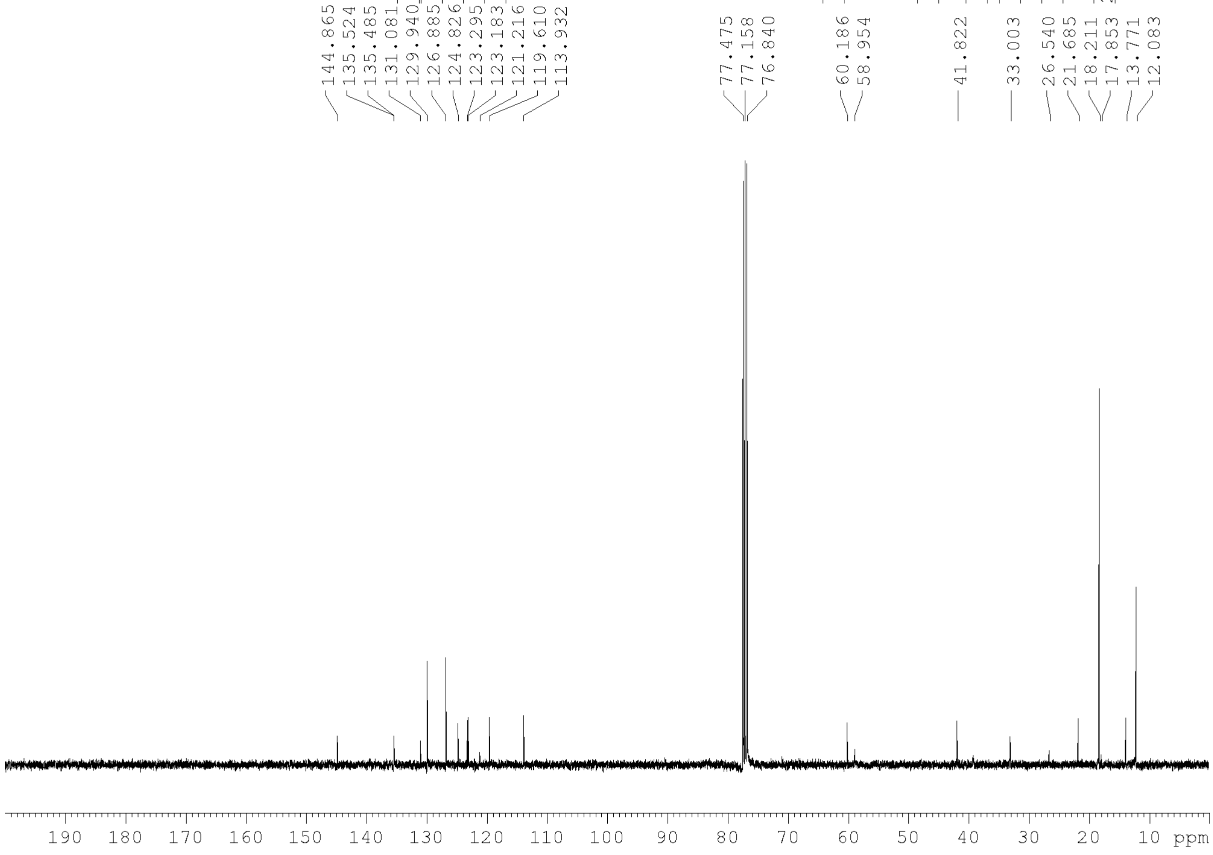
144.865
135.524
135.485
131.081
129.940
126.885
124.826
123.295
123.183
121.216
119.610
113.932
77.475
77.158
76.840
60.186
58.954
41.822
33.003
26.540
21.685
18.211
17.853
13.771
12.083
190
180
170
160
150
140
130
120
110
100
90
80
70
60
50
40
30
20
10
ppm

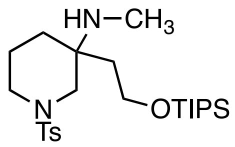

1y

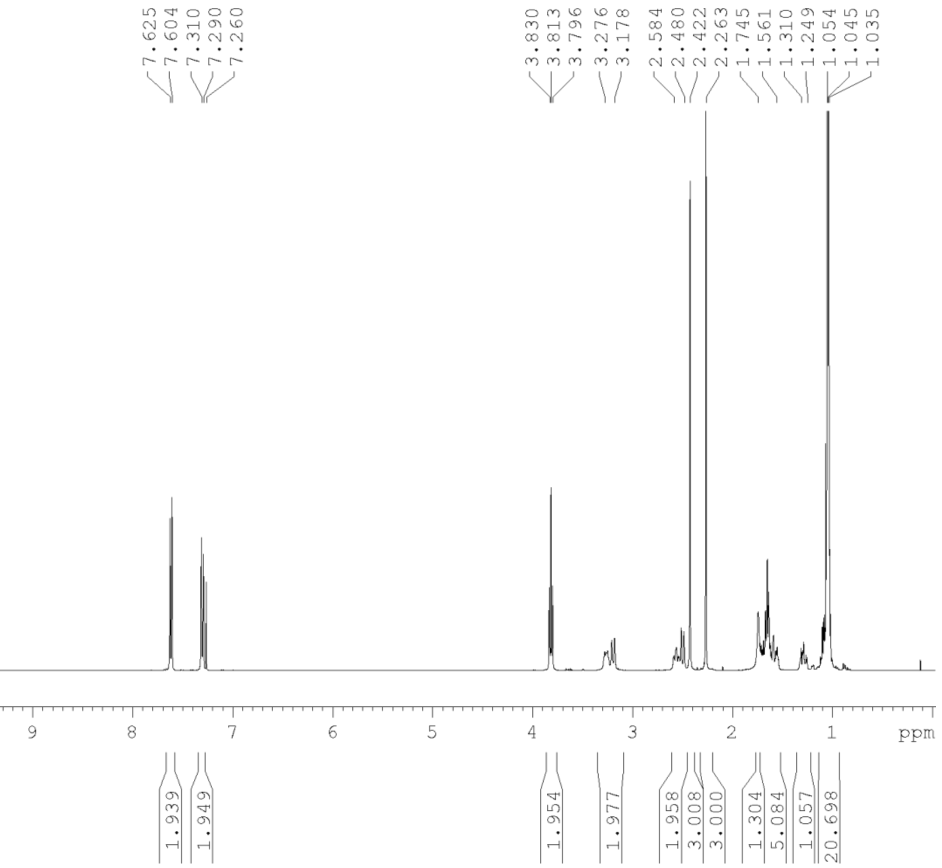
7.625
7.604
7.310
7.290
7.260
3.830
3.813
3.796
3.276
3.178
2.584
2.480
2.422
2.263
1.745
1.561
1.310
1.249
1.054
1.045
1.035
11 10 9 8 7 6 5 4 3 2 1 ppm
1.939
1.949
1.954
1.977
1.958
3.008
3.000
1.304
5.084
1.057
20.698

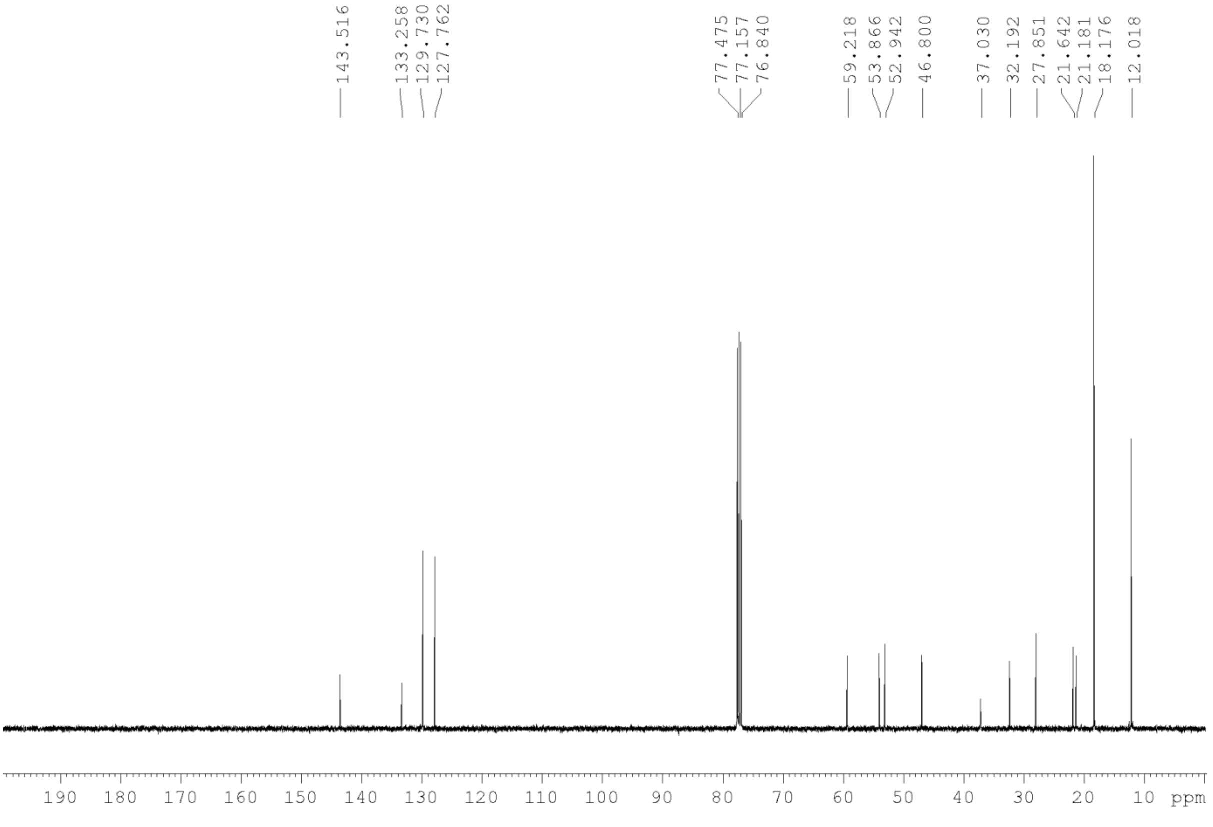
143.516
133.258
129.730
127.762
77.475
77.157
76.840
59.218
53.866
52.942
46.800
37.030
32.192
27.851
21.642
21.181
18.176
12.018
190 180 170 160 150 140 130 120 110 100 90 80 70 60 50 40 30 20 10 ppm

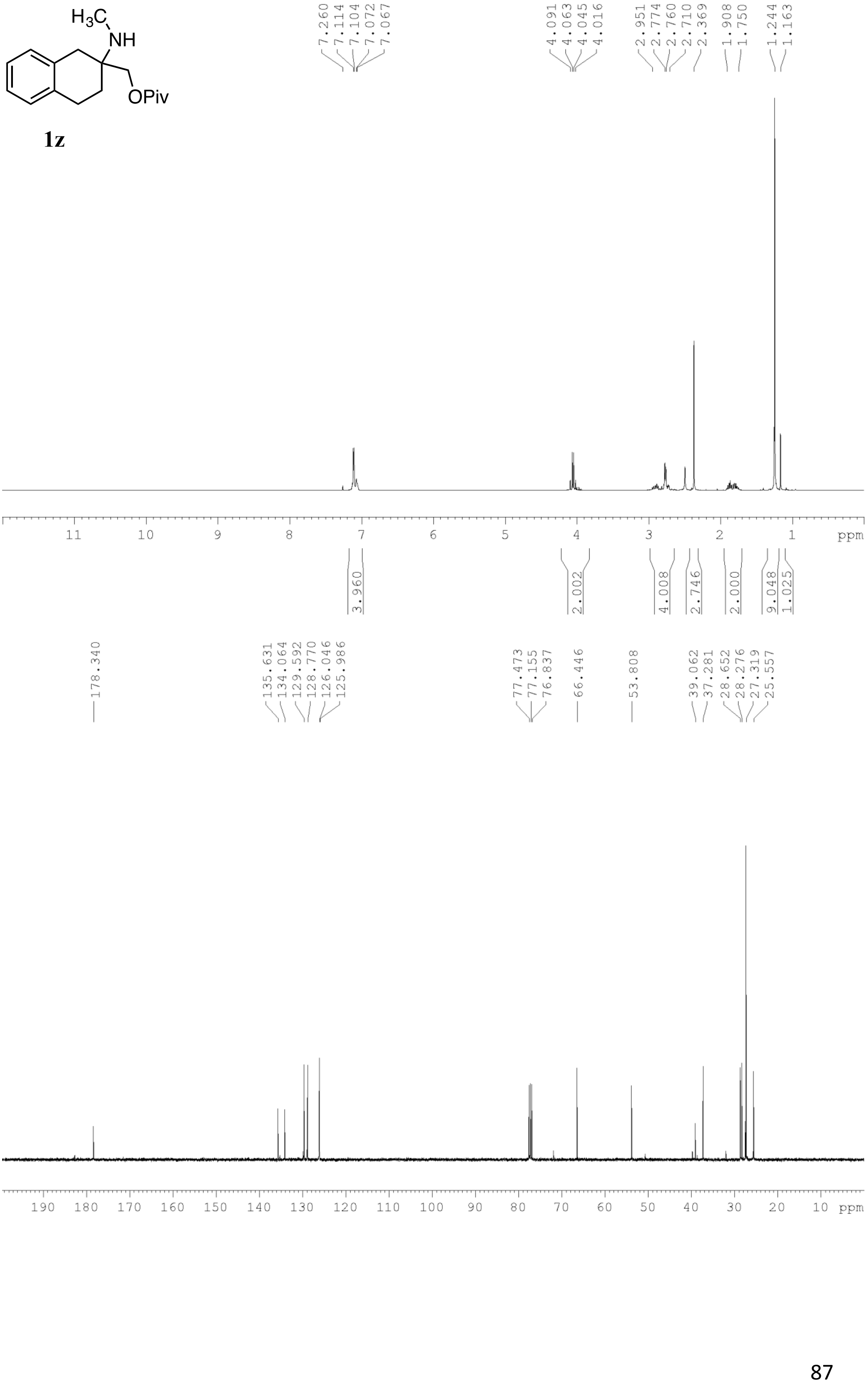
H3C
NH
OPiv
1z
7.260 7.114 7.104 7.072 7.067
4.091 4.063 4.045 4.016
2.951 2.774 2.760 2.710 2.369
1.908 1.750
1.244 1.163
11 10 9 8 7 6 5 4 3 2 1 ppm
3.960 2.002 4.008 2.746 2.000 9.048 1.025
178.340
135.631 134.064 129.592 128.770 126.046 125.986
77.473 77.155 76.837
66.446
53.808
39.062 37.281 28.652 28.276 27.319 25.557
190 180 170 160 150 140 130 120 110 100 90 80 70 60 50 40 30 20 10 ppm

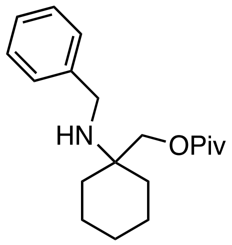
HN
OPiv

1aa

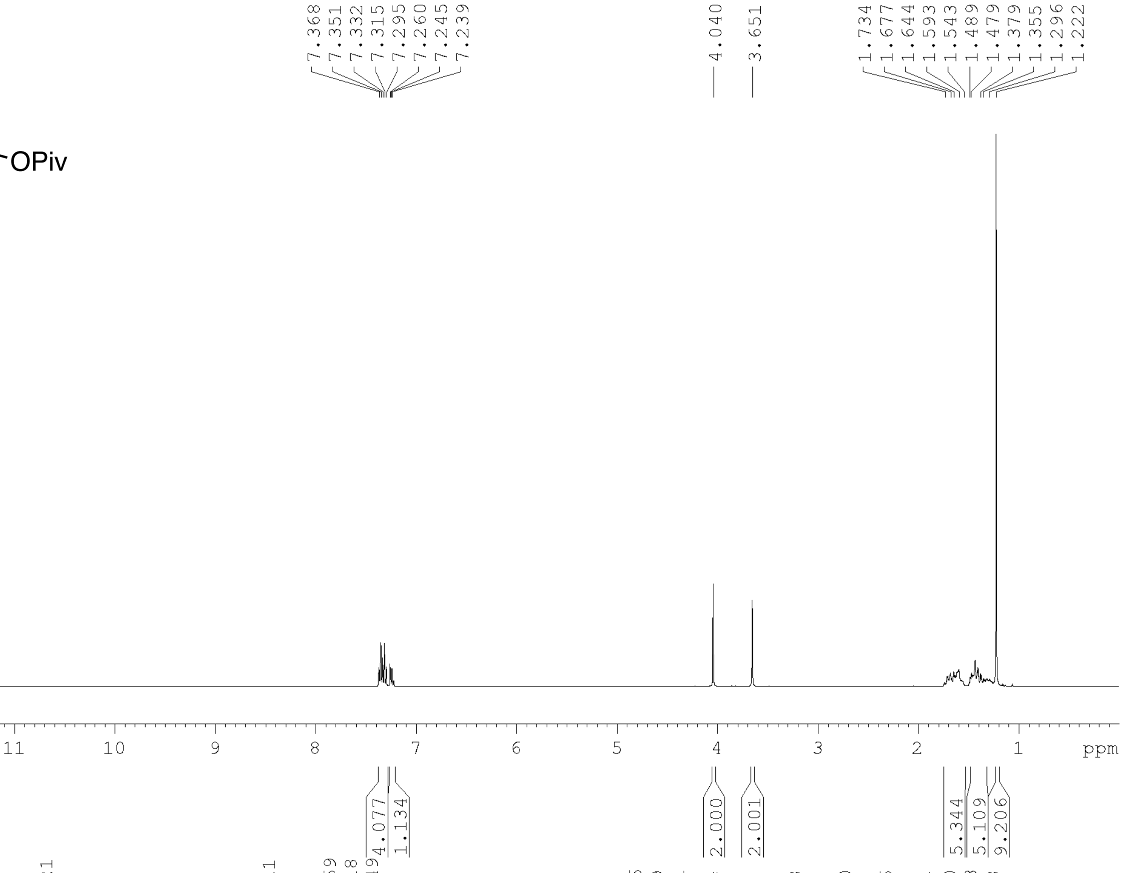
7.368
7.351
7.332
7.315
7.295
7.260
7.245
7.239
4.040
3.651
1.734
1.677
1.644
1.593
1.543
1.489
1.479
1.379
1.355
1.296
1.222
11 10 9 8 7 6 5 4 3 2 1 ppm
4.077
1.134
2.000
2.001
5.344
5.109
9.206

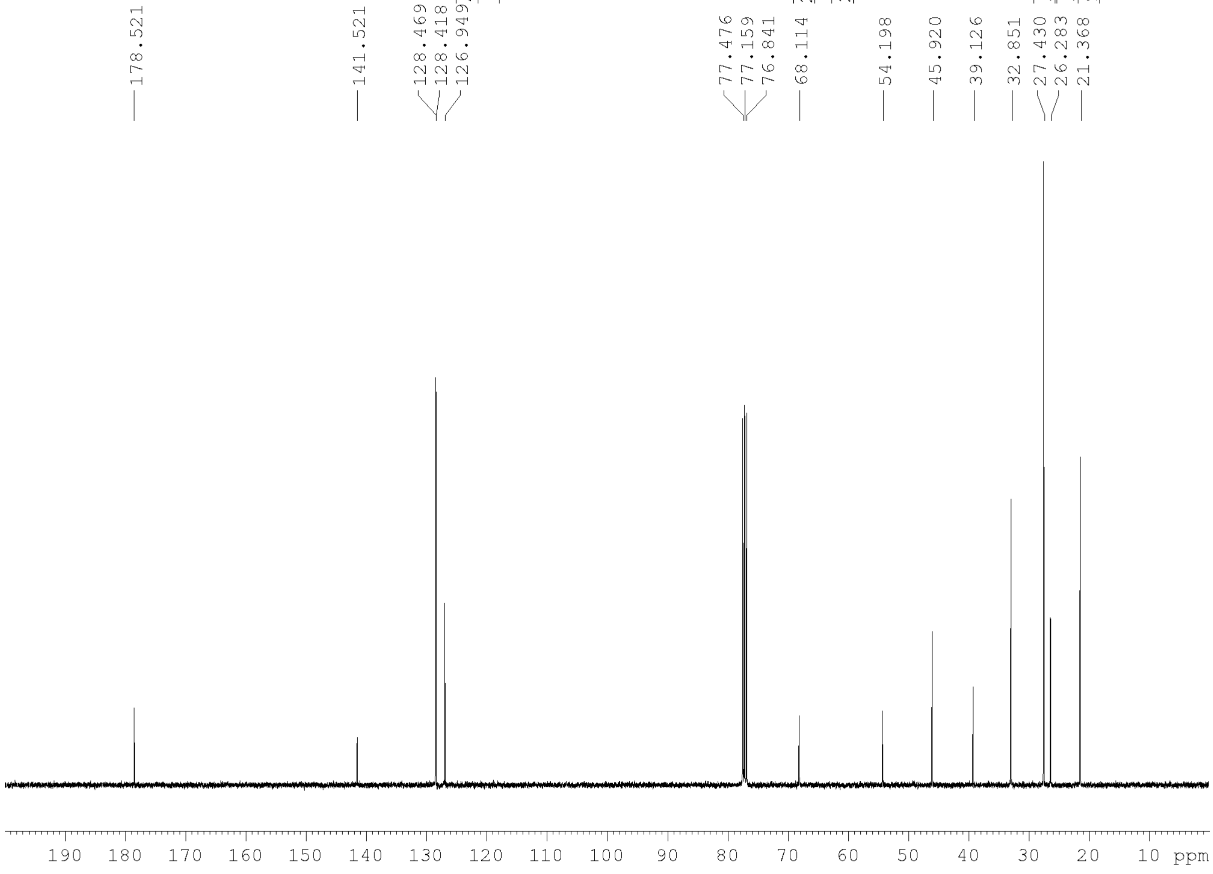
178.521
141.521
128.469
128.418
126.949
77.476
77.159
76.841
68.114
54.198
45.920
39.126
32.851
27.430
26.283
21.368
190 180 170 160 150 140 130 120 110 100 90 80 70 60 50 40 30 20 10 ppm

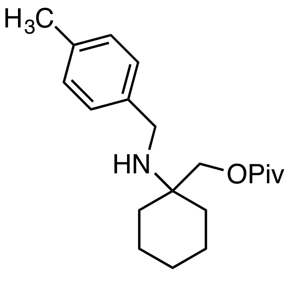

1ab

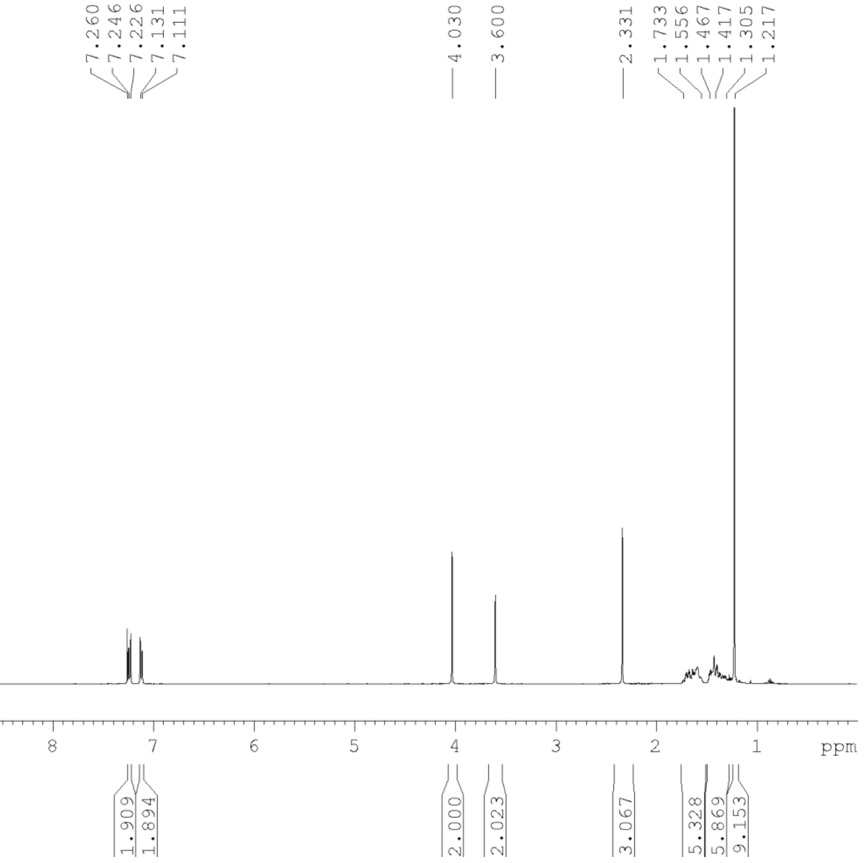
7.260
7.246
7.226
7.131
7.111
4.030
3.600
2.331
1.733
1.556
1.467
1.417
1.305
1.217
11 10 9 8 7 6 5 4 3 2 1 ppm
1.909
1.894
2.000
2.023
3.067
5.328
5.869
9.153

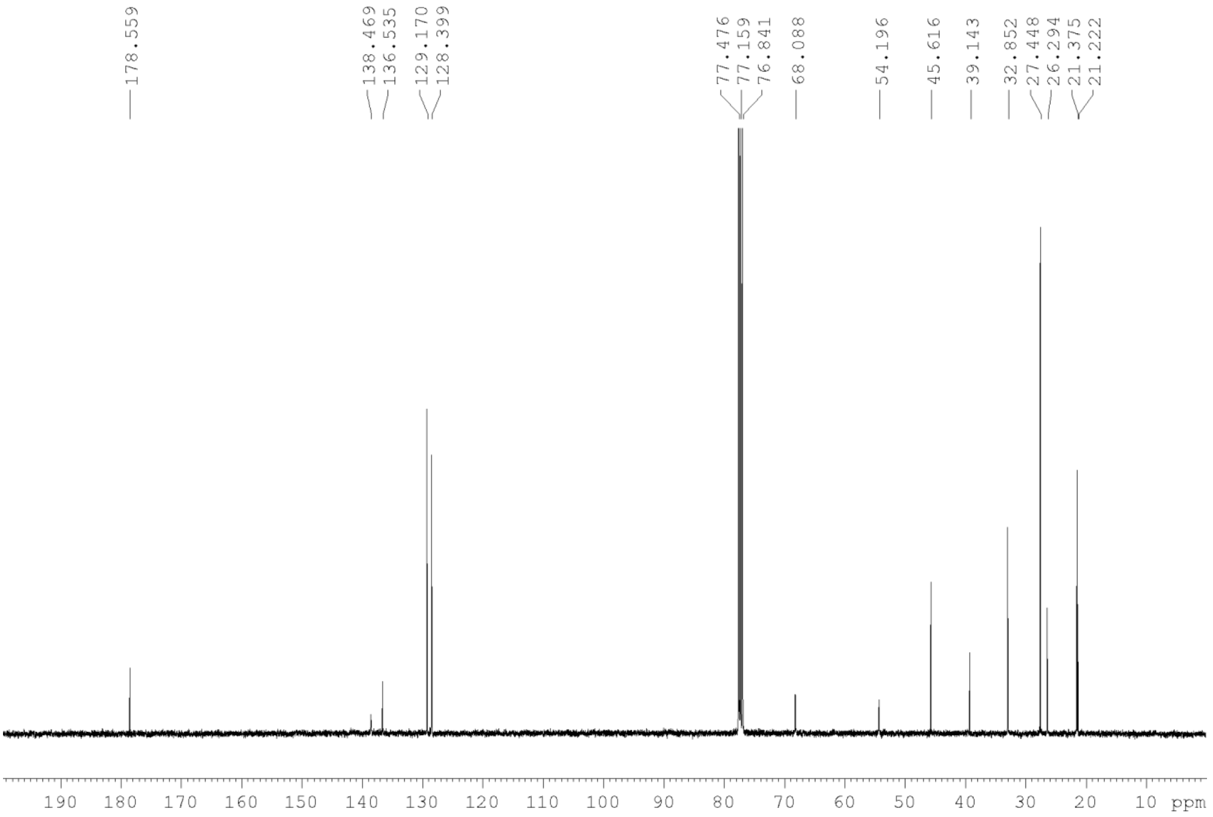
178.559
138.469
136.535
129.170
128.399
77.476
77.159
76.841
68.088
54.196
45.616
39.143
32.852
27.448
26.294
21.375
21.222
190 180 170 160 150 140 130 120 110 100 90 80 70 60 50 40 30 20 10 ppm

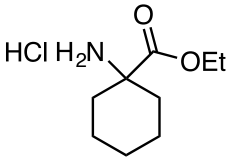
HCl H2N
O
OEt

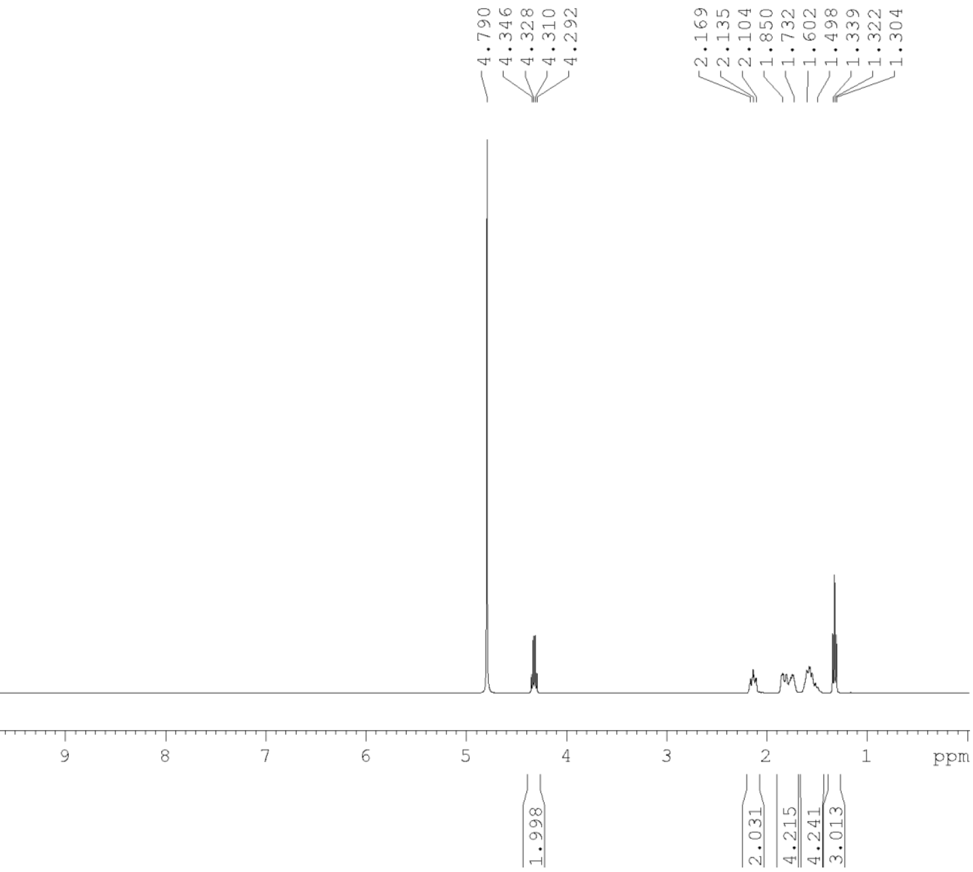
4.790
4.346
4.328
4.310
4.292
2.169
2.135
2.104
1.850
1.732
1.602
1.498
1.339
1.322
1.304
1.998
2.031
4.215
4.241
3.013
ppm

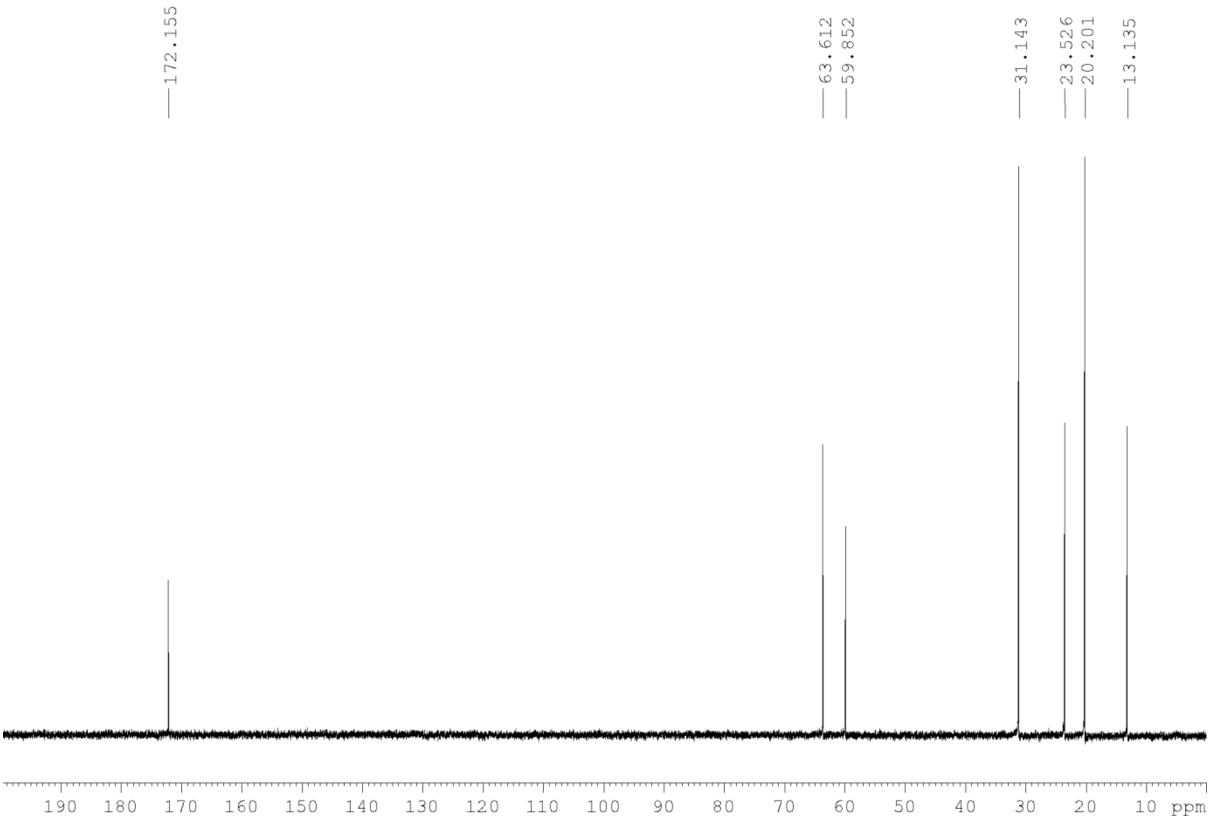
172.155
63.612
59.852
31.143
23.526
20.201
13.135
ppm

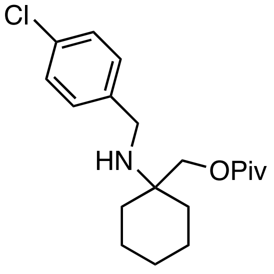
Cl
HN
OPiv

1ac

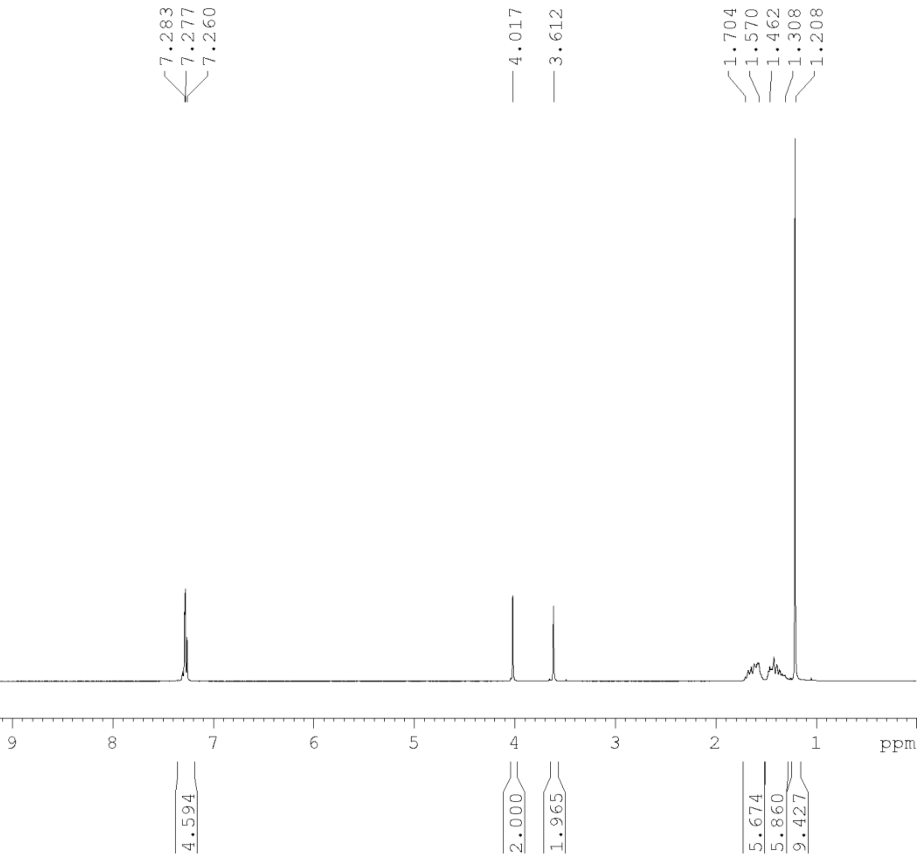
7.283
7.277
7.260
4.017
3.612
1.704
1.570
1.462
1.308
1.208
11
10
9
8
7
6
5
4
3
2
1
ppm
4.594
2.000
1.965
5.674
5.860
9.427

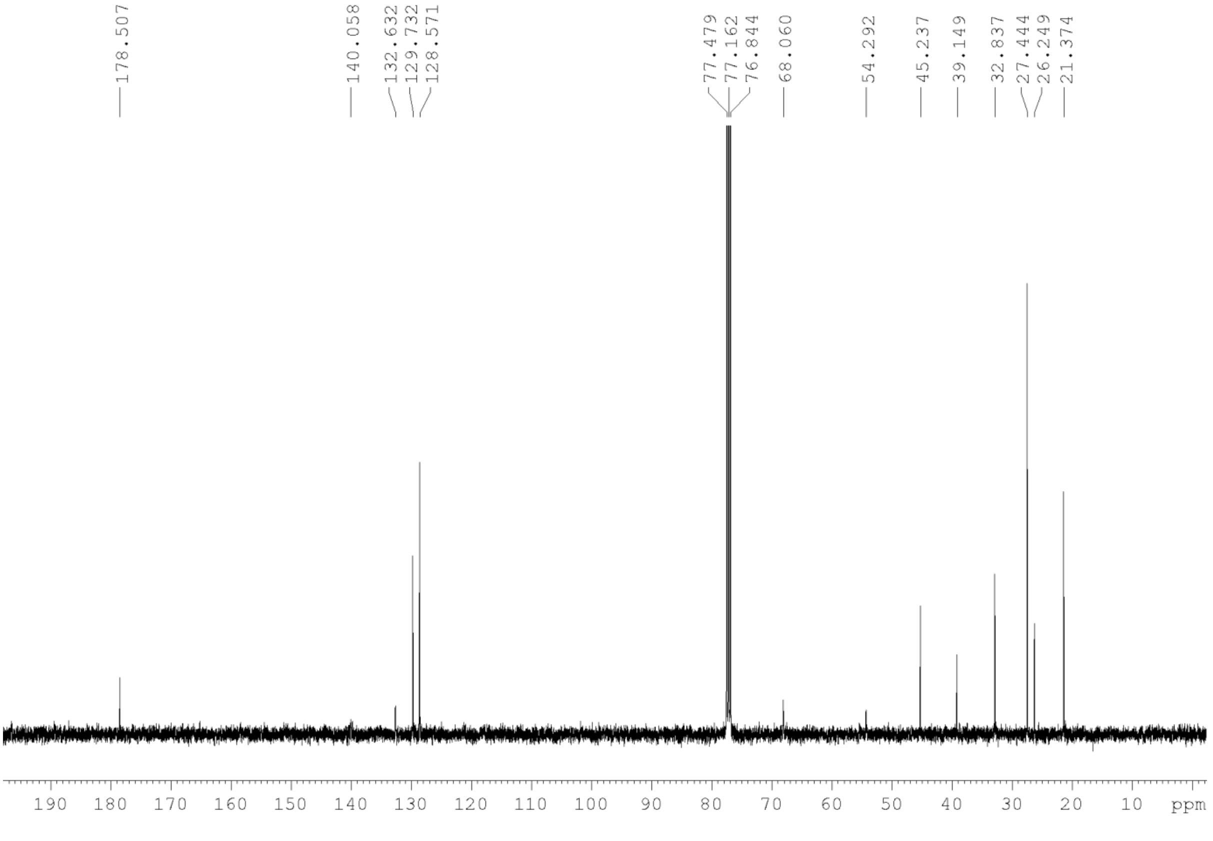
178.507
140.058
132.632
129.732
128.571
77.479
77.162
76.844
68.060
54.292
45.237
39.149
32.837
27.444
26.249
21.374
190
180
170
160
150
140
130
120
110
100
90
80
70
60
50
40
30
20
10
ppm

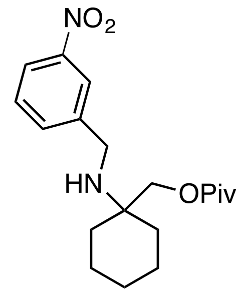
NO2
HN
OPiv

1ad

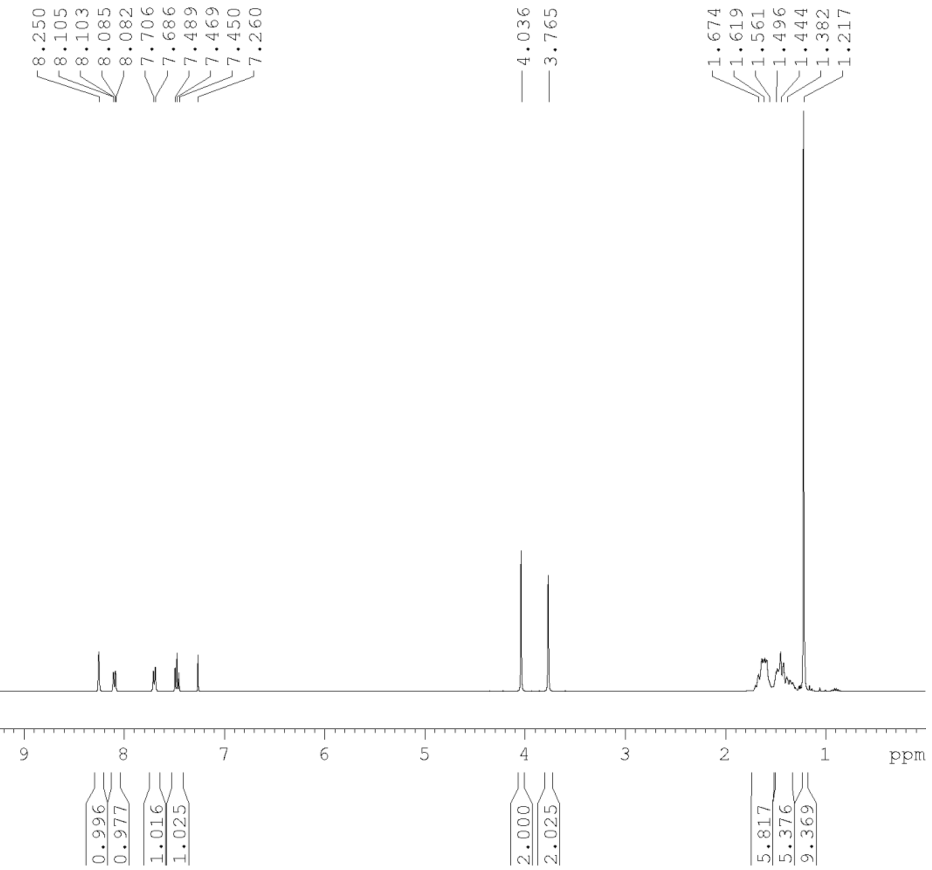
8.250
8.105
8.103
8.085
8.082
7.706
7.686
7.489
7.469
7.450
7.260
4.036
3.765
1.674
1.619
1.561
1.496
1.444
1.382
1.217
11 10 9 8 7 6 5 4 3 2 1 ppm
0.996
0.977
1.016
1.025
2.000
2.025
5.817
5.376
9.369

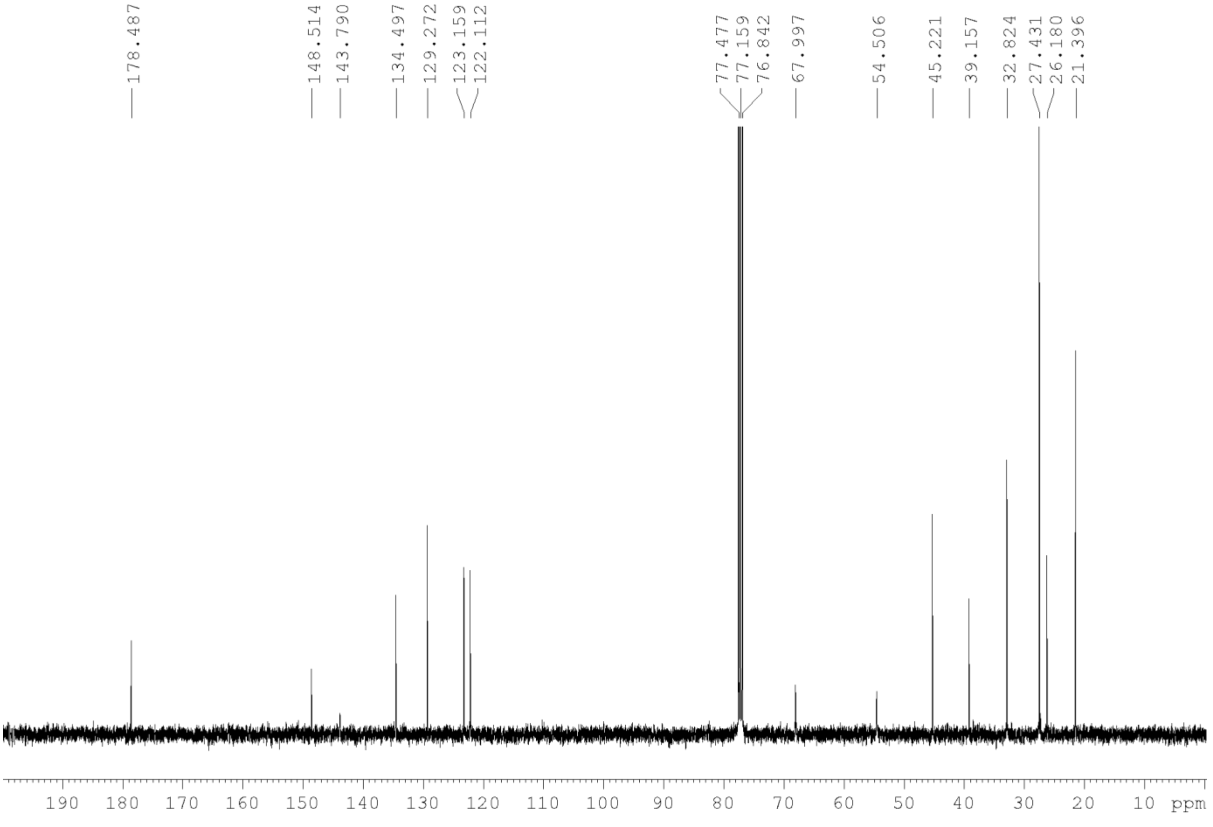
178.487
148.514
143.790
134.497
129.272
123.159
122.112
77.477
77.159
76.842
67.997
54.506
45.221
39.157
32.824
27.431
26.180
21.396
190 180 170 160 150 140 130 120 110 100 90 80 70 60 50 40 30 20 10 ppm

NMR of β-Lactam Products

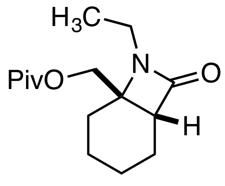
H3C
N
O
PivO
H

2a

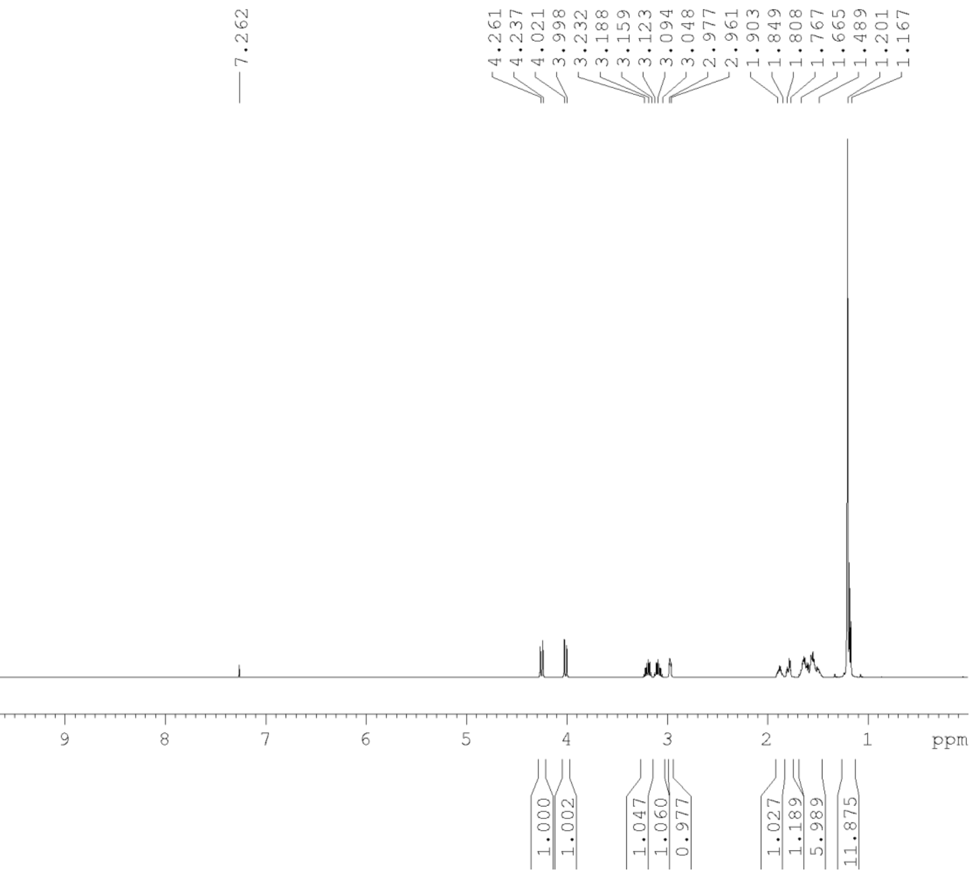
7.262
4.261
4.237
4.021
3.998
3.232
3.188
3.159
3.123
3.094
3.048
2.977
2.961
1.903
1.849
1.808
1.767
1.665
1.489
1.201
1.167
11 10 9 8 7 6 5 4 3 2 1 ppm
1.000
1.002
1.047
1.060
0.977
1.027
1.189
5.989
11.875

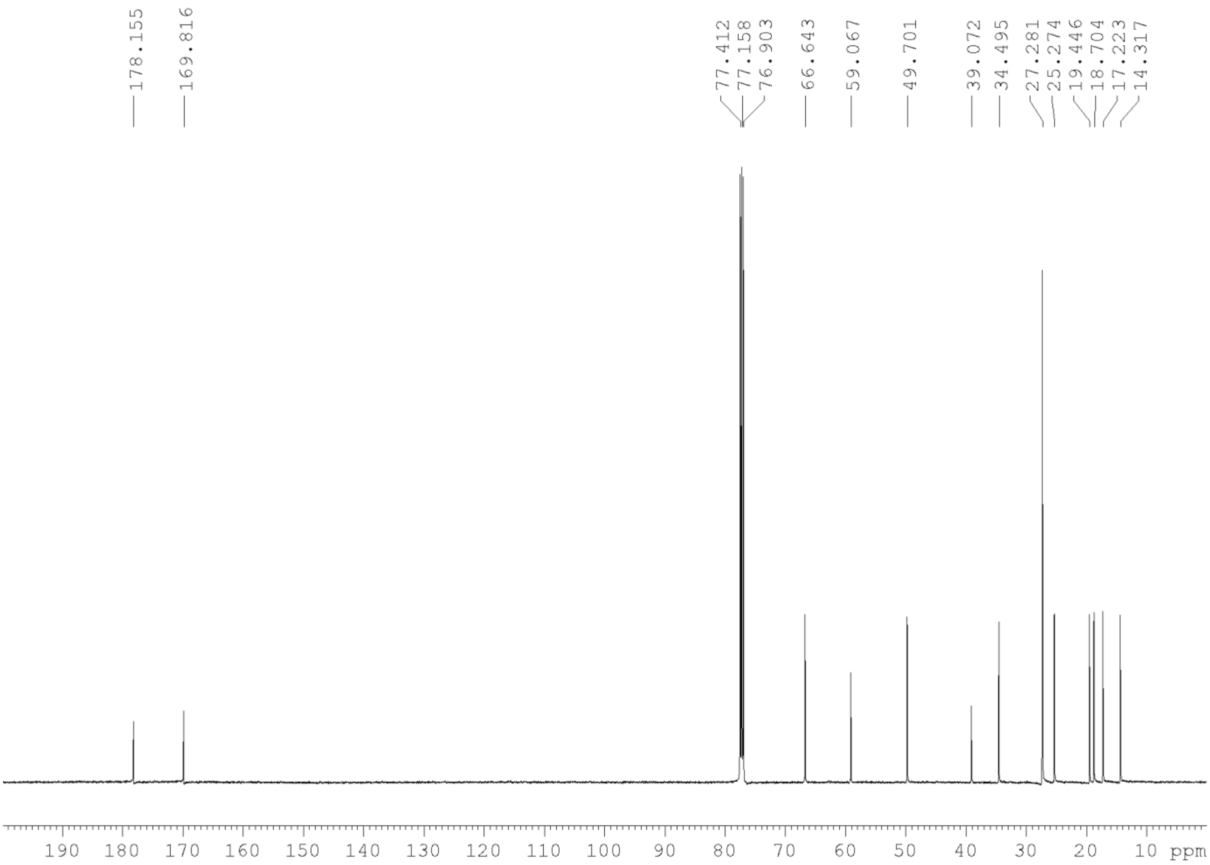
178.155
169.816
77.412
77.158
76.903
66.643
59.067
49.701
39.072
34.495
27.281
25.274
19.446
18.704
17.223
14.317
190 180 170 160 150 140 130 120 110 100 90 80 70 60 50 40 30 20 10 ppm

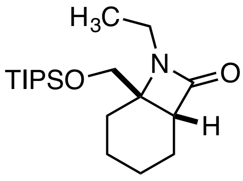
H3C
N
O
TIPSO
H

2b

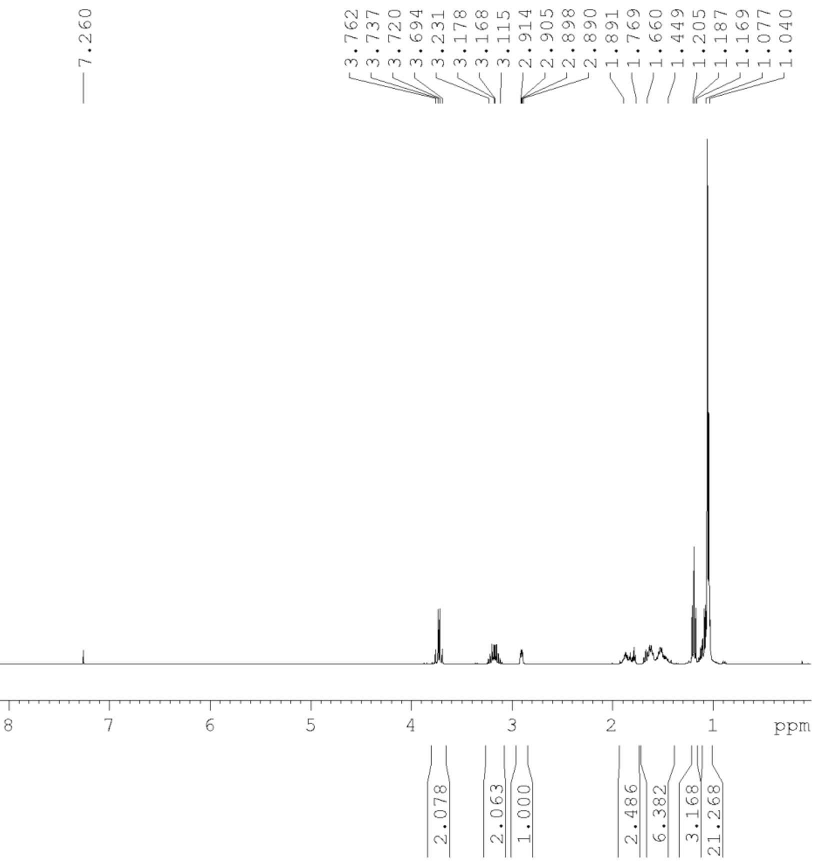
7.260
3.762
3.737
3.720
3.694
3.231
3.178
3.168
3.115
2.914
2.905
2.898
2.890
1.891
1.769
1.660
1.449
1.205
1.187
1.169
1.077
1.040
11 10 9 8 7 6 5 4 3 2 1 ppm
2.078
2.063
1.000
2.486
6.382
3.168
21.268

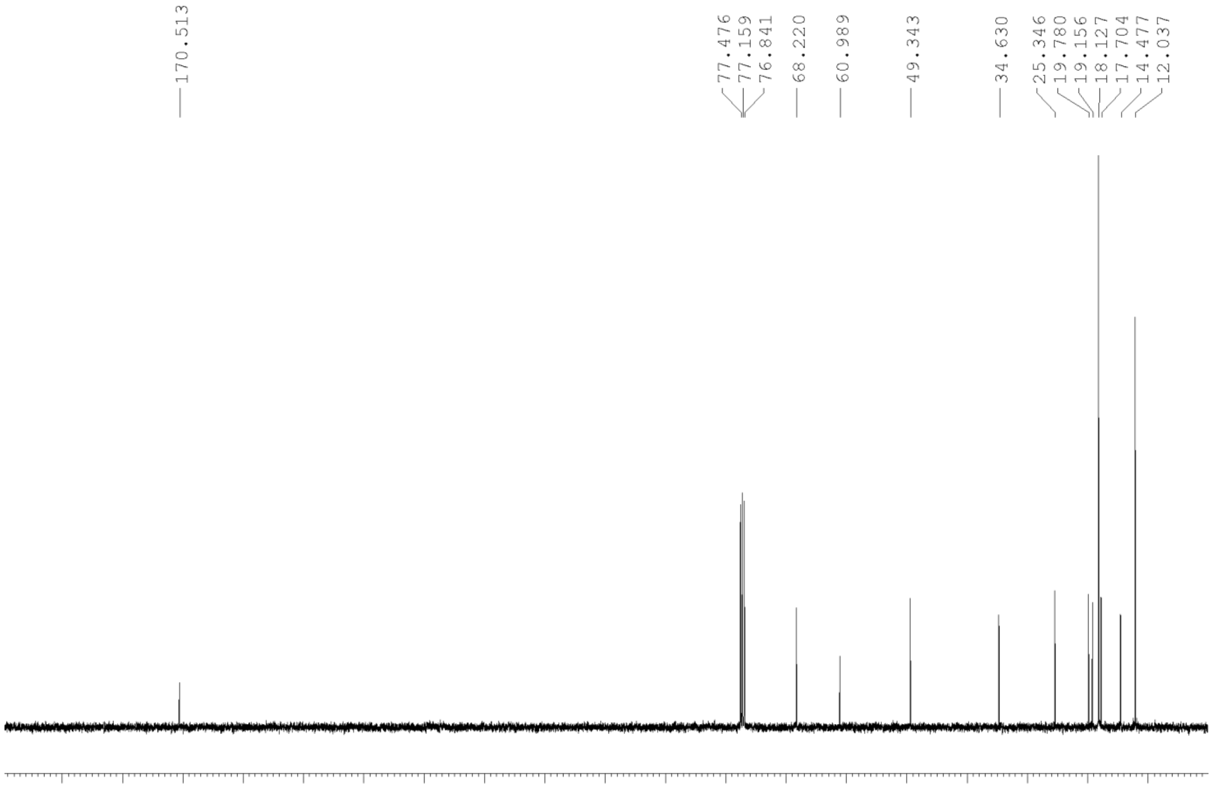
170.513
77.476
77.159
76.841
68.220
60.989
49.343
34.630
25.346
19.780
19.156
18.127
17.704
14.477
12.037
190 180 170 160 150 140 130 120 110 100 90 80 70 60 50 40 30 20 10 ppm

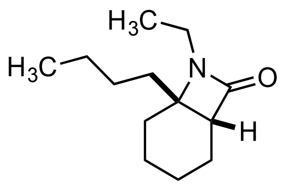

H_3C
H_3C
N
O
H

2c

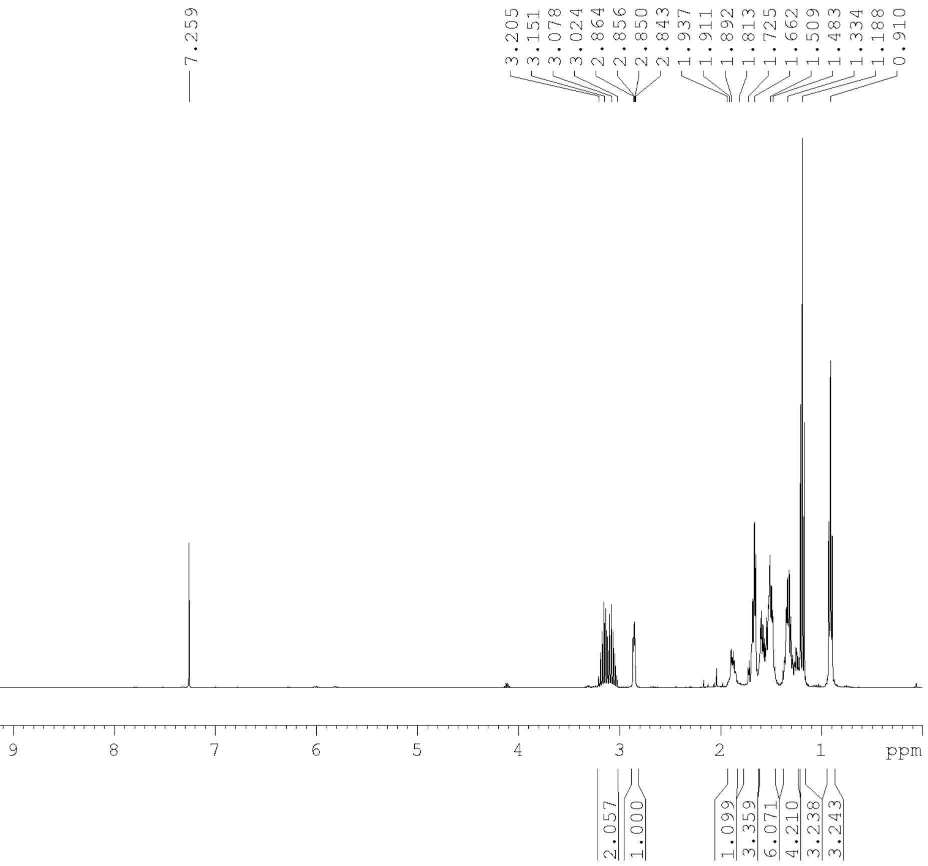

7.259
3.205
3.151
3.078
3.024
2.864
2.856
2.850
2.843
1.937
1.911
1.892
1.813
1.725
1.662
1.509
1.483
1.334
1.188
0.910
11 10 9 8 7 6 5 4 3 2 1 ppm
2.057
1.000
1.099
3.359
6.071
4.210
3.238
3.243

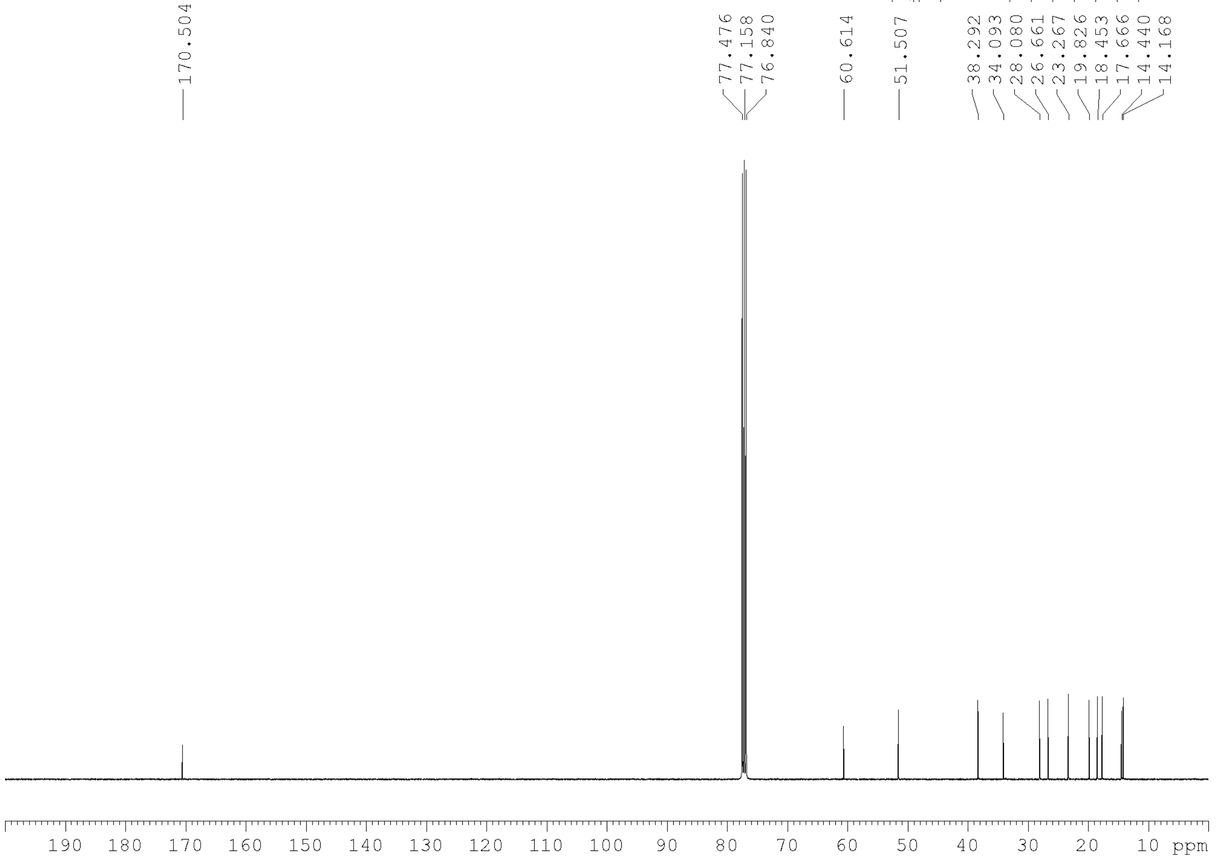

170.504
77.476
77.158
76.840
60.614
51.507
38.292
34.093
28.080
26.661
23.267
19.826
18.453
17.666
14.440
14.168
190 180 170 160 150 140 130 120 110 100 90 80 70 60 50 40 30 20 10 ppm

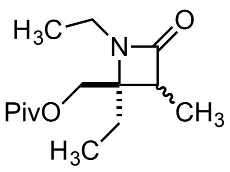
H3C
N
O
PivO
CH3
H3C

2d

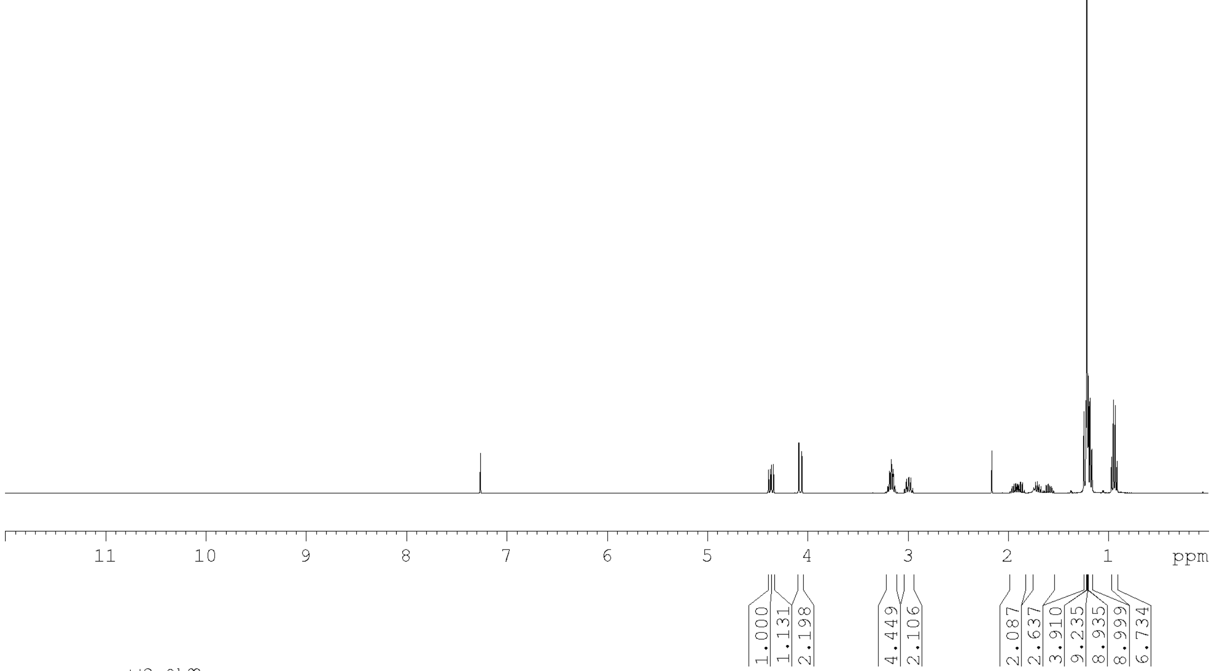
7.260
4.388
4.358
4.085
4.055
3.200
3.163
3.126
3.032
3.012
2.993
2.969
2.950
1.978
1.837
1.744
1.546
1.216
1.210
0.949
0.931
11 10 9 8 7 6 5 4 3 2 1 ppm
1.000
1.131
2.198
4.449
2.106
2.087
2.637
3.910
9.235
8.935
8.999
6.734

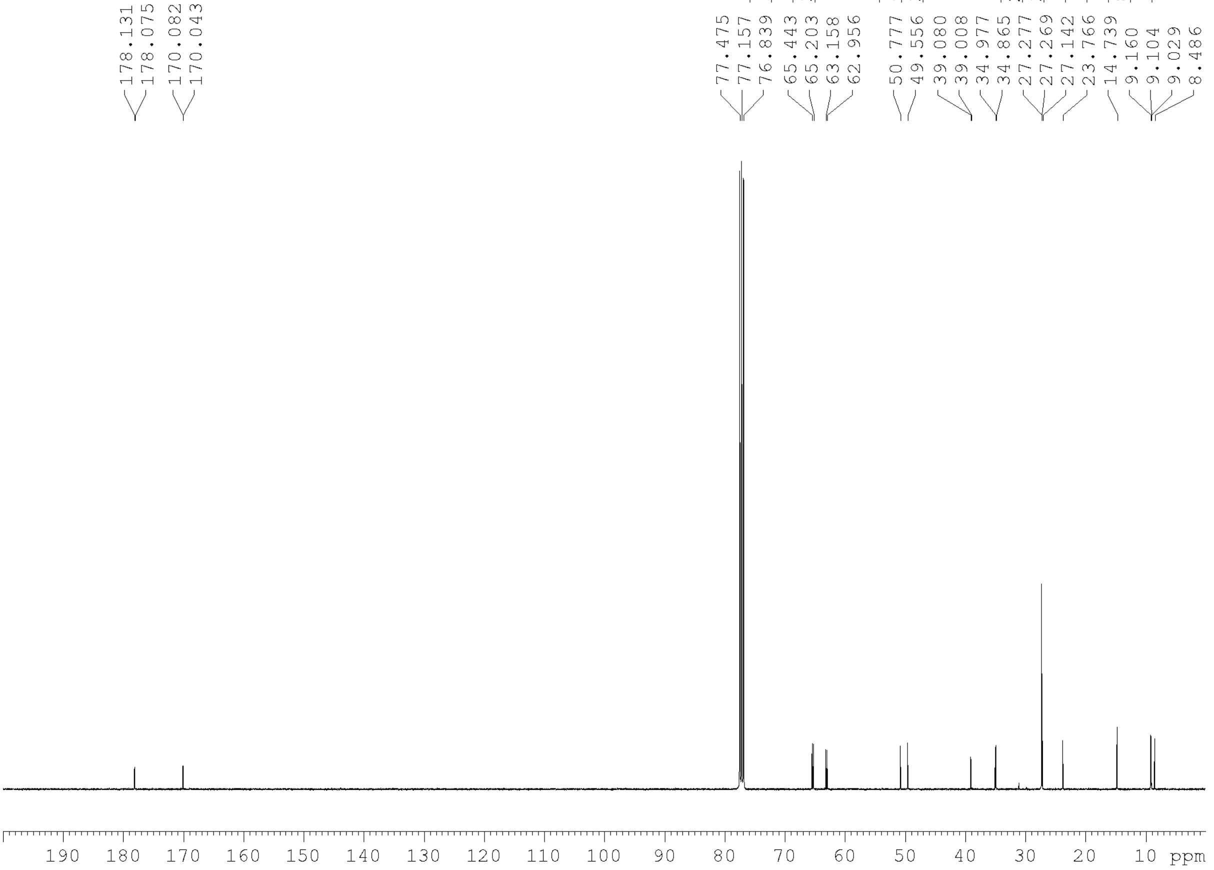
178.131
178.075
170.082
170.043
77.475
77.157
76.839
65.443
65.203
63.158
62.956
50.777
49.556
39.080
39.008
34.977
34.865
27.277
27.269
27.142
23.766
14.739
9.160
9.104
9.029
8.486
190 180 170 160 150 140 130 120 110 100 90 80 70 60 50 40 30 20 10 ppm

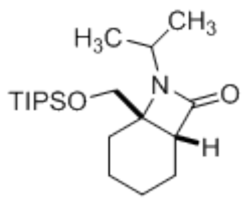
H3C
CH3
N
TIPSO
O
H

2e

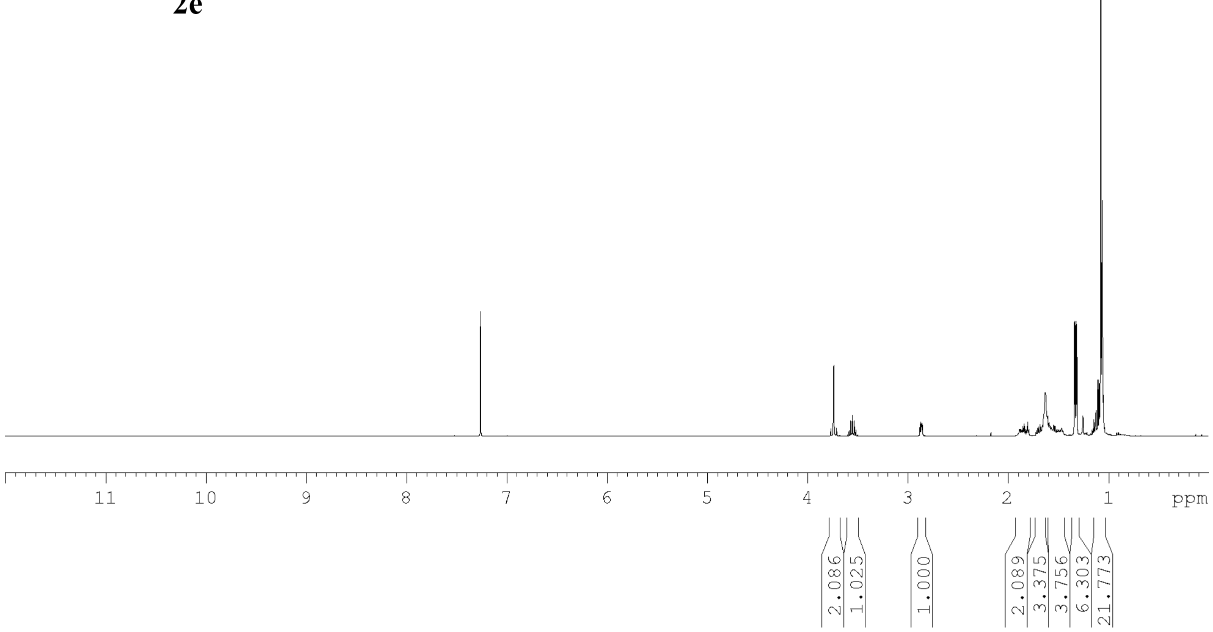
7.260
3.766 3.740 3.735 3.709 3.601 3.584 3.567 3.550 3.533 3.516 3.499 2.876 2.867 2.860 2.852 1.883 1.717 1.655 1.449 1.337 1.330 1.320 1.313 1.073
11 10 9 8 7 6 5 4 3 2 1 ppm
2.086 1.025 1.000 2.089 3.375 3.756 6.303 21.773

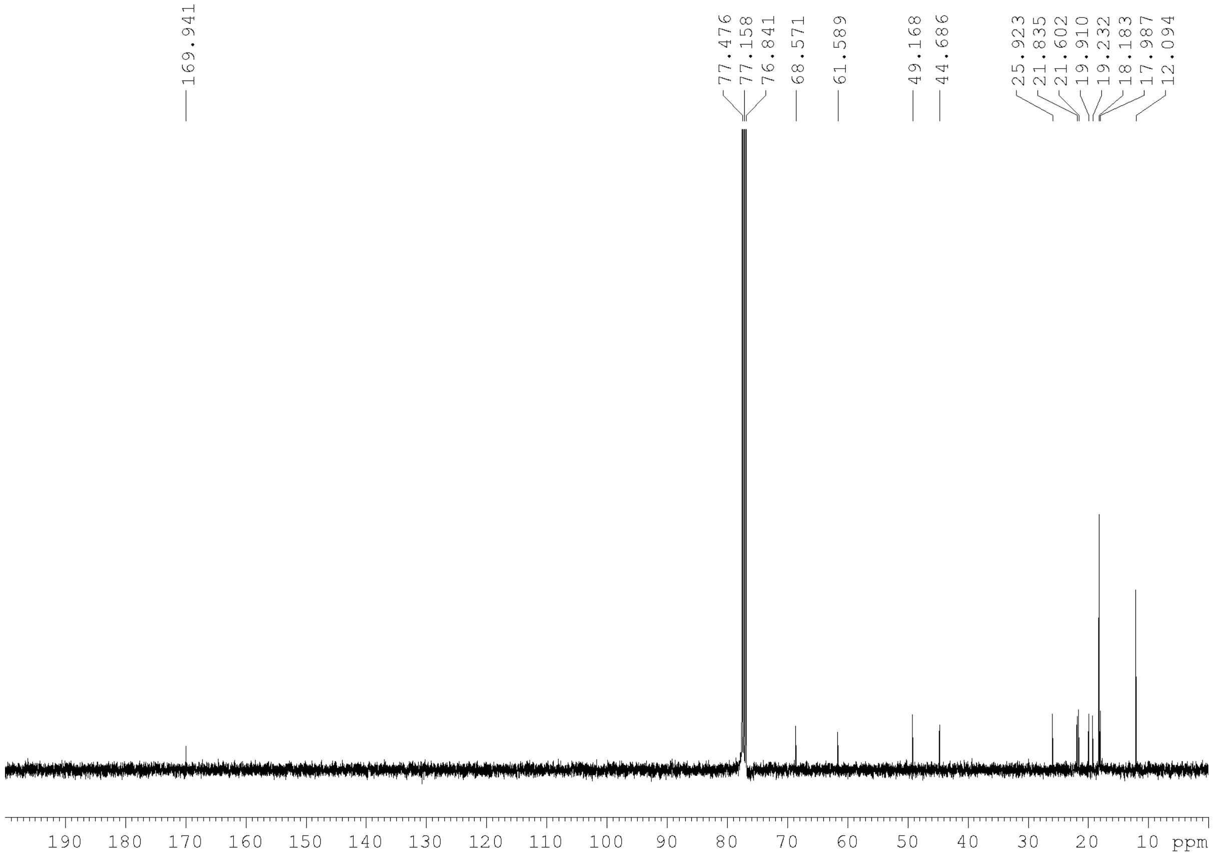
169.941
77.476 77.158 76.841 68.571 61.589 49.168 44.686 25.923 21.835 21.602 19.910 19.232 18.183 17.987 12.094
190 180 170 160 150 140 130 120 110 100 90 80 70 60 50 40 30 20 10 ppm

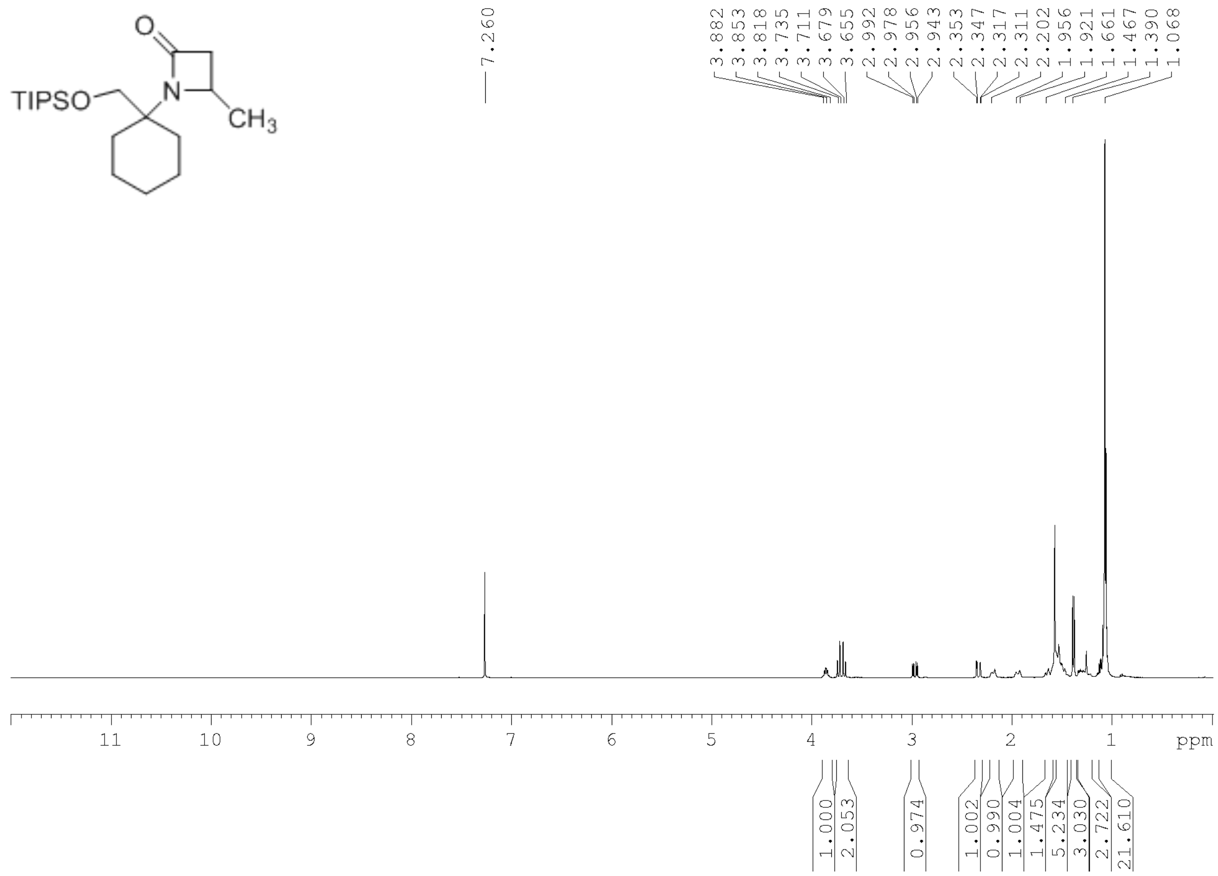
TIPSO
O
N
CH3
7.260
3.882 3.853 3.818 3.735 3.711 3.679 3.655 2.992 2.978 2.956 2.943 2.353 2.347 2.317 2.311 2.202 1.956 1.921 1.661 1.467 1.390 1.068
11 10 9 8 7 6 5 4 3 2 1 ppm
1.000 2.053 0.974 1.002 0.990 1.004 1.475 5.234 3.030 2.722 21.610

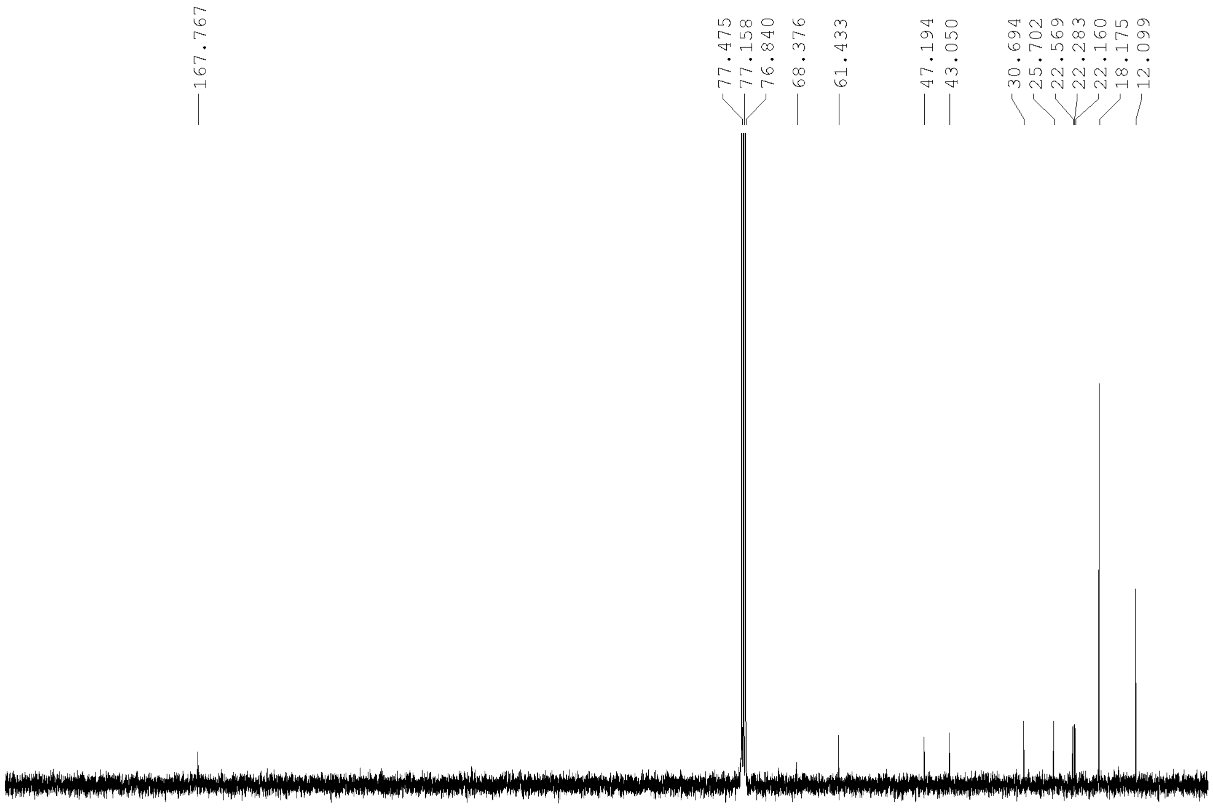
167.767
77.475 77.158 76.840 68.376 61.433 47.194 43.050 30.694 25.702 22.569 22.283 22.160 18.175 12.099
190 180 170 160 150 140 130 120 110 100 90 80 70 60 50 40 30 20 10 ppm

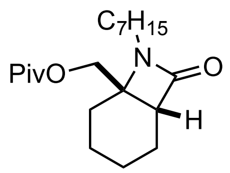
C7H15
PivO
N
O
H

2f

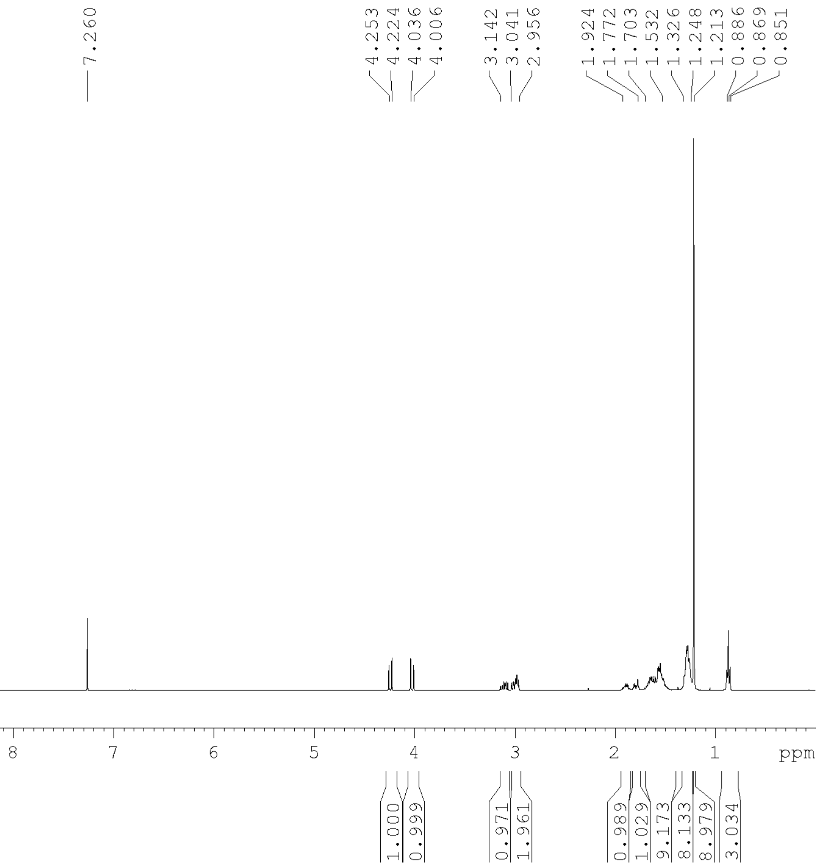
7.260
4.253
4.224
4.036
4.006
3.142
3.041
2.956
1.924
1.772
1.703
1.532
1.326
1.248
1.213
0.886
0.869
0.851
11 10 9 8 7 6 5 4 3 2 1 ppm
1.000
0.999
0.971
1.961
0.989
1.029
9.173
8.133
8.979
3.034

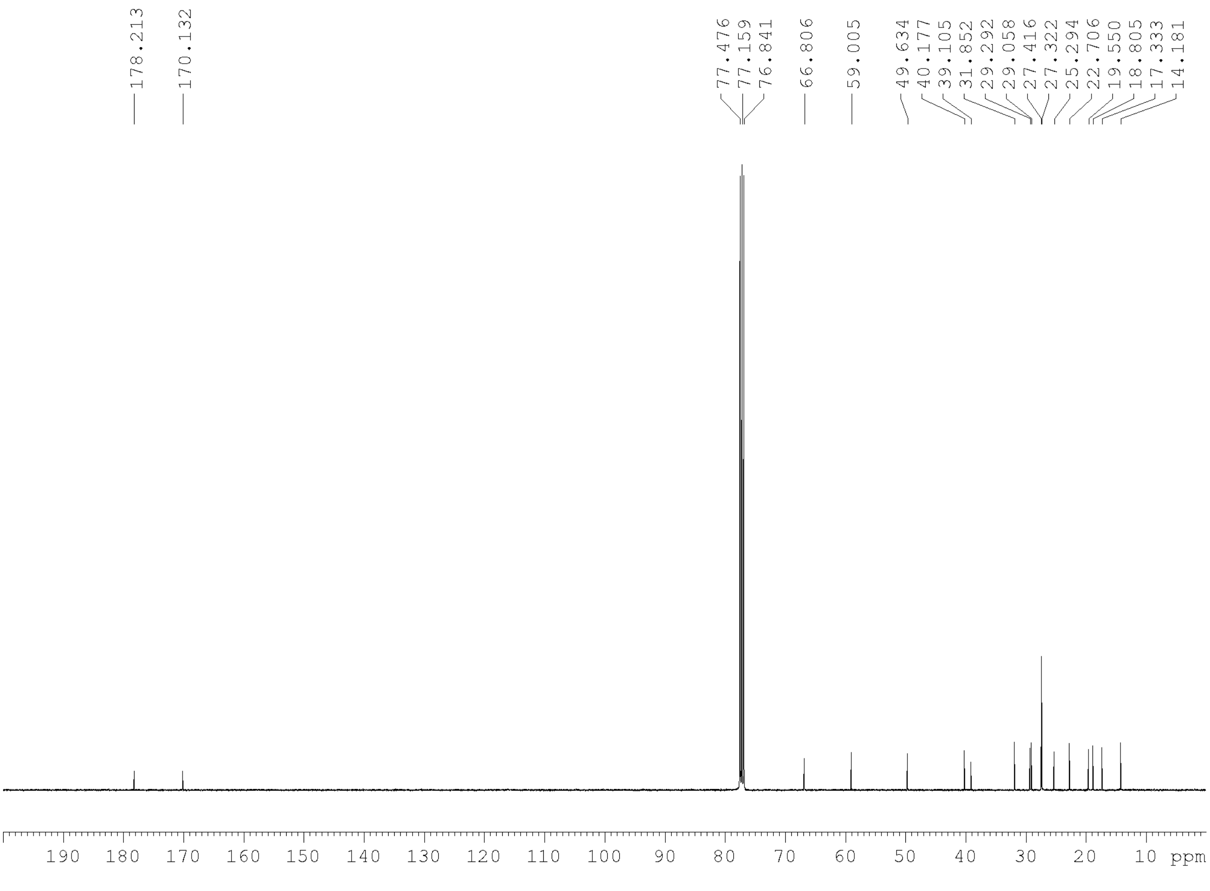
178.213
170.132
77.476
77.159
76.841
66.806
59.005
49.634
40.177
39.105
31.852
29.292
29.058
27.416
27.322
25.294
22.706
19.550
18.805
17.333
14.181
190 180 170 160 150 140 130 120 110 100 90 80 70 60 50 40 30 20 10 ppm

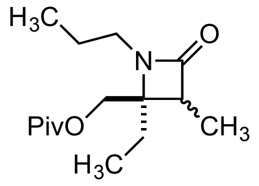
H3C
N
O
PivO
CH3
H3C

2g

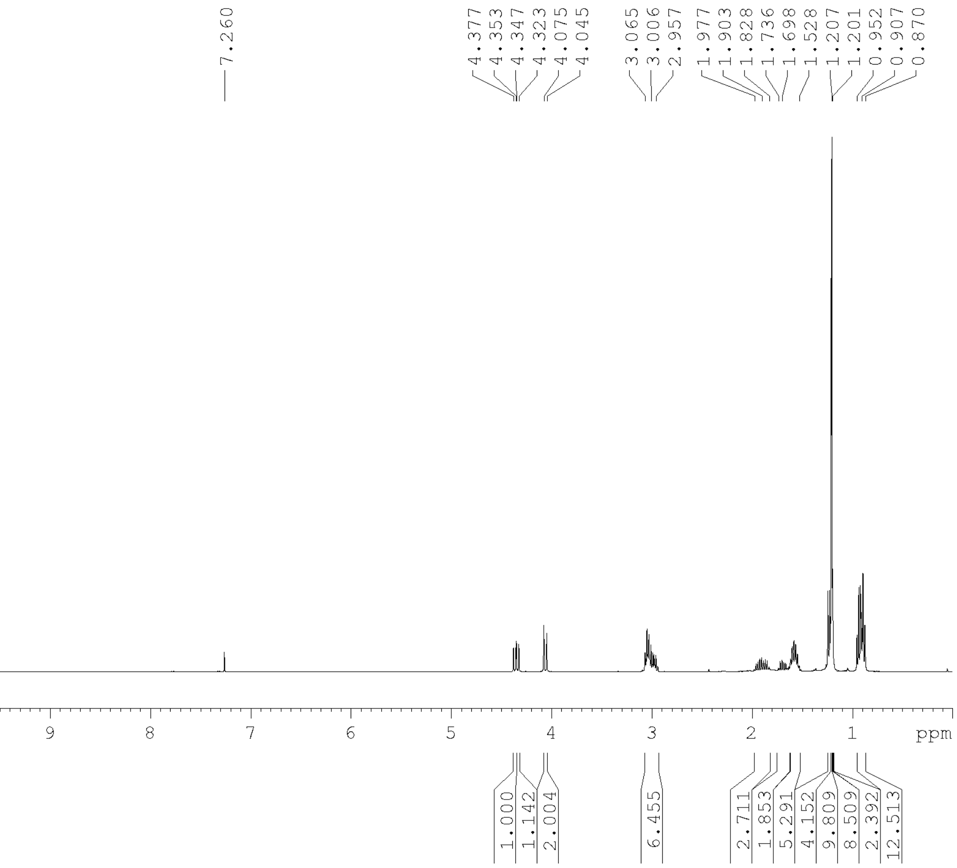
7.260
4.377
4.353
4.347
4.323
4.075
4.045
3.065
3.006
2.957
1.977
1.903
1.828
1.736
1.698
1.528
1.207
1.201
0.952
0.907
0.870
11 10 9 8 7 6 5 4 3 2 1 ppm
1.000
1.142
2.004
6.455
2.711
1.853
5.291
4.152
9.809
8.509
2.392
12.513

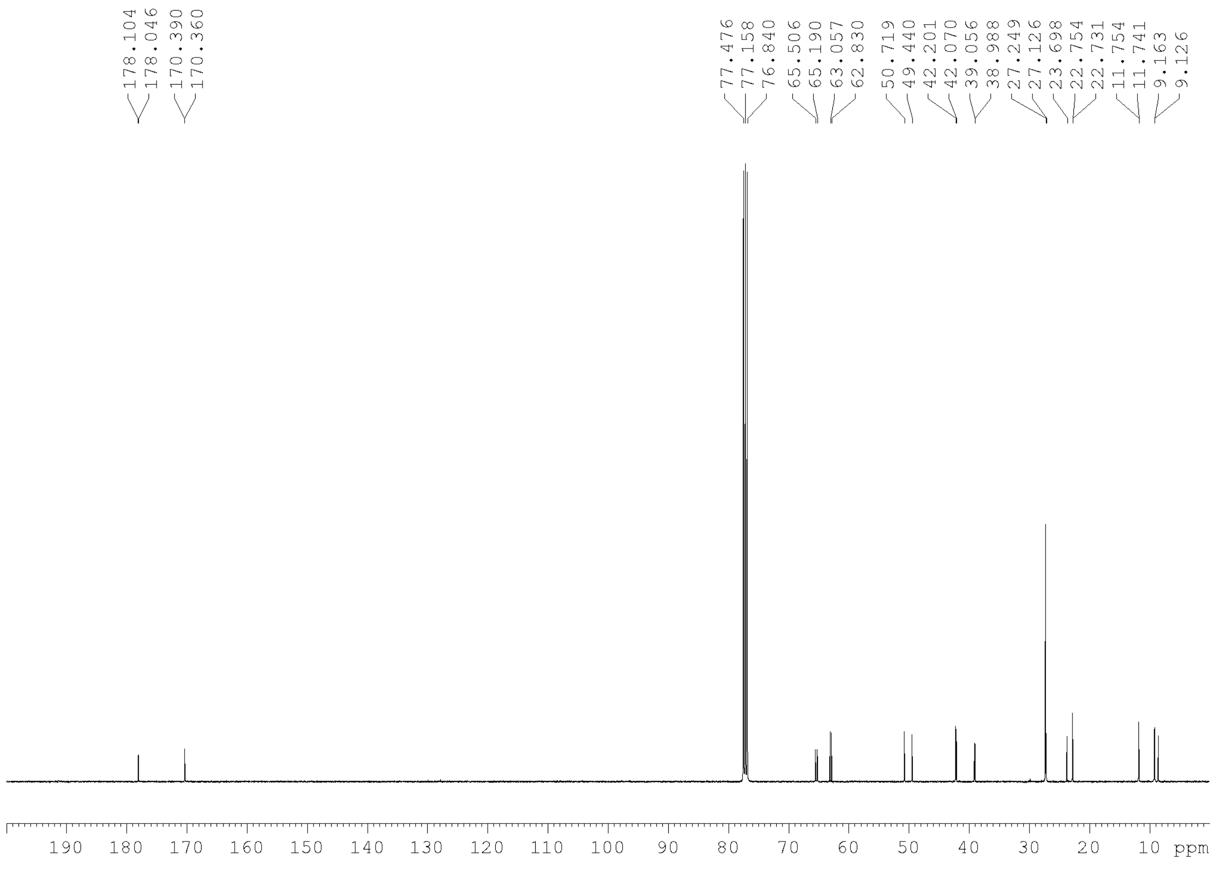
178.104
178.046
170.390
170.360
77.476
77.158
76.840
65.506
65.190
63.057
62.830
50.719
49.440
42.201
42.070
39.056
38.988
27.249
27.126
23.698
22.754
22.731
11.754
11.741
9.163
9.126
190 180 170 160 150 140 130 120 110 100 90 80 70 60 50 40 30 20 10 ppm

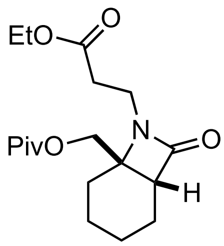
EtO
O
N
O
PivO
H

2h

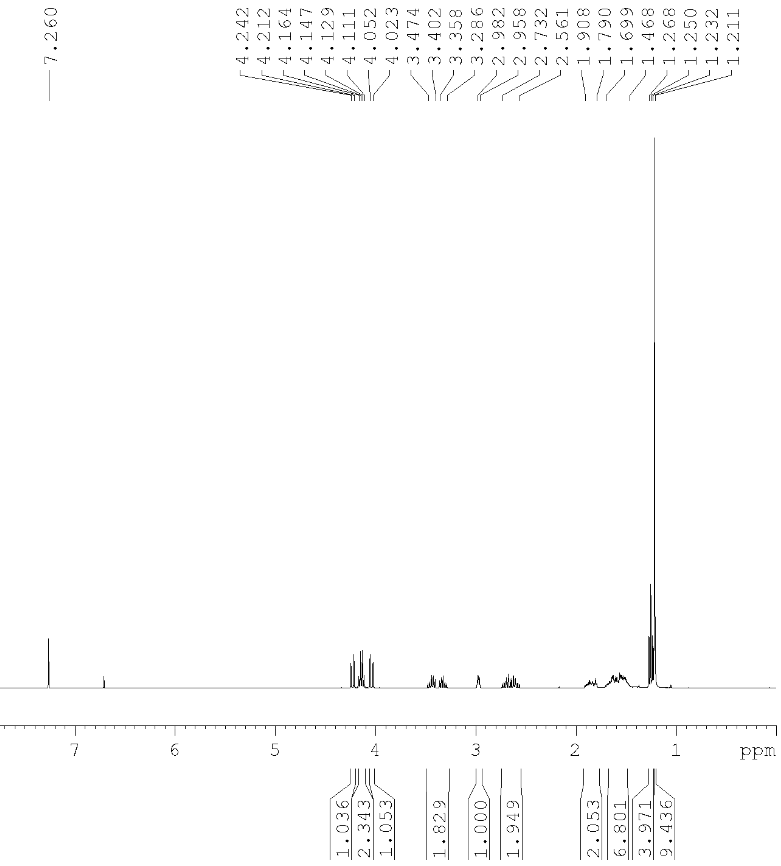
7.260
4.242
4.212
4.164
4.147
4.129
4.111
4.052
4.023
3.474
3.402
3.358
3.286
2.982
2.958
2.732
2.561
1.908
1.790
1.699
1.468
1.268
1.250
1.232
1.211
11 10 9 8 7 6 5 4 3 2 1 ppm
1.036
2.343
1.053
1.829
1.000
1.949
2.053
6.801
3.971
9.436

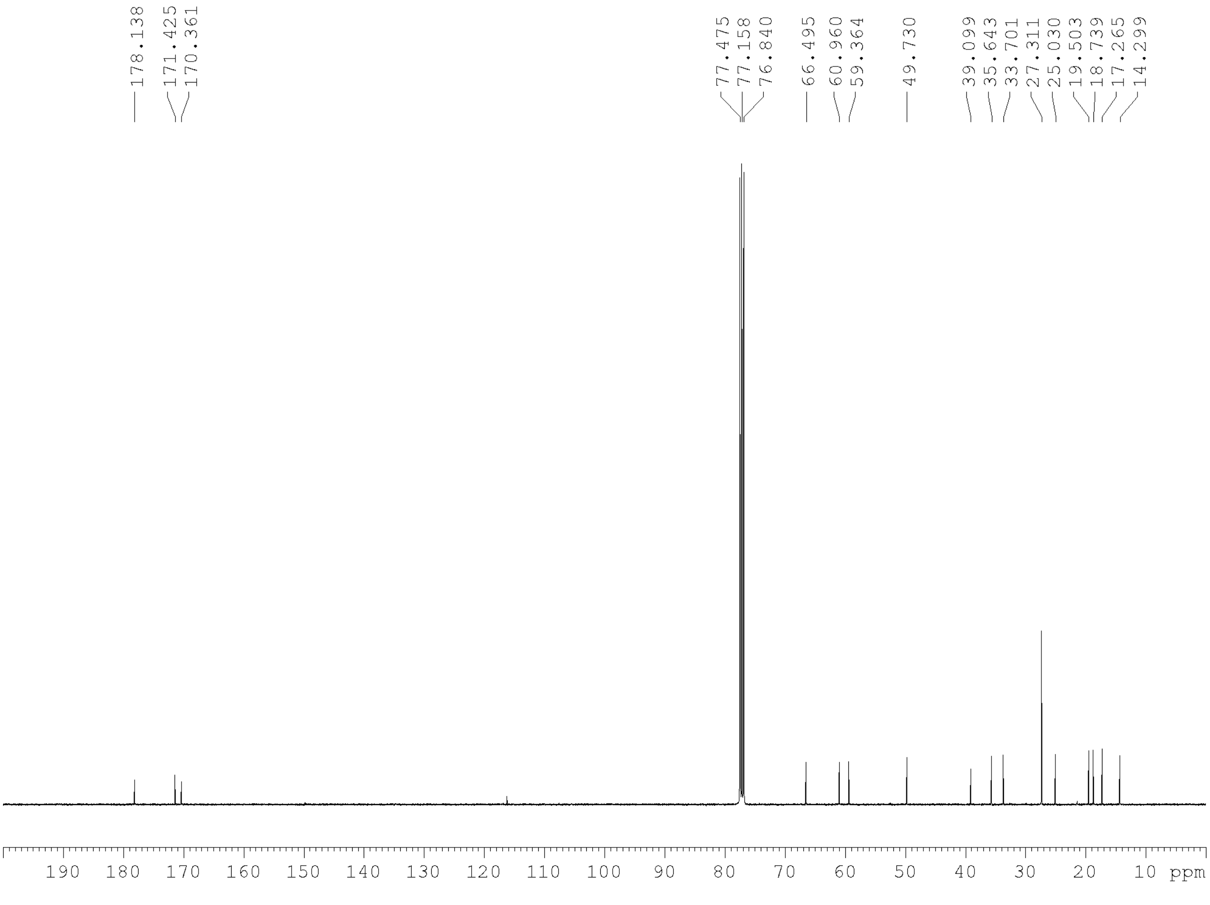
178.138
171.425
170.361
77.475
77.158
76.840
66.495
60.960
59.364
49.730
39.099
35.643
33.701
27.311
25.030
19.503
18.739
17.265
14.299
190 180 170 160 150 140 130 120 110 100 90 80 70 60 50 40 30 20 10 ppm

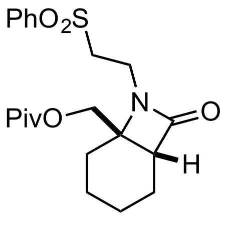
PhO2S
N
O
PivO
H

2i

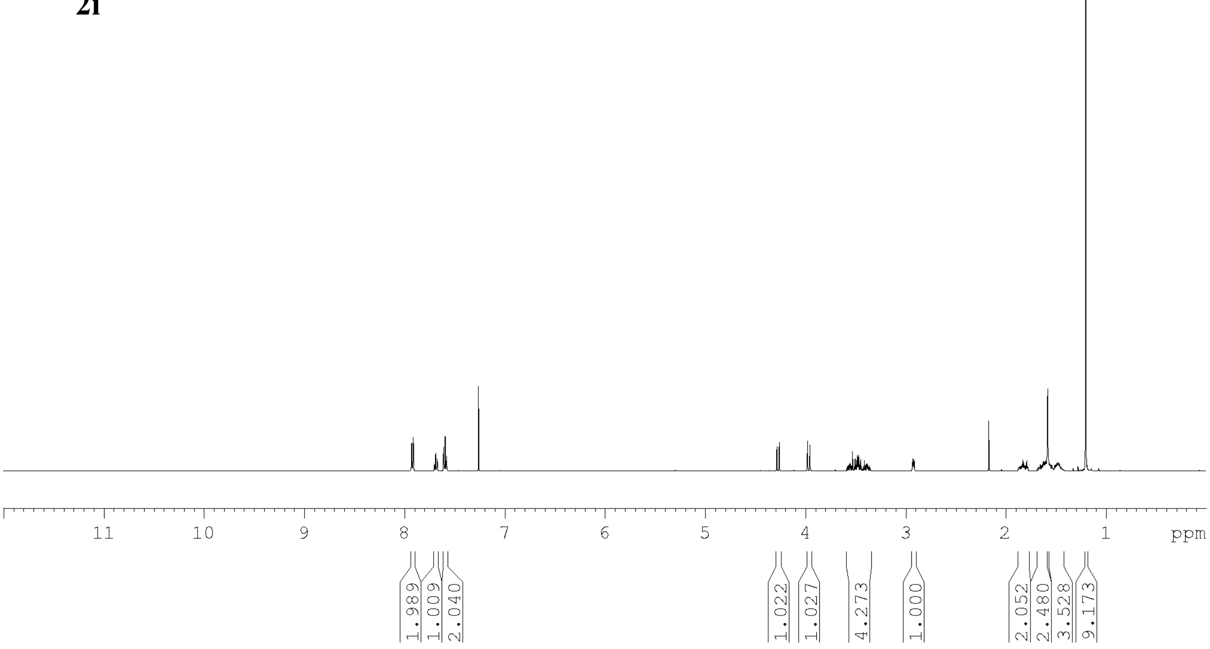
7.927
7.912
7.706
7.702
7.700
7.692
7.688
7.685
7.675
7.673
7.670
7.609
7.593
7.578
7.260
4.283
4.259
3.977
3.953
3.584
3.554
3.529
3.467
3.409
3.355
2.932
2.925
2.919
2.911
1.865
1.829
1.790
1.780
1.672
1.666
1.602
1.581
1.530
1.442
1.202
11
10
9
8
7
6
5
4
3
2
1
ppm
1.989
1.009
2.040
1.022
1.027
4.273
1.000
2.052
2.480
3.528
9.173

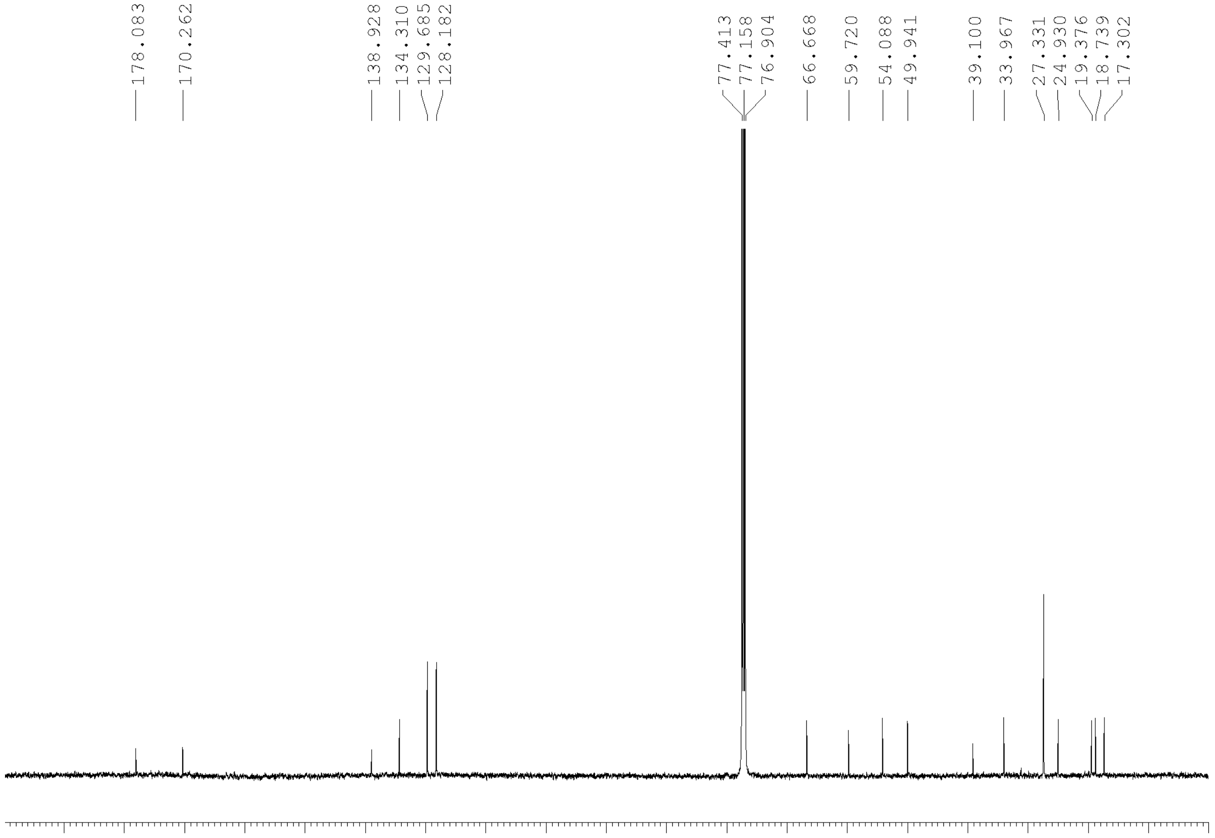
178.083
170.262
138.928
134.310
129.685
128.182
77.413
77.158
76.904
66.668
59.720
54.088
49.941
39.100
33.967
27.331
24.930
19.376
18.739
17.302
190
180
170
160
150
140
130
120
110
100
90
80
70
60
50
40
30
20
10 ppm

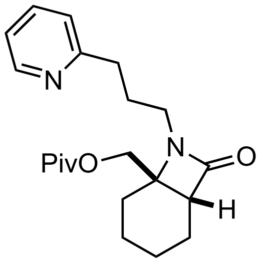

N
PivO
N
O
H

2j

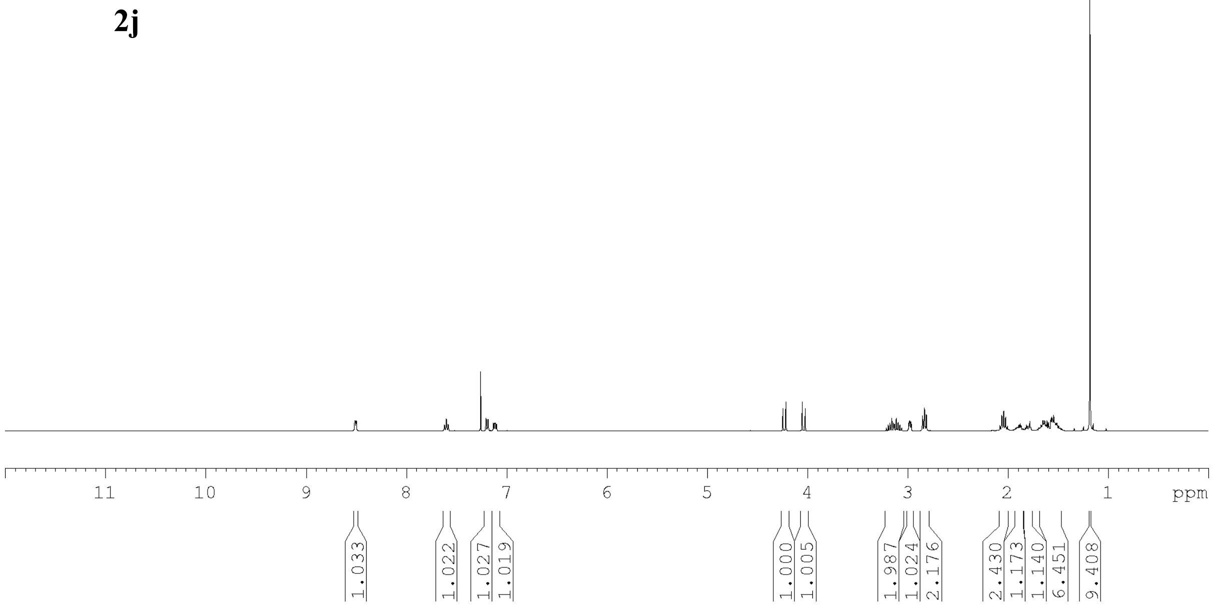

8.512
8.500
7.621
7.617
7.602
7.598
7.583
7.578
7.259
7.204
7.184
7.131
7.118
7.112
7.100
4.244
4.214
4.050
4.021
3.212
3.158
3.113
3.059
2.989
2.980
2.851
2.832
2.813
2.079
2.060
2.041
2.022
2.003
1.922
1.708
1.464
1.181
11 10 9 8 7 6 5 4 3 2 1 ppm
1.033
1.022
1.027
1.019
1.000
1.005
1.987
1.024
2.176
2.430
1.173
1.140
6.451
9.408

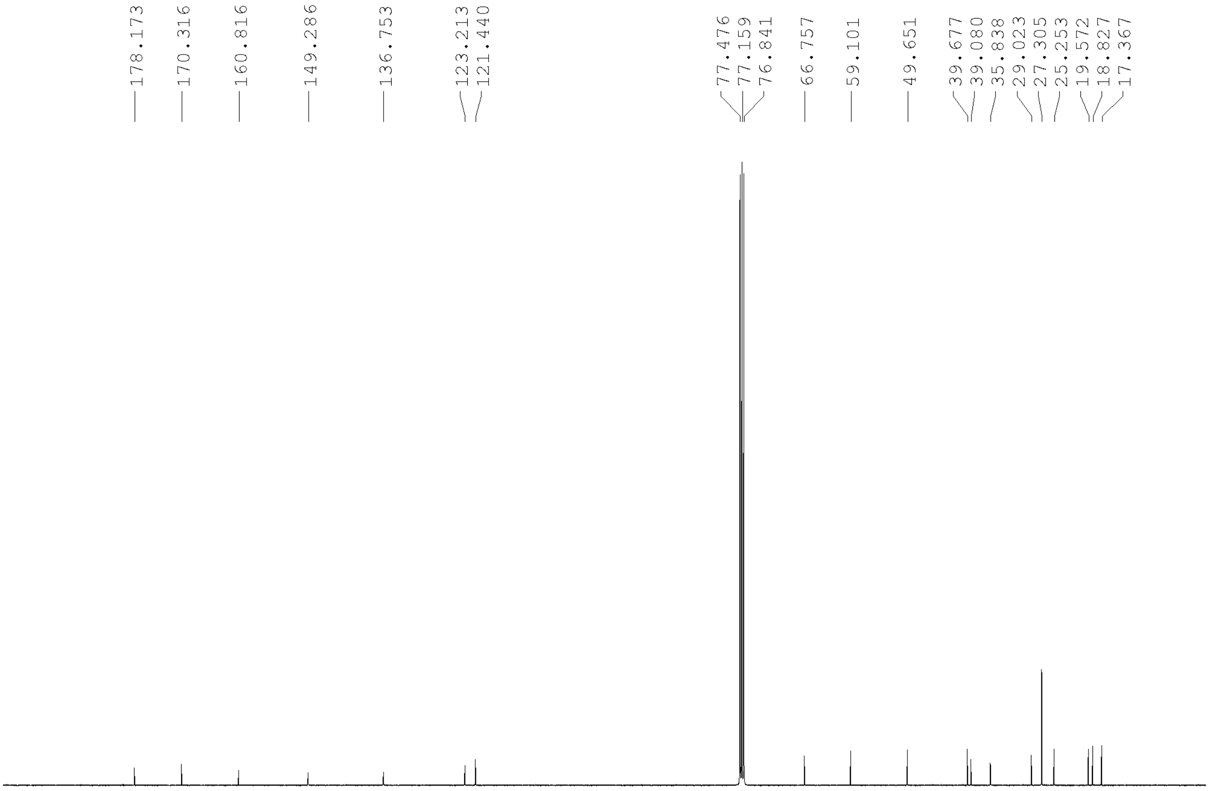

178.173
170.316
160.816
149.286
136.753
123.213
121.440
77.476
77.159
76.841
66.757
59.101
49.651
39.677
39.080
35.838
29.023
27.305
25.253
19.572
18.827
17.367
190 180 170 160 150 140 130 120 110 100 90 80 70 60 50 40 30 20 10 ppm

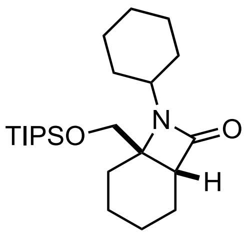
TIPSO
N
O
H

2k

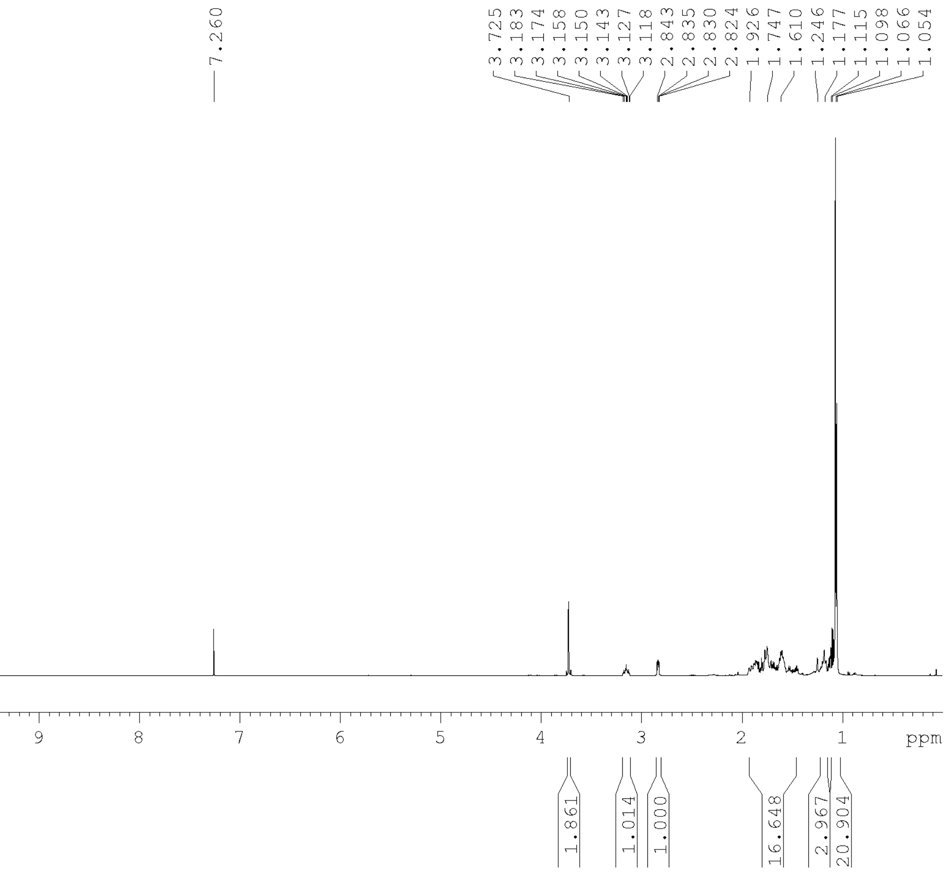
7.260
3.725
3.183
3.174
3.158
3.150
3.143
3.127
3.118
2.843
2.835
2.830
2.824
1.926
1.747
1.610
1.246
1.177
1.115
1.098
1.066
1.054
11 10 9 8 7 6 5 4 3 2 1 ppm
1.861
1.014
1.000
16.648
2.967
20.904

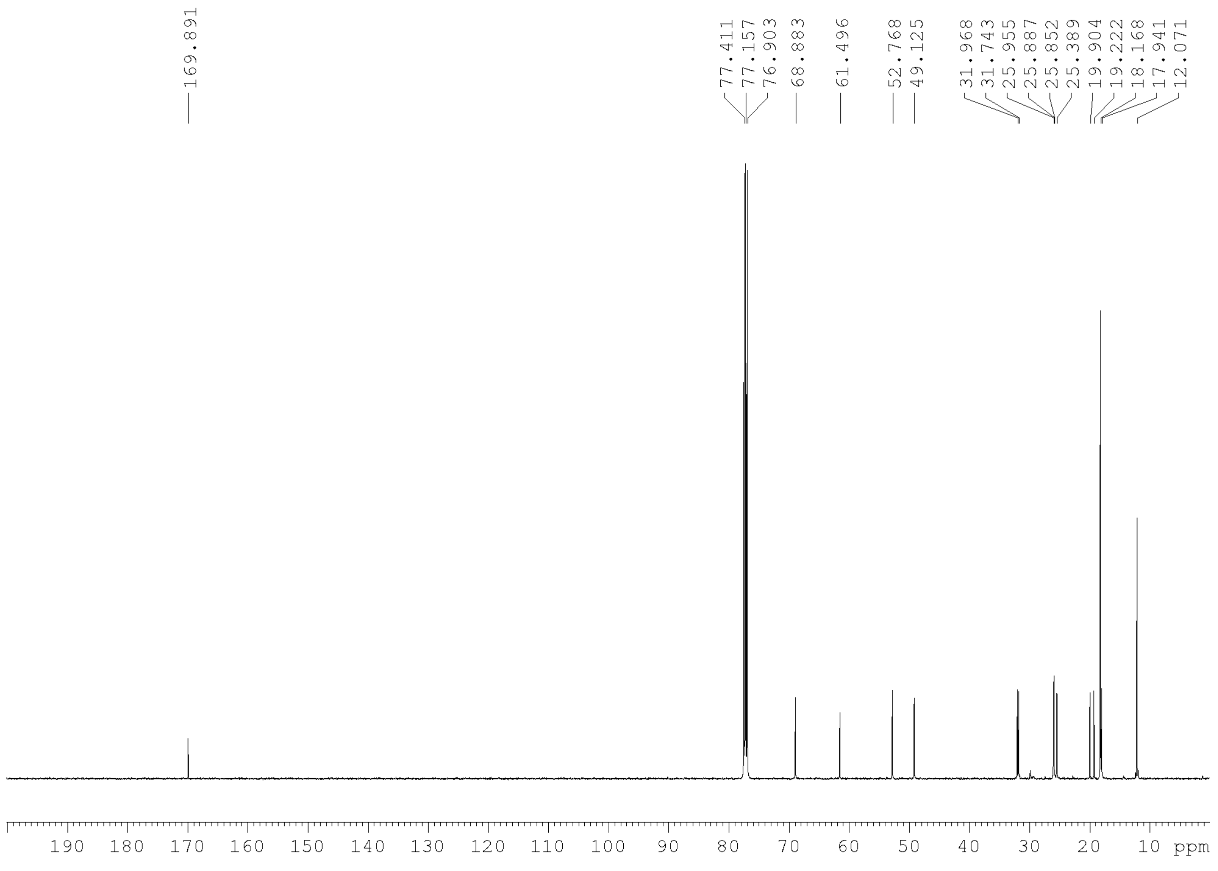
169.891
77.411
77.157
76.903
68.883
61.496
52.768
49.125
31.968
31.743
25.955
25.887
25.852
25.389
19.904
19.222
18.168
17.941
12.071
190 180 170 160 150 140 130 120 110 100 90 80 70 60 50 40 30 20 10 ppm

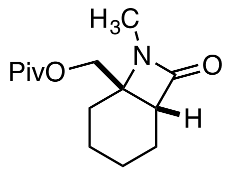
H3C
N
O
PivO
H

2m

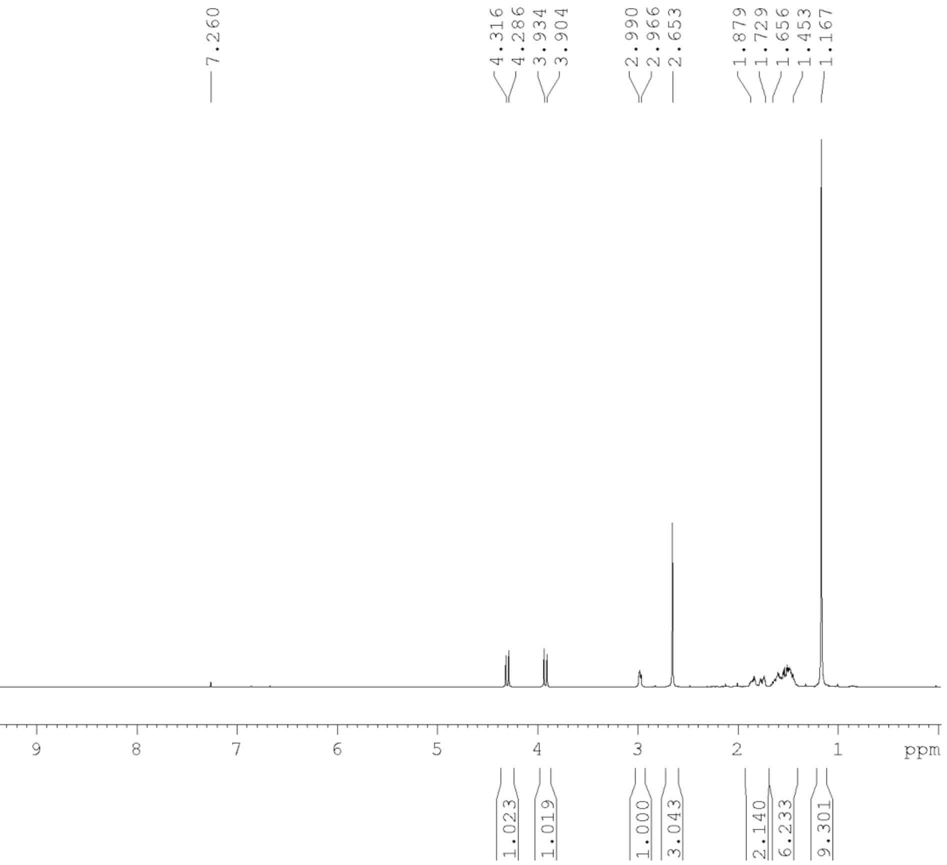
7.260
4.316
4.286
3.934
3.904
2.990
2.966
2.653
1.879
1.729
1.656
1.453
1.167
11 10 9 8 7 6 5 4 3 2 1 ppm
1.023
1.019
1.000
3.043
2.140
6.233
9.301

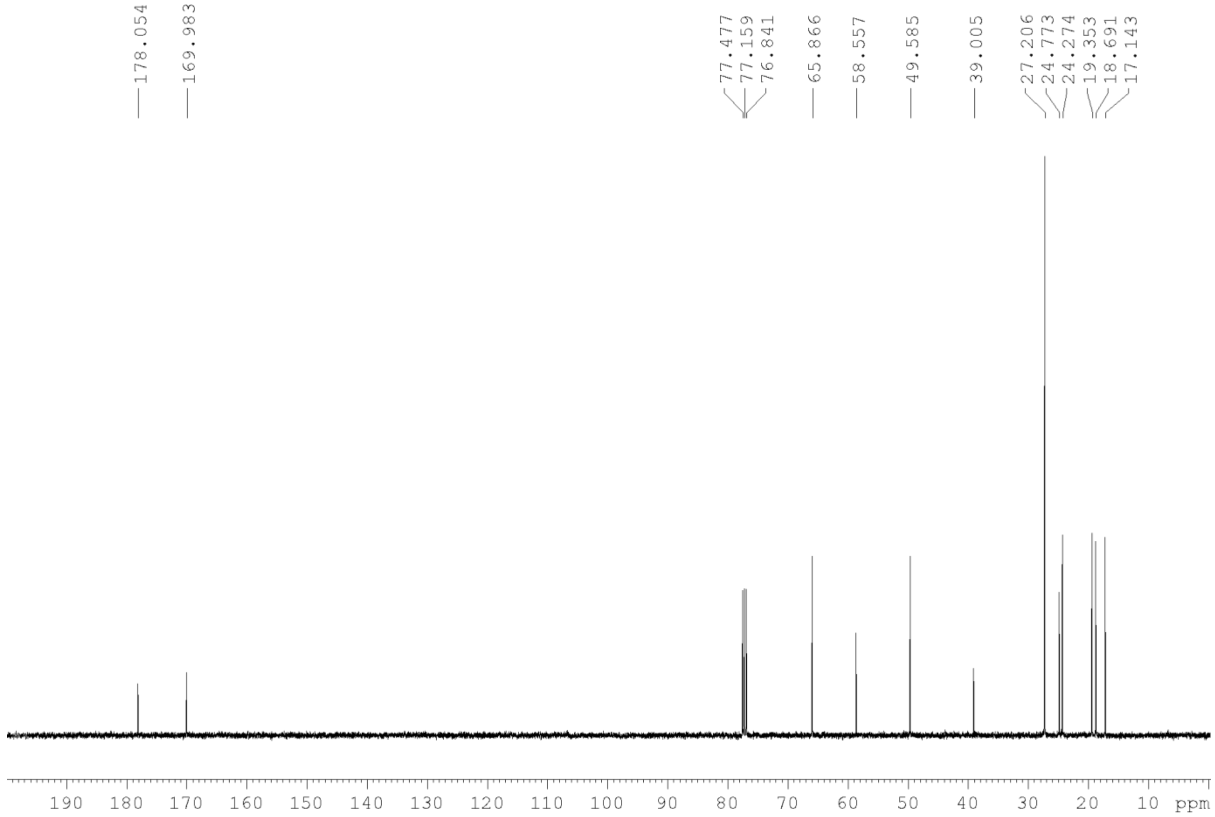
178.054
169.983
77.477
77.159
76.841
65.866
58.557
49.585
39.005
27.206
24.773
24.274
19.353
18.691
17.143
190 180 170 160 150 140 130 120 110 100 90 80 70 60 50 40 30 20 10 ppm

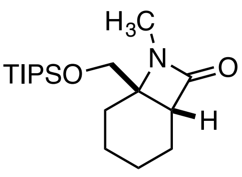
H3C
N
O
TIPSO
H

2n

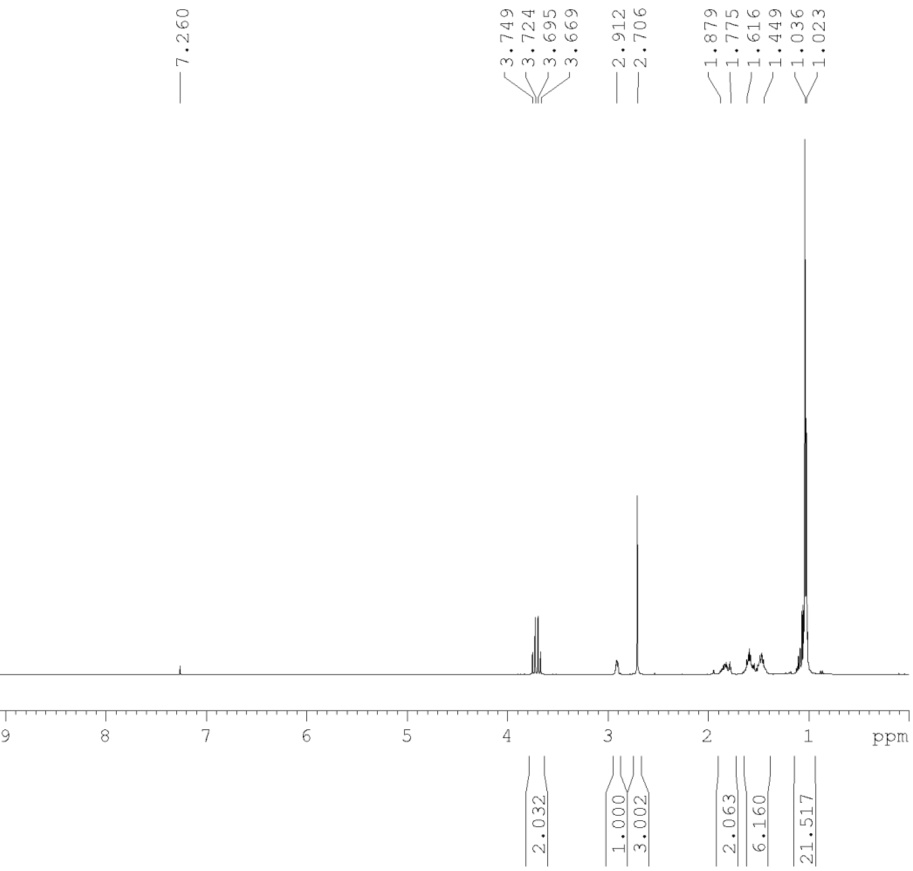
7.260
3.749
3.724
3.695
3.669
2.912
2.706
1.879
1.775
1.616
1.449
1.036
1.023
11 10 9 8 7 6 5 4 3 2 1 ppm
2.032
1.000
3.002
2.063
6.160
21.517

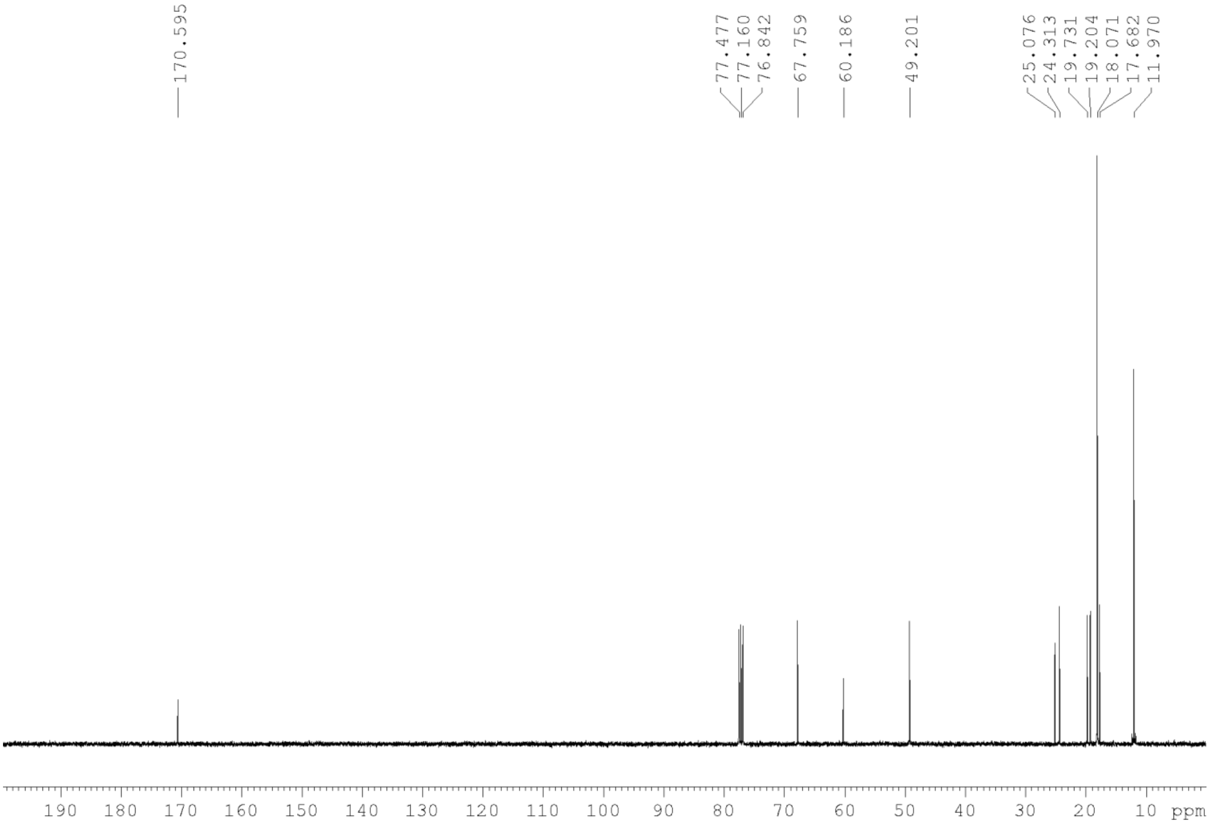
170.595
77.477
77.160
76.842
67.759
60.186
49.201
25.076
24.313
19.731
19.204
18.071
17.682
11.970
190 180 170 160 150 140 130 120 110 100 90 80 70 60 50 40 30 20 10 ppm

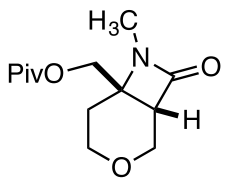
H3C
N
O
PivO
H
O

2o

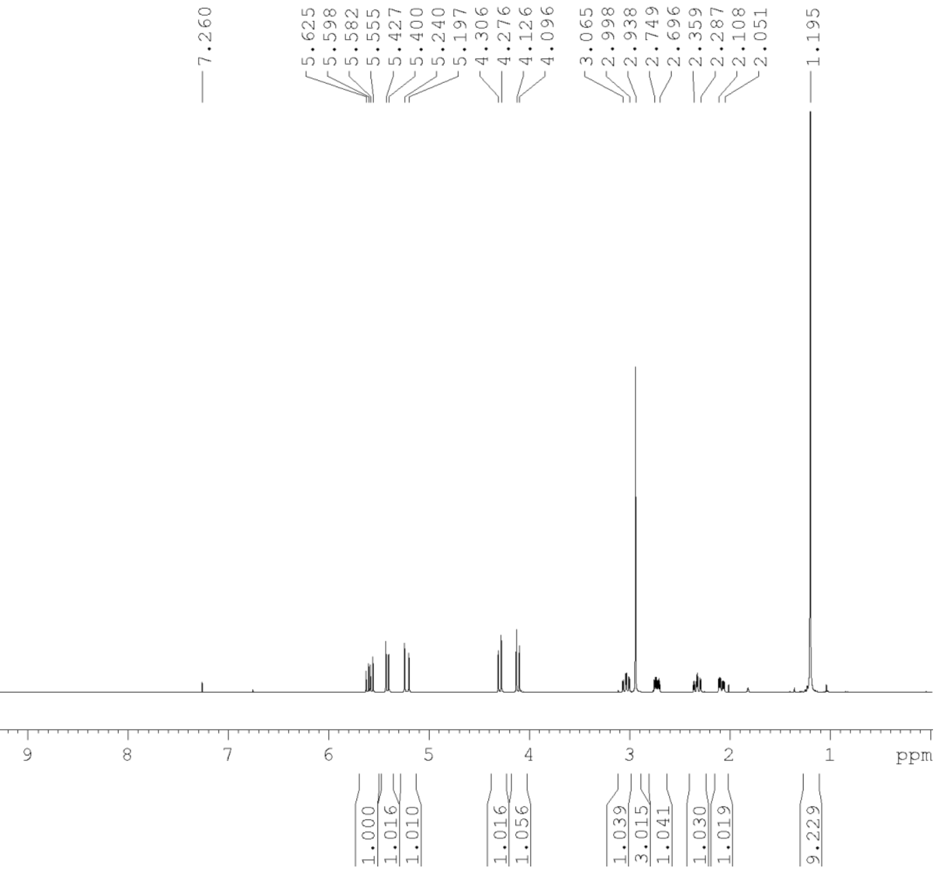
7.260
5.625
5.598
5.582
5.555
5.427
5.400
5.240
5.197
4.306
4.276
4.126
4.096
3.065
2.998
2.938
2.749
2.696
2.359
2.287
2.108
2.051
1.195
11 10 9 8 7 6 5 4 3 2 1 ppm
1.000
1.016
1.010
1.016
1.056
1.039
3.015
1.041
1.030
1.019
9.229

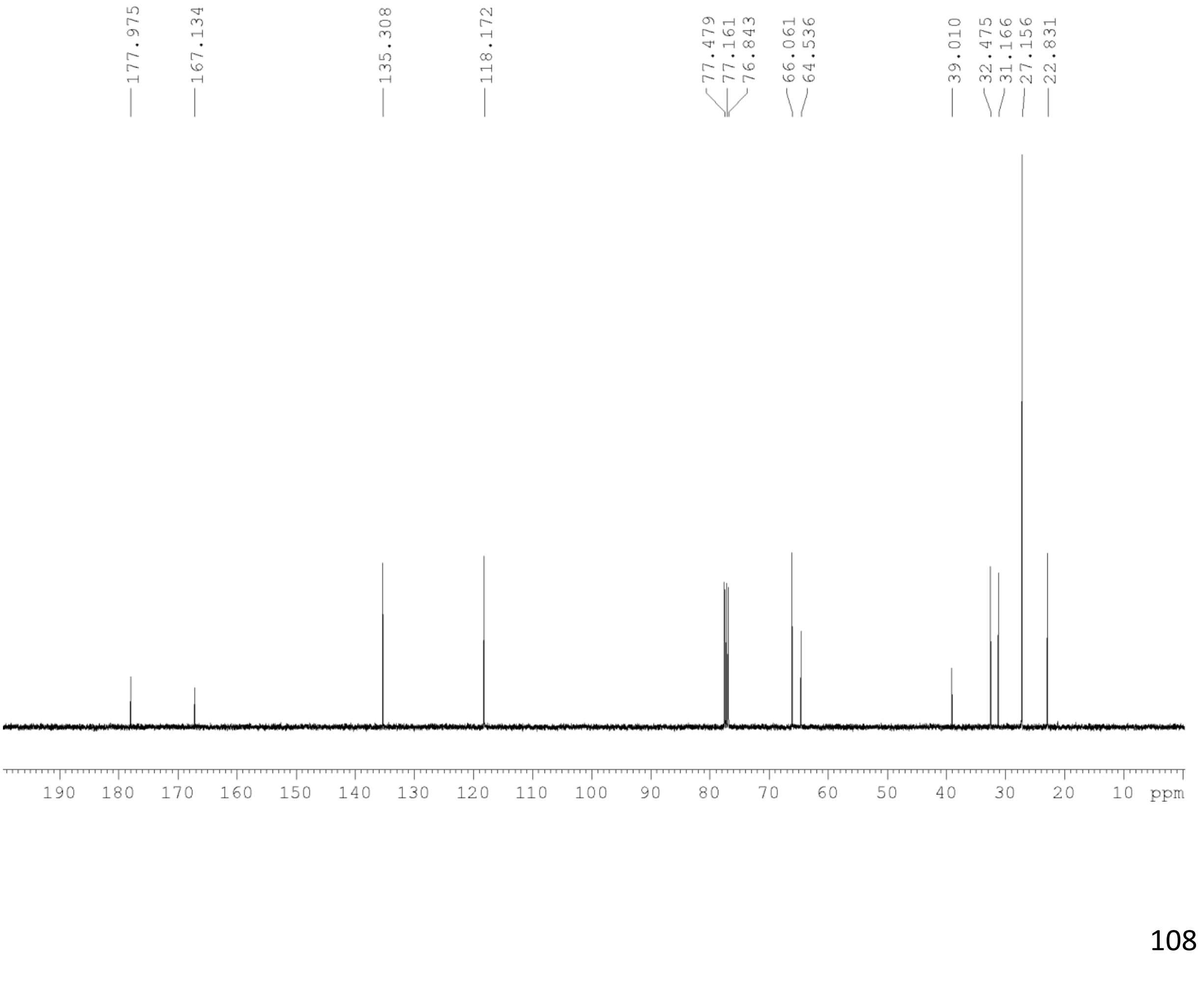
177.975
167.134
135.308
118.172
77.479
77.161
76.843
66.061
64.536
39.010
32.475
31.166
27.156
22.831
190 180 170 160 150 140 130 120 110 100 90 80 70 60 50 40 30 20 10 ppm

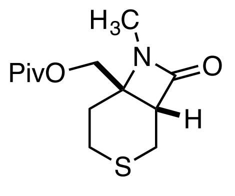

H_3C
N
O
PivO
H
S

2p

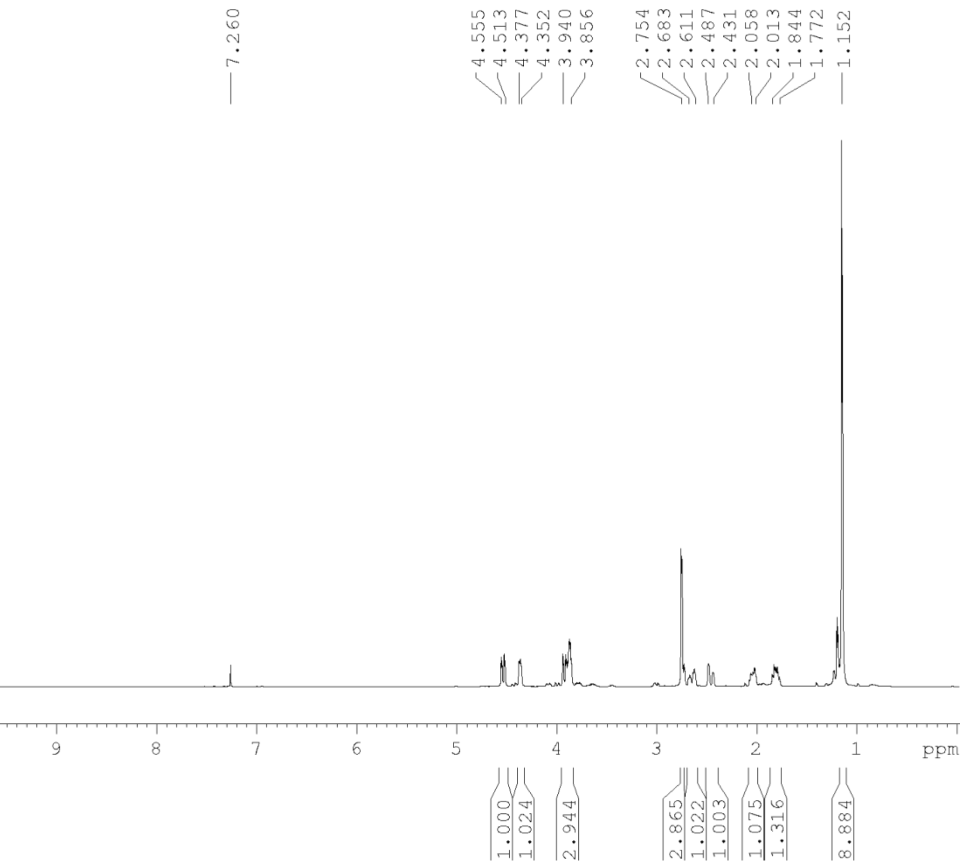

7.260
4.555
4.513
4.377
4.352
3.940
3.856
2.754
2.683
2.611
2.487
2.431
2.058
2.013
1.844
1.772
1.152
11 10 9 8 7 6 5 4 3 2 1 ppm
1.000
1.024
2.944
2.865
1.022
1.003
1.075
1.316
8.884

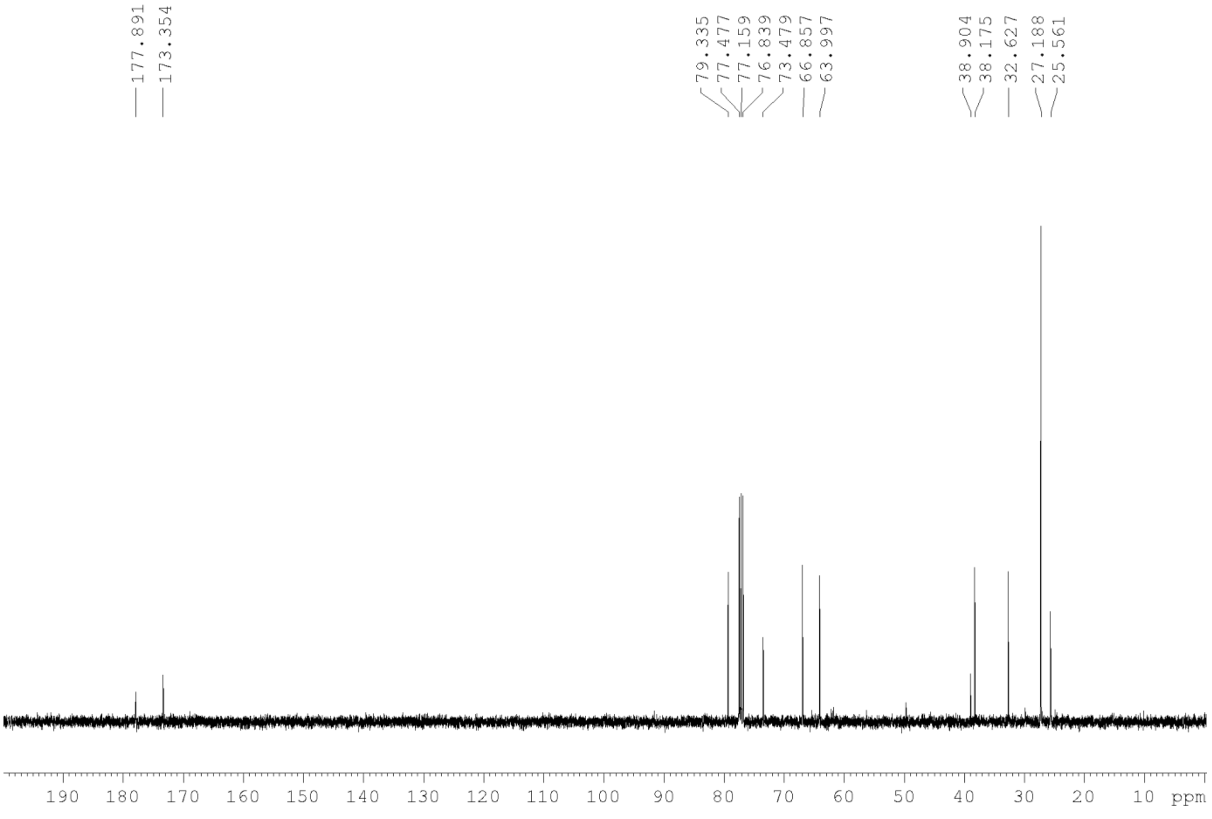

177.891
173.354
79.335
77.477
77.159
76.839
73.479
66.857
63.997
38.904
38.175
32.627
27.188
25.561
190 180 170 160 150 140 130 120 110 100 90 80 70 60 50 40 30 20 10 ppm

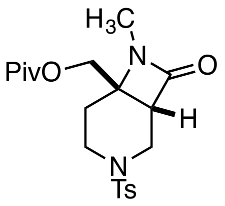

2q

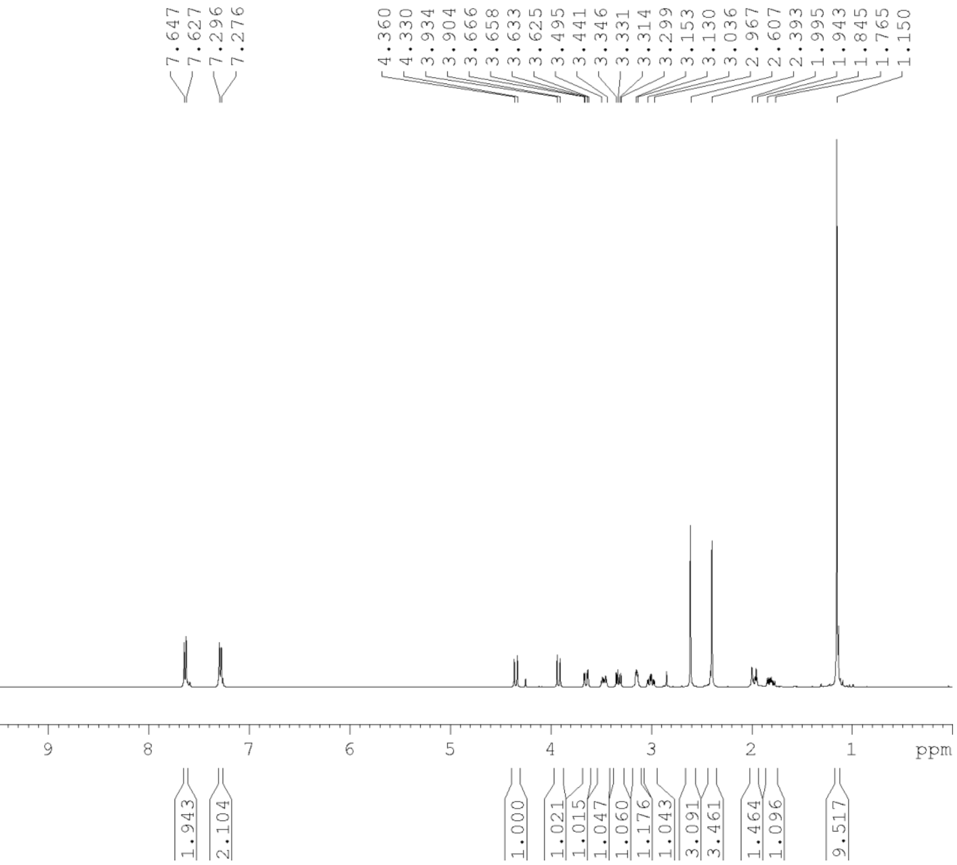

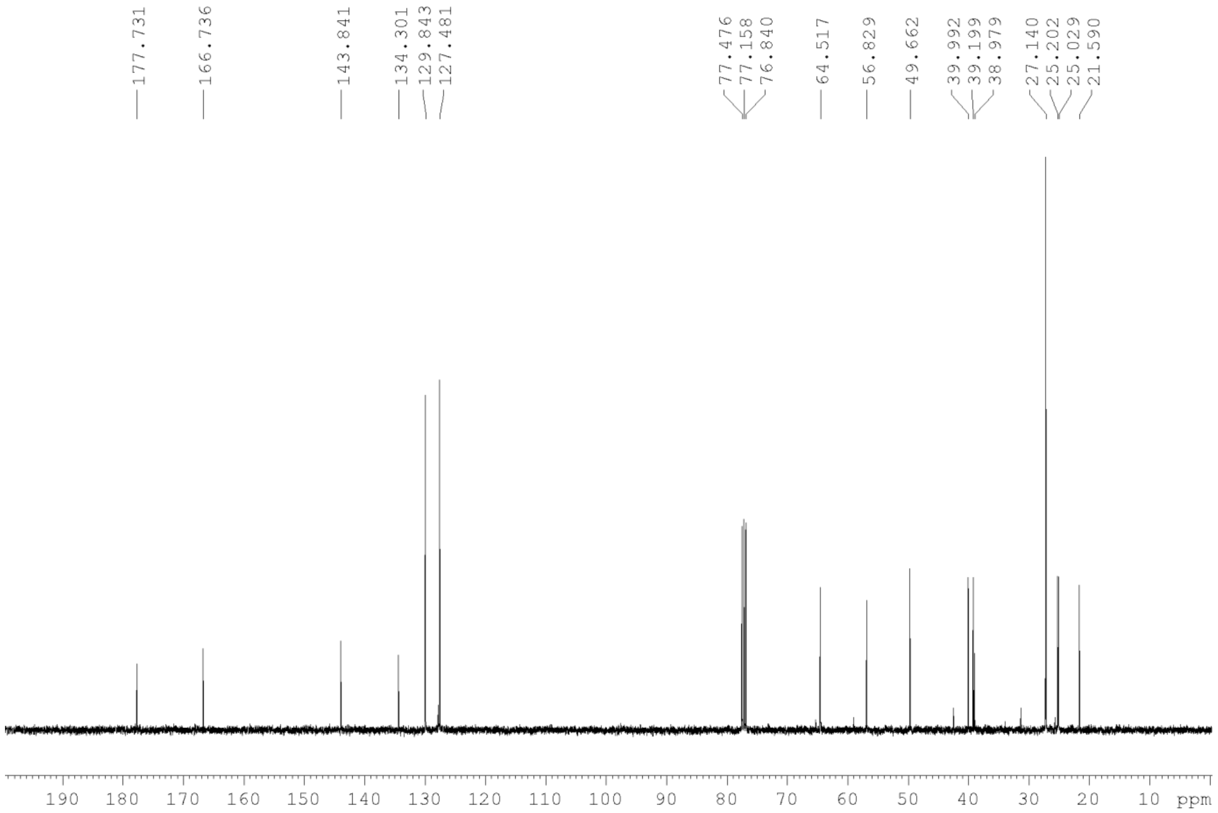

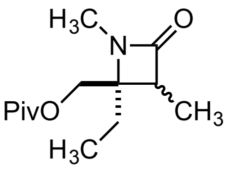
H3C
N
O
PivO
CH3
H3C

2r

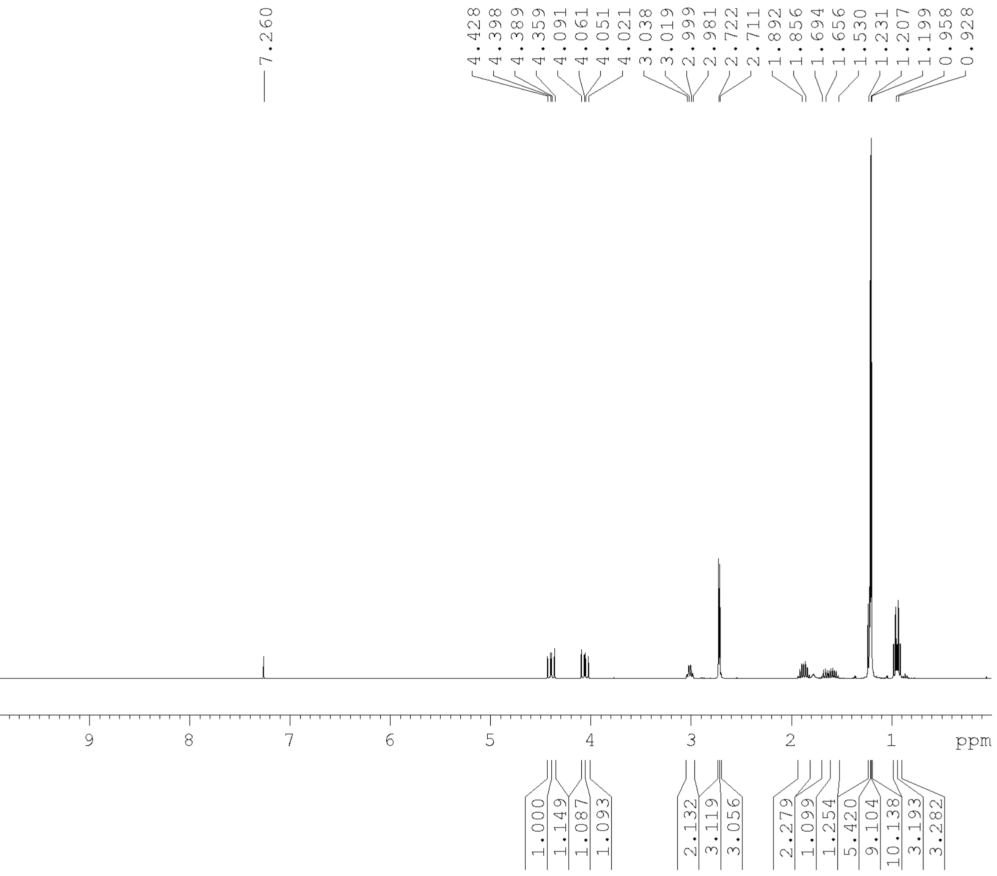
7.260
4.428
4.398
4.389
4.359
4.091
4.061
4.051
4.021
3.038
3.019
2.999
2.981
2.722
2.711
1.892
1.856
1.694
1.656
1.530
1.231
1.207
1.199
0.958
0.928
11 10 9 8 7 6 5 4 3 2 1 ppm
1.000
1.149
1.087
1.093
2.132
3.119
3.056
2.279
1.099
1.254
5.420
9.104
10.138
3.193
3.282

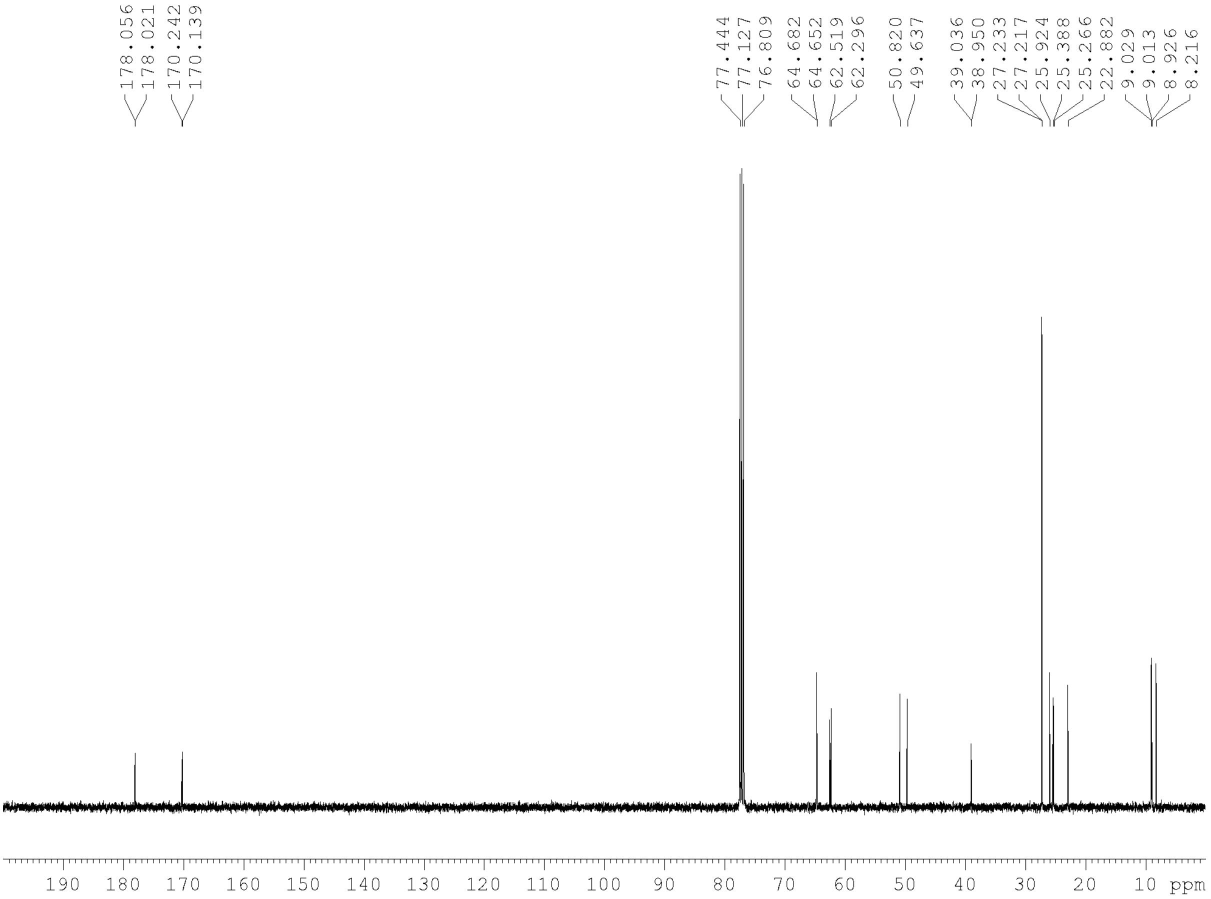
178.056
178.021
170.242
170.139
77.444
77.127
76.809
64.682
64.652
62.519
62.296
50.820
49.637
39.036
38.950
27.233
27.217
25.924
25.388
25.266
22.882
9.029
9.013
8.926
8.216
190 180 170 160 150 140 130 120 110 100 90 80 70 60 50 40 30 20 10 ppm

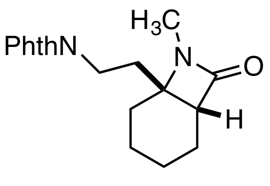
H3C
PhthN
N
O
H

2s

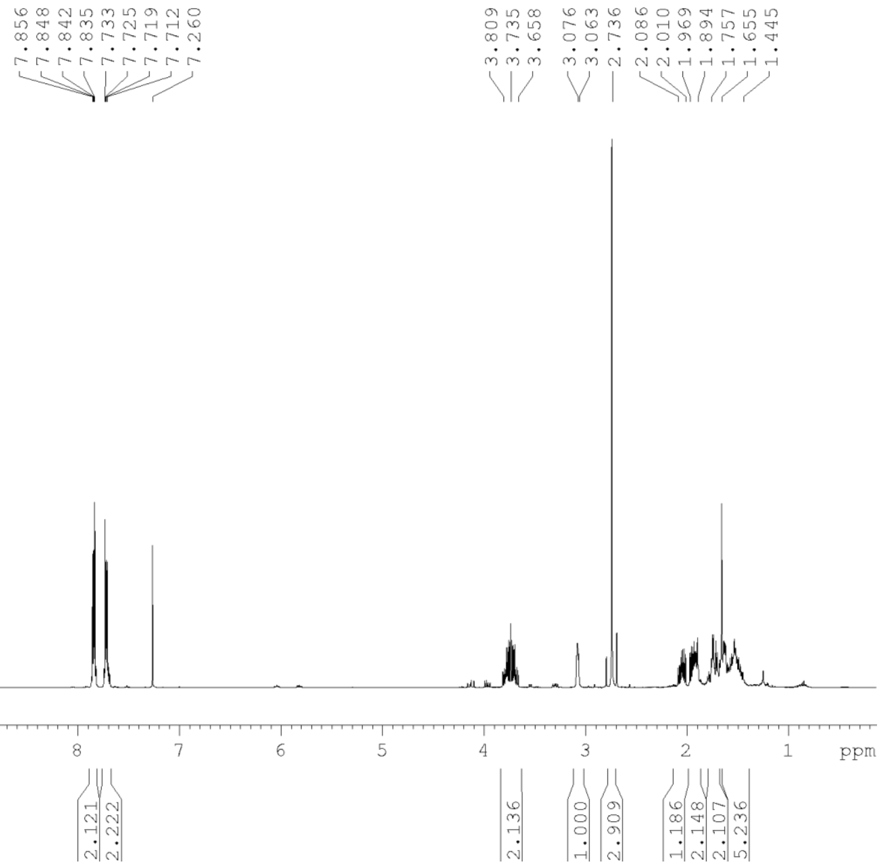
7.856
7.848
7.842
7.835
7.733
7.725
7.719
7.712
7.260
3.809
3.735
3.658
3.076
3.063
2.736
2.086
2.010
1.969
1.894
1.757
1.655
1.445
11 10 9 8 7 6 5 4 3 2 1 ppm
2.121
2.222
2.136
1.000
2.909
1.186
2.148
2.107
5.236

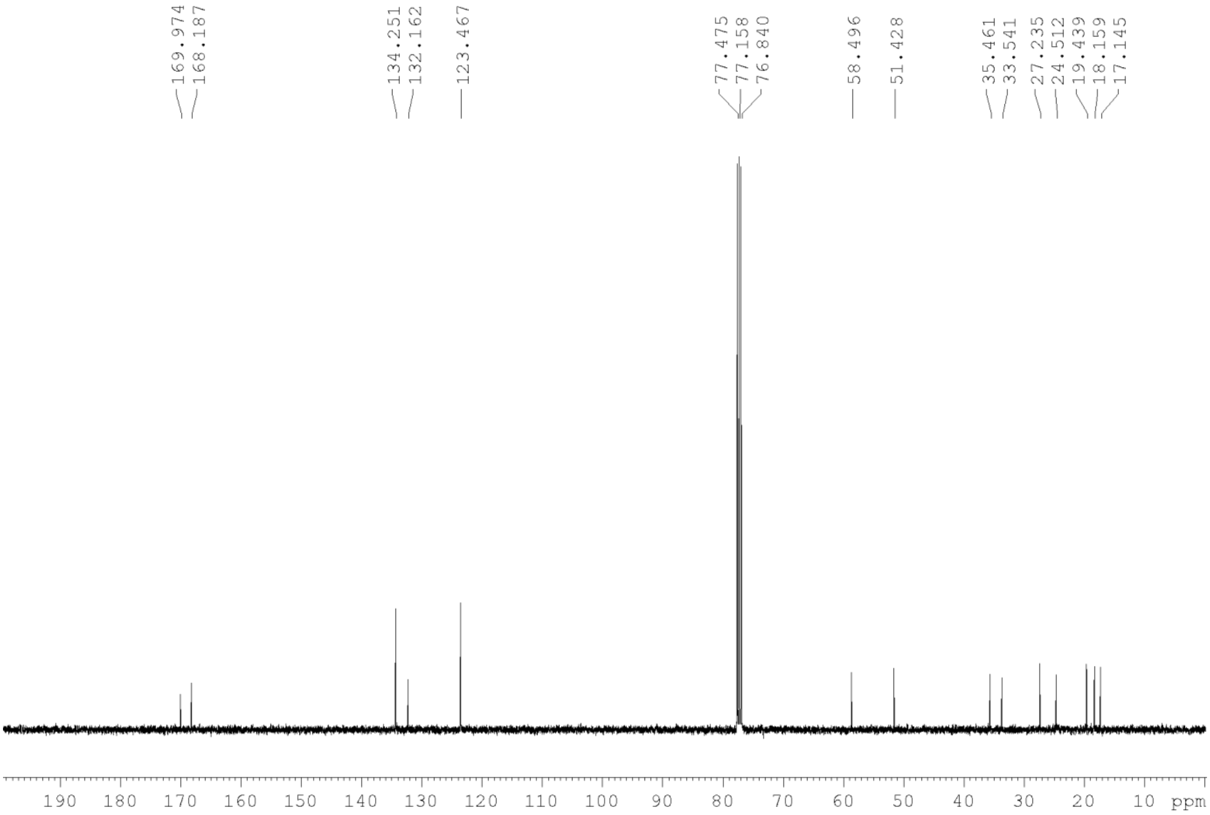
169.974
168.187
134.251
132.162
123.467
77.475
77.158
76.840
58.496
51.428
35.461
33.541
27.235
24.512
19.439
18.159
17.145
190 180 170 160 150 140 130 120 110 100 90 80 70 60 50 40 30 20 10 ppm

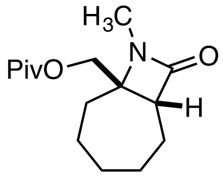
H3C
N
O
PivO
H

2t

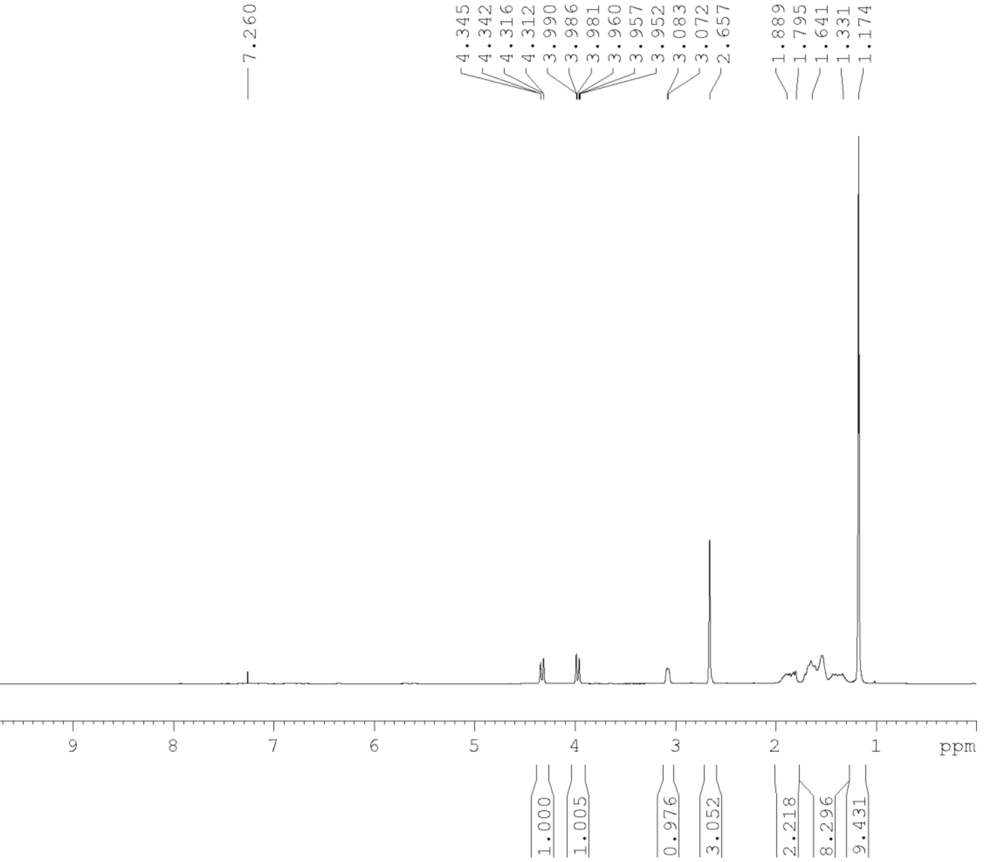
7.260
4.345
4.342
4.316
4.312
3.990
3.986
3.981
3.960
3.957
3.952
3.083
3.072
2.657
1.889
1.795
1.641
1.331
1.174
11 10 9 8 7 6 5 4 3 2 1 ppm
1.000
1.005
0.976
3.052
2.218
8.296
9.431

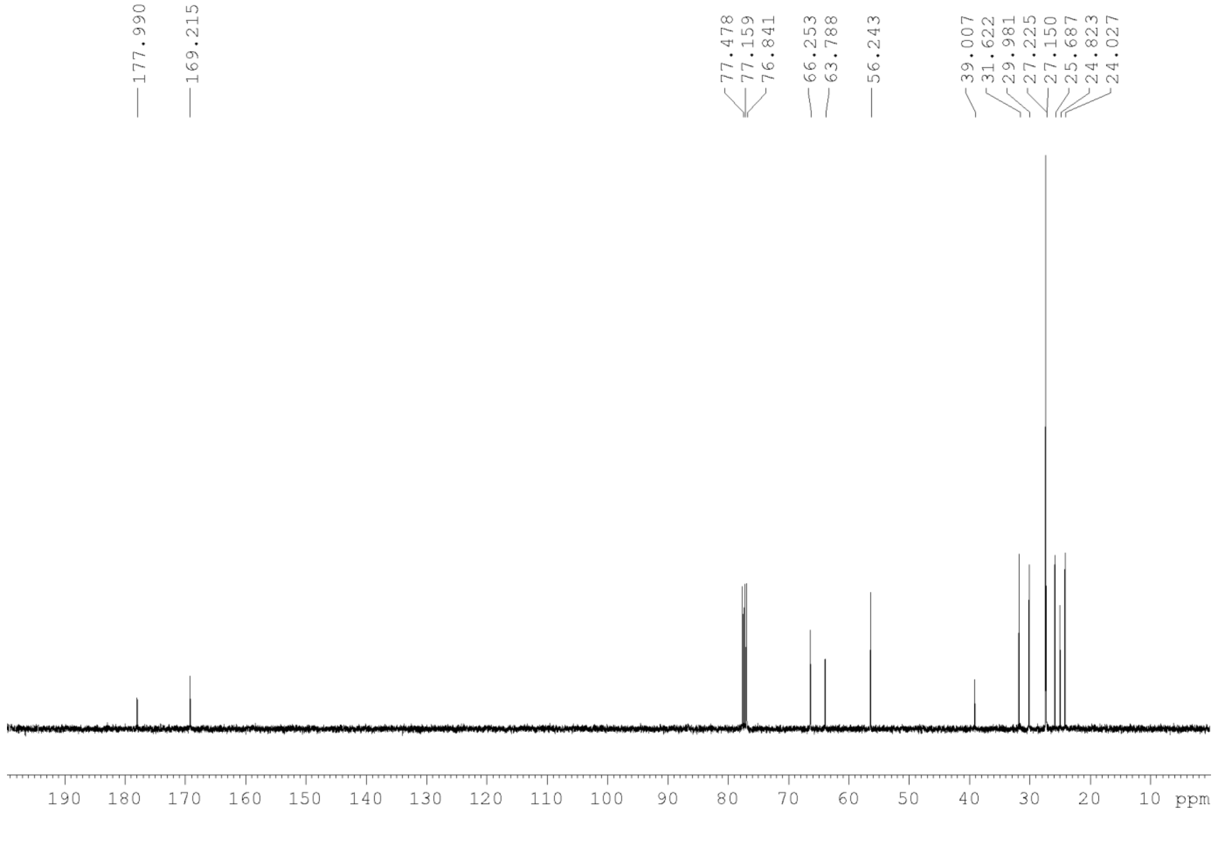
177.990
169.215
77.478
77.159
76.841
66.253
63.788
56.243
39.007
31.622
29.981
27.225
27.150
25.687
24.823
24.027
190 180 170 160 150 140 130 120 110 100 90 80 70 60 50 40 30 20 10 ppm

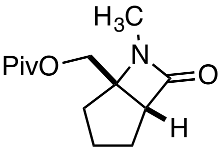
H3C
N
PivO
O
H

2u

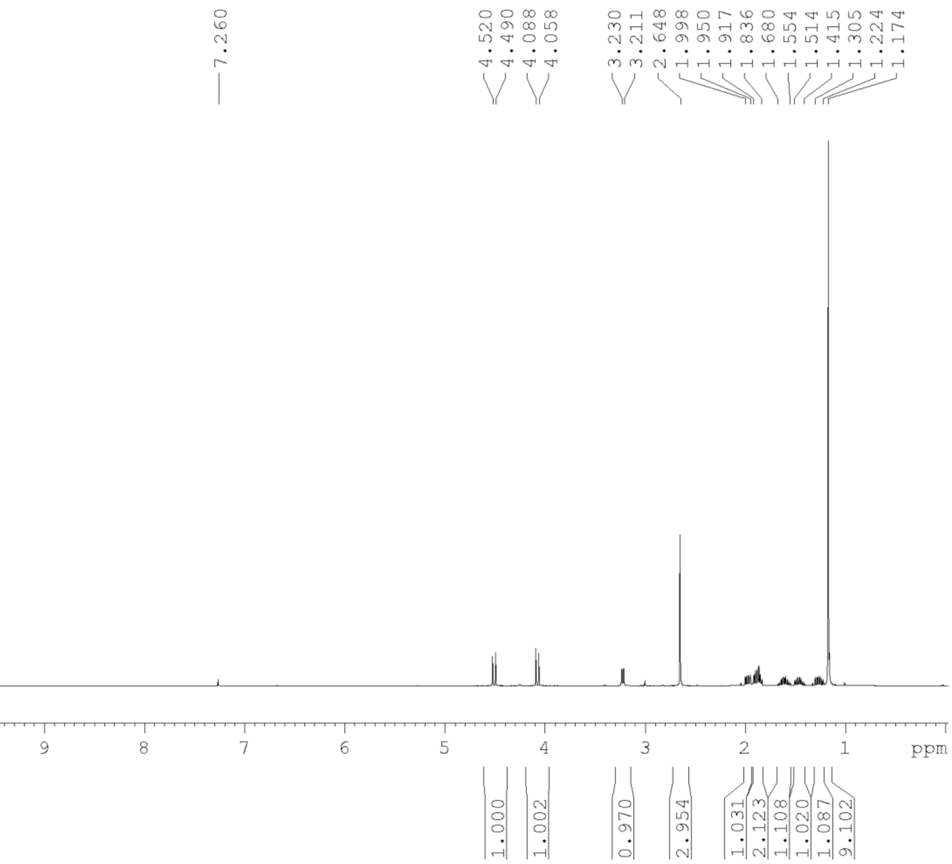
7.260
4.520
4.490
4.088
4.058
3.230
3.211
2.648
1.998
1.950
1.917
1.836
1.680
1.554
1.514
1.415
1.305
1.224
1.174
11 10 9 8 7 6 5 4 3 2 1 ppm
1.000
1.002
0.970
2.954
1.031
2.123
1.108
1.020
1.087
9.102

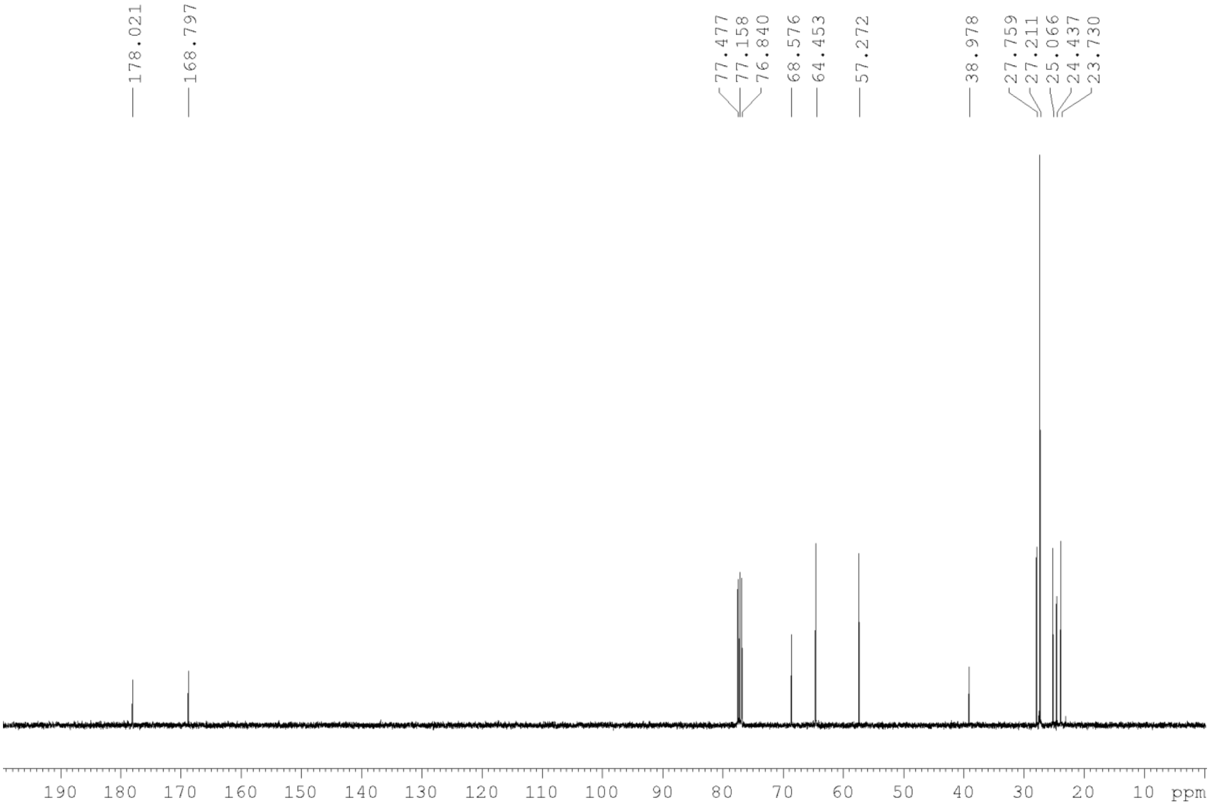
178.021
168.797
77.477
77.158
76.840
68.576
64.453
57.272
38.978
27.759
27.211
25.066
24.437
23.730
190 180 170 160 150 140 130 120 110 100 90 80 70 60 50 40 30 20 10 ppm

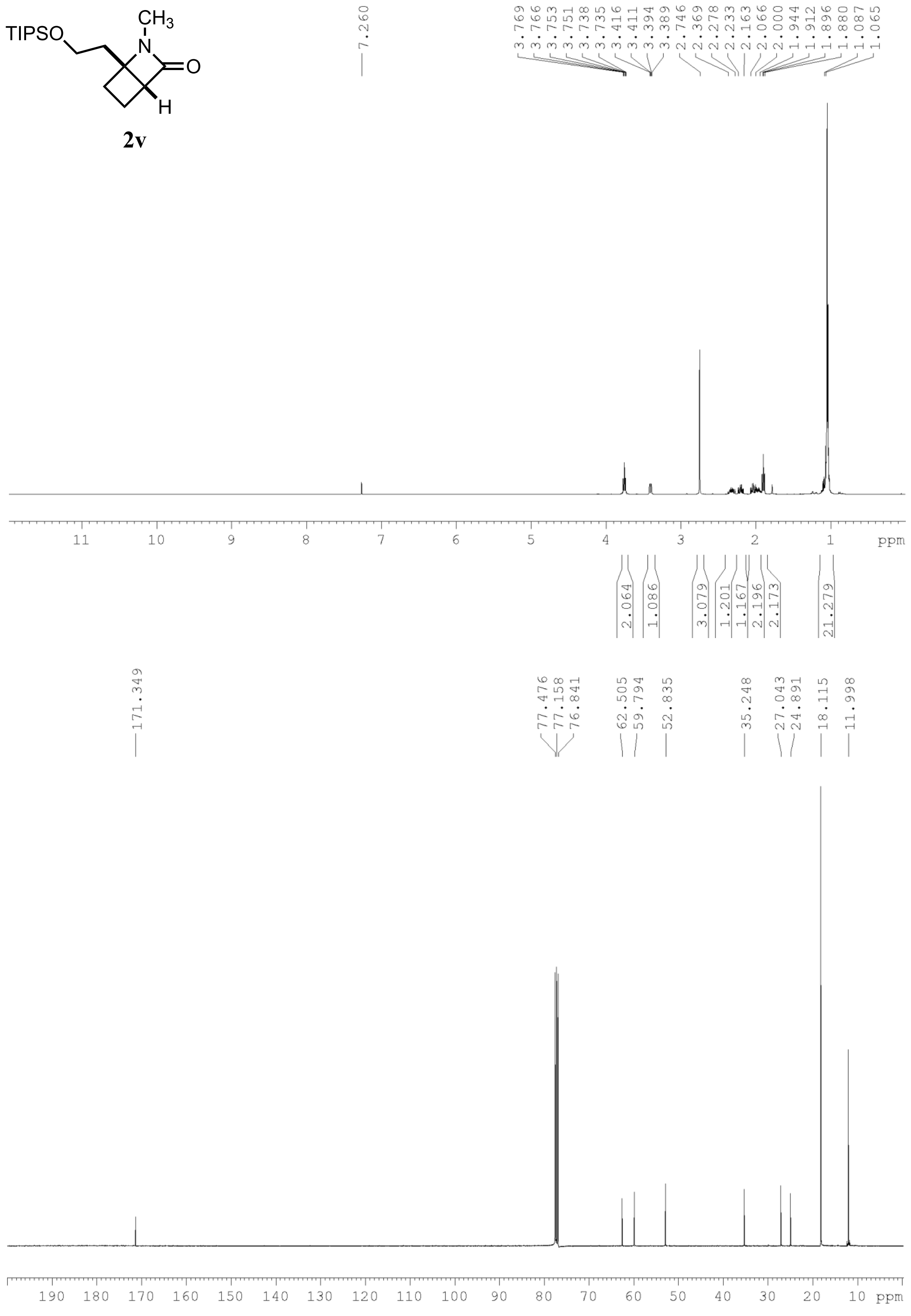
TIPSO
CH3
N
O
H
2v
7.260
3.769 3.766 3.753 3.751 3.738 3.735 3.416 3.411 3.394 3.389 2.746 2.369 2.278 2.233 2.163 2.066 2.000 1.944 1.912 1.896 1.880 1.087 1.065
11 10 9 8 7 6 5 4 3 2 1 ppm
2.064 1.086 3.079 1.201 1.167 2.196 2.173 21.279
171.349
77.476 77.158 76.841
62.505 59.794 52.835
35.248 27.043 24.891 18.115 11.998
190 180 170 160 150 140 130 120 110 100 90 80 70 60 50 40 30 20 10 ppm

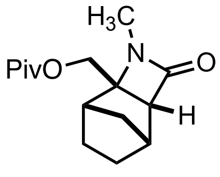
H3C
N
O
PivO
H

2w

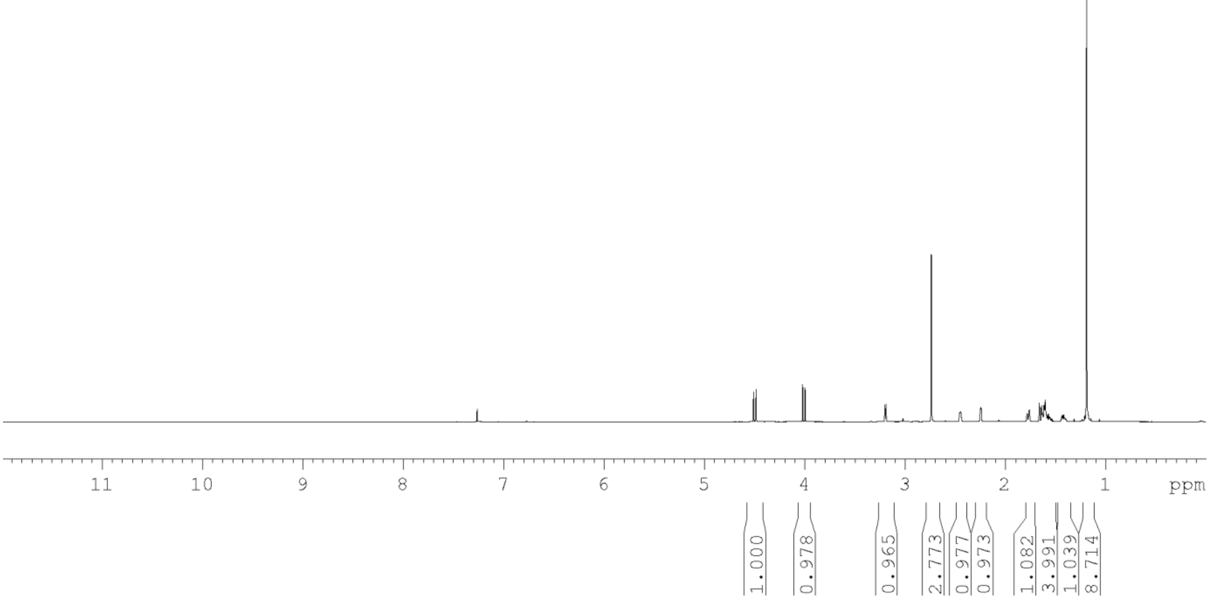
7.262
4.508
4.483
4.018
3.994
3.199
3.188
2.737
2.448
2.248
2.242
1.781
1.760
1.659
1.638
1.615
1.533
1.443
1.389
1.190
11 10 9 8 7 6 5 4 3 2 1 ppm
1.000
0.978
0.965
2.773
0.977
0.973
1.082
3.991
1.039
8.714

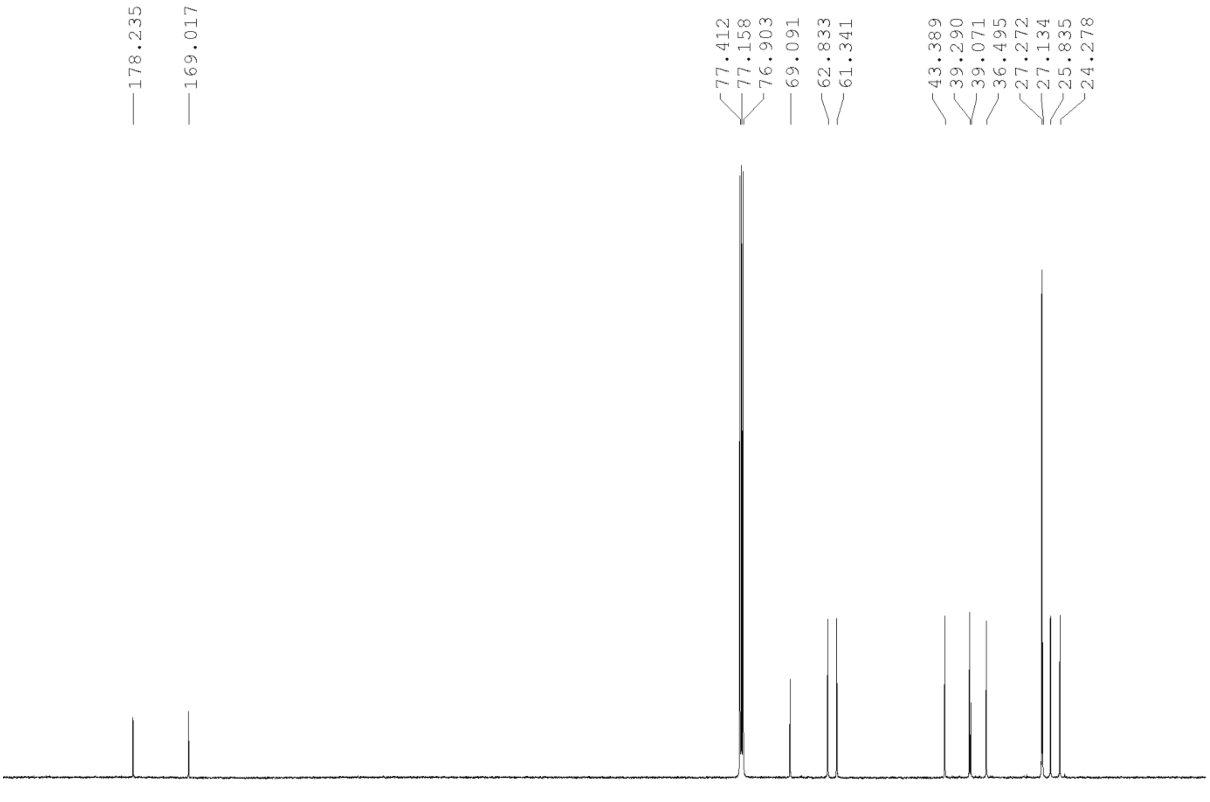
178.235
169.017
77.412
77.158
76.903
69.091
62.833
61.341
43.389
39.290
39.071
36.495
27.272
27.134
25.835
24.278
190 180 170 160 150 140 130 120 110 100 90 80 70 60 50 40 30 20 10 ppm

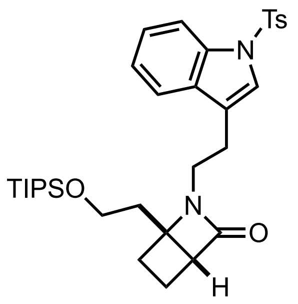
TIPSO
N
Ts
O
H

2x

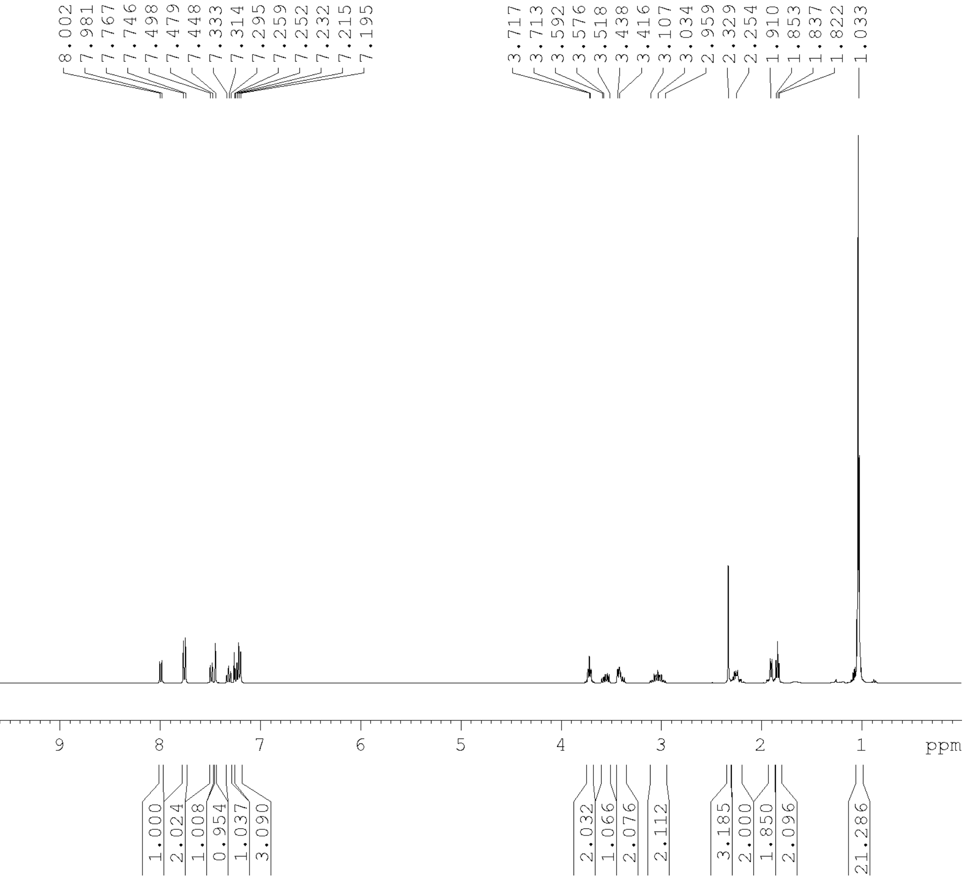
8.002
7.981
7.767
7.746
7.498
7.479
7.448
7.333
7.314
7.295
7.259
7.252
7.232
7.215
7.195
3.717
3.713
3.592
3.576
3.518
3.438
3.416
3.107
3.034
2.959
2.329
2.254
1.910
1.853
1.837
1.822
1.033
11 10 9 8 7 6 5 4 3 2 1 ppm
1.000
2.024
1.008
0.954
1.037
3.090
2.032
1.066
2.076
2.112
3.185
2.000
1.850
2.096
21.286

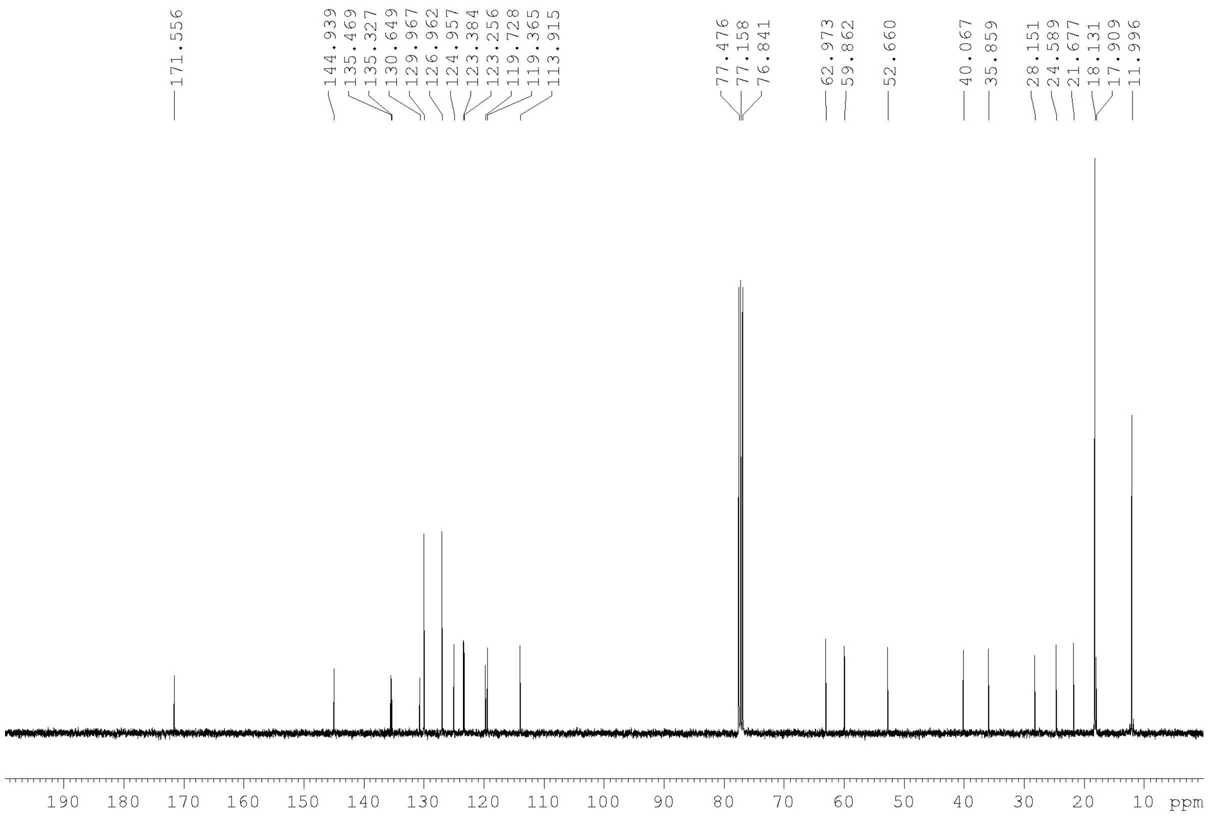
171.556
144.939
135.469
135.327
130.649
129.967
126.962
124.957
123.384
123.256
119.728
119.365
113.915
77.476
77.158
76.841
62.973
59.862
52.660
40.067
35.859
28.151
24.589
21.677
18.131
17.909
11.996
190 180 170 160 150 140 130 120 110 100 90 80 70 60 50 40 30 20 10 ppm

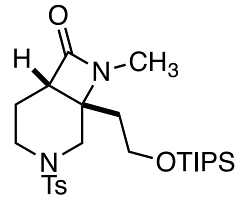
O
H
N
CH_3
OTIPS
N
Ts

2y

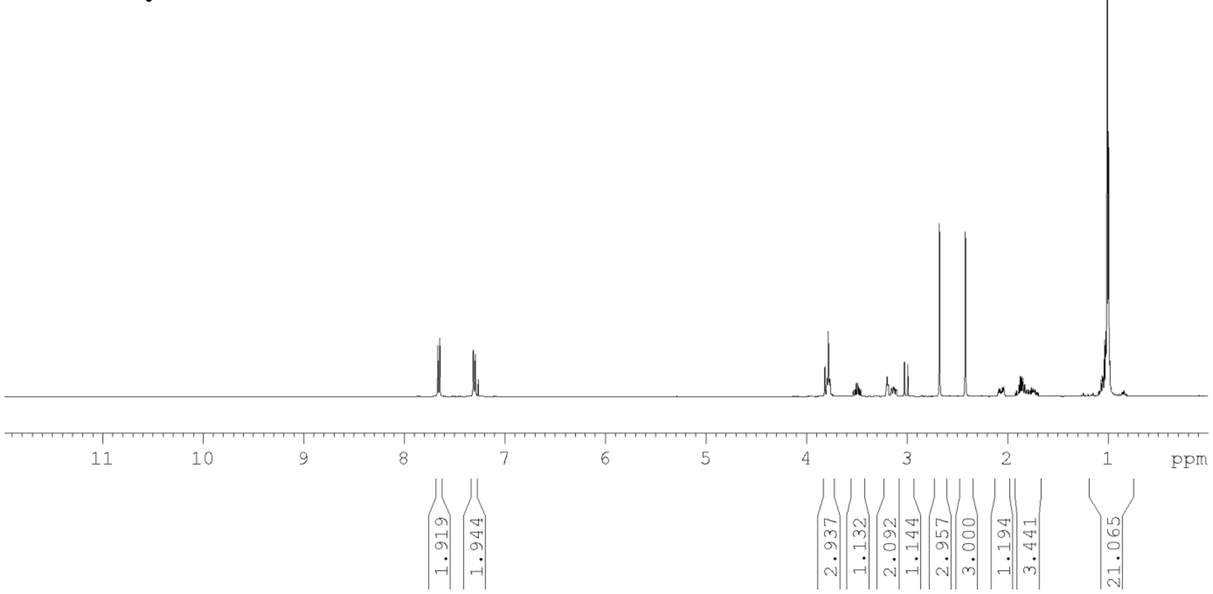
7.664
7.643
7.310
7.290
7.260
3.812
3.778
3.744
3.528
3.454
3.191
3.151
3.100
3.020
2.986
2.671
2.413
2.090
2.030
1.916
1.867
1.854
1.838
1.824
1.705
1.030
1.002
0.990
11
10
9
8
7
6
5
4
3
2
1
ppm
1.919
1.944
2.937
1.132
2.092
1.144
2.957
3.000
1.194
3.441
21.065

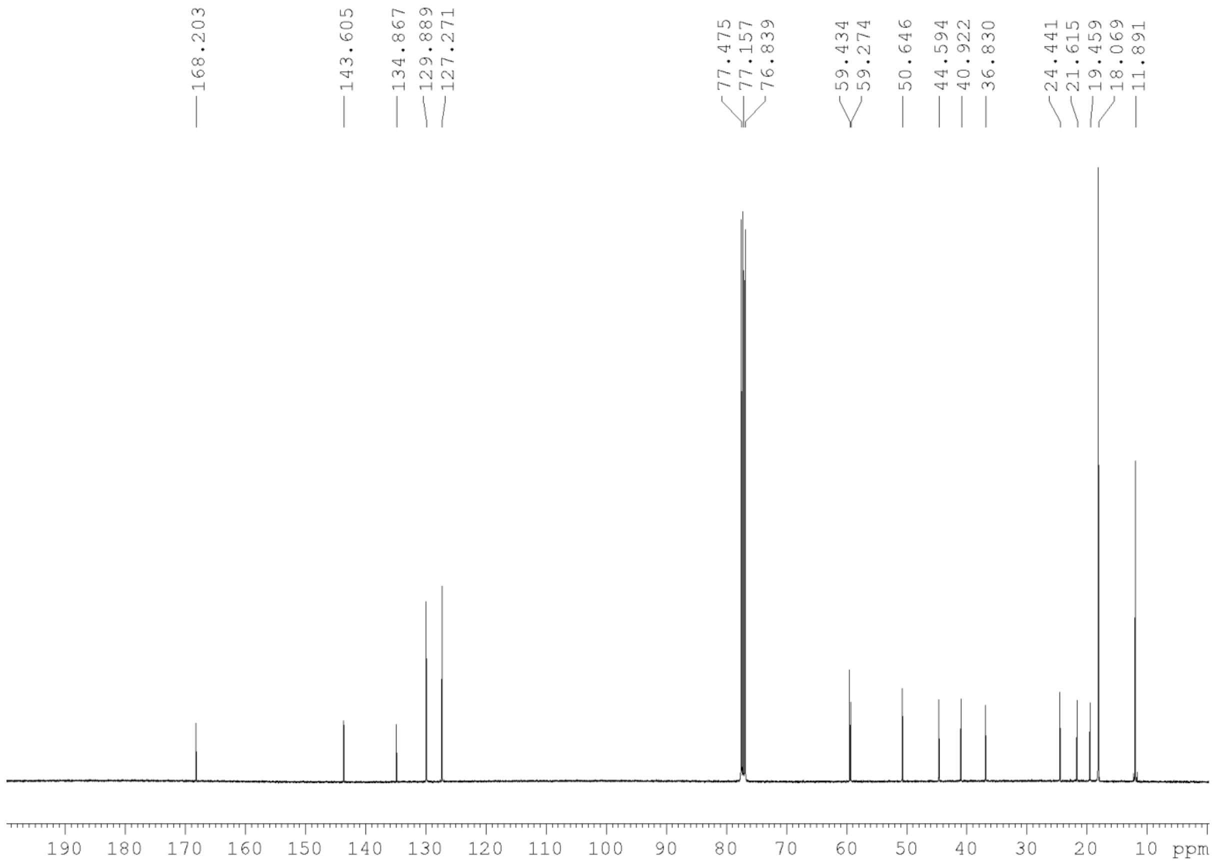
168.203
143.605
134.867
129.889
127.271
77.475
77.157
76.839
59.434
59.274
50.646
44.594
40.922
36.830
24.441
21.615
19.459
18.069
11.891
190
180
170
160
150
140
130
120
110
100
90
80
70
60
50
40
30
20
10
ppm

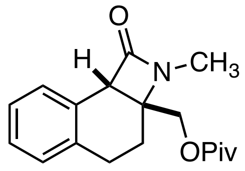
O
H
N
CH3
OPiv

2z

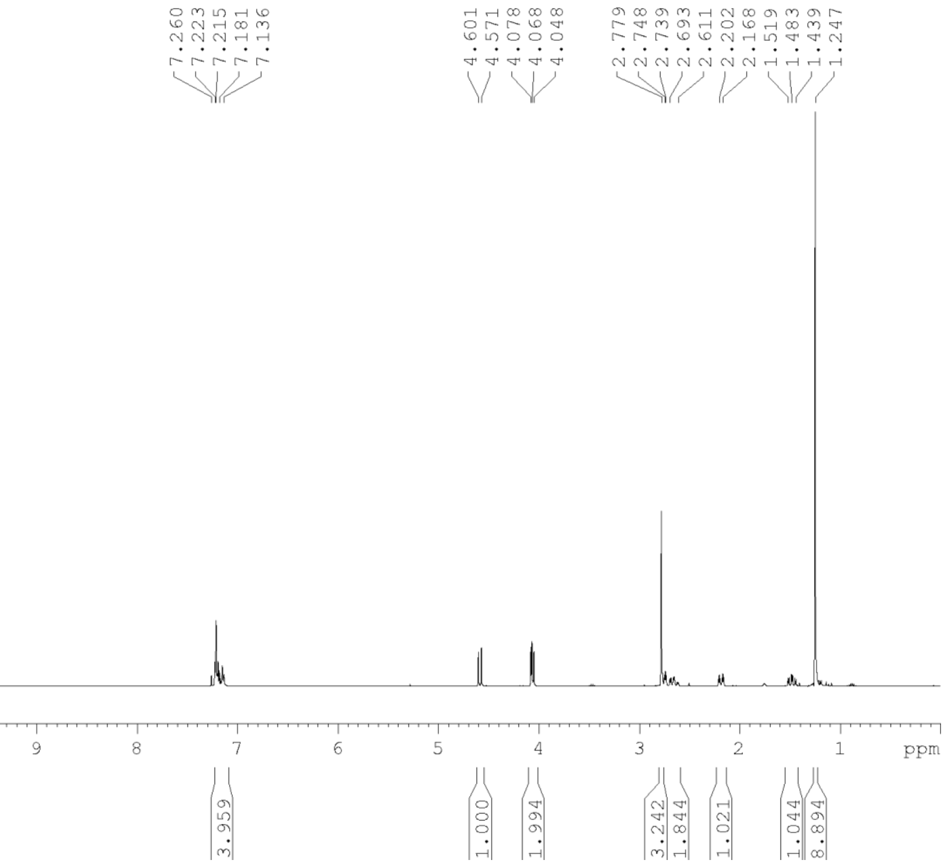
7.260
7.223
7.215
7.181
7.136
4.601
4.571
4.078
4.068
4.048
2.779
2.748
2.739
2.693
2.611
2.202
2.168
1.519
1.483
1.439
1.247
11 10 9 8 7 6 5 4 3 2 1 ppm
3.959
1.000
1.994
3.242
1.844
1.021
1.044
8.894

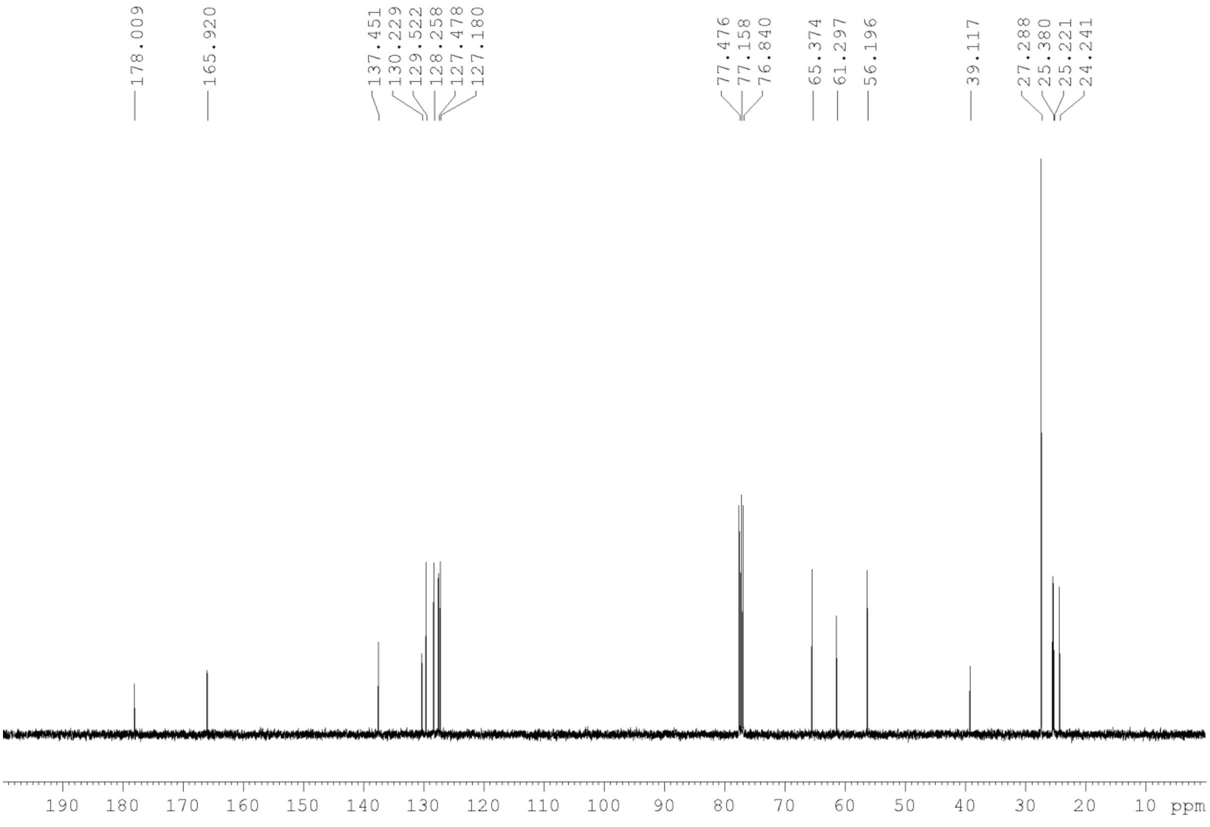
178.009
165.920
137.451
130.229
129.522
128.258
127.478
127.180
77.476
77.158
76.840
65.374
61.297
56.196
39.117
27.288
25.380
25.221
24.241
190 180 170 160 150 140 130 120 110 100 90 80 70 60 50 40 30 20 10 ppm

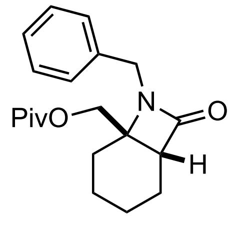
PivO
N
O
H

2aa

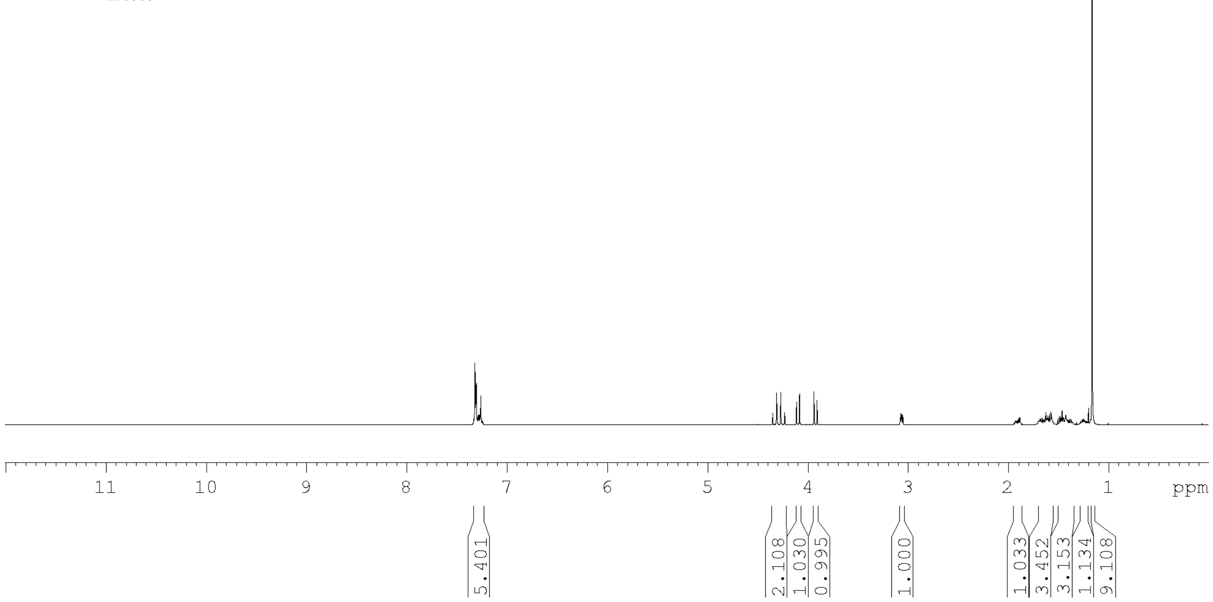
7.332
7.318
7.312
7.304
7.272
7.260
7.241
4.347
4.309
4.267
4.229
4.110
4.080
3.936
3.906
3.076
3.067
3.060
3.050
1.941
1.892
1.883
1.698
1.547
1.502
1.425
1.320
1.263
1.201
1.163
11 10 9 8 7 6 5 4 3 2 1 ppm
5.401
2.108
1.030
0.995
1.000
1.033
3.452
3.153
1.134
9.108

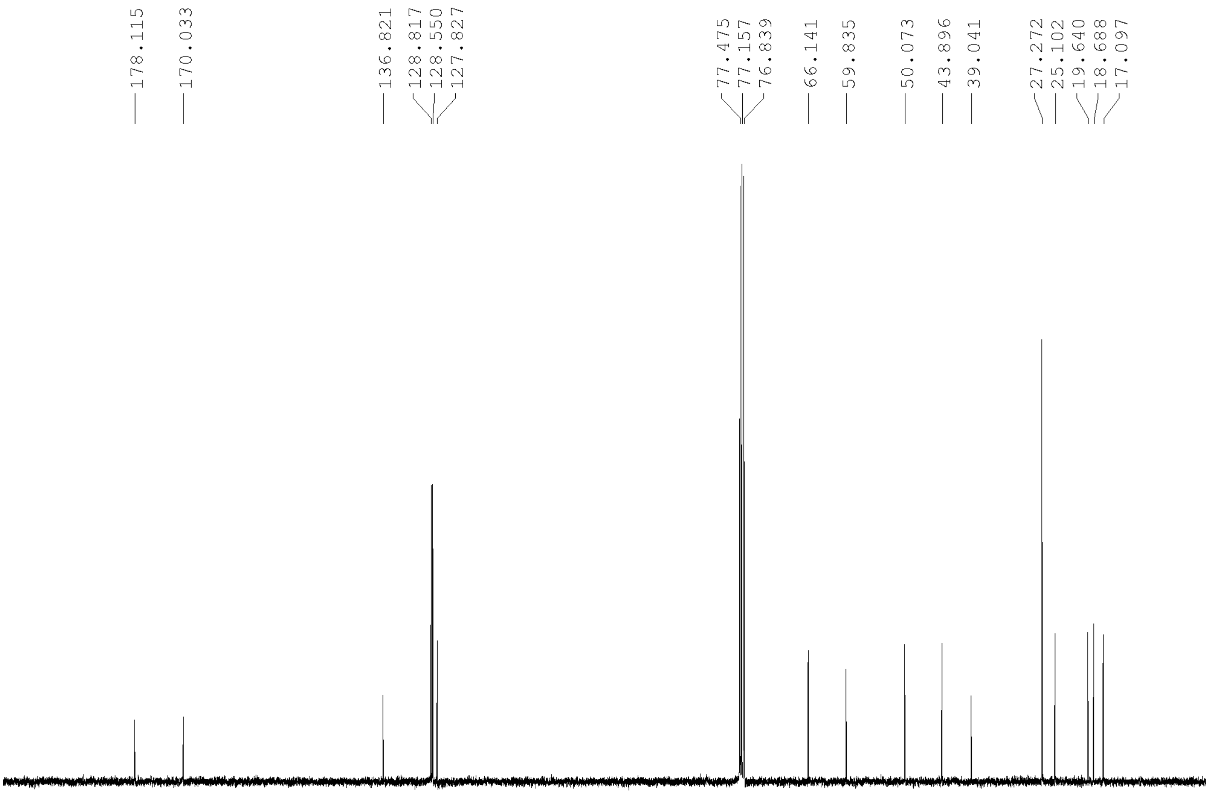
178.115
170.033
136.821
128.817
128.550
127.827
77.475
77.157
76.839
66.141
59.835
50.073
43.896
39.041
27.272
25.102
19.640
18.688
17.097
190 180 170 160 150 140 130 120 110 100 90 80 70 60 50 40 30 20 10 ppm

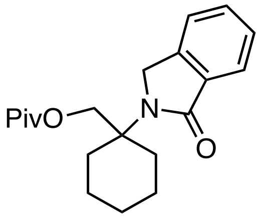
PivO
N
O

3aa

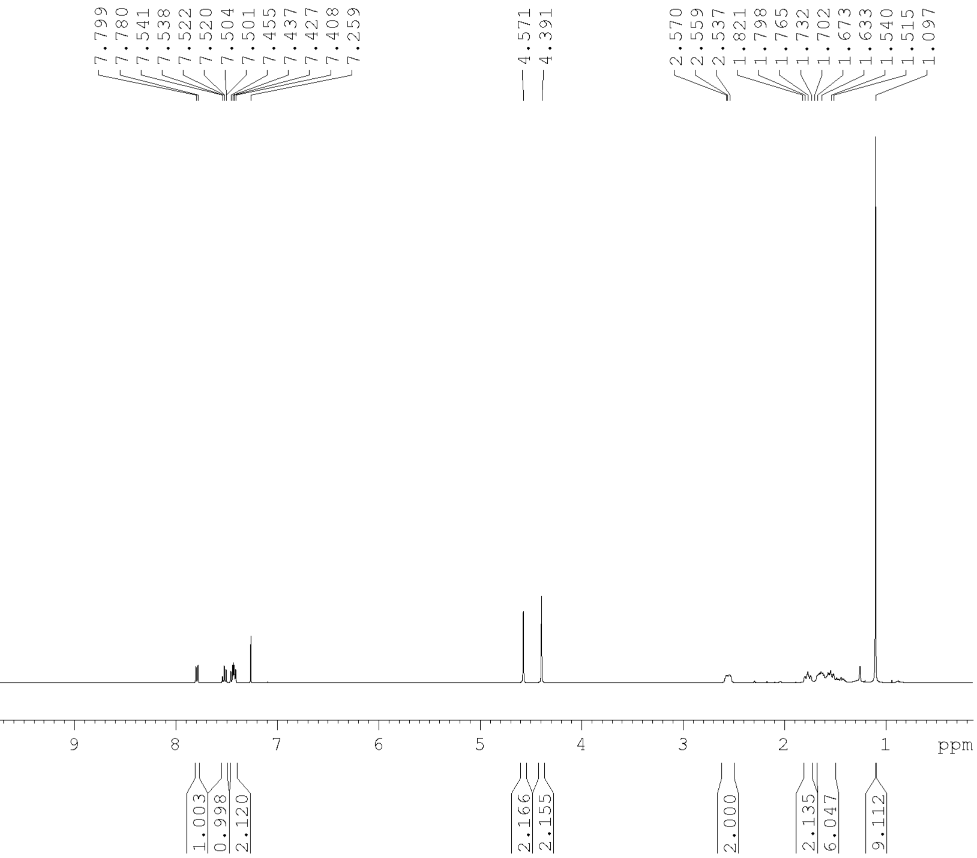
7.799
7.780
7.541
7.538
7.522
7.520
7.504
7.501
7.455
7.437
7.427
7.408
7.259
4.571
4.391
2.570
2.559
2.537
1.821
1.798
1.765
1.732
1.702
1.673
1.633
1.540
1.515
1.097
11 10 9 8 7 6 5 4 3 2 1 ppm
1.003
0.998
2.120
2.166
2.155
2.000
2.135
6.047
9.112

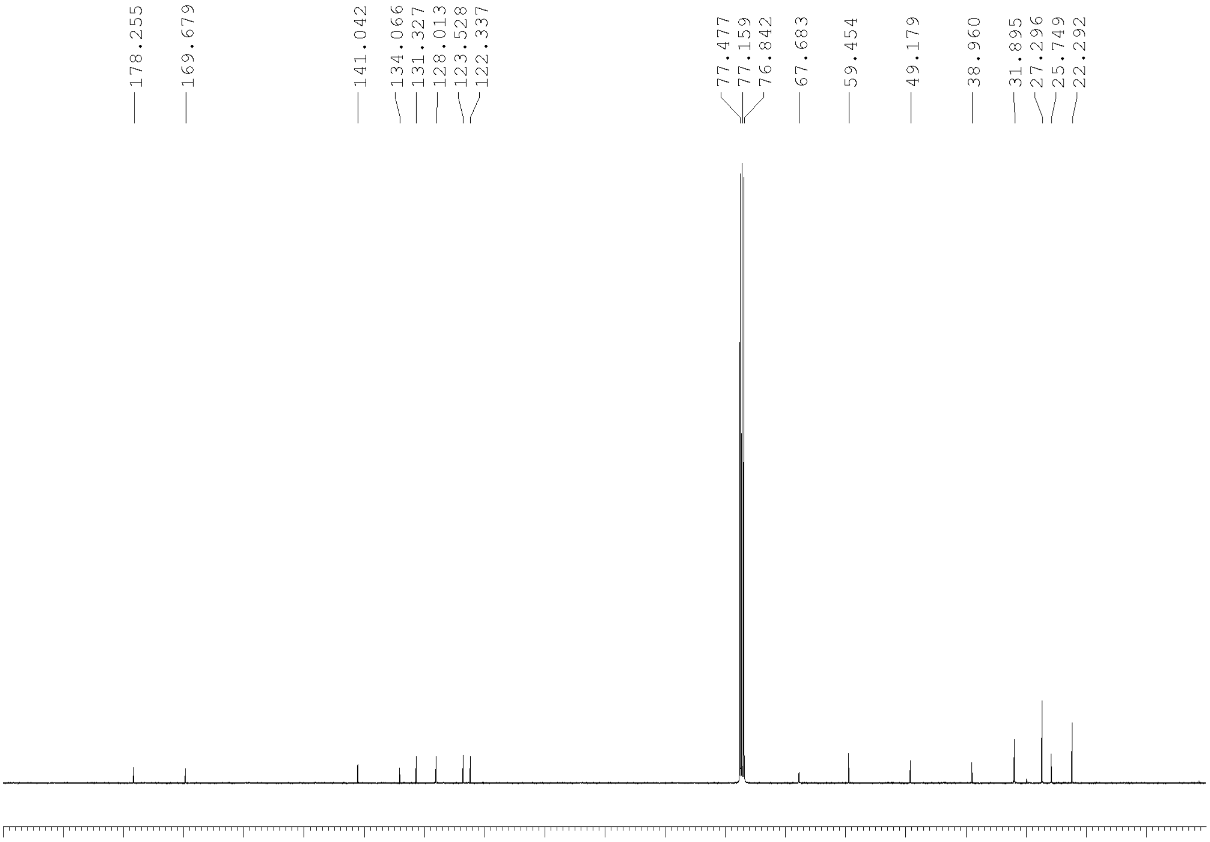
178.255
169.679
141.042
134.066
131.327
128.013
123.528
122.337
77.477
77.159
76.842
67.683
59.454
49.179
38.960
31.895
27.296
25.749
22.292
190 180 170 160 150 140 130 120 110 100 90 80 70 60 50 40 30 20 10 ppm

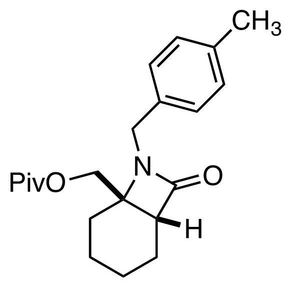
CH3
N
O
PivO
H

2ab

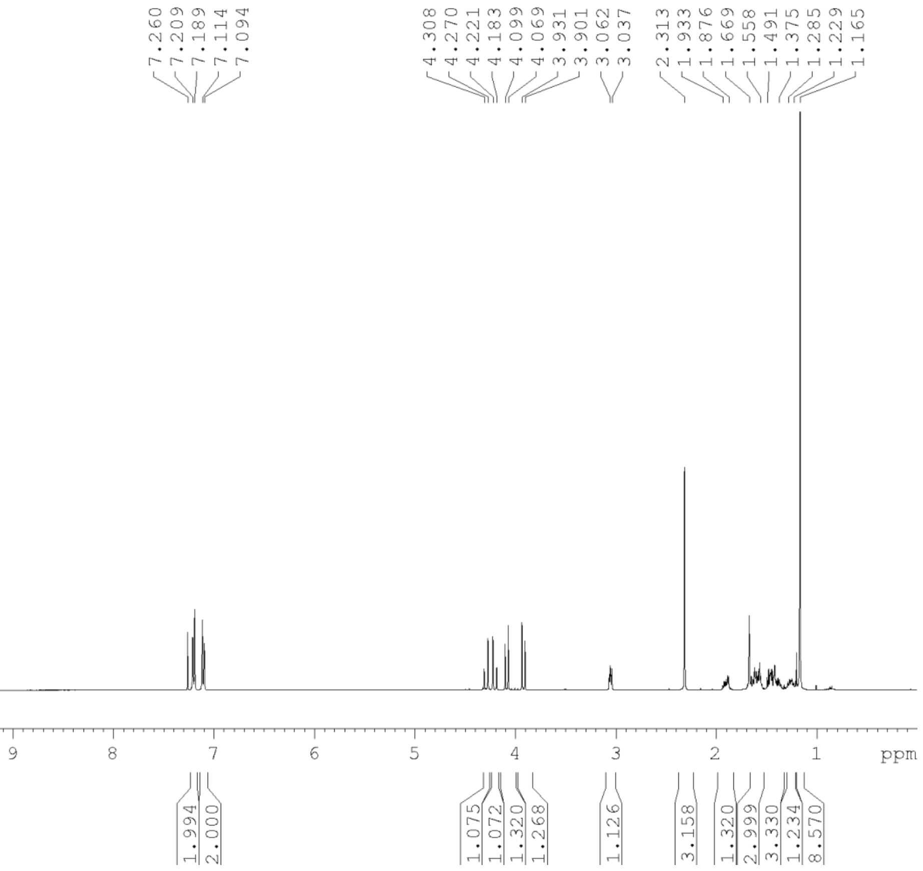
7.260
7.209
7.189
7.114
7.094
4.308
4.270
4.221
4.183
4.099
4.069
3.931
3.901
3.062
3.037
2.313
1.933
1.876
1.669
1.558
1.491
1.375
1.285
1.229
1.165
11
10
9
8
7
6
5
4
3
2
1
ppm
1.994
2.000
1.075
1.072
1.320
1.268
1.126
3.158
1.320
2.999
3.330
1.234
8.570

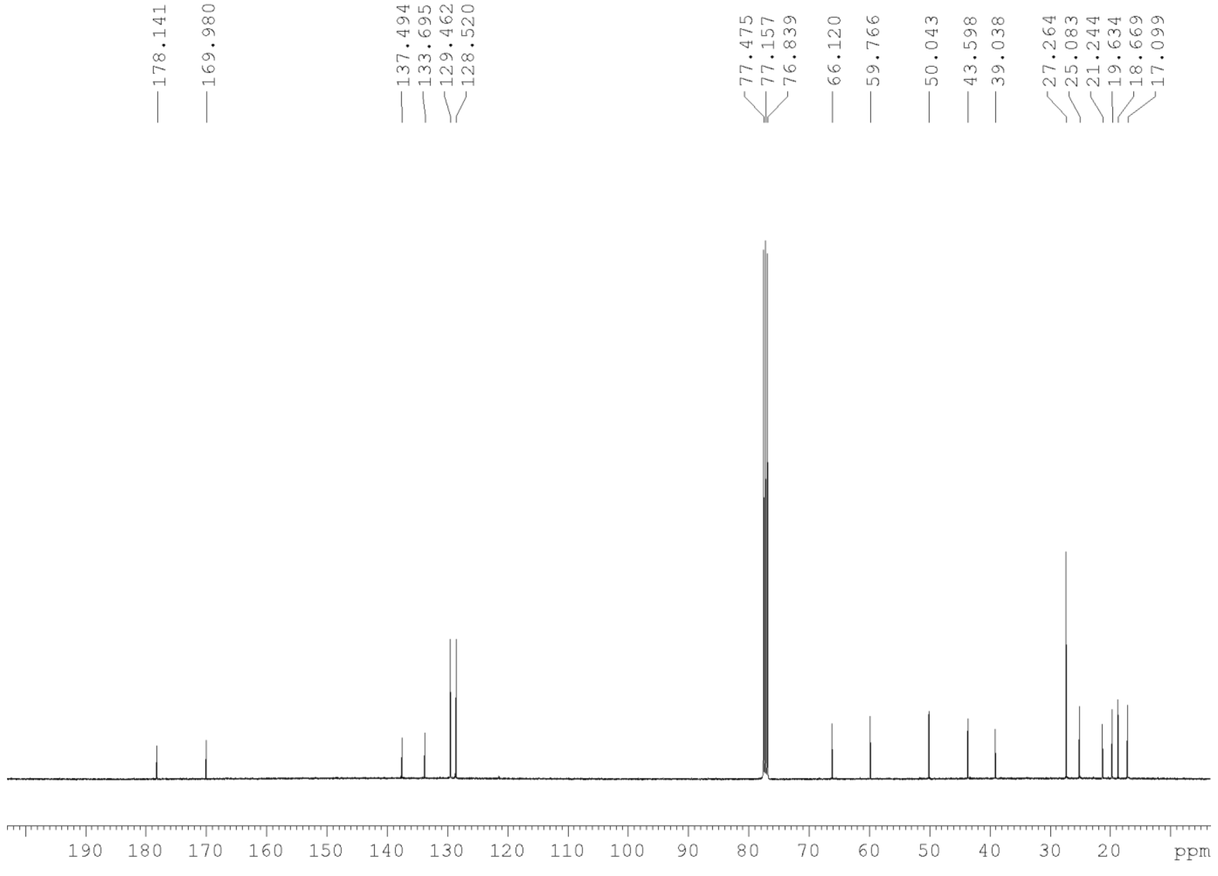
178.141
169.980
137.494
133.695
129.462
128.520
77.475
77.157
76.839
66.120
59.766
50.043
43.598
39.038
27.264
25.083
21.244
19.634
18.669
17.099
190
180
170
160
150
140
130
120
110
100
90
80
70
60
50
40
30
20
ppm

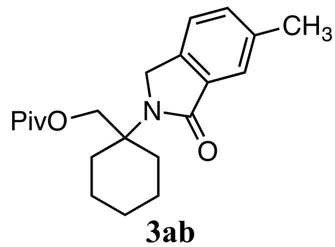
PivO
N
O
CH3

3ab

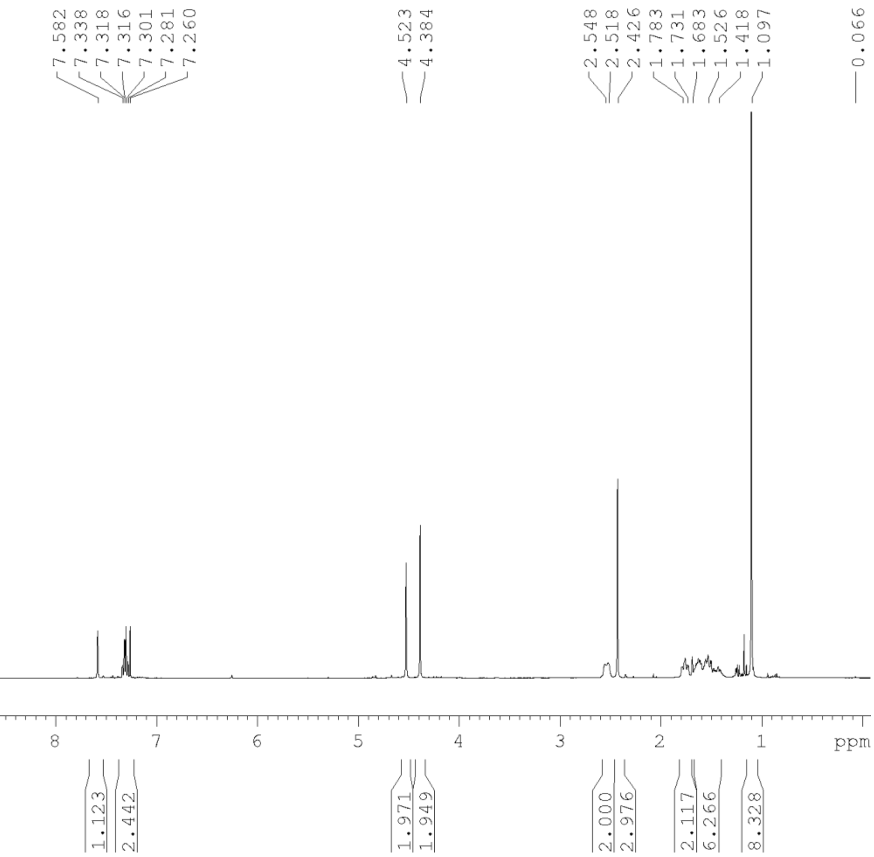
7.582
7.338
7.318
7.316
7.301
7.281
7.260
4.523
4.384
2.548
2.518
2.426
1.783
1.731
1.683
1.526
1.418
1.097
0.066
11 10 9 8 7 6 5 4 3 2 1 ppm
1.123
2.442
1.971
1.949
2.000
2.976
2.117
6.266
8.328

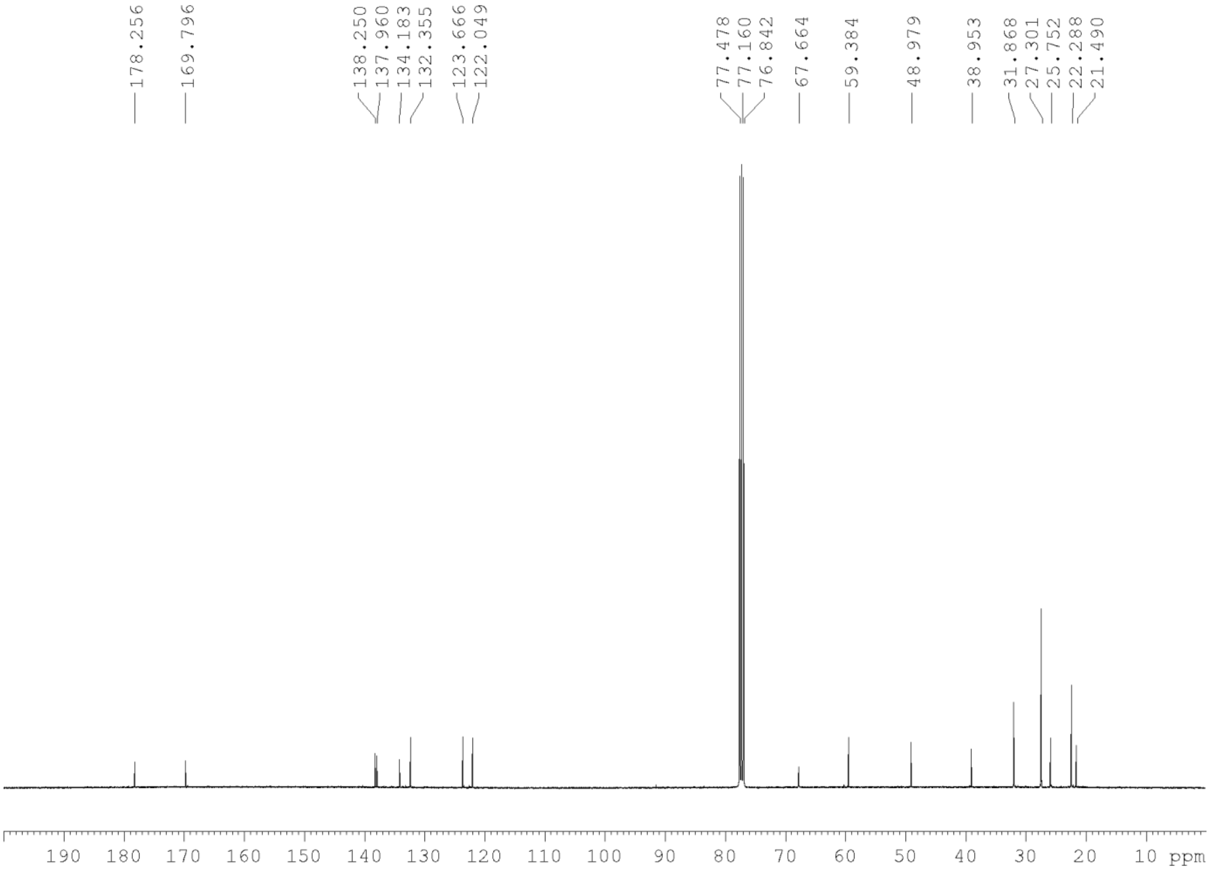
178.256
169.796
138.250
137.960
134.183
132.355
123.666
122.049
77.478
77.160
76.842
67.664
59.384
48.979
38.953
31.868
27.301
25.752
22.288
21.490
190 180 170 160 150 140 130 120 110 100 90 80 70 60 50 40 30 20 10 ppm

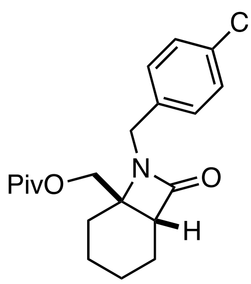
Cl
PivO
N
O
H

2ac

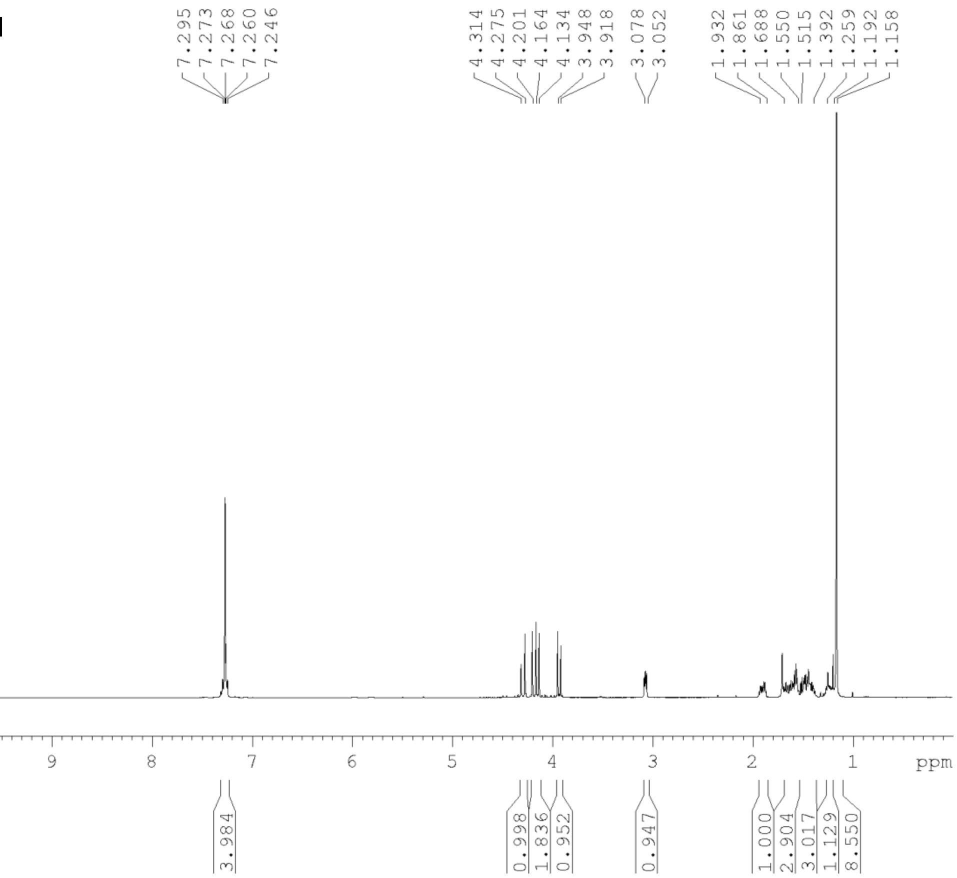
7.295
7.273
7.268
7.260
7.246
4.314
4.275
4.201
4.164
4.134
3.948
3.918
3.078
3.052
1.932
1.861
1.688
1.550
1.515
1.392
1.259
1.192
1.158
11 10 9 8 7 6 5 4 3 2 1 ppm
3.984
0.998
1.836
0.952
0.947
1.000
2.904
3.017
1.129
8.550

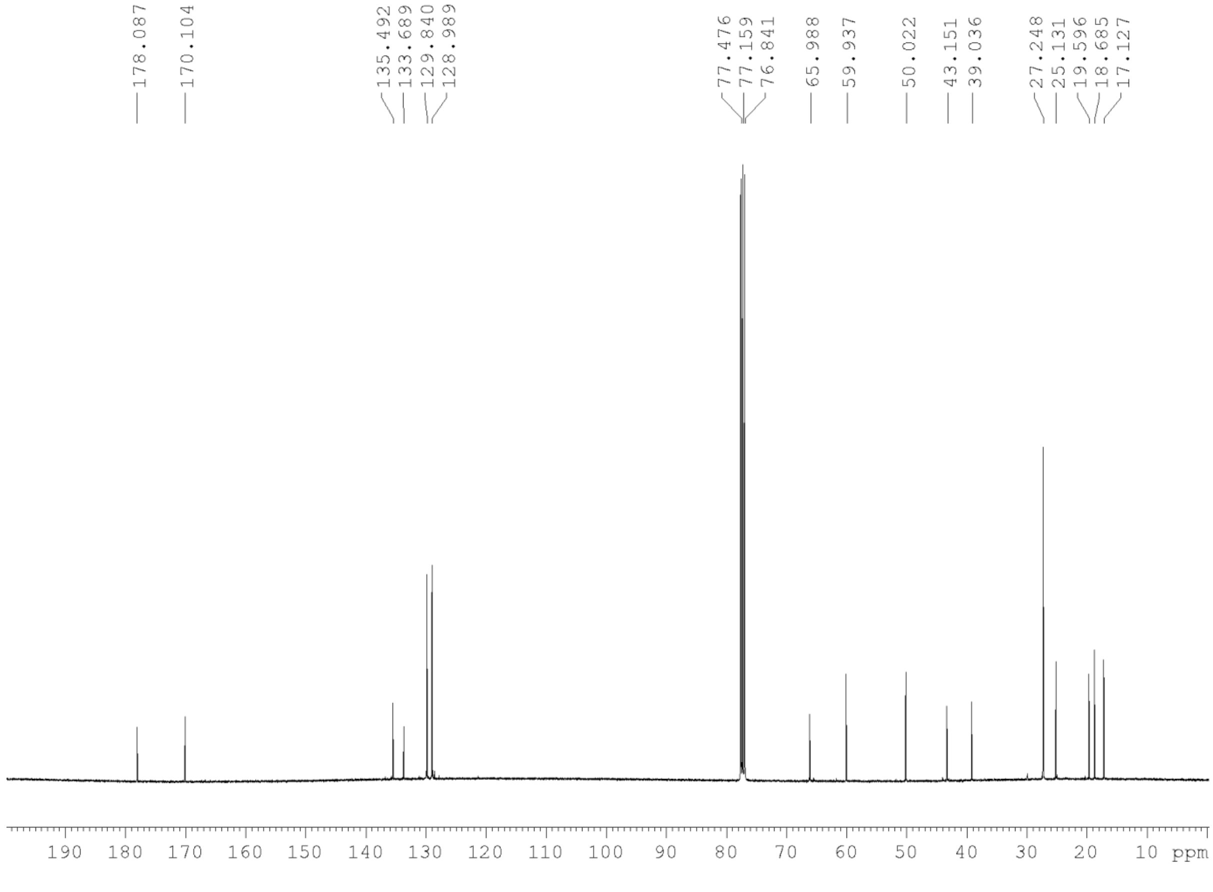
178.087
170.104
135.492
133.689
129.840
128.989
77.476
77.159
76.841
65.988
59.937
50.022
43.151
39.036
27.248
25.131
19.596
18.685
17.127
190 180 170 160 150 140 130 120 110 100 90 80 70 60 50 40 30 20 10 ppm

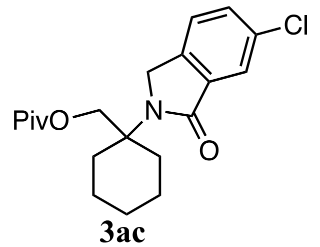
Cl
N
O
PivO

3ac

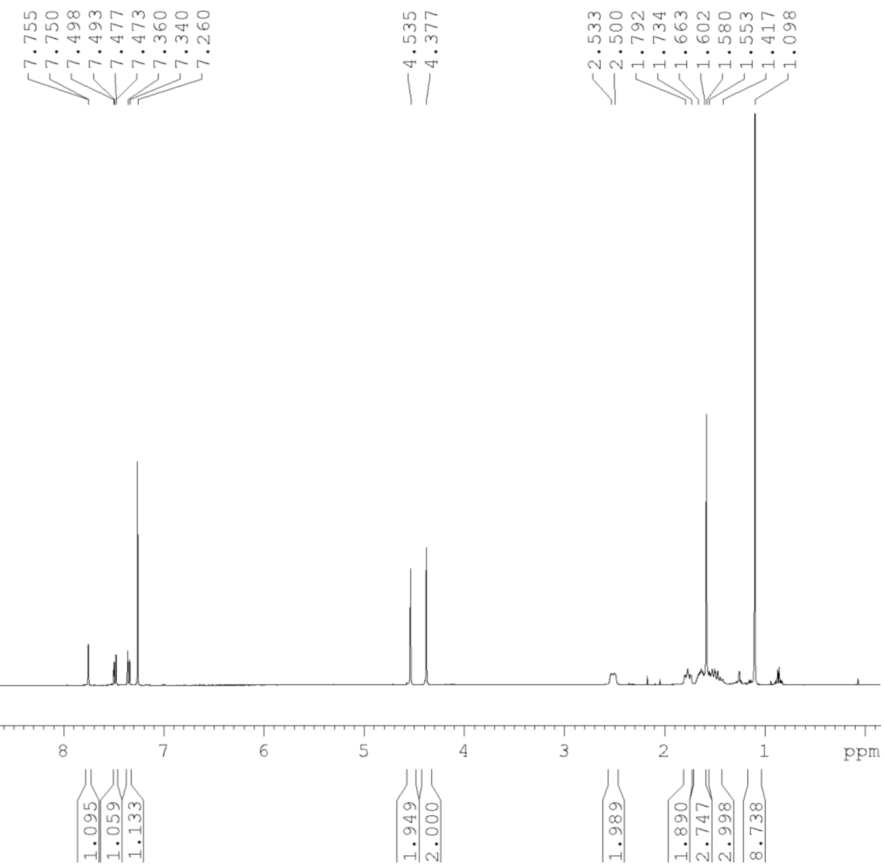
7.755
7.750
7.498
7.493
7.477
7.473
7.360
7.340
7.260
4.535
4.377
2.533
2.500
1.792
1.734
1.663
1.602
1.580
1.553
1.417
1.098
11 10 9 8 7 6 5 4 3 2 1 ppm
1.095
1.059
1.133
1.949
2.000
1.989
1.890
2.747
2.998
8.738

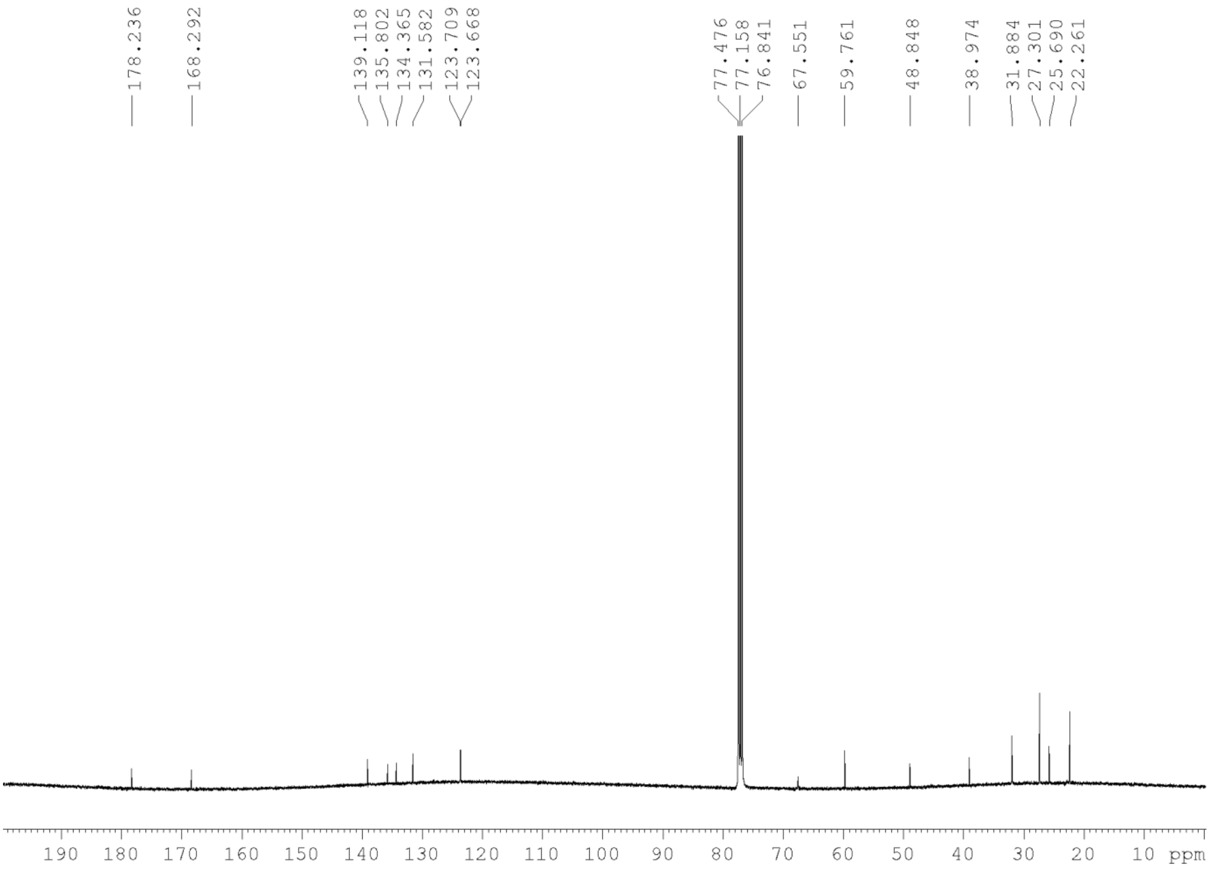
178.236
168.292
139.118
135.802
134.365
131.582
123.709
123.668
77.476
77.158
76.841
67.551
59.761
48.848
38.974
31.884
27.301
25.690
22.261
190 180 170 160 150 140 130 120 110 100 90 80 70 60 50 40 30 20 10 ppm

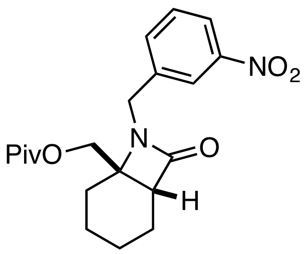
NO2
PivO
N
O
H

2ad

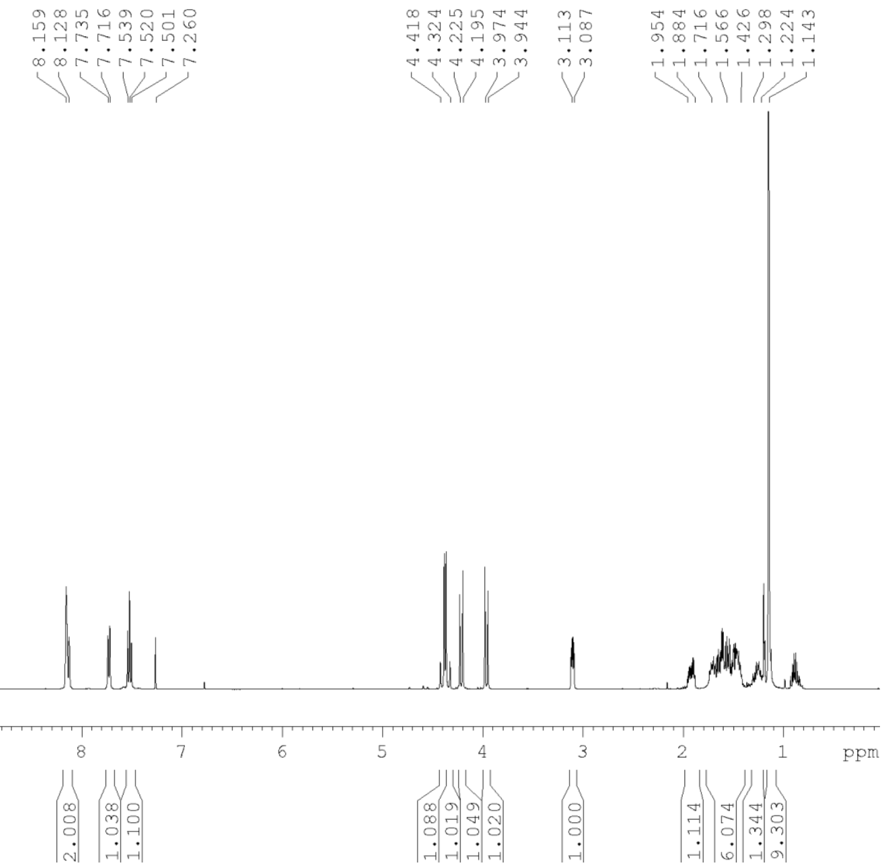
8.159
8.128
7.735
7.716
7.539
7.520
7.501
7.260
4.418
4.324
4.225
4.195
3.974
3.944
3.113
3.087
1.954
1.884
1.716
1.566
1.426
1.298
1.224
1.143
11
10
9
8
7
6
5
4
3
2
1
ppm
2.008
1.038
1.100
1.088
1.019
1.049
1.020
1.000
1.114
6.074
1.344
9.303

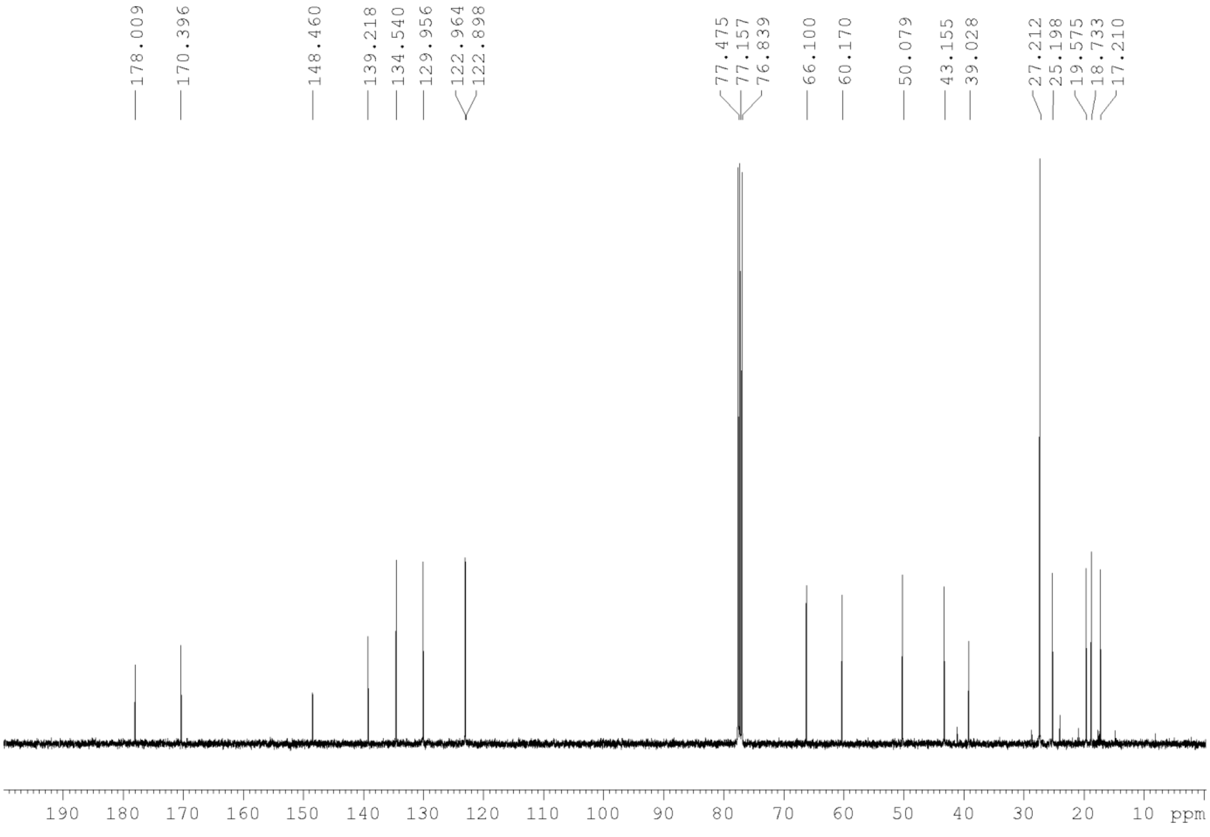
178.009
170.396
148.460
139.218
134.540
129.956
122.964
122.898
77.475
77.157
76.839
66.100
60.170
50.079
43.155
39.028
27.212
25.198
19.575
18.733
17.210
190
180
170
160
150
140
130
120
110
100
90
80
70
60
50
40
30
20
10
ppm

NMR of Functionalized Products

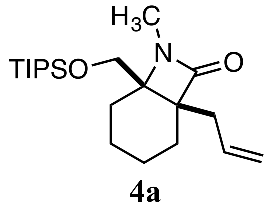
H3C
N
O
TIPSO

4a

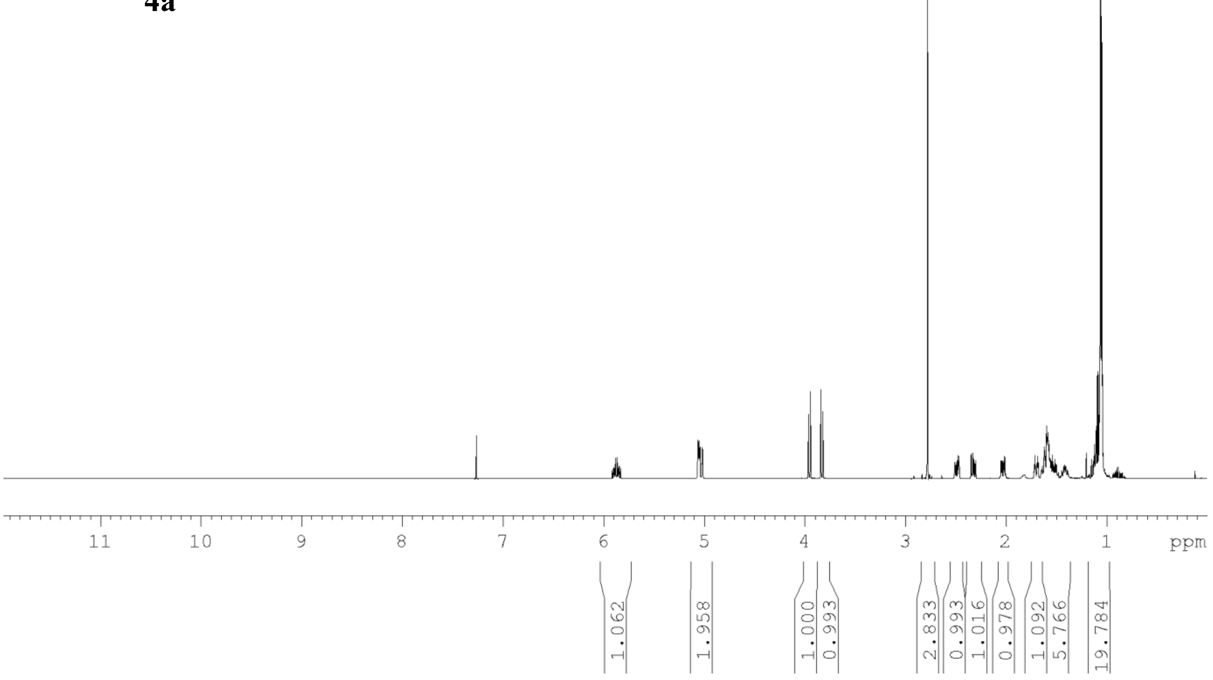
7.262
5.912
5.879
5.864
5.829
5.059
5.017
3.961
3.941
3.838
3.817
2.777
2.506
2.464
2.343
2.298
2.046
2.010
1.712
1.684
1.651
1.497
1.441
1.386
1.092
1.047
11 10 9 8 7 6 5 4 3 2 1 ppm
1.062
1.958
1.000
0.993
2.833
0.993
1.016
0.978
1.092
5.766
19.784

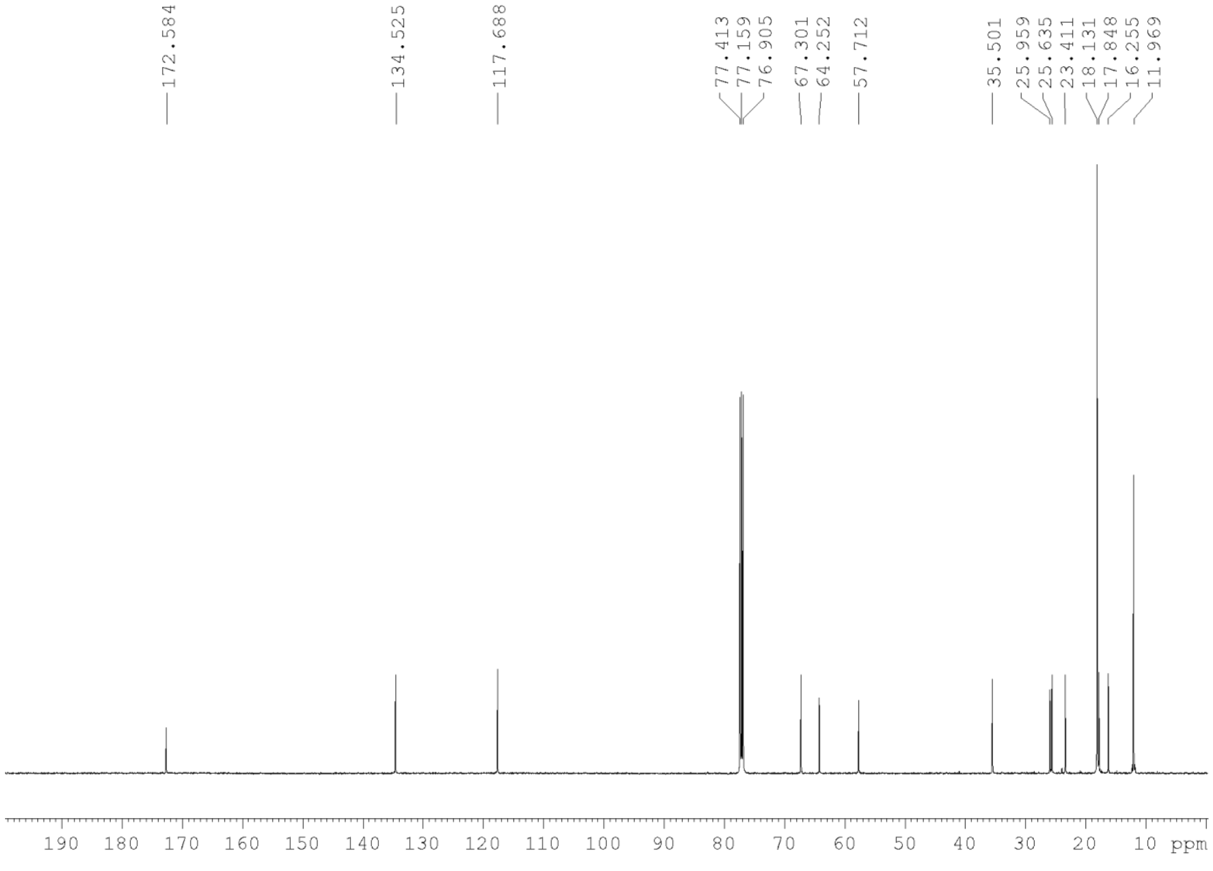
172.584
134.525
117.688
77.413
77.159
76.905
67.301
64.252
57.712
35.501
25.959
25.635
23.411
18.131
17.848
16.255
11.969
190 180 170 160 150 140 130 120 110 100 90 80 70 60 50 40 30 20 10 ppm

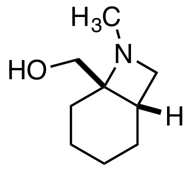
H3C
N
HO
H

4b

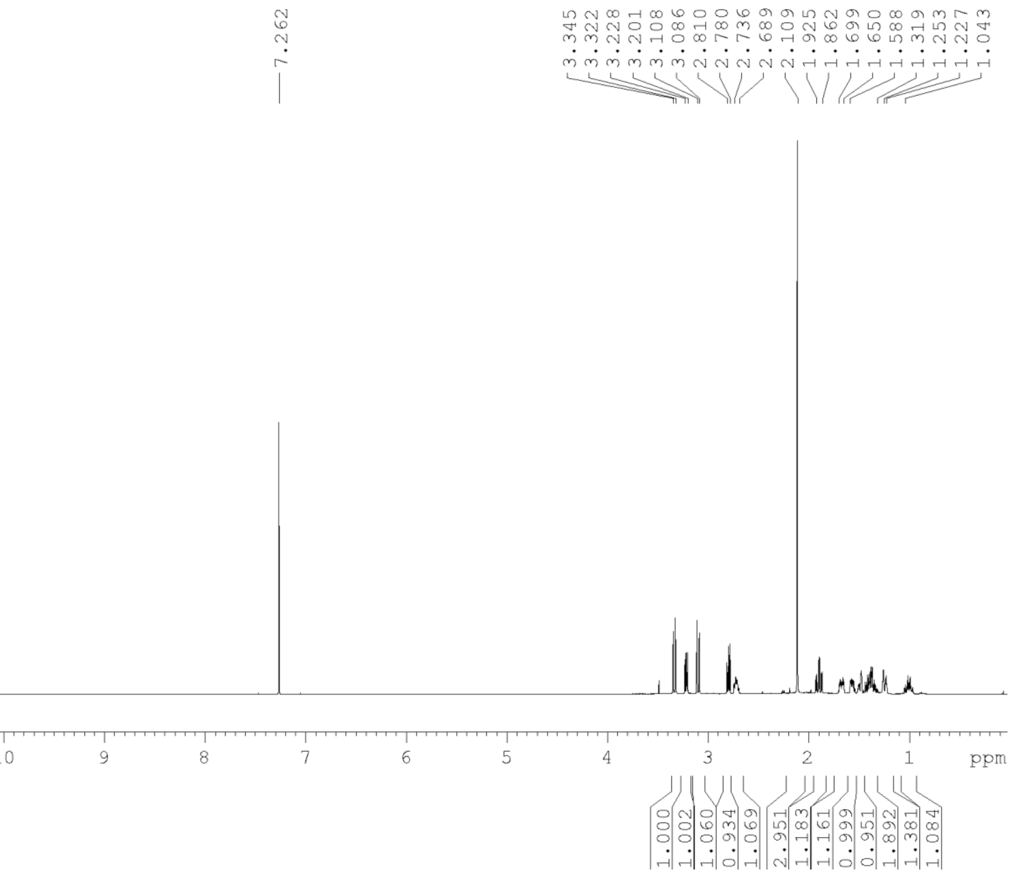
7.262
3.345
3.322
3.228
3.201
3.108
3.086
2.810
2.780
2.736
2.689
2.109
1.925
1.862
1.699
1.650
1.588
1.319
1.253
1.227
1.043
11 10 9 8 7 6 5 4 3 2 1 ppm
1.000
1.002
1.060
0.934
1.069
2.951
1.183
1.161
0.999
0.951
1.892
1.381
1.084

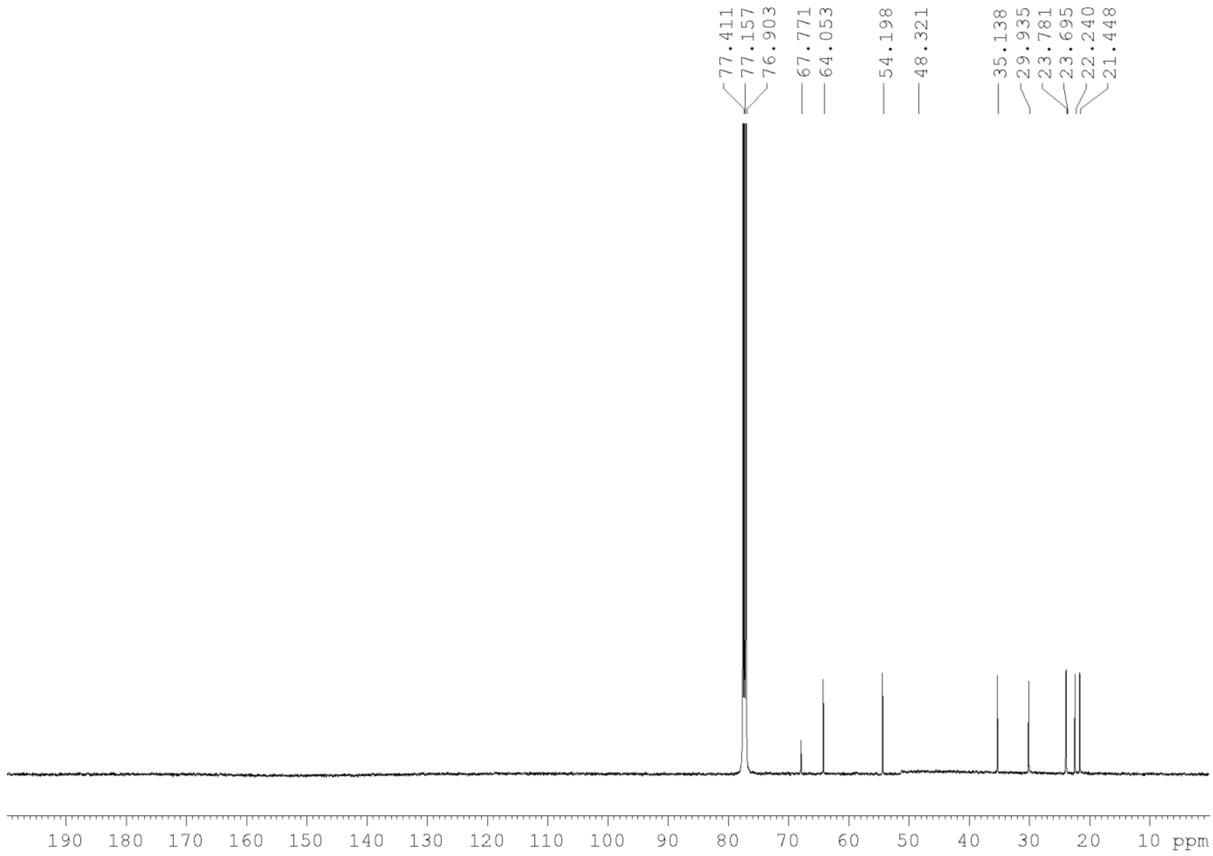
77.411
77.157
76.903
67.771
64.053
54.198
48.321
35.138
29.935
23.781
23.695
22.240
21.448
190 180 170 160 150 140 130 120 110 100 90 80 70 60 50 40 30 20 10 ppm

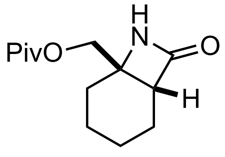
PivO
H
N
O
H

4c

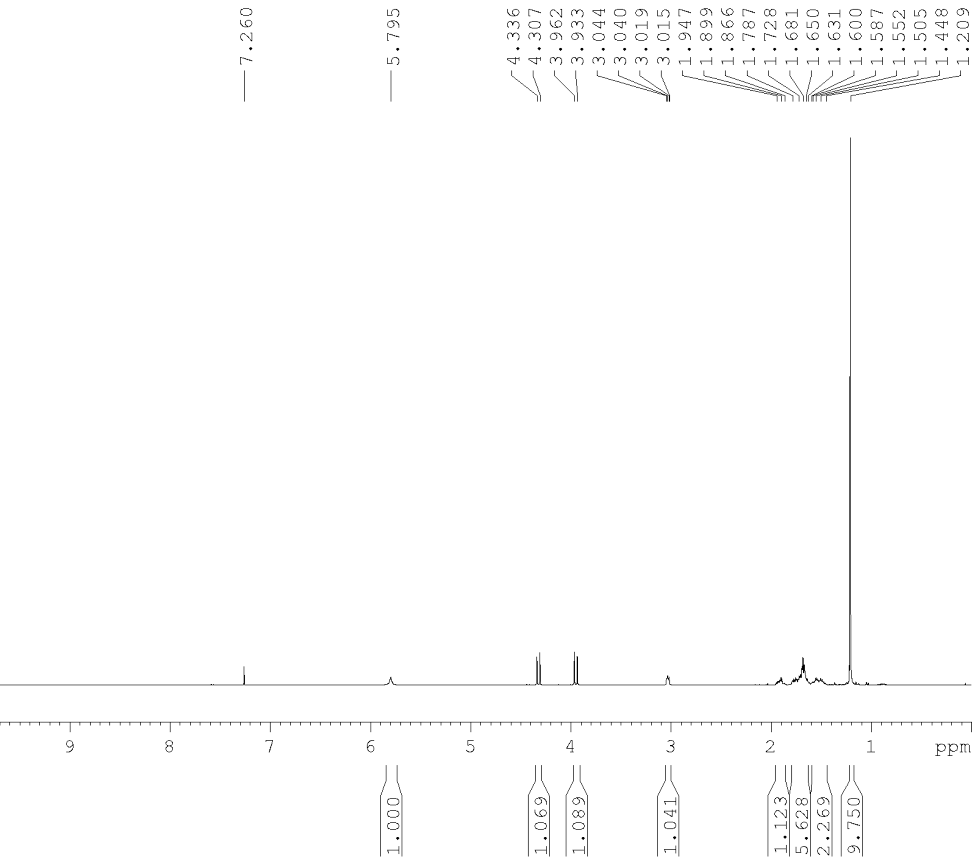
7.260
5.795
4.336
4.307
3.962
3.933
3.044
3.040
3.019
3.015
1.947
1.899
1.866
1.787
1.728
1.681
1.650
1.631
1.600
1.587
1.552
1.505
1.448
1.209
11 10 9 8 7 6 5 4 3 2 1 ppm
1.000
1.069
1.089
1.041
1.123
5.628
2.269
9.750

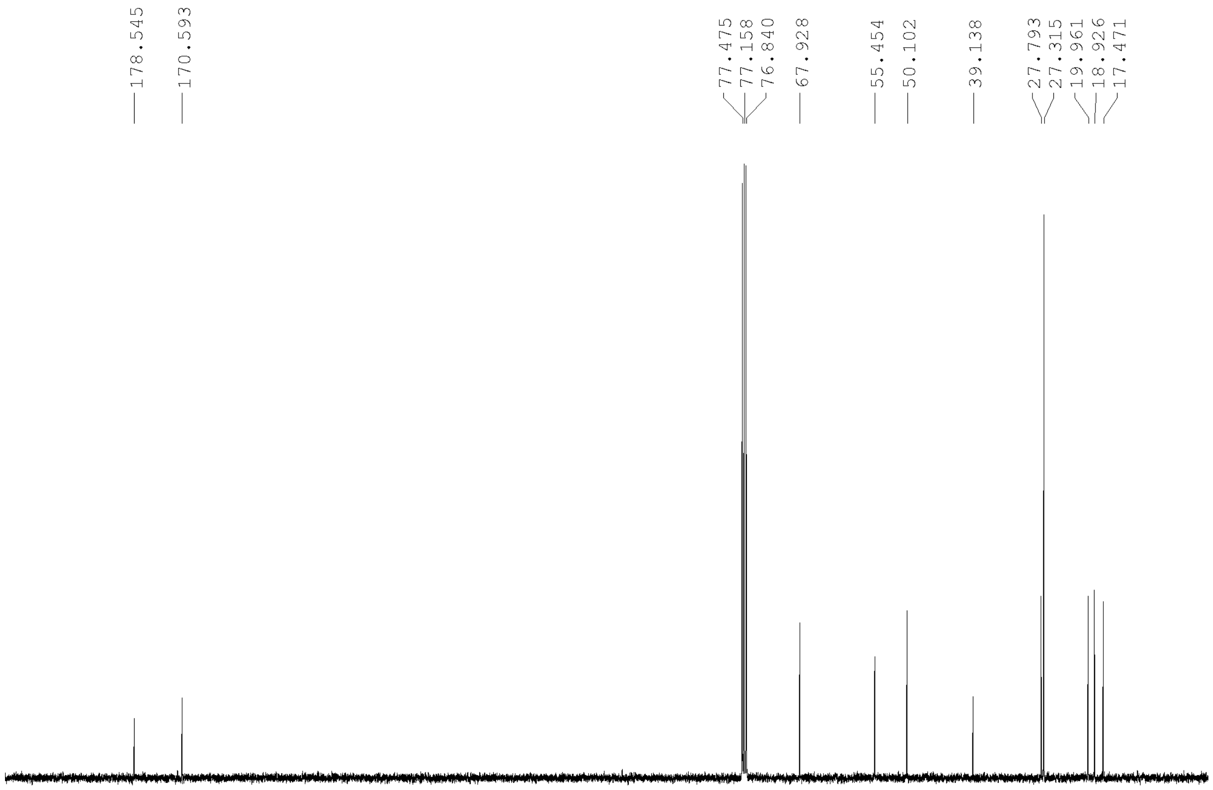
178.545
170.593
77.475
77.158
76.840
67.928
55.454
50.102
39.138
27.793
27.315
19.961
18.926
17.471
190 180 170 160 150 140 130 120 110 100 90 80 70 60 50 40 30 20 10 ppm
